# An in-silico approach for discovery of microRNA-TF regulation of DISC1 interactome mediating neuronal migration

**DOI:** 10.1038/s41540-019-0094-3

**Published:** 2019-05-07

**Authors:** John P. John, Priyadarshini Thirunavukkarasu, Koko Ishizuka, Pravesh Parekh, Akira Sawa

**Affiliations:** 1 Multimodal Brain Image Analysis Laboratory (MBIAL), National Institute of Mental Health and Neurosciences (NIMHANS), Bangalore, 560029 India; 2 Department of Psychiatry, National Institute of Mental Health and Neurosciences (NIMHANS), Bangalore, 560029 India; 3 Department of Psychiatry and Behavioral Sciences, School of Medicine, Johns Hopkins University, Baltimore, MD 21287 USA; 4 Departments of Psychiatry, Mental Health, Neuroscience, and Biomedical Engineering, School of Medicine, Bloomberg School of Public Health, Johns Hopkins University, Baltimore, MD 21287 USA

**Keywords:** Computational biology and bioinformatics, Neuroscience, Logic gates

## Abstract

Neuronal migration constitutes an important step in corticogenesis; dysregulation of the molecular mechanisms mediating this crucial step in neurodevelopment may result in various neuropsychiatric disorders. By curating experimental data from published literature, we identified eight functional modules involving Disrupted-in-schizophrenia 1 (DISC1) and its interacting proteins that regulate neuronal migration. We then identified miRNAs and transcription factors (TFs) that form functional feedback loops and regulate gene expression of the DISC1 interactome. Using this curated data, we conducted in-silico modeling of the DISC1 interactome involved in neuronal migration and identified the proteins that either facilitate or inhibit neuronal migrational processes. We also studied the effect of perturbation of miRNAs and TFs in feedback loops on the DISC1 interactome. From these analyses, we discovered that STAT3, TCF3, and TAL1 (through feedback loop with miRNAs) play a critical role in the transcriptional control of DISC1 interactome thereby regulating neuronal migration. To the best of our knowledge, regulation of the DISC1 interactome mediating neuronal migration by these TFs has not been previously reported. These potentially important TFs can serve as targets for undertaking validation studies, which in turn can reveal the molecular processes that cause neuronal migration defects underlying neurodevelopmental disorders. This underscores the importance of the use of in-silico techniques in aiding the discovery of mechanistic evidence governing important molecular and cellular processes. The present work is one such step towards the discovery of regulatory factors of the DISC1 interactome that mediates neuronal migration.

## Neuroinformatics: Framework for simulating regulation of neurodevelopment

Computer simulation (in-silico) methods offer a potent approach to deal with the enormous complexity of regulatory interactions mediating neurodevelopment. John P. John and Priyadarshini Thirunavukkarasu (National Institute of Mental Health and Neurosciences, India) and Akira Sawa (Johns Hopkins University, USA), along with their colleagues, developed an in-silico simulation of the regulation of genes interacting with Disrupted-in-schizophrenia 1 gene (the DISC1 interactome) that mediate neuronal migration. Through in-silico perturbation of microRNAs and transcription factors (TFs), they discovered three TFs, namely, STAT3, TCF3 and TAL1, that play a critical role in the regulation of the DISC1 interactome. Further experimental validation studies can confirm the role of these TFs in neuronal migration. This framework for discovery of critical factors that can be targets for in-vivo studies may aid in advancing our understanding regarding molecular pathophysiology of neurodevelopmental disorders.

## Introduction

Neuronal migration is one of the most crucial steps of neurodevelopment. Any disturbance of this process can lead to cortical dysgenesis, i.e., abnormal development of the cerebral cortex.^1^ Disrupted-in-schizophrenia 1 (DISC1) is an extensively studied molecule in its regulation of neuronal migration and other stages of neurodevelopment, as well as its mediation of higher brain functions.^2^ It performs its role through its interactions with multiple other proteins.^3^ Although genetic implication of *DISC1* has turned out to be very specific to the original Scottish pedigree, and not for sporadic cases of schizophrenia, biological perturbation of DISC1 clearly leads to neurodevelopmental and behavioral deficits.^4^ Therefore, the importance of DISC1, originally proposed on the basis of a rare genetic case, is very high in neurobiology.

There is considerable evidence for interplay between transcriptional and post-transcriptional regulators of gene expression underlying the molecular pathology of various diseases.^5^ MicroRNA (miRNA) and transcription factors (TF) in miRNA-TF feedback loops strongly regulate each other and many target genes.^5^ Furthermore, miRNAs and TFs in these feedback loops have higher *in-degree* and *out-degree* in comparison to those that are not involved in these feedback loops.^6^ Thus, the miRNA-TF feedback loop is suggested as a common mechanism of gene regulation at a systems level.^7^

Network models that integrate protein−protein interactions (PPIs) involving DISC1 (the DISC1 interactome) and the regulation of expression of genes encoding these proteins by miRNA-TF feedback loops can advance our understanding of the complex molecular mechanisms that regulate neuronal migration. Several mathematical approaches have been used to model such interactions.^8–11^ Boolean network models are one of the most widely used discrete mathematical models in contexts where the biological kinetic parameters are not known^12^; e.g., colitis-associated colon cancer,^13^ apoptosis,^14^ survival signaling of T-cell large granular lymphocyte leukemia^15^ and p53 regulatory circuit.^16^ Using real-world data, Boolean network models allow simulation of interactions between genes and identification of the most important gene regulatory elements in the network.^17,18^ Multiple regulators determining the activity of a given gene are combined using logical operators and a Boolean function determines the next state of a gene, based on the current state of its regulators. Boolean models provide insight about the dynamics of biological systems such as multiple cell fates and cellular phenotypes.^19^

In this study, we constructed a Boolean network integrating the DISC1 interactome that mediates neuronal migration using experimental evidence curated from relevant databases. We determined the stable-states reached by PPIs between proteins constituting the DISC1 interactome regulating neuronal migration and determined the modes of neuronal migration that are facilitated or inhibited in each stable state. We then demonstrated how perturbation of each miRNA, gene, and TF affects neuronal migration in each functional module (FM). We also examined the influence of miRNA and TF in feedback loops regulating two or more FMs of migration. Finally, we constructed a comprehensive network model of regulation of proteins in the DISC1 interactome by miRNA-TF feedback loops, which can provide a framework for further examination of the molecular mechanisms that regulate neuronal migration in experimental studies.

## Results

### DISC1 protein−protein interactions/functional modules (the DISC1 interactome) involved in neuronal migration

**Fig. 1:**
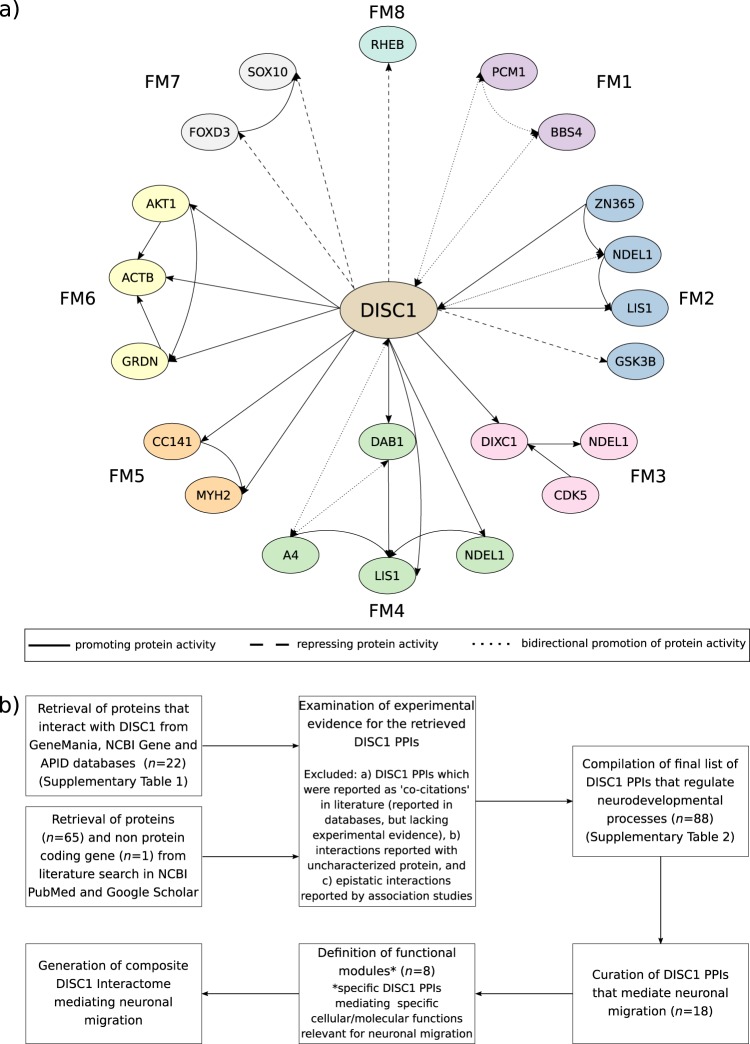
**a** DISC1 interactome that regulates neuronal migration through eight functional modules; **b** Method followed for generating the interactome. DISC1 interacts with 18 proteins forming eight functional modules (FM) that mediate neuronal migration. FM1 mediates radial migration of immature neurons, FM2 mediates radial migration of basal progenitors, FM3 mediates radial migration of newborn neurons, FM4 mediates radial migration of neuronal precursor cells, FM5 mediates radial migration of apical and basal progenitors, FM6 mediates tangential migration of cortical interneurons, FM7 mediates cranial neural crest cell migration and FM8 mediates migration of adult hippocampal progenitors

We observed that DISC1 interacts with 87 proteins as well as with DISC1 fusion partner 1 (*DISC1FP1*) regulating various neurodevelopmental functions (Supplementary Table S1 and Supplementary References). Of these, 18 proteins regulated eight FMs of neuronal migration through DISC1 PPIs. From these PPIs, we generated a composite DISC1 interactome, integrating the above FMs (Fig. 1).

### Identification of miRNA-TF feedback loops that regulate genes encoding the proteins involved in neuronal migration

**Fig. 2:**
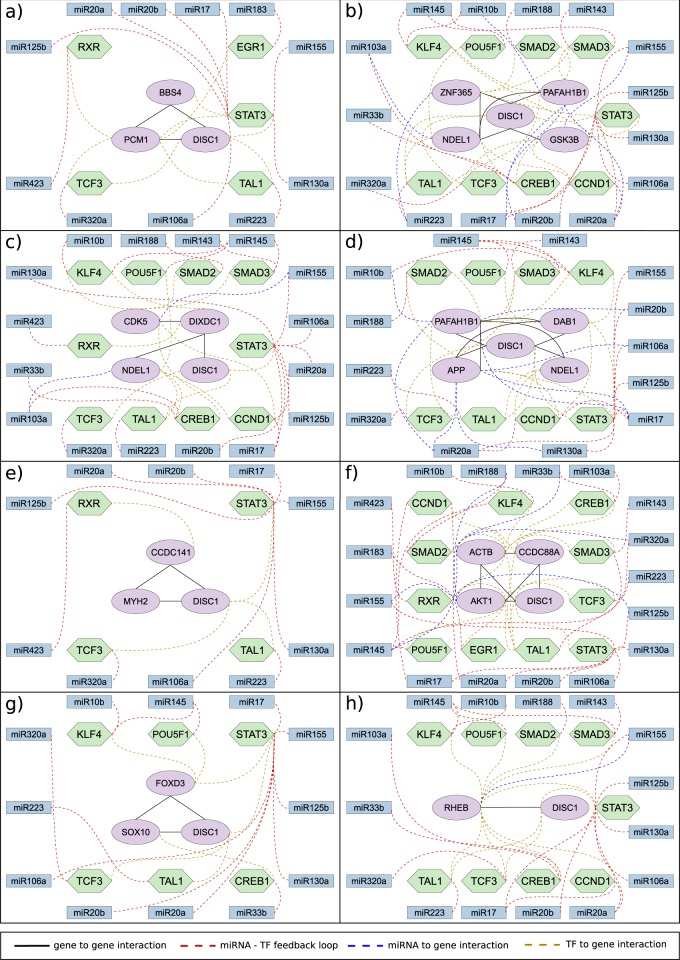
miRNA-TF feedback loops regulating each functional module (FM) of neuronal migration. **a** miRNA-TF feedback loops that regulate FM1, which mediates radial migration of immature neurons; **b** miRNA-TF feedback loops that regulate FM2, which mediates radial migration of basal progenitors; **c** miRNA-TF feedback loops that regulate FM3, which mediates radial migration of newborn neurons; **d** miRNA-TF feedback loops that regulate FM4, which mediates radial migration of neuronal precursor cells; **e** miRNA-TF feedback loops that regulate FM5, which mediates radial migration of apical and basal progenitors; **f** miRNA-TF feedback loops that regulate FM6, which mediates tangential migration of cortical interneurons; **g** miRNA-TF feedback loops that regulate FM7, which mediates cranial neural crest cell migration; and **h** miRNA-TF feedback loops that regulate FM8, which mediates migration of adult hippocampal progenitors

**Fig. 3:**
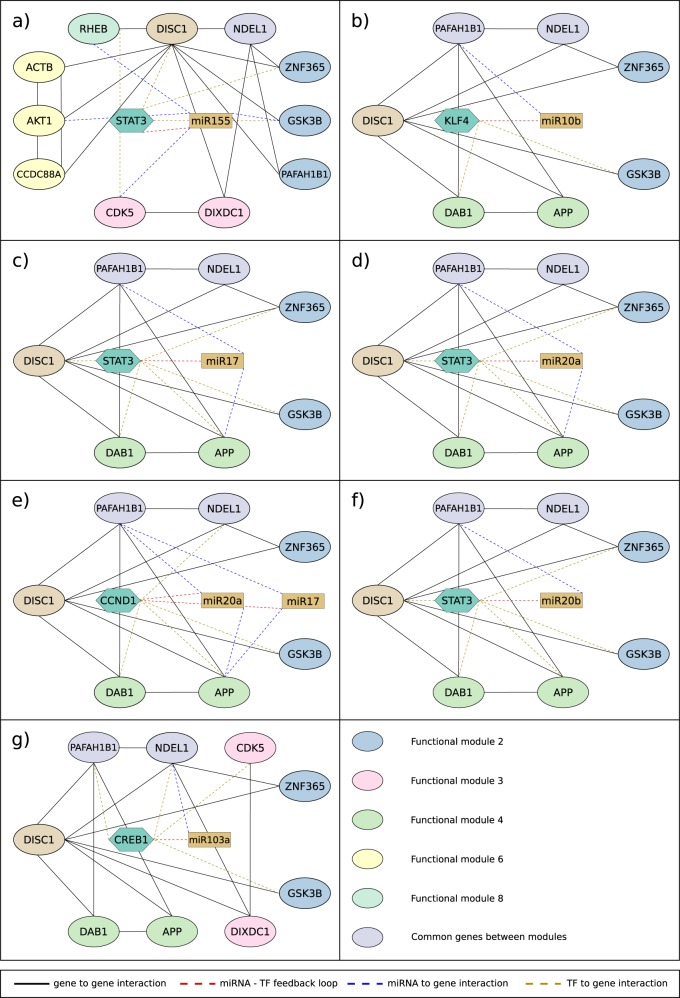
miRNA-TF feedback loops that regulate two or more functional modules of neuronal migration. **a** miR155-STAT3 feedback loop that regulates functional modules 2, 3, 6, and 8; **b** miR10b-KLF4 feedback loop that regulates functional modules 2 and 4; **c** miR17-STAT3 that regulates functional modules 2 and 4; **d** miR20a-STAT3 feedback loop that regulates functional modules 2 and 4; **e** miR17-CCND1 and miR20a-CCND1 feedback loops that regulate functional modules 2 and 4; **f** miR20b-STAT3 feedback loop that regulates functional modules 2 and 4; **g** miR103a-CREB1 feedback loop that regulates functional modules 2–4

We curated 402 experimentally validated miRNAs targeting 17 of the above 19 genes (including *DISC1*) involved in neuronal migration (Supplementary Table S2) (http://zmf.umm.uni-heidelberg.de/apps/zmf/mirwalk2/). No experimentally validated miRNAs were found for *DAB1* and *SOX10* genes. Then, using ChEA datasets, we found 32 experimentally validated TFs regulating 18 of the 19 genes involved in neuronal migration (Supplementary Table S3). No experimentally validated TFs were found that regulated *MYH2* expression. From the 402 experimentally validated miRNAs and 32 experimentally validated TFs (Supplementary Table S3), we identified 21 miRNA-TF feedback loops comprising 17 miRNAs and 11 TFs that regulate gene expression in the 8 FMs (Fig. 2, Supplementary Tables S4−S15 & Supplementary Figs. S1, S2). Eight of the 21 miRNA-TF feedback loops regulated two or more FMs of migration (Supplementary Table S16 and Fig. 3).

### Simulation results

#### Attractor analysis

Starting with 2^19^ initial states (19 proteins mediating migration), we obtained 18 steady-state attractors (i.e. state with one transition leading to the same state) (Table 1). These attractors have differential activity of one or the other of the 19 proteins and represent states wherein neuronal migration is facilitated in all or some of the FMs (*n* = 14) or inhibited in all the eight FMs (*n* = 4). We found a high frequency of occurrence of these 18 attractors in the perturbed networks (3000 perturbations), thereby confirming the robustness of these states (Table 1).

**Table 1:** Attractors (stable-states) reached from 2^19^ initial states by the 19 proteins regulating neuronal migration

Attractor state number	Attractors (stable-states) in asynchronous network (2^19^ states)	Protein order in the attractor sequence (proteins highlighted in bold are proteins that are not activated)	Number of times each attractor occurred in 3000 perturbed networks	Regulation of neuronal migration in functional module (FM)
1	11111111111**0**1**000**111	PCM1 BBS4 CDK5 DIXC1 NDEL1 A4 LIS1 DAB1 CC141 MYH2 ZN365 **GSK3B** DISC1 **SOX10 FOXD3 RHEB** ACTB AKT1 GRDN	1173	Facilitation of migration in all the FMs
2	1111111111**00**1**000**111	PCM1 BBS4 CDK5 DIXC1 NDEL1 A4 LIS1 DAB1 CC141 MYH2 **ZN365 GSK3B** DISC1 **SOX10 FOXD3 RHEB** ACTB AKT1 GRDN	1179	Facilitation of migration in FM1, FM3, FM4, FM5, FM6, FM7, and FM8
3	11111**0**1**0**111**0**1**000**111	PCM1 BBS4 CDK5 DIXC1 NDEL1 **A4** LIS1 **DAB1** CC141 MYH2 ZN365 **GSK3B** DISC1 **SOX10 FOXD3 RHEB** ACTB AKT1 GRDN	1329	Facilitation of migration in FM1, FM2, FM3, FM5, FM6, FM7, and FM8
4	11111**0**1**0**11**00**1**000**111	PCM1 BBS4 CDK5 DIXC1 NDEL1 **A4** LIS1 **DAB1** CC141 MYH2 **ZN365 GSK3B** DISC1 **SOX10 FOXD3 RHEB** ACTB AKT1 GRDN	1345	Facilitation of migration in FM1, FM3, FM5, FM6, FM7, and FM8
5	11**00**1111111**0**1**000**111	PCM1 BBS4 **CDK5 DIXC1** NDEL1 A4 LIS1 DAB1 CC141 MYH2 ZN365 **GSK3B** DISC1 **SOX10 FOXD3 RHEB** ACTB AKT1 GRDN	1281	Facilitation of migration in FM1, FM2, FM4, FM5, FM6, FM7, and FM8
6	11**00**111111**00**1**000**111	PCM1 BBS4 **CDK5 DIXC1** NDEL1 A4 LIS1 DAB1 CC141 MYH2 **ZN365 GSK3B** DISC1 **SOX10 FOXD3 RHEB** ACTB AKT1 GRDN	1271	Facilitation of migration in FM1, FM4, FM5, FM6, FM7, and FM8
7	11**00**1**0**1**0**111**0**1**000**111	PCM1 BBS4 **CDK5 DIXC1** NDEL1 **A4** LIS1 **DAB1** CC141 MYH2 ZN365 **GSK3B** DISC1 **SOX10 FOXD3 RHEB** ACTB AKT1 GRDN	1437	Facilitation of migration in FM1, FM2, FM5, FM6, FM7, and FM8
8	11**00**1**0**1**0**11**00**1**000**111	PCM1 BBS4 **CDK5 DIXC1** NDEL1 **A4** LIS1 **DAB1** CC141 MYH2 **ZN365 GSK3B** DISC1 **SOX10 FOXD3 RHEB** ACTB AKT1 GRDN	1437	Facilitation of migration in FM1, FM5, FM6, FM7, and FM8
9	**00**111111111**0**1**000**111	**PCM1 BBS4** CDK5 DIXC1 NDEL1 A4 LIS1 DAB1 CC141 MYH2 ZN365 **GSK3B** DISC1 **SOX10 FOXD3 RHEB** ACTB AKT1 GRDN	1346	Facilitation of migration in FM2, FM3, FM4, FM5, FM6, FM7, and FM8
10	**00**11111111**00**1**000**111	**PCM1 BBS4** CDK5 DIXC1 NDEL1 A4 LIS1 DAB1 CC141 MYH2 **ZNF365 GSK3B** DISC1 **SOX10 FOXD3 RHEB** ACTB AKT1 GRDN	1350	Facilitation of migration in FM3, FM4, FM5, FM6, FM7, and FM8
11	**00**111**0**1**0**111**0**1**000**111	**PCM1 BBS4** CDK5 DIXC1 NDEL1 **A4** LIS1 **DAB1** CC141 MYH2 ZNF365 **GSK3B** DISC1 **SOX10 FOXD3 RHEB** ACTB AKT1 GRDN	1501	Facilitation of migration in FM2, FM3, FM5, FM6, FM7, and FM8
12	**00**1**0000000**11**0**111**000**	**PCM1 BBS4** CDK5 **DIXC1 NDEL1 A4 LIS1 DAB1 CC141 MYH2** ZN365 GSK3B **DISC1** SOX10 FOXD3 RHEB **ACTB AKT1 GRDN**	1728	**Inhibition of migration in all FMs**
13	**00**1**00000000**1**0**111**000**	**PCM1 BBS4** CDK5 **DIXC1 NDEL1 A4 LIS1 DAB1 CC141 MYH2 ZNF365** GSK3B **DISC1** SOX10 FOXD3 RHEB **ACTB AKT1 GRDN**	1744	**Inhibition of migration in all FMs**
14	**0000**1111111**0**1**000**111	**PCM1 BBS4 CDK5 DIXC1** NDEL1 A4 LIS1 DAB1 CC141 MYH2 ZNF365 **GSK3B** DISC1 **SOX10 FOXD3 RHEB** ACTB AKT1 GRDN	1454	Facilitation of migration in FM2, FM4, FM5, FM6, FM7, and FM8
15	**0000**111111**00**1**000**111	**PCM1 BBS4 CDK5 DIXC1** NDEL1 A4 LIS1 DAB1 CC141 MYH2 **ZN365 GSK3B** DISC1 **SOX10 FOXD3 RHEB** ACTB AKT1 GRDN	1442	Facilitation of migration in FM4, FM5, FM6, FM7, and FM8
16	**0000**1**0**1**0**111**0**1**000**111	**PCM1 BBS4 CDK5 DIXC1** NDEL1 **A4** LIS1 **DAB1** CC141 MYH2 ZN365 **GSK3B** DISC1 **SOX10 FOXD3 RHEB** ACTB AKT1 GRDN	1609	Facilitation of migration in FM2, FM5, FM6, FM7, and FM8
17	**0000000000**11**0**111**000**	**PCM1 BBS4 CDK5 DIXC1 NDEL1 A4 LIS1 DAB1 CC141 MYH2** ZN365 GSK3B **DISC1** SOX10 FOXD3 RHEB **ACTB AKT1 GRDN**	1727	**Inhibition of migration in all FMs**
18	**00000000000**1**0**111**000**	**PCM1 BBS4 CDK5 DIXC1 NDEL1 A4 LIS1 DAB1 CC141 MYH2 ZN365** GSK3B **DISC1** SOX10 FOXD3 RHEB **ACTB AKT1 GRDN**	1743	**Inhibition of migration in all FMs**

DISC1, NDEL1, LIS1, CC141, MYH2, ACTB, AKT1, and GRDN were activated in all 14 states that facilitate migration (attractors 1–11, 14–16) while GSK3B, RHEB, SOX10, and FOXD3 were not activated in all these states. Among the 14 states facilitating migration, proteins in all eight FMs were activated only in the attractor 1 state, while in the other attractor states, one (attractor states 2, 3, 5, and 9), two (attractor states 4, 6, 7, 10, 11, and 14) or three (attractor states 8, 15, and 16) FMs were not activated, affecting neuronal migration in that particular module but facilitating migration in all the other FMs.

GSK3B, FOXD3, SOX10, and RHEB were activated in all four attractor states inhibiting migration in all eight FMs (attractors 12, 13, 17, and 18), while DISC1, PCM1, BBS4, DIXC1, NDEL1, A4, LIS1, DAB1, CC141, MYH2, ACTB, AKT1, and GRDN were not activated in these four states.

From the above, it may be inferred that the most critical proteins within the DISC1 interactome facilitating neuronal migration are DISC1, NDEL1, LIS1, CC141, MYH2, ACTB, AKT1, and GRDN, while GSK3B, FOXD3, SOX10, and RHEB are the most critical inhibitory proteins (Fig. 1 and Table 1). Interestingly, these critical proteins that facilitate and inhibit migration were not simultaneously upregulated in any of the 18 attractor states.

#### Node perturbation analysis for each functional module of migration

We determined the influence of miRNA-TF feedback loops on neuronal migration by overexpression (OE) and knockout (KO) of each node (gene/miRNA/TF) in each FM. Using experimental evidence, perturbations that upregulate (100%) or downregulate (0%) neuronal migrational processes were identified for each FM, as shown in Supplementary Figs. S3–S34.

**Fig. 4:**
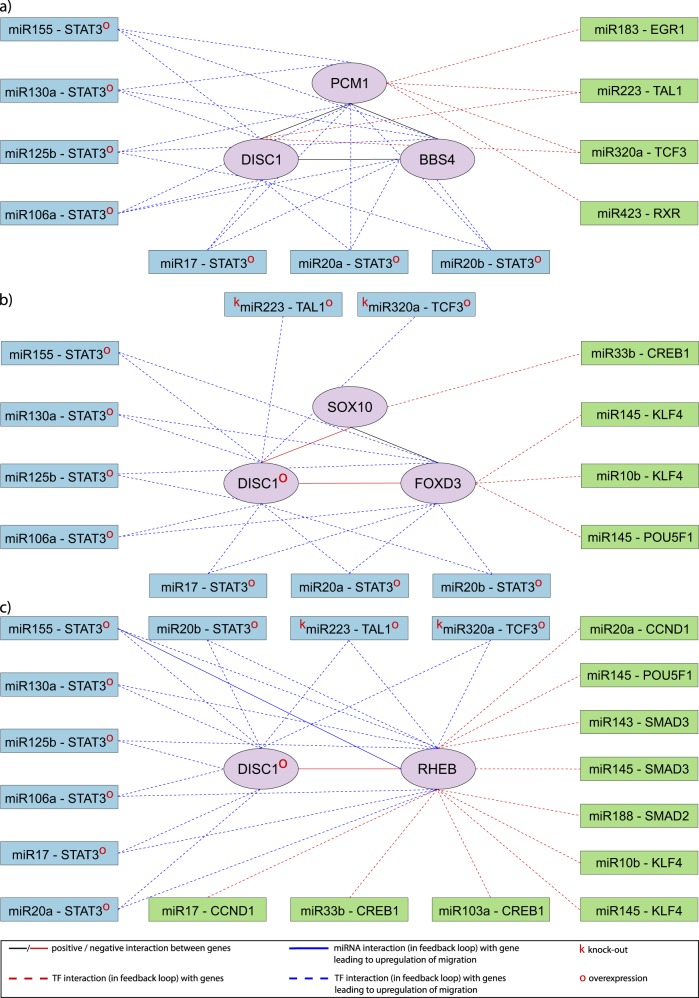
Perturbed nodes in functional module 1 (FM1), functional module 7 (FM7), and functional module 8 (FM8) that upregulate migration. Depending upon the type of simulation, perturbation of nodes (overexpression/knockout) in **a** FM1, **b** FM7, and **c** FM8 resulted in upregulation of migration (see text for details). Green-colored nodes represent nodes, which when perturbed, did not upregulate migration

Given that TF can activate or repress miRNA and target gene expression, we performed four different simulations (Simulations 1–4), as detailed in the Methods section. Results of simulations 1 and 3 were similar in each FM as TF activates gene expression in both. Similarly results of simulations 2 and 4 were similar as TF represses gene expression in both. The FMs in which perturbation of nodes resulted in upregulation of migration are depicted in Fig. 4. In FM1, during simulations 1 and 3, OE of STAT3 upregulated migration (100%) (Supplementary Figs. S3, S5), while in simulations 2 and 4, OE of STAT3 downregulated migration (0%) (Supplementary Figs. S4, S6). This shows major influence of TF STAT3 in the regulation of FM1 through regulation of the expression of *PCM1*, *BBS4*, and *DISC1*. In FMs 2–6, OE or KO of any node did not result in upregulation of migration (Supplementary Figs. S7–S10 (FM2); S11–S14 (FM3); S15–S18 (FM4); S19–S22 (FM5); S23–S26 (FM6)); this indicates that miRNA-TF feedback loops may be responsible for downregulating the neuronal migration process in these FMs. In FMs 7 and 8, *DISC1* OE resulted in upregulation of migration (100%) in all four simulations (Supplementary Figs. S27–S30 (FM7) and Supplementary Figs. S31–S34 (FM8)). This facilitatory effect of *DISC1* OE on migration is mediated through repression of the inhibitory *SOX10* and *FOXD3* in FM7 and *RHEB* in FM8. This shows that *DISC1* OE surmounted the regulatory control by miRNA-TF feedback loop, thereby upregulating migration in FMs 7 and 8.

##### Perturbation of miRNAs

miR223 (regulating TAL1 expression) and miR320a (regulating TCF3 expression) and the TFs: TAL1, TCF3 and STAT3 in FMs 7 and 8 showed pivotal role of these miRNAs and TFs in either upregulation or downregulation of migration by regulating *DISC1* expression. In simulations 1 and 3, KO of miR223 or miR320a and OE of STAT3 or TCF3 or TAL1 showed upregulation of migration (100%), as *DISC1* was expressed (Supplementary Figs. S27, S29 (FM7) and S31, S33 (FM8)). In simulations 2 and 4, KO of miR320a or miR223 or OE of TCF3 or TAL1 or STAT3 resulted in downregulation (0%) of migration in FMs 7 and 8, as *DISC1* was not expressed (Supplementary Figs. S28, S30 (FM7) and S32, S34 (FM8)). However, perturbation of miRNAs regulating *STAT3* expression (miR155 or miR106a or miR20a or miR17 or miR103a or miR125b or miR20b) showed neither upregulation nor downregulation of migration compared to perturbation of miR320a (regulating *TCF3*) or miR223 (regulating *TAL1* expression), as STAT3 was expressed only when all the miRNAs regulating its expression in feedback loop were repressed (Supplementary Figs. S27–S30 (FM7) and Supplementary Figs. S31–S34 (FM8)).

Analysis of the perturbation results of miRNA-TF feedback loops regulating two or more FMs of migration revealed that KO and OE of miRNA or TF in each of these feedback loops (except miR155-STAT3 feedback loop) at time steps *t* = 0 and *t* = 140 showed similar effects on neuronal migration in the FMs, i.e. migration was not upregulated (100%) but was either downregulated (0%) or regulated between 0 and 100% (Supplementary Figs. S35−S41). This similar pattern of regulation of neuronal migration observed in the FMs reflects similar mechanisms by which different modes of neuronal migration coexisting in the developing central nervous system are regulated. However, in the miR155-STAT3 feedback loop regulating FMs 2, 3, 6, and 8, OE of STAT3 (simulations 1 and 3) resulted in upregulation (100%) of migration only in FM8 (migration of adult hippocampal progenitors), as STAT3 upregulates *DISC1* expression that represses *RHEB* expression and mediates migration (Supplementary Fig. S42). Though STAT3 upregulates *DISC1* expression in FMs 2, 3, and 6, expression of other genes is also crucial for mediation of neuronal migration in these modules. This finding indicates a critical role of STAT3 in regulating migration of adult hippocampal progenitors (repressing *RHEB* expression by activating *DISC1* expression).

Thus, from the results of node perturbation analysis, we infer that the miRNA-TAL1, miRNA-TCF3, and miRNA-STAT3 feedback loops play a major role in upregulating (100%) neuronal migration in FMs 1 (Supplementary Figs. S3–S6), 7 (Supplementary Figs. S27–S30) and 8 (Supplementary Figs. S31–S34). Perturbation of these feedback loops in other FMs (2, 3, 4, 5, and 6) showed the role played by these feedback loops in negative regulation of migration (Supplementary Figs. S7–S10 (FM2); S11–S14 (FM3); S15–S18 (FM4); S19–S22 (FM5); S23–S27 (FM6)).

**Fig. 5:**
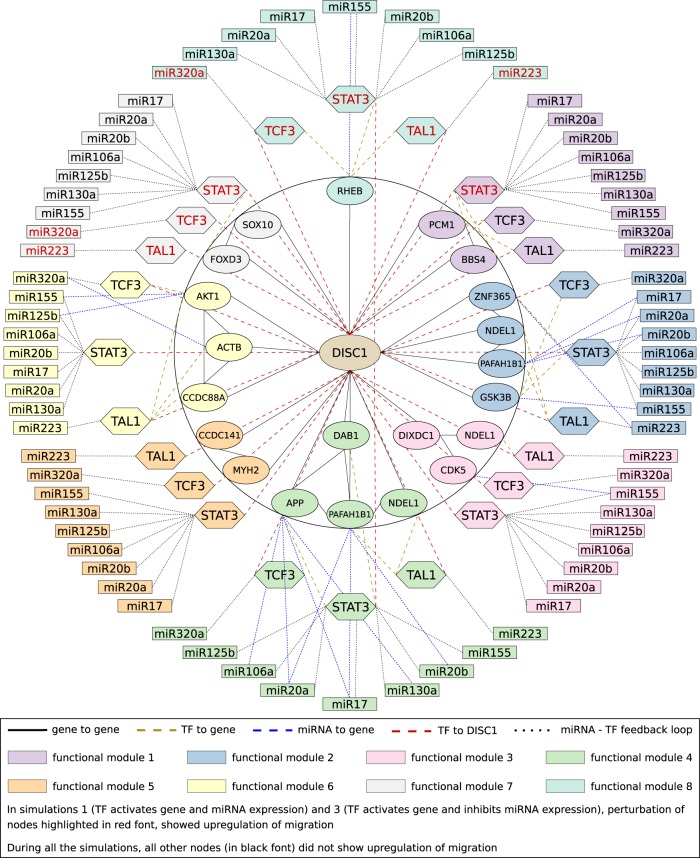
Comprehensive network model of regulation of functional modules of migration by miRNA-STAT3, miRNA-TAL1 and miRNA-TCF3 feedback loops. The figure illustrates the various interactions between genes (central circle), the three most critical TFs, viz., STAT3, TAL1, and TCF3 (middle zone) as well as all possible miRNAs (outer zone) that form feedback loops with these TFs. These three TFs in feedback loops with miRNA are considered critical since they directly upregulate *DISC1* expression. The miRNAs along with the above three critical TFs that are involved in upregulation of migration in functional modules 1, 7, and 8 are shown in red fonts. In functional module 1, overexpression of STAT3 upregulates migration in simulations 1 and 3, while it downregulates migration in simulations 2 and 4. In functional modules 2–6, overexpression of each gene or miRNA or TF did not result in upregulation of migration, indicating these miRNA-TF feedback loops exhibit an inhibitory control of the genes in these functional modules. In functional module 7, *DISC1* overexpression upregulates migration through repression of the inhibitory *SOX10* and *FOXD3*, while in functional module 8, *DISC1* overexpression upregulates migration through repression of the inhibitory *RHEB*. In both functional modules 7 and 8, knockout of miR320a (that regulates TCF3 expression) and miR223 (that regulates TAL1 expression), or overexpression of TCF3 or TAL1 or STAT3 resulted in upregulation of migration in simulations 1 and 3 and downregulation of migration in simulations 2 and 4. Unlike TAL1 and TCF3, STAT3 is expressed only when all the miRNAs regulating its expression in feedback loops are repressed, and therefore upregulation or downregulation of migration does not occur when any of these miRNAs that regulate STAT3 expression are individually upregulated or downregulated. Finally, apart from their direct regulation of *DISC1* expression, TAL1, TCF3, and STAT3 directly regulate expression of *PCM1 and BBS4* genes in functional module 1 while STAT3 regulates expression of *FOXD3* in functional module 7. Furthermore, these TFs along with the respective miRNAs in feedback loops directly regulate expression of *PAFAH1B1, ZNF365, GSK3B, and NDEL1* genes in functional module 2, *CDK5 and NDEL1* genes in functional module 3, *APP, NDEL1, DAB1, and PAFAH1B1* genes in functional module 4, *AKT1, ACTB, and CCDC88A* genes in functional module 6 and *RHEB* gene in functional module 8

Integrating these regulatory interactions, we constructed a comprehensive network model showing regulation of gene expression in each FM by these feedback loops (Fig. 5). Apart from regulating *DISC1* expression, TFs in these FBLs directly regulated gene expression as seen in FM1 or, both TF and miRNA directly regulated expression of genes as seen in FMs 2–4, 6, and 8. But in FM5, these three TFs only regulated *DISC1* expression.

## Discussion

Using an in-silico Boolean approach, we were able to build a comprehensive network model depicting the regulation of proteins within the FMs of the DISC1 interactome by miRNA-TF feedback loops (Fig. 5). This model posits that depending upon the direction of regulation by TFs, perturbation of either TFs or miRNA in feedback loop affects gene expression in various FMs, which in turn affects PPIs in the DISC1 interactome mediating neuronal migration. Such an approach enabled us to identify the most critical proteins within the DISC1 interactome mediating neuronal migration, viz., DISC1, NDEL1, LIS1, CC141, MYH2, ACTB, AKT1, and GRDN (facilitatory); GSK3B, SOX10, FOXD3, and RHEB (inhibitory); as well as the most critical miRNA-TF feedback loops regulating the DISC1 interactome viz., miR223-TAL1, miR320a-TCF3, and miR155-STAT3.

The results of this in-silico study are robustly supported by published experimental evidence. The critical role of *DISC1* in neuronal migration and its association with various neuronal migration and neuropsychiatric disorders is well established.^3^ DISC1 has been shown to interact with NDEL1 and LIS1 that are integral components of dynein motor protein complex, which regulates radial migration. This complex couples nucleus and centrosome, an essential step in radial migration of cortical excitatory neurons.^20^ DISC1/GSK3B interaction determines the transition of neural progenitor self-renewal to neuronal migration in the developing brain.^21^ In FM2 (Fig. 1), when DISC1 inhibits GSK3B at the distal end, LIS1 is retrogradely transported along the microtubules, aiding in migration.^22^ MYH2, a motor protein, regulates centrosomal positioning by associating with CC141 and DISC1.^23^ MYH2 and dynein act in concert by interacting with specific cytoskeletal elements.^24^ Further, *NDEL1* and *PAFAH1B1* (LIS1) have been strongly linked to lissencephaly, microcephaly,^25^ and Miller−Dieker syndrome^26^ while *MYH2* mutations have been reported as a rare cause of distal arthrogryposis type 5.^27^

DISC1 has complex interactions with AKT1 and GRDN in a network that also involves GSK3B, whereby incorrect neuronal localization resulting from over-extended migration into the outer granule cell layer and the molecular cell layer due to enhanced AKT1 signaling is prevented.^28,29^ ACTB, a structural protein involved in cytoskeletal organization,^30^ interacts with DISC1, AKT1, and GRDN regulating tangential migration of cortical interneurons^31^ (Fig. 1). Furthermore, AKT1 and GRDN are major regulatory proteins of mammalian target of rapamycin (mTOR) signaling, coordinating brain morphogenesis.^32^

DISC1 regulates cranial neural crest (CNC) cell migration and differentiation through the transcriptional repression of *FOXD3* and *SOX10* involved in glial differentiation in CNC cells.^33^ RHEB, inhibited by DISC1 in FM8, is an activator of mTOR signaling. Deletion of *Rheb* was found to rescue neurons from migratory defects, as mTOR signaling was not activated. Similarly, when mTOR signaling was inhibited in newborn neurons, the neurons were rescued from DISC1 deficiency-induced migratory defects.^34^

The genes encoding the above proteins that play a critical role in migration through their interactions with DISC1, viz., *NDEL1, PAFAH1B1* (LIS1)*, CCDC141* (CC141), *AKT1, ACTB*, *CCDC88A* (GRDN), *GSK3B*, *SOX10*, and *RHEB* have been shown to be associated with schizophrenia^35^ (see Supplementary References 52–56,60,61 for original articles), autism^36^ (see Supplementary References 57–62 for original articles), Alzheimer’s disease^37^ and bipolar disorder.^38^

The most important finding to emerge from this in-silico study is the major role played by three miRNA-TF feedback loops viz., miR223-TAL1, miR320a-TCF3, and miR155-STAT3, in regulating the DISC1 interactome mediating neuronal migration. To the best of our knowledge, no experimental studies have so far linked these TFs with the transcriptional control of DISC1 interactome. From our simulation results, we have found evidence for the role of these TFs as primary factors (through feedback loops with miRNAs) in regulation of the DISC1 interactome involved in migration. This new insight generated from our study highlights the role of such in-silico methods for discovery of key TFs that could constitute important targets for further experimental validation. Identification of such TFs in experimental studies is challenging owing to the large set of regulatory factors (such as genes, miRNAs, and TFs) involved in a given function, often without converging evidence. As an example, in the current study we curated 18 DISC1-interacting proteins that were shown to be involved in migration. This would mean an almost impossible task of testing out an immense number of interactions between these 19 proteins and their regulatory miRNAs and TFs in a systematic way to find critical regulatory factors mediating neuronal migration. We were able to identify these potentially important factors by the use of Boolean modeling without having to explore each interaction independently.

Though the evidence generated by our study for the role of STAT3, TAL1, and TCF3 in the regulation of the DISC1 interactome mediating neuronal migration is a new discovery, there is evidence from existing literature that supports the role of these TFs in neuronal migration as well as in pathophysiology of various migrational disorders in neuropsychiatry. STAT3 pathway has been shown to promote neurite outgrowth and neuronal migration by inhibiting apoptosis.^39^ Expression studies have shown upregulation of miRNAs such as miR155, miR17, miR20a targeting *STAT3* in superior temporal gyrus^40^ and miR20b in dorsolateral prefrontal cortex (DLPFC) post-mortem samples from patients with schizophrenia.^41^ STAT3 was found to be one of TFs enriched in autism, X-linked intellectual disorder, ADHD and schizophrenia (AXAS) PPI network. Moreover, the *SLC25A12* gene associated with autism has STAT3 binding site.^42^ STAT3 regulates migration by inhibiting the activity of stathmin, a microtubule destabilizing protein.^43^ *TAL1* is one of the TFs involved in GABA-ergic neuronal development, a process that includes patterning of neuroepithelium, specification and generation of post-mitotic neural precursors, differentiation and migration.^44^ *TAL1* has been shown to be differentially regulated in people with autism compared to healthy subjects.^45^ In another study, miR223 regulating *TAL1* expression was found to be upregulated in post-mortem DLPFC samples in patients with schizophrenia.^40^ TCF3 has been found to regulate vertebrate head formation and patterning.^46^ Its role in migration was demonstrated in an earlier report which showed recapitulation of neural crest migration defects induced by 6-bromoindirubin-3-oxime, through activation of LEF1/TCF3 signaling.^47^ Further, miR320a regulating *TCF3* expression was found to be downregulated in peripheral blood of patients with schizophrenia.^48^

This in-silico study provides new insights into the molecular mechanisms that underlie various FMs of neuronal migration involving DISC1. We have explained our simulation results based on existing experimental evidence. This model provides a computationally derived framework for further experimental validation studies that could enhance our understanding of processes involved in neuronal migration. The Boolean network model of the DISC1 interactome from the present study can be expanded to integrate all known transcriptomic regulatory elements regulating migration. Using pathway enrichment analysis tools,^49,50^ we can determine the multiple pathways regulated by the key TFs, viz., TAL1, TCF3, and STAT3 during neuronal migration. Using brain expression datasets from Brain Span^51^ and Human Brain Transcriptome,^52^ we can then determine the genes that are coexpressed along with these regulatory TFs during neuronal migration. Proteins or regulatory factors in a transcriptional network that have a high degree of connectivity are referred to as “hub” proteins/regulatory factors. These hub proteins/regulatory factors are known to be densely interconnected to each other forming “rich clubs” in the interactome.^53,54^ Elucidation of the functions of these rich clubs in the transcriptional network of the DISC1 interactome can enhance our understanding of regulatory factors involved in neuronal migration. For example, rich clubs in the network of dysregulated proteins in cerebral ischemia were shown to regulate coagulation and complement cascade, indicating the role of these proteins in the causation of this condition.^55^

Using molecular experimental methods, it is extremely challenging to identify all the regulatory factors involved in various stages of neurodevelopment. A network model similar to the transcriptional network of the DISC1 interactome can be developed, integrating all known transcriptomic elements regulating all the various stages of neurodevelopment. With the vast amount of molecular data available from projects such as ENCODE (Encyclopedia of DNA Elements),^56,57^ modENCODE (Model Organism ENCyclopedia Of DNA Elements)^58,59^ and REMC (Roadmap Epigenomics),^60^ we can additionally incorporate epigenetic regulation of specific molecular/cellular processes related to neurodevelopment into the model. Building such an in-silico mechanistic model comprising of several functional modules regulating molecular and cellular processes could provide a holistic framework to understand how these processes coordinate and regulate various stages of neurodevelopment. This framework also captures the state of the critical elements that would stabilize or disrupt the neurodevelopmental network, providing clues about the transcriptional and epigenetic regulation machinery in neurodevelopmental disorders. These mechanistic models can then be tested using carefully designed molecular biology experiments.

## Methods

### Generation of the composite DISC1 interactome

Initially, we explored proteins interacting with DISC1 using GeneMania^61^ (https://genemania.org/), NCBI Gene (https://www.ncbi.nlm.nih.gov/gene) databases and Agile Protein Interaction Data Analyzer (APID) (http://apid.dep.usal.es/)^62^ till 31 August 2017.

Although databases provide a convenient means of analyzing interactions between proteins, there exists certain amount of bias and omissions of annotation with these databases, which could lead to missing out of important interaction data in a given database.^63,64^ Therefore, to identify proteins that have not been curated in the databases, we also performed an extensive search of the biomedical literature resources viz., NCBI PubMed and Google Scholar, using the following keywords: DISC1 interactions; DISC1-interacting genes; DISC1 neurogenesis; DISC1 neurodevelopment; DISC1 migration; DISC1 genetic interactions; DISC1 protein interactions; DISC1 signaling; DISC1 regulatory role; DISC1 biological functions; DISC1 molecular functions; DISC1 cellular functions; DISC1 functions. The retrieved interactions were reported using model organisms such as *Rattus norvegicus*, *Mus musculus*, *Danio rerio, Caenorhabditis elegans* as well as PC12, HEK293, COS-7, and SH-SY5Y cell lines. From the output of the search in the databases (GeneMania, NCBI Gene and APID) as well as from the Biomedical literature resources (NCBI PubMed and Google Scholar), we compiled the final list of proteins that interact with DISC1. We excluded those DISC1 PPIs retrieved from the databases which were reported based on “co-citations” in the literature (but, lacking experimental evidence), interactions reported with uncharacterized proteins as well as epistatic interactions reported by association studies.

We then examined the experimental evidence for the role of above-retrieved DISC1 PPIs in neurodevelopment. Specifically, we curated the experimental evidence from PubMed and Google Scholar search engines (see keywords above) of how these DISC1 PPIs regulate cellular/molecular functions that are relevant for neuronal migration and included only those in our subsequent steps. We meticulously verified the regulation of DISC1 PPIs in neuronal migration by curating details such as type of interaction (direct/indirect), direction of interaction (directed/bidirectional), regulation of interaction on migration (facilitation/inhibition), experiments/methods (high throughput/low throughput) and the model organisms/cell lines used to study the interaction. We defined specific DISC1 PPIs that mediate specific cellular/molecular functions relevant for neuronal migration as “functional modules”.^65,66^ Finally, a composite DISC1 interactome that integrates all the above functional modules involved in neuronal migration was generated (Fig. 1).

### Identification of miRNA-TF feedback loops that regulate the DISC1 interactome mediating neuronal migration

We used miRWALK2.0 database^67^ to retrieve miRNAs that target genes involved in neuronal migration; and using Enrichr server,^68^ ChEA 2016 datasets were used for identifying the TFs that regulate expression of genes involved in neuronal migration. In ChEA datasets, statistical enrichment is computed by implementing Fishers exact test.^69^ Only those TFs enriched at a statistically significant *p* value ≤ 0.05 were considered for subsequent analysis as shown in Supplementary Table S3.

To identify miRNAs (from miRWALK2.0 database) and TFs (from ChEA 2016 datasets) that mutually regulate each other in feedback loops, we derived the list of miRNAs regulating expression of TFs from miRTarBase^70^ and the list of TFs regulating expression of miRNAs from TransmiR v1.0 ^71^ and ChIPBase v2.0 databases^72^ (an outline of the steps followed is shown in Supplementary Fig. S1).

### Boolean modeling

In a Boolean network, genes and their interactions are represented as a directed graph *G*(*V,E*), where *V* represents the genes and *E* represents the interaction between these genes. Each gene can have a value of either 1 (True) or 0 (False); thus, a Boolean network with *n* genes will have 2^*n*^ possible states. Each gene has a set of states in the network $X = \left\{ {X_i|i = 1,2,3, \ldots ,n} \right\}$ and a set of Boolean functions, $f = \left\{ {f_i|i = 1,2,3, \ldots ,k} \right\}$. For example, the state of gene *V*_*i*_ at time *t* is denoted as *X*_*i*_(*t*) and at *t* + 1 as $X_i\left( {t + 1} \right) = f_i\left( {X_{i1},X_{i2},X_{i3}, \ldots ,X_{ik}} \right)$.

Based on experimental evidence curated from literature and databases, each of the 19 proteins mediating neuronal migration was given a transition function (update rule), expressed using logical operators (https://github.com/mbialnimhans/DISC1_interactome). Similarly, for node perturbation analysis (see below), depending upon the type of simulation, a transition function for each gene, miRNA, and TF for each FM of migration was specified (https://github.com/mbialnimhans/DISC1_interactome).

### Attractor identification

To determine the attractor/stable state reached from these 19 proteins (involved in neuronal migration), we applied an asynchronous mode of transition^73,74^ where the transition function of only one protein is chosen at random and the corresponding protein is updated at the next transition step. Since there were 19 proteins involved in neuronal migration, we considered all 2^19^ states as initial states; the state transition from these states to reach the attractor state was determined by random walk phase method, i.e., “a high number of random state transitions are performed to enter an attractor with high probability”.^73^ To test the robustness of the 18 attractors thus obtained, we created 3000 perturbed networks by shuffle method and calculated the number of times each of these attractors occurred in these networks using random walk phase method. In shuffle method, the output of transition function was randomly permuted under the function “perturbNetwork” in R BoolNet 2.1.3 package.

### Node perturbation analysis

We performed the node perturbation analysis, using Python BooleanNet 1.2.7 module.^75^ In node perturbation analysis, we set the nodes (TF/miRNA/gene) to true (overexpression: OE) or false (knockout: KO) and analyzed the effect of this perturbed node on each FM of migration. We repeated asynchronous simulations 1000 times at each time step (from *t* = 0 to *t* = 150) and simulations were performed for the same initial condition with random update orders. For calculating the activation frequency of neuronal migration, we divided the number of simulations in which the node (neuronal migration) is ON by the number of simulations.^75^ We plotted the results of activation frequency (percent) at which migration is in TRUE state using a smoothing window of ten time steps, i.e. 140 time steps. We performed four different simulations for each FM: when TF activates gene and miRNA expression (Simulation 1); when TF represses both miRNA and gene expression (Simulation 2); when TF represses miRNA and activates gene expression (Simulation 3); and when TF represses gene and activates miRNA expression (Simulation 4).

For analyzing the trend of regulation of migration by miRNA-TF feedback loops regulating two or more FMs of migration, we examined the frequency of activation of migration in each FM at *t* = 0 and *t* = 140.

### Reporting summary

Further information on research design is available in the Nature Research Reporting Summary linked to this article.

## Supplementary information

Supplementary information S1

Supplementary Table S2

Supplementary Table S3

Supplementary references

reporting summary

## Data Availability

All data needed to evaluate the conclusions in the paper are present in the paper and/or the Supplementary Materials. Additional data related to this paper may be requested from the authors.

## Supplementary information

**Supplementary information** accompanies the paper on the *npj Systems Biology and Applications* website (10.1038/s41540-019-0094-3).

## Code availability

Attractor analysis was performed using R (version 3.4.1) BoolNet 2.1.3 package. Node perturbation analysis was performed using Python (version 2.7.11) BooleanNet 1.2.7 module. The scripts are available at https://github.com/mbialnimhans/DISC1_interactome

## References

[1] Tabata H, Nagata KI (2016). Decoding the molecular mechanisms of neuronal migration using in utero electroporation. Med. Mol. Morphol..

[2] Ishizuka K (2011). DISC1-dependent switch from progenitor proliferation to migration in the developing cortex. Nature.

[3] Brandon NJ, Sawa A (2011). Linking neurodevelopmental and synaptic theories of mental illness through DISC1. Nat. Rev. Neurosci..

[4] Niwa M (2016). DISC1 a key molecular lead in psychiatry and neurodevelopment: No-More Disrupted-in-Schizophrenia 1. Mol. Psychiatry.

[5] Zhang HM (2015). Transcription factor and microRNA co-regulatory loops: important regulatory motifs in biological processes and diseases. Brief. Bioinforma..

[6] Martinez NJ (2008). A *C. elegans* genome-scale microRNA network contains composite feedback motifs with high flux capacity. Genes Dev..

[7] Afshar AS, Xu J, Goutsias J (2014). Integrative identification of deregulated miRNA/TF-mediated gene regulatory loops and networks in prostate cancer. PLoS ONE.

[8] Alvis B, Schlitt T (2003). Reverse engineering of gene regulatory networks: a finite state linear model. Genome Biol..

[9] Matsuno H, Doi A, Nagasaki M, Miyano S (2000). Hybrid Petri net representation of gene regulatory network. Pac. Symp. Biocomput..

[10] Paulsson J (2005). Models of stochastic gene expression. Phys. Life Rev..

[11] Polynikis A, Hogan SJ, di Bernardo M (2009). Comparing different ODE modelling approaches for gene regulatory networks. J. Theor. Biol..

[12] Jafari M, Ansari-Pour N, Azimzadeh S, Mirzaie M (2017). A logic-based dynamic modeling approach to explicate the evolution of the central dogma of molecular biology. PLoS ONE.

[13] Lu J (2015). Network modelling reveals the mechanism underlying colitis-associated colon cancer and identifies novel combinatorial anti-cancer targets. Sci. Rep..

[14] Schlatter R (2009). ON/OFF and beyond—a boolean model of apoptosis. PLoS Comput. Biol..

[15] Zhang R (2008). Network model of survival signaling in large granular lymphocyte leukemia. Proc. Natl Acad. Sci. USA.

[16] Choi M, Shi J, Jung SH, Chen X, Cho KH (2012). Attractor landscape analysis reveals feedback loops in the p53 network that control the cellular response to DNA damage. Sci. Signal.

[17] Kauffman SA (1969). Metabolic stability and epigenesis in randomly constructed genetic nets. J. Theor. Biol..

[18] Thomas R (1973). Boolean formalization of genetic control circuits. J. Theor. Biol..

[19] Albert R, Thakar J (2014). Boolean modeling: a logic-based dynamic approach for understanding signaling and regulatory networks and for making useful predictions: Boolean modeling. Wiley Interdiscip. Rev.: Syst. Biol. Med..

[20] Shu T (2004). Ndel1 operates in a common pathway with LIS1 and cytoplasmic dynein to regulate cortical neuronal positioning. Neuron.

[21] Kim WY, Snider WD (2011). Functions of GSK-3 signaling in development of the nervous system. Front Mol. Neurosci..

[22] Okamoto M (2015). DBZ regulates cortical cell positioning and neurite development by sustaining the anterograde transport of Lis1 and DISC1 through control of Ndel1 dual-phosphorylation. J. Neurosci..

[23] Fukuda T, Sugita S, Inatome R, Yanagi S (2010). CAMDI, a novel disrupted in schizophrenia 1 (DISC1)-binding protein, is required for radial migration. J. Biol. Chem..

[24] Vallee RB, Seale GE, Tsai JW (2009). Emerging roles for myosin II and cytoplasmic dynein in migrating neurons and growth cones. Trends Cell Biol..

[25] Sasaki S (2005). Complete loss of Ndel1 results in neuronal migration defects and early embryonic lethality. Mol. Cell. Biol..

[26] Liu JS (2011). Molecular genetics of neuronal migration disorders. Curr. Neurol. Neurosci. Rep..

[27] Bamshad M, Van Heest AE, Pleasure D (2009). Arthrogryposis: a review and update. J. Bone Jt. Surg. Am..

[28] Kim JY (2009). DISC1 regulates new neuron development in the adult brain via modulation of AKT-mTOR signaling through KIAA1212. Neuron.

[29] Enomoto A (2009). Roles of disrupted-in-schizophrenia 1-interacting protein girdin in postnatal development of the dentate gyrus. Neuron.

[30] Matthews PR, Eastwood SL, Harrison PJ (2012). Reduced myelin basic protein and actin-related gene expression in visual cortex in schizophrenia. PLoS ONE.

[31] Steinecke A, Gampe C, Nitzsche F, Bolz J (2014). DISC1 knockdown impairs the tangential migration of cortical interneurons by affecting the actin cytoskeleton. Front. Cell. Neurosci..

[32] Ka M, Condorelli G, Woodgett JR, Kim WY (2014). mTOR regulates brain morphogenesis by mediating GSK3 signaling. Development.

[33] Drerup CM, Wiora HM, Topczewski J, Morris JA (2009). Disc1 regulates foxd3 and sox10 expression, affecting neural crest migration and differentiation. Development.

[34] Kang E (2015). Rheb1 mediates DISC1-dependent regulation of new neuron development in the adult hippocampus. Neurogenesis (Austin).

[35] Deutsch SI, Burket JA, Katz E (2010). Does subtle disturbance of neuronal migration contribute to schizophrenia and other neurodevelopmental disorders? Potential genetic mechanisms with possible treatment implications. Eur. Neuropsychopharmacol..

[36] Reiner O, Karzbrun E, Kshirsagar A, Kaibuchi K (2016). Regulation of neuronal migration, an emerging topic in autism spectrum disorders. J. Neurochem..

[37] Koran MEI, Hohman TJ, Meda SA, Thornton-Wells TA (2014). Genetic interactions within inositol-related pathways are associated with longitudinal changes in ventricle size. J. Alzheimers Dis..

[38] Karege F (2010). Association of AKT1 gene variants and protein expression in both schizophrenia and bipolar disorder. Genes Brain Behav..

[39] Liu H, Liu G, Bi Y (2014). CNTF regulates neurite outgrowth and neuronal migration through JAK2/STAT3 and PI3K/Akt signaling pathways of DRG explants with gp120-induced neurotoxicity in vitro. Neurosci. Lett..

[40] Beveridge NJ, Gardiner E, Carroll AP, Tooney PA, Cairns MJ (2010). Schizophrenia is associated with an increase in cortical microRNA biogenesis. Mol. Psychiatry.

[41] Perkins DO (2007). microRNA expression in the prefrontal cortex of individuals with schizophrenia and schizoaffective disorder. Genome Biol..

[42] Cristino AS (2014). Neurodevelopmental and neuropsychiatric disorders represent an interconnected molecular system. Mol. Psychiatry.

[43] Tuma RS (2006). Stat3 stabilizes microtubules. J. Cell Biol..

[44] Achim K (2013). The role of Tal2 and Tal1 in the differentiation of midbrain GABAergic neuron precursors. Biol. Open.

[45] Wall DP (2009). Comparative analysis of neurological disorders focuses genome-wide search for autism genes. Genomics.

[46] Kim CH (2000). Repressor activity of Headless/Tcf3 is essential for vertebrate head formation. Nature.

[47] Maj E (2016). Controlled levels of canonical Wnt signaling are required for neural crest migration. Dev. Biol..

[48] Vachev TI, Todorov Popov N, Krasteva Stoyanova V, Yordanov Ivanov H, Savov Minchev D (2016). Down regulation of MIR-320 gene family members in the peripheral blood of schizophrenia patients. Int. J. Curr. Microbiol. Appl. Sci..

[49] Huang DW, Sherman BT, Lempicki RA (2009). Systematic and integrative analysis of large gene lists using DAVID bioinformatics resources. Nat. Protoc..

[50] Kamburov A (2011). ConsensusPathDB: toward a more complete picture of cell biology. Nucleic Acids Res..

[51] Sunkin SM (2013). Allen Brain Atlas: an integrated spatio-temporal portal for exploring the central nervous system. Nucleic Acids Res..

[52] Kang HJ (2011). Spatio-temporal transcriptome of the human brain. Nature.

[53] Ma A, Mondragón RJ (2015). Rich-cores in networks. PLoS ONE.

[54] Csermely P (2012). Disordered proteins and network disorder in network descriptions of protein structure, dynamics and function: hypotheses and a comprehensive review. Curr. Protein Pept. Sci..

[55] Alawieh A, Sabra Z, Sabra M, Tomlinson S, Zaraket FA (2015). A rich-club organization in brain ischemia protein interaction network. Sci. Rep..

[56] The ENCODE Project Consortium. (2012). An integrated encyclopedia of DNA elements in the human genome. Nature.

[57] Davis CA (2018). The Encyclopedia of DNA elements (ENCODE): data portal update. Nucleic Acids Res..

[58] Gerstein MB (2010). Integrative analysis of the *Caenorhabditis elegans* enome by the modENCODE Project. Science.

[59] Cheng C (2011). Construction and analysis of an integrated regulatory network derived from high-throughput sequencing data. PLoS Comput. Biol..

[60] Roadmap Epigenomics Consortium (2015). Integrative analysis of 111 reference human epigenomes. Nature.

[61] Mostafavi S, Ray D, Warde-Farley D, Grouios C, Morris Q (2008). GeneMANIA: a real-time multiple association network integration algorithm for predicting gene function. Genome Biol..

[62] Alonso-López D (2016). APID interactomes: providing proteome-based interactomes with controlled quality for multiple species and derived networks. Nucleic Acids Res..

[63] Mrowka R, Patzak A, Herzel H (2001). Is there a bias in proteome research?. Genome Res..

[64] Pattin KA, Moore JH (2009). Role for protein−protein interaction databases in human genetics. Expert Rev. Proteom..

[65] Barabási AL, Gulbahce N, Loscalzo J (2011). Network medicine: a network-based approach to human disease. Nat. Rev. Genet..

[66] Boyanova D (2014). Functional module search in protein networks based on semantic similarity improves the analysis of proteomics data. Mol. Cell Proteom..

[67] Dweep H, Gretz N (2015). miRWalk2.0: a comprehensive atlas of microRNA-target interactions. Nat. Methods.

[68] Kuleshov MV (2016). Enrichr: a comprehensive gene set enrichment analysis web server 2016 update. Nucleic Acids Res..

[69] Lachmann A (2010). ChEA: transcription factor regulation inferred from integrating genome-wide ChIP-X experiments. Bioinformatics.

[70] Hsu SD (2011). miRTarBase: a database curates experimentally validated microRNA-target interactions. Nucleic Acids Res..

[71] Wang J, Lu M, Qiu C, Cui Q (2010). TransmiR: a transcription factor-microRNA regulation database. Nucleic Acids Res..

[72] Yang JH, Li JH, Jiang S, Zhou H, Qu LH (2013). ChIPBase: a database for decoding the transcriptional regulation of long non-coding RNA and microRNA genes from ChIP-Seq data. Nucleic Acids Res..

[73] Müssel C, Hopfensitz M, Kestler HA (2010). BoolNet—an R package for generation, reconstruction and analysis of Boolean networks. Bioinformatics.

[74] Fauré A, Naldi A, Chaouiya C, Thieffry D (2006). Dynamical analysis of a generic Boolean model for the control of the mammalian cell cycle. Bioinformatics.

[75] Albert I, Thakar J, Li S, Zhang R, Albert R (2008). Boolean network simulations for life scientists. Source Code Biol. Med.

