# Supplementary material for: An in-silico approach for discovery of microRNA-TF regulation of DISC1 interactome mediating neuronal migration
Source: NPJ Syst Biol Appl. 2019 May 7;5:17. doi: 10.1038/s41540-019-0094-3 (PMC6504871; doi:10.1038/s41540-019-0094-3)
Supplement: Supplementary file 1 — Supplementary information S1 [file 41540_2019_94_MOESM1_ESM.pdf]

An in-silico approach for discovery of microRNA-TF regulation of DISC1 interactome mediating neuronal migration: Supplementary Information

Authors: *John P. John[1,2 †], *Priyadarshini Thirunavukkarasu[1,2], Koko Ishizuka[3], Pravesh Parekh[1,2], and Akira Sawa[4]

Affiliation and Addresses: [1]Multimodal Brain Image Analysis Laboratory (MBIAL); [2]Department of Psychiatry, National Institute of Mental Health and Neurosciences (NIMHANS), Bangalore 560029, India

[3]Department of Psychiatry and Behavioral Sciences, Johns Hopkins University, School of Medicine, Baltimore, MD 21287, USA.

[4]Departments of Psychiatry, Mental Health, Neuroscience, and Biomedical Engineering, Johns Hopkins University School of Medicine, Bloomberg School of Public Health, Baltimore, MD 21287, USA.

* These authors contributed equally to this work

†Correspondence should be addressed to Dr. John P. John
Tel: (+91) 080-26993529,
Email: jpj@nimhans.ac.in, jpjnimhans@gmail.com

List of Tables

Supplementary Table S1: List of proteins that interact with DISC1, retrieved from GeneMania, NCBI Gene and APID databases 5

Supplementary Table S4: List of miRNAs from miRWALK targeting transcription factors, curated from miRTarBase 9

Supplementary Table S5: List of miRNA regulated by TFs curated from ChIPBase v2.0 and TransmiR v1.0 databases 12

Supplementary Table S6: List of 21 miRNA-TF feedback loops mutually regulated by overlapping 17 miRNAs and 11 TFs 13

Supplementary Table S7: Genes being regulated by miRNA-TF feedback loop 14

Supplementary Table S8: miRNA-TF feedback loop regulating functional module 1 of neuronal migration 15

Supplementary Table S9: miRNA-TF feedback loop regulating functional module 2 of neuronal migration 16

Supplementary Table S10: miRNA-TF feedback loop regulating functional module 3 of neuronal migration 17

Supplementary Table S11: miRNA-TF feedback loop regulating functional module 4 of neuronal migration 18

Supplementary Table S12: miRNA-TF feedback loop regulating functional module 5 of neuronal migration 19

Supplementary Table S13: miRNA-TF feedback loop regulating functional module 6 of neuronal migration 20

Supplementary Table S14: miRNA-TF feedback loop regulating functional module 7 of neuronal migration 21

Supplementary Table S15: miRNA-TF feedback loop regulating functional module 8 of neuronal migration 22

Supplementary Table S16: miRNA-TF feedback loops regulating two or more functional modules of migration 23

List of Figures

Supplementary Figure S1: Outline of the steps followed for identification of miRNA-TF feedback loops regulating the functional modules of neuronal migration...............................25

Supplementary Figure S2: miRNAs (N=17) and TFs (N=11) that regulate each other in feedback loops 26

Supplementary Figure S3: Perturbation analysis FM1 Simulation 1......................................27

Supplementary Figure S4: Perturbation analysis FM1 Simulation 2......................................28

Supplementary Figure S5: Perturbation analysis FM1 Simulation 3......................................29

Supplementary Figure S6: Perturbation analysis FM1 Simulation 4......................................30

Supplementary Figure S7: Perturbation analysis FM2 Simulation 1......................................32

Supplementary Figure S8: Perturbation analysis FM2 Simulation 2......................................34

Supplementary Figure S9: Perturbation analysis FM2 Simulation 3......................................36

Supplementary Figure S 10: Perturbation analysis FM2 Simulation 4...................................38

Supplementary Figure S11: Perturbation analysis FM3 Simulation 1...................................40

Supplementary Figure S12: Perturbation analysis FM3 Simulation 2....................................42

Supplementary Figure S13: Perturbation analysis FM3 Simulation 3....................................44

Supplementary Figure S14: Perturbation analysis FM3 Simulation 4....................................46

Supplementary Figure S15: Perturbation analysis FM4 Simulation 1....................................48

Supplementary Figure S16: Perturbation analysis FM4 Simulation 2....................................50

Supplementary Figure S17: Perturbation analysis FM4 Simulation 3....................................52

Supplementary Figure S18: Perturbation analysis FM4 Simulation 4....................................54

Supplementary Figure S19: Perturbation analysis FM5 Simulation 1....................................55

Supplementary Figure S20: Perturbation analysis FM5 Simulation 2....................................56

Supplementary Figure S21: Perturbation analysis FM5 Simulation 3....................................57

Supplementary Figure S22: Perturbation analysis FM5 Simulation 4....................................58

Supplementary Figure S23: Perturbation analysis FM6 Simulation 1....................................60

Supplementary Figure S24: Perturbation analysis FM6 Simulation 2....................................62

Supplementary Figure S25: Perturbation analysis FM6 Simulation 3....................................64

Supplementary Figure S26: Perturbation analysis FM6 Simulation 4....................................66

Supplementary Figure S27: Perturbation analysis FM7 Simulation 1....................................67

Supplementary Figure S28: Perturbation analysis FM7 Simulation 2....................................68

Supplementary Figure S29: Perturbation analysis FM7 Simulation 3....................................69

Supplementary Figure S30: Perturbation analysis FM7 Simulation 4....................................70
Supplementary Figure S31: Perturbation analysis FM8 Simulation 1....................................72
Supplementary Figure S32: Perturbation analysis FM8 Simulation 2....................................74
Supplementary Figure S33: Perturbation analysis FM8 Simulation 3....................................76
Supplementary Figure S34: Perturbation analysis FM8 Simulation 4....................................78
Supplementary Figure S35: Perturbation results of miR10b and KLF4 in feedback loop regulating functional modules 2 and 4..79
Supplementary Figure S36: Perturbation results of miR17 and STAT3 in feedback loop regulating functional modules 2 and 4..80
Supplementary Figure S37: Perturbation results of miR20a and CCND1 in feedback loop regulating functional modules 2 and 4..81
Supplementary Figure S38: Perturbation results of miR20a and STAT3 in feedback loop regulating functional modules 2 and 4..82
Supplementary Figure S39: Perturbation results of miR20b and STAT3 in feedback loop regulating functional modules 2 and 4..83
Supplementary Figure S40: Perturbation results of miR103a and CREB1 in feedback loop regulating functional modules 2, 3 and 4...84
Supplementary Figure S41: Perturbation results of miR17 and CCND1 in feedback loop regulating functional modules 2 and 4..85
Supplementary Figure S42: Perturbation results of miR155 and STAT3 in feedback loop regulating functional modules 2, 3, 6 and 8..86

Supplementary Table S1: List of proteins that interact with DISC1, retrieved from GeneMania, NCBI Gene and APID databases

Databases	Proteins that were shown to interact with DISC1					Proteins that were experimentally shown to interact with DISC1 and regulate neurodevelopmental functions		Proteins that were experimentally shown to interact with DISC1 /protein and regulate neurodevelopmental function, from all the 4 databases	
GeneMania	ATF5	ATF4	NDEL1	TRIO	IMMT	ATF5	ATF4	ATF4	ATF5
	GSK3B	CCDC141	CEP63	KIAA137	EIF3H	NDEL1	TRIO	NDEL1	TRIO
	PCNT	RASSF7	TRAF3IP1	GRIPAP1	KCNQ5	KALRN	NDE1	GSK3B	PCNT
	AKAP9	KATNAL1	MYO1A	NDE1	PPFIA4	GSK3B	PCNT	NDE1	MAP1A
	CEP290	DMTN	GPD2	MGAT3	SRR	MAP1A	MIC60 (IMMT)	FEZ1	ZNF365
	TENC1	AQP8	BDNF	CSF1R	KALRN	RASF7 (RASSF7)		PDE4B	KALRN
	MAP1A	MGST2	NKIRAS2	SEMA7A	SPTBN4	MIPT3 (TRAF3IP1)		TNIK	EXOC1
	SYNE1	ZRSR2	ATP8A2	BMP5	CACBN4	CC141 (CCDC141) **(*N*=13)**		SRR	MIC60 (IMMT)
	CDON	CFD	CHD3	CIT	CRHR2			1433E (YWHAE)	
	CRIM1	DEGS1	EDNRB	EFS	FAM155A			RASF7 (RASSF7)	
	FH	FKBP15	FLAD1	FRMD4A	GLRA1			GRDN (CCDC88A)	
	GPR173	GRIA2	GRPR	GUCY1A3	GZMA			CC141 (CCDC141)	
	HEY1	HIST1H3I	HSPG2	ITIH5	ITSN1			PAFAH1B1 (LIS1)	
	KCNJ1	KCTD15	KIAA0754	KIF17	KIR2DL4			TRAF3IP1 (MIPT3) **(*N*=22)**	
	LHB	MAPK8IP2	MKS1	NPPB	NR5A2				
	NRL	NUDT3	OR3A2	PDLIM5	NYX				

	PITPNC1	RAB24	RXRG	SCN2A	SLC34A1			
	SLC44A2	SLC4A7	SLCO3A1	SMARCE1	SNCAIP			
	SPTAN1	ST18	TEP1	TRIM52	TRPC7			
	WNT2B	ZBTB39	ZNF324	**(*N*=99)**				
NCBI Gene	ACTN2	ANKHD1	ATF4	ATF5	ATF7IP	ATF4	ATF5	
	EIF3H	ITSN1	MAP1A	NDEL1	PCNT	MAP1A	NDEL1	
	RANBP9	SPTBN4	TRAF3IP1	CCDC141	CIT	PCNT	FEZ1	
	FEZ1	KCNQ5	SYNE1	YWHAG	ACTG1	TNIK	TRIO	
	AGTBP1	AKAP6	AKAP9	ARIH2	BICD1	KALRN	NDE1	
	BZRAP1	C14orf166	CCDC136	CCDC24	CCDC88A	EXOC1	SRR	
	CDC5L	CDK5RAP3	CEP126	CEP170	CEP290	EXOC1	PDE4B	
	CEP57L1	CEP63	COL4A1	DCTN1	DCTN2	ZNF365	MIC60 (IMMT)	
	DMTN	DNAJC7	DPYSL3	DPYSL2	DISC1	1433E (YWHAE)		
	DST	DYNC1H1	EEF2	EIF3A	EIF3H	GRDN (CCDC88A)		
	EXOC1	EXOC4	EXOC7	FBXO41	FRYL	RASF7 (RASSF7)		
	GNBL	GNBTAB	GOLGA2	GPRASP2	HERC2P2	PAFAH1B1 (LIS1)		
	IFT20	IMMT	KALRN	KANSL1	KATNAL1	CC141 (CCDC141)		
	KIF3A	KIF3C	KIFAP3	MACF1	MATR3	MIPT3 (TRAF3IP1) **(*N*=22)**		
	NEFM	MEMO1	MGAT3	MLLT10	MPPED1			
	MVP	MYO1A	MYT1L	NDE1	NUP160			
	OLFM1	PAFAH1B1	PCNXL4	PDE4B	PGK1			

	PPFIA4	PPM1E	PPP4R1	PPP5C	RABGAP1			
	RAD21	RASSF7	RSBN	ROGDI	SH3BP5			
	SMARCE1	SMC2	SMC3	SNX6	SPARCL1			
	SPTAN1	SRGAP2	SRGAP3	SRR	STX18			
	SYBU	Srr	TFIP11	TIAM2	TNIK			
	TNKS	TRIO	TUBB	TUBB2A	UTRN			
	XPNPEP1	XRN2	YWHAE	YWHAQ	YWHAZ			
	ZNF197	ZNF365	**(*N*=122)**					
APID	SRR (Mouse)		TNKS2	ATF5	MCAF1	NDEL1	PCNT	
	CO6A2	CEP63	CCDC141	ITSN1	MIPT3	ATF5	FEZ1	
	RANB9	SYNE1	TBG1	PCNT	ACTG	MAP1A	PDE4B	
	CBPC1	AKAP9	ARI2	BICD1	PCX4	ZNF365	KALRN	
	MPPD1	MEMO1	CDC5L	CK5P3	CLUS	TNIK	TRIO	
	CRNL1	DCTN2	CC136	DMD	DNJC7	ATF4	NDE1	
	DYHC1	DPYL2	DPYL3	EF2	EXOC7	SRR	MIC60 (IMMT)	
	GBB1	GASP2	MIC60	CE170	KALRN	1443E (YWHAE)		
	FRYL	KIF3A	KIF3C	KIF3	MACF1	GRDN (CCDC88A)		
	MATR3	HERC2	MYT1L	NUL160	LIS1	RASF7 (RASSF7)		
	NOE1	PPM1E	PP4R1	RBGP1	RAD21	CC141(CCDC141)		
	EXOC1	3BP5	SMCE1	SMC2	SNX6	PAFAH1B1 (LIS1)		
	SPTNB1	SPTNB2	SRGP2	STX18	TFP11	MIPT3 (TRAF3IP1) **(*N*=20)**		

	Q59EY7	TNIK	TNKS1	TRIO	TBB5		
	TBB2A	UTRO	XPP1	XRN2	1443E		
	1443Z	ZN197	CN166	CE57L	DCTN1		
	ROGD1	FBX41	GNPTA	CCD24	PDE4B		
	PGK1	PPP5	Q8N4S1	1443T	ZNF365		
	FEZ1	CO4A1	1433G	CTRO	KCNQ5		
	SRGP3	TIAM2	EXOC4	SYBU	NFM		
	SMC3	AF10	AKAP6	IFT20	KANL1		
	NDE1	SYNEM	ACTN2	ANKH1	ATF4		
	EIF3H	RASF7	CE126	MAP1A	MYH7		
	NDEL1	SPTN4	DYST	GRDN	DISC1		
	SRR (Human)		RIMB1	MVP	CQ059		
	TCL1B	STX11	CRBA4	MYF6	SKP2		
	RIBC1	P4HA2	ASB3	CDC16	P4HA3		
	DC1I1	CELA	**(*N*=137)**				

Supplementary Table S4: List of miRNAs from miRWALK targeting transcription factors, curated from miRTarBase

No	List of miRNA (from miRWALK) that target genes involved in migration	TFs targeted by miRNAs, curated using miRTarBase
1.	hsa-let-7b-5p	AR, CCND1, HCFC1, NUCKS1, TRIM28, YAP1
2.	hsa-miR-124-3p	CLOCK, EGR1, EZH2, AR, KLF4, PIAS1, STAT3, TCF3
3.	hsa-miR-185-5p	AR, EZH2
4.	hsa-miR-19a-3p	CCND1
5.	hsa-miR-196a-5p	CCND1
6.	hsa-miR-3973	CCND1
7.	hsa-miR-92a-3p	CCND1, HCFC1, MYBL2, RNF2, RXR, SIN3B, STAT3, TRIM28
8.	hsa-miR-16-5p	CCND1, SMAD3, TTF2, YAP1
9.	hsa-miR-26b-5p	CCND1, CLOCK, EZH2, RNF2, RXR, SMC4, SOX3, YAP1
10.	hsa-miR-193b-3p	CCND1, EZH2, HCFC1, NUCKS1, SMAD3, TRIM28
11.	hsa-miR-155-5p	CCND1, MEF2A, NUCKS1, RNF2, SMAD2, SMAD3, SPI1, STAT3
12.	hsa-miR-302a-3p	CCND1
13.	hsa-miR-302c-3p	CCND1
14.	hsa-miR-365a-3p	CCND1
15.	hsa-miR-106b-5p	CCND1, CLOCK, STAT3
16.	hsa-miR-106a-5p	CCND1, STAT3
17.	hsa-miR-20a-5p	CCND1, PHC, STAT3
18.	hsa-miR-4311	CCND1
19.	hsa-miR-17-5p	CCND1, NUCKS1, SMAD3, STAT3, TCF3
20.	hsa-miR-15b-5p	SMAD3
21.	hsa-miR-6838-5p	CCND1
22.	hsa-miR-497-5p	CCND1
23.	hsa-miR-424-5p	CCND1, PIAS1, SPI1
24.	hsa-miR-195-5p	CCND1, SMAD3
25.	hsa-let-7f-5p	CCND1
26.	hsa-let-7e-5p	CCND1, STAT3
27.	hsa-miR-545-3p	CCND1
28.	hsa-miR-490-3p	CCND1
29.	hsa-miR-374b-5p	CCND1
30.	hsa-miR-425-5p	CCND1

31.	hsa-miR-183-5p	CCND1, EGR1, HCFC1, TRIM28, TTF2
32.	hsa-miR-93-5p	CCND1, EZH2, NUCKS1, STAT3
33.	hsa-miR-182-5p	CLOCK, CREB1
34.	hsa-miR-557	CLOCK
35.	hsa-miR-19b-3p	CLOCK, CREB1, MEF2A
36.	hsa-miR-33b-5p	CREB1
37.	hsa-miR-513c-3p	CREB1
38.	hsa-miR-513a-3p	CREB1
39.	hsa-miR-3606-3p	CREB1
40.	hsa-miR-103a-3p	CREB1, KLF4
41.	hsa-miR-215-5p	EGR1, NUCKS1, SMC4, TTF2
42.	hsa-miR-192-5p	EGR1, NUCKS1, SMC4, TTF2
43.	hsa-let-7a-5p	EZH2
44.	hsa-miR-320a	EZH2, TCF3
45.	hsa-miR-101-3p	EZH2
46.	hsa-miR-98-5p	EZH2
47.	hsa-miR-26a-5p	EZH2
48.	hsa-miR-199a-5p	EZH2
49.	hsa-miR-30d-5p	EZH2
50.	hsa-miR-484	NUCKS1, SMAD2, SMC4, TRIM28, HCFC1
51.	hsa-miR-222-3p	HCFC1
52.	hsa-miR-615-3p	HCFC1, TCF3
53.	hsa-miR-23a-3p	HCFC1
54.	hsa-miR-765	HNF4A
55.	hsa-miR-145-5p	KLF4, SMAD3
56.	hsa-miR-335-5p	KLF4, MEF2A, RXR, SIN3B, SMAD3
57.	hsa-miR-30b-5p	KLF4
58.	hsa-miR-10b-5p	KLF4
59.	hsa-miR-7-5p	KLF4
60.	hsa-miR-149-3p	MYBL2
61.	hsa-miR-30e-5p	MYBL2
62.	hsa-miR-93-3p	NUCKS1, TRIM28
63.	hsa-miR-877-3p	NUCKS1, TRIM28
64.	hsa-miR-423-3p	RXR
65.	hsa-miR-346	RXR

66.	hsa-miR-324-5p	SMAD2
67.	hsa-miR-188-5p	SMAD2
68.	hsa-miR-3529-3p	SMAD3
69.	hsa-miR-143-3p	SMAD3
70.	hsa-miR-18a-5p	SMAD3
71.	hsa-miR-125b-5p	STAT3
72.	hsa-miR-130a-5p	STAT3
73.	hsa-miR-20b-5p	STAT3
74.	hsa-miR-223-3p	TAL1
75.	hsa-miR-744-5p	TRIM28
76.	hsa-miR-93-3p	TRIM28
77.	hsa-miR-221-3p	TRIM28
78.	hsa-miR-30a-5p	TRIM28
79.	hsa-miR-375	YAP1
80.	hsa-miR-630	YAP1
81.	hsa-let-7g-3p	SMAD2

Supplementary Table S5: List of miRNA regulated by TFs curated from ChIPBase v2.0 and TransmiR v1.0 databases

List of TFs (from ChEA 2016 datasets)	miRNAs regulated by TFs, curated using ChIPBase v2.0	miRNA regulated by TF, curated using TransmiR v1.0 database
CREB1	hsa-miR-33b-5p<br>hsa-miR-103a-3p	hsa-miR-20a-5p<br>hsa-miR-17-5p
EGR1	hsa-miR-183-5p	hsa-miR-183-5p
KLF4	hsa-miR-145-5p<br>hsa-miR-10b-5p	
RXR	hsa-miR-423-3p	
SMAD2	hsa-miR-188-5p	
SMAD3	hsa-miR-145-5p	hsa-miR-145-5p<br>hsa-miR-143-3p
STAT3	hsa-miR-155-5p<br>hsa-miR-106a-5p<br>hsa-miR-20a-5p<br>hsa-miR-17-5p<br>hsa-miR-130a-5p<br>hsa-miR-20b-5p	hsa-miR-125b-5p
TAL1	hsa-miR-223-3p	
TCF3	hsa-miR-320a	
CCND1		hsa-miR-20a-5p<br>hsa-miR-17-5p

Supplementary Table S6: List of 21 miRNA-TF feedback loops mutually regulated by overlapping 17 miRNAs and 11 TFs

No	miRNA-TF feedback loop
1.	hsa-miR-20a-5p -CCND1
2.	hsa-miR-17-5p -CCND1
3.	hsa-miR-33b-5p -CREB1
4.	hsa-miR-103a-3p -CREB1
5.	hsa-miR-183-5p -EGR1
6.	hsa-miR-145-5p -KLF4
7.	hsa-miR-10b-5p -KLF4
8.	hsa-miR-145-5p -POU5F1
9.	hsa-miR-188-5p -SMAD2
10.	hsa-miR-145-5p -SMAD3
11.	hsa-miR-143-3p -SMAD3
12.	hsa-miR-223-3p -TAL1
13.	hsa-miR-320a -TCF3
14.	hsa-miR-423-3p -RXR
15.	hsa-miR-155-5p -STAT3
16.	hsa-miR-106a-5p -STAT3
17.	hsa-miR-20a-5p -STAT3
18..	hsa-miR-17-5p -STAT3
19.	hsa-miR-130a-5p-STAT3
20.	hsa-miR-125b-5p -STAT3
21.	hsa-miR-20b-5p -STAT3

Supplementary Table S7: Genes being regulated by miRNA-TF feedback loop

miRNA-TF feedback loop	Genes regulated by miRNA	Genes regulated by TF
hsa-miR-20a-5p-CCND1<br>hsa-miR-17-5p -CCND1	APP, PAFAH1B1<br>APP, PAFAH1B1	GSK3B, APP, DAB1, CDK5, RHEB, ACTB, NDEL1
hsa-miR-33b-5p -CREB1<br>hsa-miR-103a-3p -CREB1	ACTB<br>NDEL1	GSK3B, CCDC88A, CDK5, RHEB, SOX10, ACTB, PAFAH1B1, NDEL1
hsa-miR-183-5p -EGR1	GSK3B	PCM1, AKT1
hsa-miR-145-5p -KLF4<br>hsa-miR-10b-5p -KLF4	ACTB<br>PAFAH1B1	FOXD3, GSK3B, DAB1, CDK5, RHEB, DIXDC1, ACTB
hsa-miR-145-5p -POU5F1	ACTB	FOXD3, DAB1, RHEB, AKT1, NDEL1
hsa-miR-188-5p -SMAD2	AKT1	APP, CCDC88A, CDK5, RHEB, ACTB, PAFAH1B1, ZNF365
hsa-miR-145-5p -SMAD3<br>hsa-miR-143-3p -SMAD3	ACTB<br>AKT1	APP, CCDC88A, CDK5, RHEB, ACTB, PAFAH1B1, ZNF365
hsa-miR-223-3p -TAL1	ZNF365	RHEB, AKT1, DISC1, ACTB, NDEL1, CCDC88A, PCM1, PAFAH1B1
hsa-miR-320a -TCF3	ACTB	GSK3B, PCM1, RHEB, AKT1, DISC1
hsa-miR-423-3p -RXR	APP	PCM1, DIXDC1, AKT1, CCDC141, ACTB
hsa-miR-155-5p -STAT3<br>hsa-miR-106a-5p -STAT3<br>hsa-miR-20a-5p -STAT3<br>hsa-miR-17-5p -STAT3<br>hsa-miR-130a-5p -STAT3<br>hsa-miR-125b-5p -STAT3<br>hsa-miR-20b-5p -STAT3	AKT1, RHEB, CDK5, GSK3B<br>APP<br>APP, PAFAH1B1<br>APP, PAFAH1B1<br>APP<br>AKT1<br>PAFAH1B1	FOXD3, GSK3B, APP, PCM1, DAB1, CDK5, RHEB, DISC1, BBS4, ZNF365

Supplementary Table S8: miRNA-TF feedback loop regulating functional module 1 of neuronal migration

Genes in FM1	miRNA-TF loop regulating genes in FM1[a]	Regulation of miRNA by TF in feedback loop[b]	Genes regulated by TF in the feedback loop[c]	Genes regulated by miRNA in the feedback loop[d]
PCM1, BBS4 and DISC1	hsa-miR-183-5p-EGR1	Not reported	PCM1	None
	hsa-miR-223-3p-TAL1	Not reported	PCM1, DISC1	None
	hsa-miR-320a-TCF3	Not reported	PCM1, DISC1	None
	hsa-miR-155-5p-STAT3	Not reported	PCM1, BBS4, DISC1	None
	hsa-miR-106a-5p-STAT3	Not reported		None
	hsa-miR-20a-5p-STAT3	Not reported		None
	hsa-miR-17-5p-STAT3	Not reported		None
	hsa-miR-130a-5p-STAT3	Not reported		None
	hsa-miR-125b-5p-STAT3	Activation		None
	hsa-miR-20b-5p-STAT3	Not reported		None
	hsa-miR-423-3p-RXR	Not reported	PCM1	None

[a] **curated from miRWALK2.0 and TransmiR v1.0 databases**

[b] **curated from TransmiR v1.0 and ChIPBase v2.0 databases**

[c] **curated from ChEA 2016 datasets**

[d] **curated from miRWALK2.0 database**

Supplementary Table S9: miRNA-TF feedback loop regulating functional module 2 of neuronal migration

Genes in FM2	miRNA-TF loop regulating genes in FM2[a]	Regulation of miRNA by TF in feedback loop[b]	Genes regulated by TF in the feedback loop[c]	Genes regulated by miRNA in the feedback loop[d]
ZNF365, GSK3B, NDEL1, PAFAH1B1 and DISC1	hsa-miR-188-5p-SMAD2	Not reported	ZNF365, PAFAH1B1	None
	hsa-miR-145-5p-SMAD3	Activation	ZNF365, PAFAH1B1	None
	hsa-miR-143-3p-SMAD3	Activation		None
	hsa-miR-155-5p-STAT3	Not reported	ZNF365, GSK3B, DISC1	GSK3B
	hsa-miR-106a-5p-STAT3	Not reported		None
	hsa-miR-20a-5p-STAT3	Not reported		PAFAH1B1
	hsa-miR-17-5p-STAT3	Not reported		PAFAH1B1
	hsa-miR-130a-5p-STAT3	Not reported		None
	hsa-miR-125b-5p-STAT3	Activation		None
	hsa-miR-20b-5p-STAT3	Not reported		PAFAH1B1
	hsa-miR-145-5p-KLF4	Not reported	GSK3B, PAFAH1B1	None
	hsa-miR-10b-5p-KLF4	Not reported		PAFAH1B1
	hsa-miR-320a-TCF3	Not reported	GSK3B, DISC1	None
	hsa-miR-223-3p-TAL1	Not reported	DISC1, NDEL1, PAFAH1B1	ZNF365
	hsa-miR-20a-5p-CCND1	Activation	GSK3B, NDEL1	PAFAH1B1
	hsa-miR-17-5p-CCND1	Activation		PAFAH1B1
	hsa-miR-33b-5p-CREB1	Not reported	GSK3B, NDEL1	None
	hsa-miR-103a-3p-CREB1	Not reported		NDEL1
	hsa-miR-145-5p-POU5F1	Not reported	NDEL1	None

[a] **curated from miRWALK2.0 and TransmiR v1.0 databases**

[b] **curated from TransmiR v1.0 and ChIPBase v2.0 databases**

[c] **curated from ChEA 2016 datasets**

[d] **curated from miRWALK2.0 database**

Supplementary Table S10: miRNA-TF feedback loop regulating functional module 3 of neuronal migration

Genes in FM 3	miRNA-TF loop regulating genes in FM3[a]	Regulation of miRNA by TF in feedback loop[b]	Genes regulated by TF in the feedback loop[c]	Genes regulated by miRNA in the feedback loop[d]
CDK5, DIXDC1, DISC1 and NDEL1	hsa-miR-20a-5p-CCND1	Activation	CDK5, NDEL1	None
	hsa-miR-17-5p-CCND1	Activation		None
	hsa-miR-33b-5p-CREB1	Not reported	CDK5, NDEL1	None
	hsa-miR-103a-3p-CREB1	Not reported		NDEL1
	hsa-miR-145-5p-KLF4	Not reported	CDK5, DIXDC1	None
	hsa-miR-10b-5p-KLF4	Not reported		None
	hsa-miR-188-5p-SMAD2	Not reported	CDK5	None
	hsa-miR-145-5p-SMAD3	Activation	CDK5	None
	hsa-miR-143-3p-SMAD3	Activation		None
	hsa-miR-423-3p-RXR	Not reported	DIXDC1	None
	hsa-miR-145-5p-POU5F1	Not reported	NDEL1	None
	hsa-miR-223-3p-TAL1	Not reported	NDEL1, DISC1	None
	hsa-miR-320a-TCF3	Not reported	DISC1	None
	hsa-miR-155-5p-STAT3	Not reported	DISC1	CDK5
	hsa-miR-106a-5p-STAT3	Not reported		None
	hsa-miR-20a-5p-STAT3	Not reported		None
	hsa-miR-17-5p-STAT3	Not reported		None
	hsa-miR-130a-5p-STAT3	Not reported		None
	hsa-miR-125b-5p-STAT3	Activation		None
	hsa-miR-20b-5p-STAT3	Not reported		None

[a] **curated from miRWALK2.0 and TransmiR v1.0 databases**

[b] **curated from TransmiR v1.0 and ChIPBase v2.0 databases**

[c] **curated from ChEA 2016 datasets**

[d] **curated from miRWALK2.0 database**

Supplementary Table S11: miRNA-TF feedback loop regulating functional module 4 of neuronal migration

Genes in FM4	miRNA-TF loop regulating genes in FM4[a]	Regulation of miRNA by TF in feedback loop[b]	Genes regulated by TF in the feedback loop[c]	Genes regulated by miRNA in the feedback loop[d]
APP, DAB1, NDEL1, PAFAH1B1 and DISC1	hsa-miR-20a-5p-CCND1	Activation	APP, DAB1, NDEL1	APP, PAFAH1B1
	hsa-miR-17-5p-CCND1	Activation		APP, PAFAH1B1
	hsa-miR-188-5p-SMAD2	Not reported	APP, PAFAH1B1	None
	hsa-miR-145-5p-SMAD3	Activation	APP, PAFAH1B1	None
	hsa-miR-143-3p-SMAD3	Activation		None
	hsa-miR-155-5p-STAT3	Not reported	APP, DAB1, DISC1	None
	hsa-miR-106a-5p-STAT3	Not reported		APP
	hsa-miR-20a-5p-STAT3	Not reported		APP, PAFAH1B1
	hsa-miR-17-5p-STAT3	Not reported		APP, PAFAH1B1
	hsa-miR-130a-5p-STAT3	Not reported		APP
	hsa-miR-125b-5p-STAT3	Activation		None
	hsa-miR-20b-5p-STAT3	Not reported		PAFAH1B1
	hsa-miR-145-5p-KLF4	Not reported	DAB1, PAFAH1B1	None
	hsa-miR-10b-5p-KLF4	Not reported		PAFAH1B1
	hsa-miR-145-5p-POU5F1	Not reported	DAB1	None
	hsa-miR-223-3p-TAL1	Not reported	NDEL1, DISC1, PAFAH1B1	None
	hsa-miR-320a-TCF3	Not reported	DISC1	None

[a] **curated from miRWALK2.0 and TransmiR v1.0 databases**

[b] **curated from TransmiR v1.0 and ChIPBase v2.0 databases**

[c] **curated from ChEA 2016 datasets**

[d] **curated from miRWALK2.0 database**

Supplementary Table S12: miRNA-TF feedback loop regulating functional module 5 of neuronal migration

Genes in FM5	miRNA-TF loop regulating genes in FM5[a]	Regulation of miRNA by TF in feedback loop[b]	Genes regulated by TF in the feedback loop[c]	Genes regulated by miRNA in the feedback loop[d]
CCDC141, MYH2 and DISC1	hsa-miR-223-3p-TAL1	Not reported	DISC1	None
	hsa-miR-320a-TCF3	Not reported	DISC1	None
	hsa-miR-155-5p-STAT3	Not reported		None
	hsa-miR-106a-5p-STAT3	Not reported		None
	hsa-miR-20a-5p-STAT3	Not reported		None
	hsa-miR-17-5p-STAT3	Not reported	DISC1	None
	hsa-miR-130a-5p-STAT3	Not reported		None
	hsa-miR-125b-5p-STAT3	Activation		None
	hsa-miR-20b-5p-STAT3	Not reported		None
	hsa-miR-423-3p-RXR	Not reported	CCDC141	None

[a] **curated from miRWALK2.0 and TransmiR v1.0 databases**

[b] **curated from TransmiR v1.0 and ChIPBase v2.0 databases**

[c] **curated from ChEA 2016 datasets**

[d] **curated from miRWALK2.0 database**

Supplementary Table S13: miRNA-TF feedback loop regulating functional module 6 of neuronal migration

Genes in FM6	miRNA-TF loop regulating genes in FM6[a]	Regulation of miRNA by TF in feedback loop[b]	Genes regulated by TF in the feedback loop[c]	Genes regulated by miRNA in the feedback loop[d]
ACTB, AKT1, CCDC88A and DISC1	hsa-miR-33b-5p-CREB1	Not reported	ACTB, CCDC88A	ACTB
	hsa-miR-103a-3p-CREB1	Not reported		None
	hsa-miR-145-5p-KLF4	Not reported	ACTB	ACTB
	hsa-miR-10b-5p-KLF4	Not reported		None
	hsa-miR-188-5p-SMAD2	Not reported	ACTB, CCDC88A	AKT1
	hsa-miR-145-5p-SMAD3	Activation	ACTB, CCDC88A	ACTB
	hsa-miR-143-3p-SMAD3	Activation		None
	hsa-miR-223-3p-TAL1	Not reported	ACTB, AKT1, DISC1, CCDC88A	None
	hsa-miR-423-3p-RXR	Not reported	ACTB, AKT1	None
	hsa-miR-20a-5p-CCND1	Activation	ACTB	None
	hsa-miR-17-5p-CCND1	Activation		None
	hsa-miR-183-5p-EGR1	Not reported	AKT1	None
	hsa-miR-145-5p-POU5F1	Not reported	AKT1	ACTB
	hsa-miR-320a-TCF3	Not reported	DISC1, AKT1	ACTB
	hsa-miR-155-5p-STAT3	Not reported	DISC1	AKT1
	hsa-miR-106a-5p-STAT3	Not reported		None
	hsa-miR-20a-5p-STAT3	Not reported		None
	hsa-miR-17-5p-STAT3	Not reported		None
	hsa-miR-130a-5p-STAT3	Not reported		None
	hsa-miR-125b-5p-STAT3	Activation		AKT1
	hsa-miR-20b-5p-STAT3	Not reported		None

[a] **curated from miRWALK2.0 and TransmiR v1.0 databases**

[b] **curated from TransmiR v1.0 and ChIPBase v2.0 databases**

[c] **curated from ChEA 2016 datasets**

[d] **curated from miRWALK2.0 database**

Supplementary Table S14: miRNA-TF feedback loop regulating functional module 7 of neuronal migration

Genes in FM7	miRNA-TF loop regulating genes in FM7[a]	Regulation of miRNA by TF in feedback loop[b]	Genes regulated by TF in the feedback loop[c]	Genes regulated by miRNA in the feedback loop[d]
SOX10, FOXD3 and DISC1	hsa-miR-320a-TCF3	Not reported	DISC1	None
	hsa-miR-223-3p-TAL1	Not reported	DISC1	None
	hsa-miR-155-5p-STAT3	Not reported	DISC1, FOXD3	None
	hsa-miR-106a-5p-STAT3	Not reported		None
	hsa-miR-20a-5p-STAT3	Not reported		None
	hsa-miR-17-5p-STAT3	Not reported		None
	hsa-miR-130a-3p-STAT3	Not reported		None
	hsa-miR-125b-5p-STAT3	Activation		None
	hsa-miR-20b-5p-STAT3	Not reported		None
	hsa-miR-33b-5p-CREB1	Not reported	SOX10	None
	hsa-miR-145-5p-KLF4	Not reported	FOXD3	None
	hsa-miR-10b-5p-KLF4	Not reported		None
	hsa-miR-145-5p-POU5F1	Not reported		None

[a] **curated from miRWALK2.0 and TransmiR v1.0 databases**

[b] **curated from TransmiR v1.0 and ChIPBase v2.0 databases**

[c] **curated from ChEA 2016 datasets**

[d] **curated from miRWALK2.0 database**

Supplementary Table S15: miRNA-TF feedback loop regulating functional module 8 of neuronal migration

Genes in FM8	miRNA-TF loop regulating genes in FM8[a]	Regulation of miRNA by TF in feedback loop[b]	Genes regulated by TF in the feedback loop[c]	Genes regulated by miRNA in the feedback loop[d]
RHEB and DISC1	hsa-miR-20a-5p-CCND1	Activation	RHEB	None
	hsa-miR-17-5p-CCND1	Not reported		None
	hsa-miR-33b-5p-CREB1	Not reported	RHEB	None
	hsa-miR-103a-3p-CREB1	Not reported		None
	hsa-miR-145-5p-KLF4	Not reported	RHEB	None
	hsa-miR-10b-5p-KLF4	Not reported		None
	hsa-miR-320a-TCF3	Not reported	RHEB, DISC1	None
	hsa-miR-223-3p-TAL1	Not reported	RHEB, DISC1	None
	hsa-miR-188-5p-SMAD2	Not reported	RHEB	None
	hsa-miR-145-5p-SMAD3	Activation	RHEB	None
	hsa-miR-143-3p-SMAD3	Activation		None
	hsa-miR-145-5p-POU5F1	Not reported	RHEB	None
	hsa-miR-155-5p-STAT3	Not reported	RHEB, DISC1	RHEB
	hsa-miR-106a-5p-STAT3	Not reported		None
	hsa-miR-20a-5p-STAT3	Not reported		None
	hsa-miR-17-5p-STAT3	Not reported		None
	hsa-miR-130a-5p-STAT3	Not reported		None
	hsa-miR-125b-5p-STAT3	Activation		None
	hsa-miR-20b-5p-STAT3	Not reported		None

[a] **curated from miRWALK2.0 and TransmiR v1.0 databases**

[b] **curated from TransmiR v1.0 and ChIPBase v2.0 databases**

[c] **curated from ChEA 2016 datasets**

[d] **curated from miRWALK2.0 database**

Supplementary Table S16: miRNA-TF feedback loops regulating two or more functional modules of migration

No	miRNA-TF feedback loop	Functional modules regulated
1.	hsa-miR-155-5p-STAT3	FM2 (**ZNF365**, ***<u>GSK3B</u>***, **DISC1**, NDEL1, PAFAH1B1);<br>FM3 (***<u>CDK5</u>***, DIXDC1, **DISC1**, NDEL1);<br>FM6 (*AKT1*, ACTB, **DISC1**, CCDC88A);<br>FM8 (**DISC1**, ***<u>RHEB</u>***)
2.	hsa-miR-20a-5p-STAT3	FM2 (**ZNF365, GSK3B**, **DISC1**, NDEL1, *PAFAH1B1*);<br>FM4 (***<u>APP</u>***, **DAB1, DISC1**, NDEL1, *PAFAH1B1*)
3.	hsa-miR-17-5p-STAT3	FM2 (**ZNF365, GSK3B**, **DISC1**, NDEL1, *PAFAH1B1*);<br>FM4 (***<u>APP</u>***, **DAB1, DISC1**, NDEL1, *PAFAH1B1*)
4.	hsa-miR-20b-5p-STAT3	FM2 (**ZNF365, GSK3B**, **DISC1**, NDEL1, *PAFAH1B1*);<br>FM4 (**APP**, **DAB1, DISC1**, NDEL1, *PAFAH1B1*)
5.	hsa-miR-20a-5p-CCND1	FM2 (ZNF365**, GSK3B**, DISC1, **NDEL1**, *PAFAH1B1*);<br>FM4 (***<u>APP</u>***, **DAB1,** DISC1, NDEL1, *PAFAH1B1*)
6.	hsa-miR-17-5p-CCND1	FM2 (ZNF365**, GSK3B**, DISC1, **NDEL1**, *PAFAH1B1*);<br>FM4 (***<u>APP</u>***, **DAB1,** DISC1, NDEL1, *PAFAH1B1*)
7.	hsa-miR-103a-3p-CREB1	FM2 (ZNF365, **GSK3B**, DISC1, ***<u>NDEL1</u>***, **PAFAH1B1**);<br>FM3 (**CDK5**, DIXDC1, DISC1, ***<u>NDEL1</u>***);<br>FM4 (APP, DAB1, DISC1, ***<u>NDEL1</u>***, **PAFAH1B1**)
8.	hsa-miR-10b-5p-KLF4	FM2 (ZNF365**, GSK3B**, DISC1, NDEL1, *PAFAH1B1*);<br>FM4 (APP, **DAB1**, DISC1, NDEL1, *PAFAH1B1*)

Genes highlighted in bold are genes regulated by TF in the miRNA-TF feedback loop;

Genes italicized are genes regulated by miRNA in the miRNA-TF feed-back loop;

Genes highlighted in bold, italicized and underlined are genes regulated by both miRNA and TF in the miRNA-TF feedback loop

Supplementary Figures: Summary

Supplementary Figure S1: shows the steps followed for identification of miRNA-TF feedback loops regulating the functional modules of neuronal migration

Supplementary Figure S2: shows miRNAs (N=17) and TFs (N=11) regulating each other in feedback loop

Supplementary Figures S3-S34: shows the effect of perturbation of gene/TF/miRNA in each functional module of migration (i.e. activation frequency of migration by perturbed node) in percentage and plotted for 140-time steps. Inset in each graph show the perturbation results for the first 20-time steps.

Supplementary Figures S35-S42: shows the effect of perturbation of miRNA-TF feedback loops in each functional module of migration in percentage and plotted at t=0 and t=140.

100% - Upregulation of migration

0% - Downregulation of migration

Between 0 and 100% - Migration is regulated at basal level

Perturbations (overexpression, OE and knock out, KO) have been carried out for four different simulations.

Simulation 1 - TF activates gene and miRNA expression

Simulation 2 - TF represses gene and miRNA expression

Simulation 3 - TF activates gene and represses miRNA expression

Simulation 4 - TF activates miRNA and represses gene expression

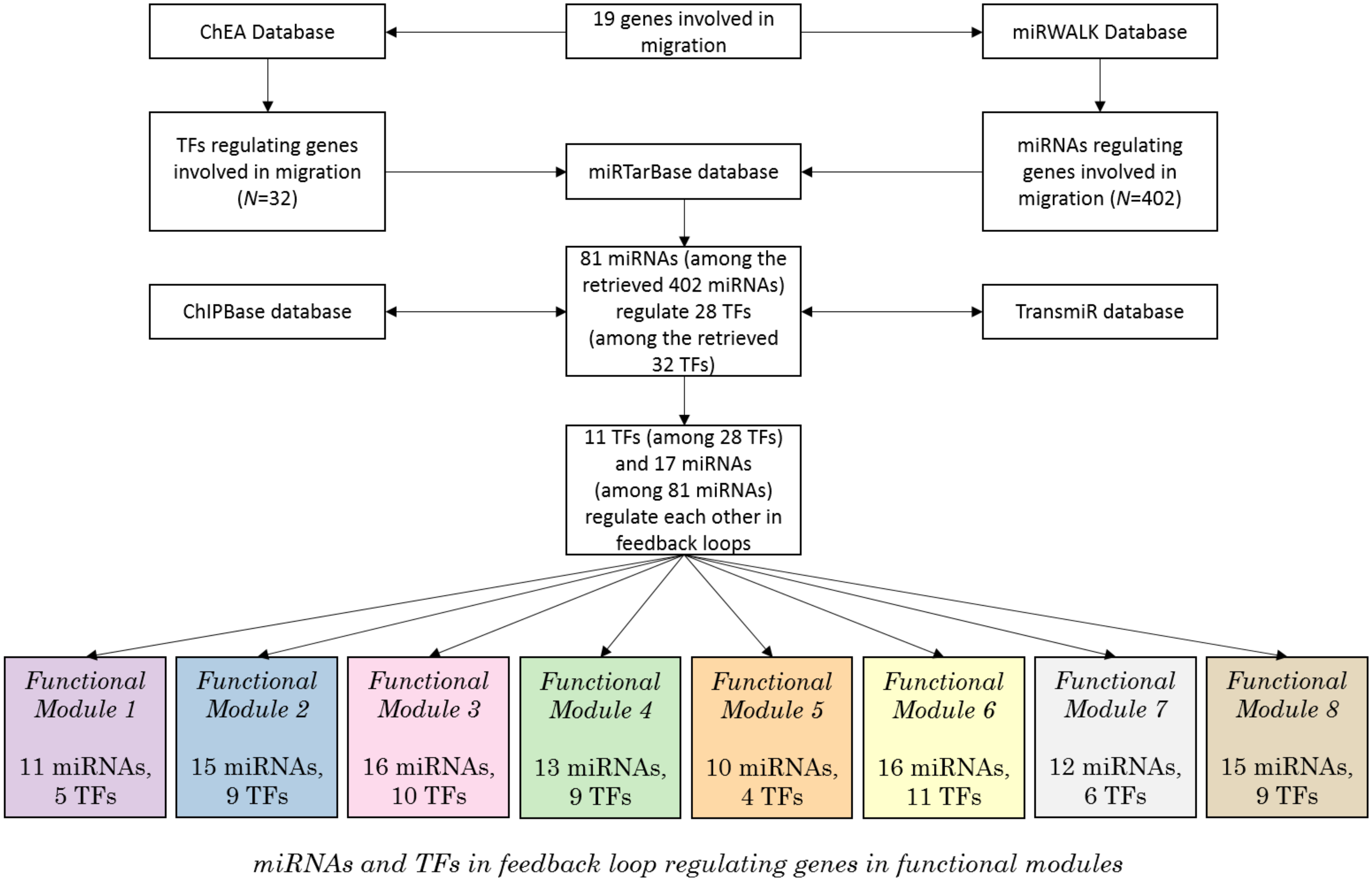

Supplementary Figure S1: Outline of the steps followed for identification of miRNA-TF feedback loops regulating the functional modules of neuronal migration

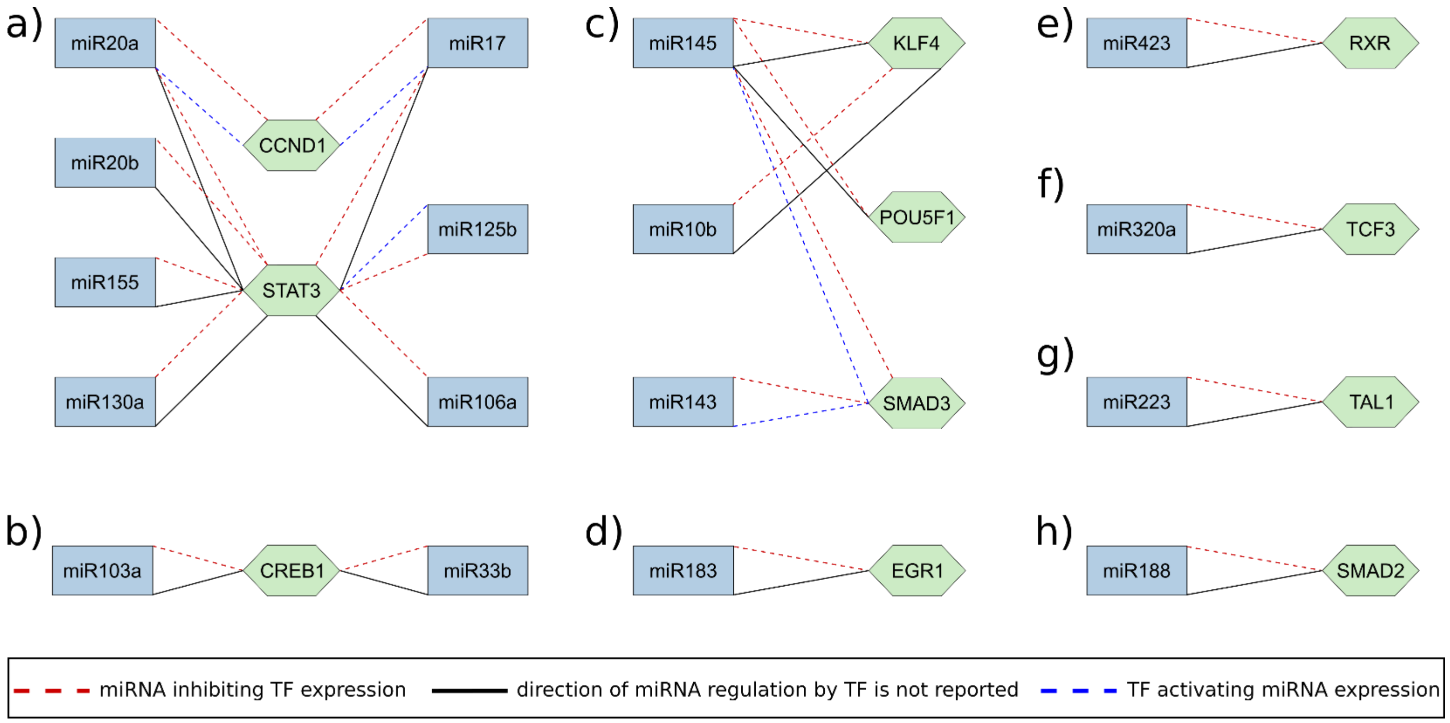

Supplementary Figure S2: miRNAs (N=17) and TFs (N=11) that regulate each other in feedback loops

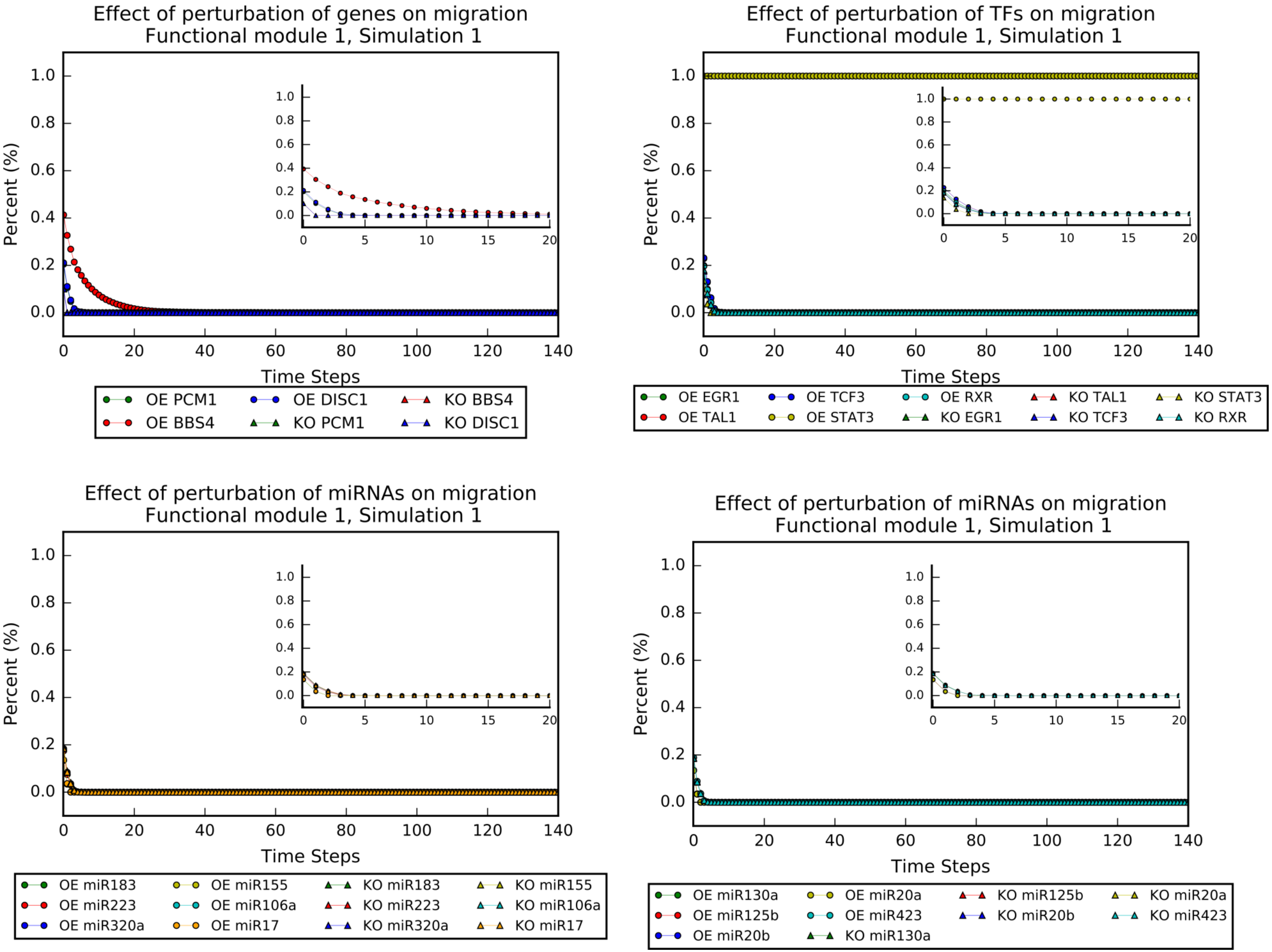

Supplementary Figure S3: Perturbation analysis FM1 Simulation 1

TF activates gene and miRNA expression. Perturbation of each gene did not upregulate migration, as expression of all three genes is essential for migration in FM1. When each TF was overexpressed (OE) (except for STAT3), migration was downregulated as miRNAs (activated by TF) inhibit gene expression. When each TF was knocked out (KO), migration was downregulated, as miRNAs downregulated gene expression. OE of STAT3, upregulated migration, as STAT3 activated expression of PCM1, DISC1 and BBS4 genes.
Perturbation of each miRNA showed downregulation of migration, as when miRNA was OE, it downregulated TF and gene expression. When miRNA was knocked out, TF or gene expression gets inhibited by expressed miRNAs in the functional module.

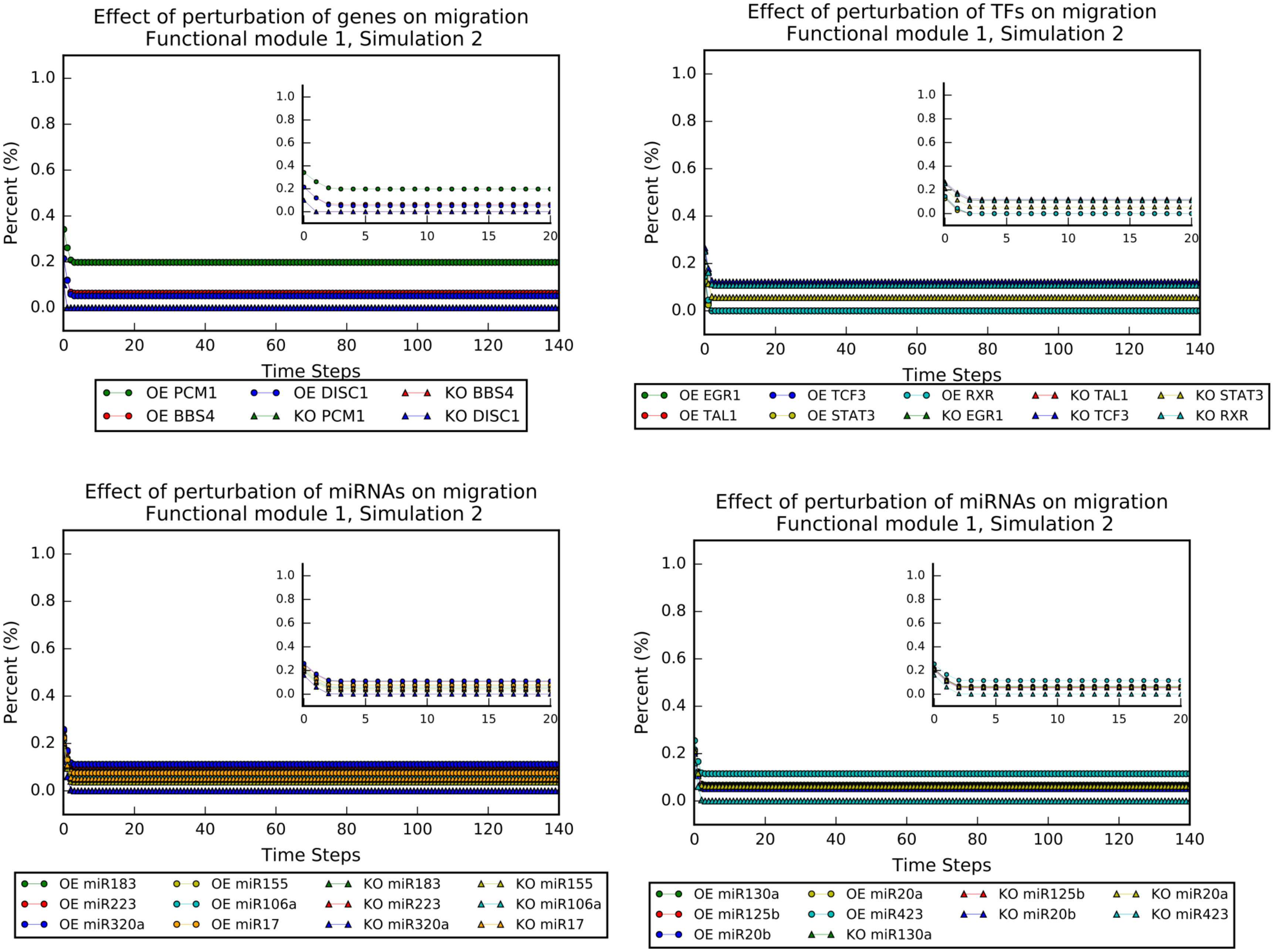

Supplementary Figure S4: Perturbation analysis FM1 Simulation 2

TF represses gene and miRNA expression. Perturbation of each gene did not upregulate migration as expression of all three genes is essential for migration in FM1. Overexpression (OE) of each TF showed downregulation of migration, as TFs repressed gene expression; Knock-out (KO) of each TF did not show upregulation of migration, as expressed miRNAs or TFs in the module inhibited gene expression.
OE of each miRNA did not show upregulation of migration, as miRNA repressed gene expression. KO of each miRNA also did not show upregulation of migration, as TFs/miRNAs which are expressed downregulated gene expression

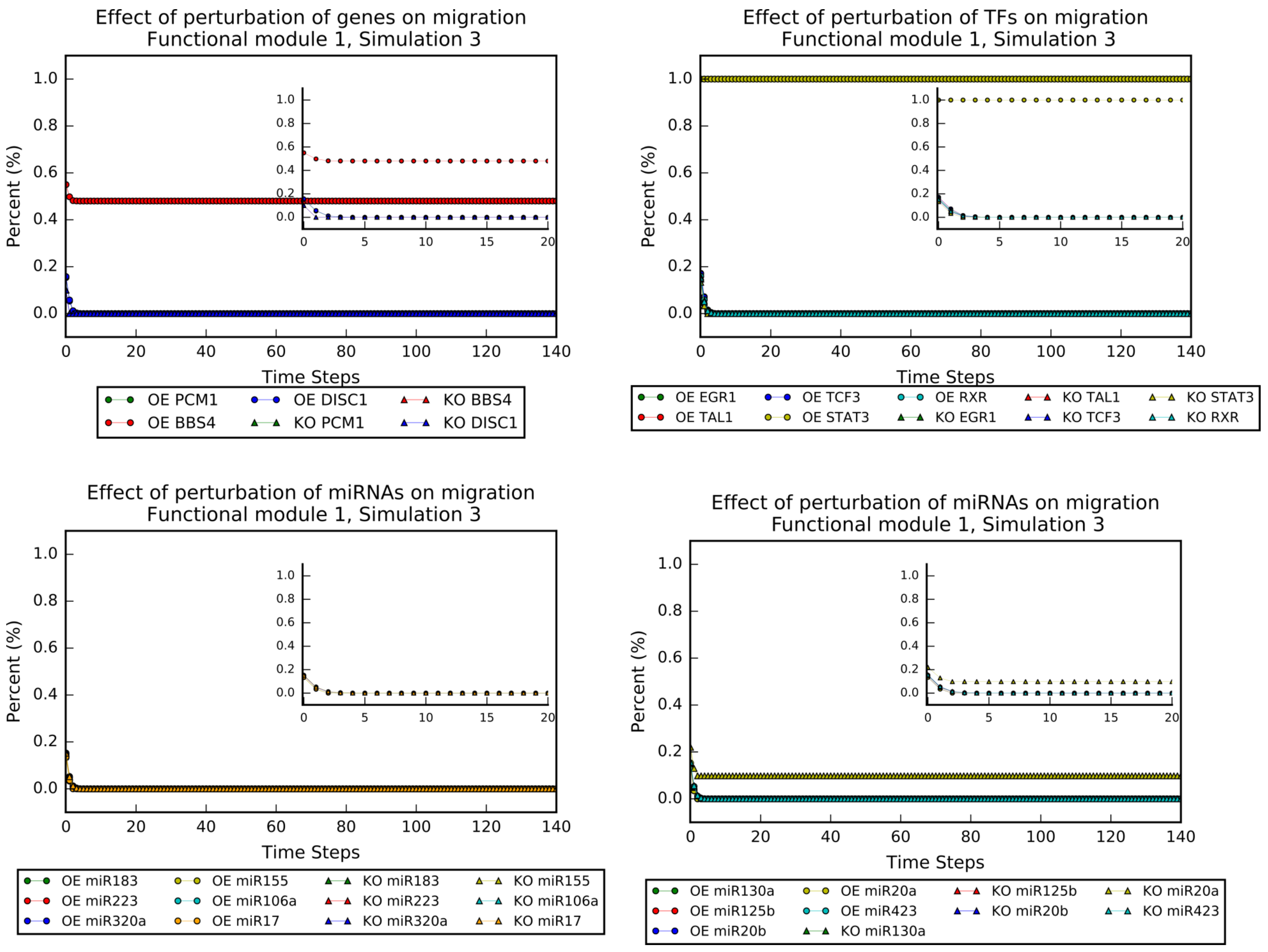

Supplementary Figure S5: Perturbation analysis FM1 Simulation 3

TF activates gene and inhibits miRNA expression. Overexpression (OE) of BBS4 showed regulation of migration (48%). When BBS4 gene was OE, DISC1 and PCM1 gets expressed. But, here migration was not upregulated, as OE of gene could not overcome the inhibition by miRNA. Knock out (KO) of each gene did not upregulate migration as expression of all three genes is essential for migration in FM1.
When each TF was OE (except STAT3), migration was downregulated as miRNA inhibited gene expression. But, when STAT3 was OE, it upregulated migration as STAT3 activated expression of PCM1, BBS4 and DISC1. When each TF was KO, migration was downregulated as gene expression gets repressed. When each miRNA was OE, migration was downregulated as miRNA repressed TF or gene expression. When each miRNA was KO (except miR125b), migration was not upregulated as expression of TF/gene gets inhibited by expressed miRNAs (which regulate other genes in the module)

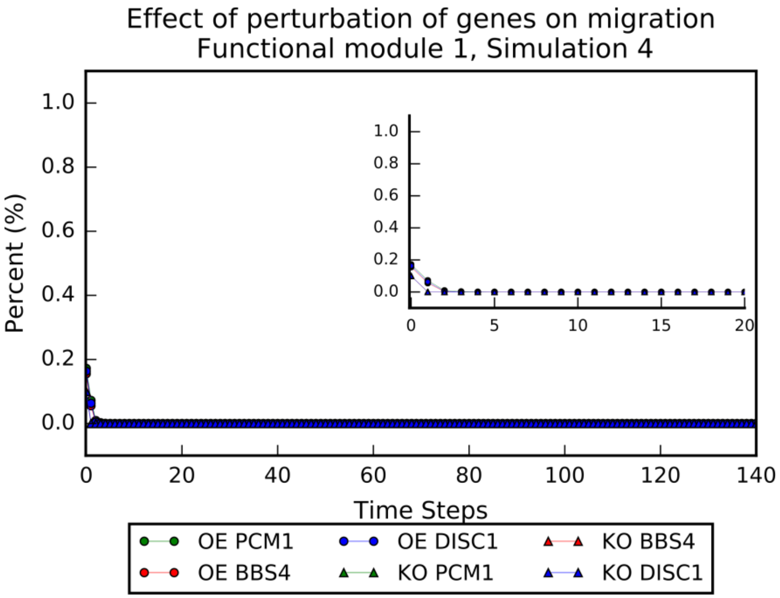

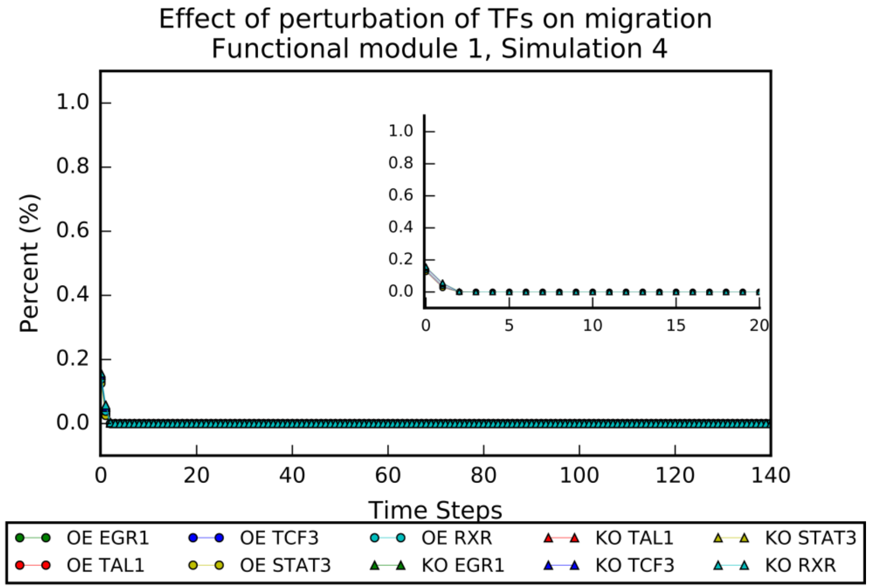

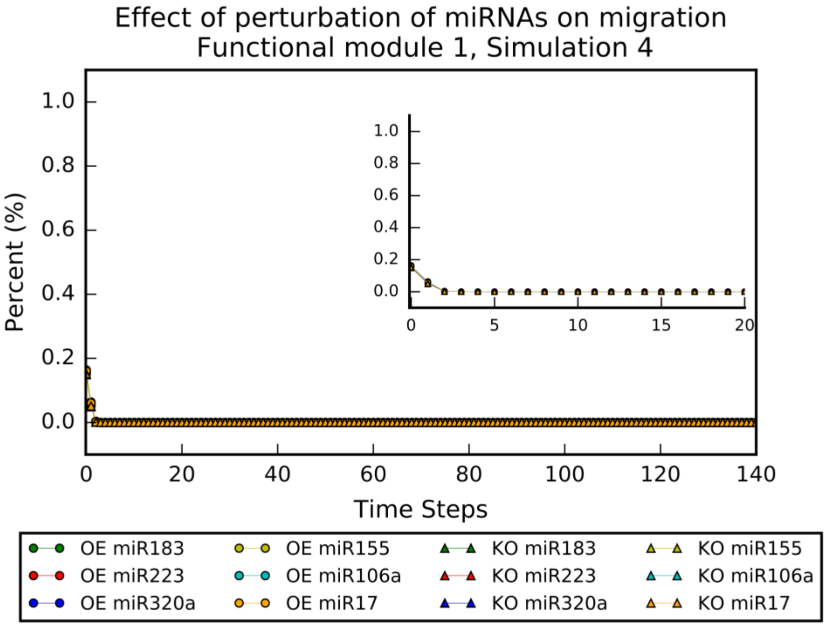

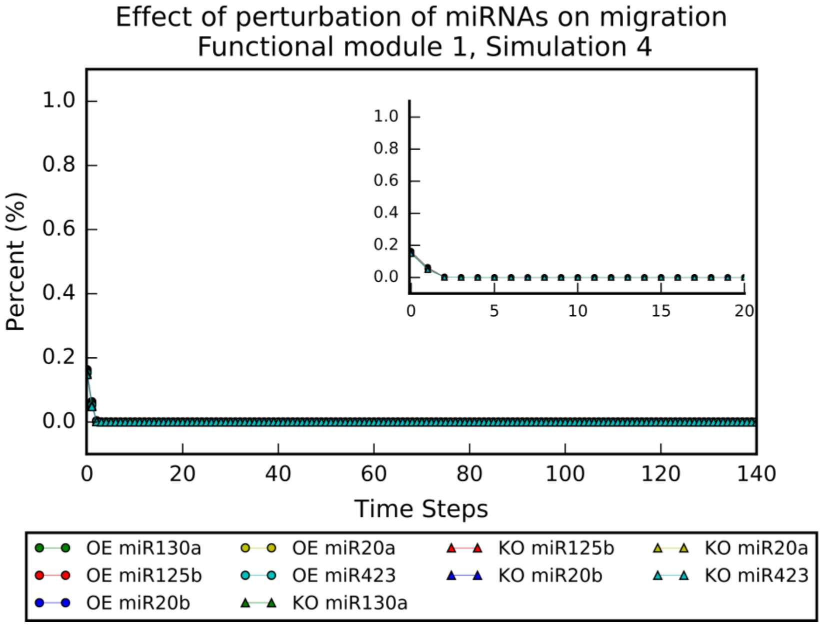

Supplementary Figure S6: Perturbation analysis FM1 Simulation 4

TF activates miRNA and inhibits gene expression. Perturbation of each gene did not upregulate migration as expression of all three genes is essential for migration in FM1. When each TF was overexpressed (OE), migration was downregulated (0%) as TF repressed gene expression. When each TF was knocked out (KO), migration was downregulated as expressed TFs in the module inhibited gene expression. When each miRNA was OE, TF expression gets downregulated and the gene expression gets inhibited by expressed TFs (which regulate other genes in the module), leading to downregulation of migration (0%). When each miRNA was KO, migration was downregulated as TF expression gets upregulated, which in turn inhibits gene expression.

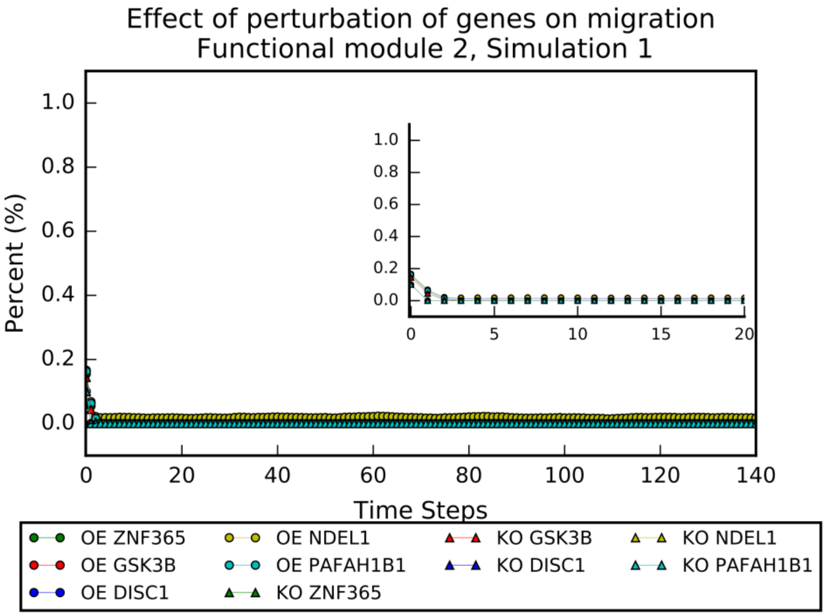
Effect of perturbation of genes on migration
Functional module 2, Simulation 1
Percent (%)
Time Steps
OE ZNF365
OE GSK3B
OE DISC1
OE NDEL1
OE PAFAH1B1
KO ZNF365
KO GSK3B
KO DISC1
KO NDEL1
KO PAFAH1B1

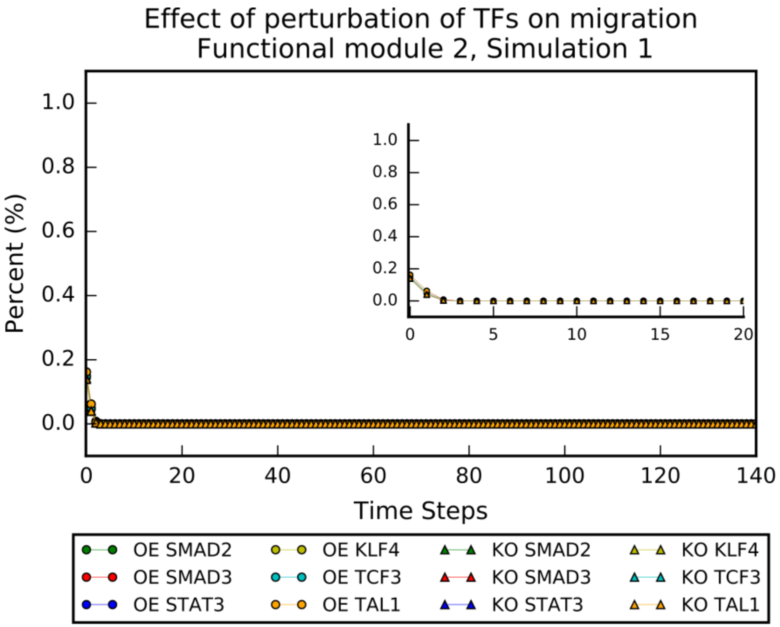
Effect of perturbation of TFs on migration
Functional module 2, Simulation 1
Percent (%)
Time Steps
OE SMAD2
OE SMAD3
OE STAT3
OE KLF4
OE TCF3
OE TAL1
KO SMAD2
KO SMAD3
KO STAT3
KO KLF4
KO TCF3
KO TAL1

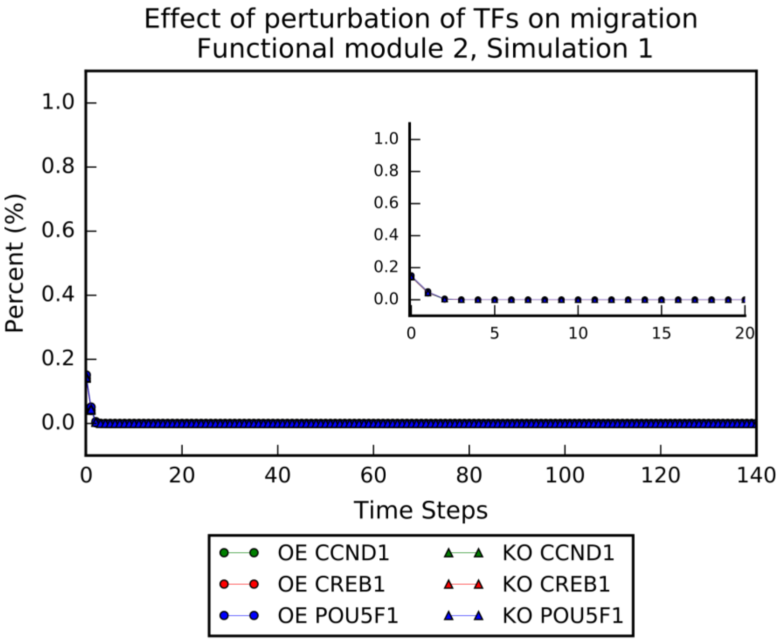
Effect of perturbation of TFs on migration
Functional module 2, Simulation 1
Percent (%)
Time Steps
OE CCND1
OE CREB1
OE POU5F1
KO CCND1
KO CREB1
KO POU5F1

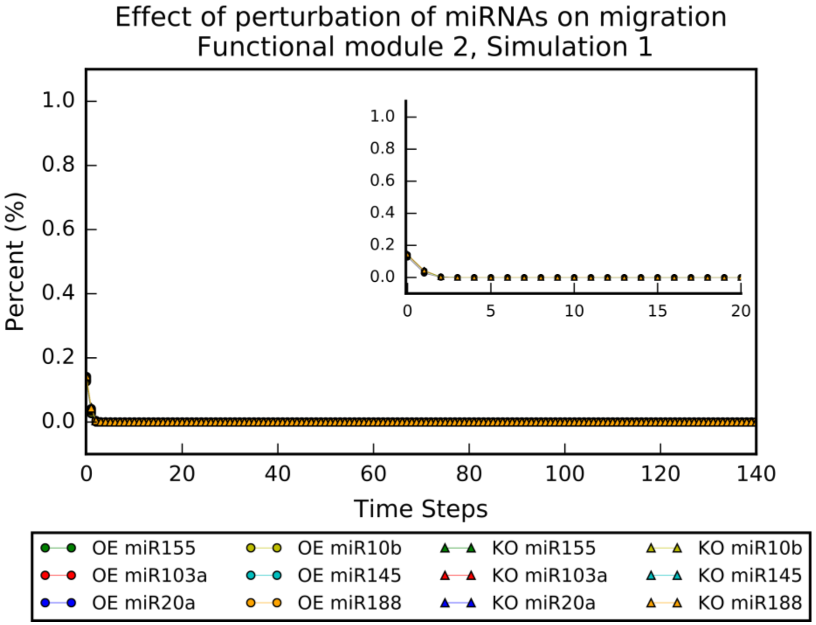
Effect of perturbation of miRNAs on migration
Functional module 2, Simulation 1
Percent (%)
Time Steps
OE miR155
OE miR103a
OE miR20a
OE miR10b
OE miR145
OE miR188
KO miR155
KO miR103a
KO miR20a
KO miR10b
KO miR145
KO miR188

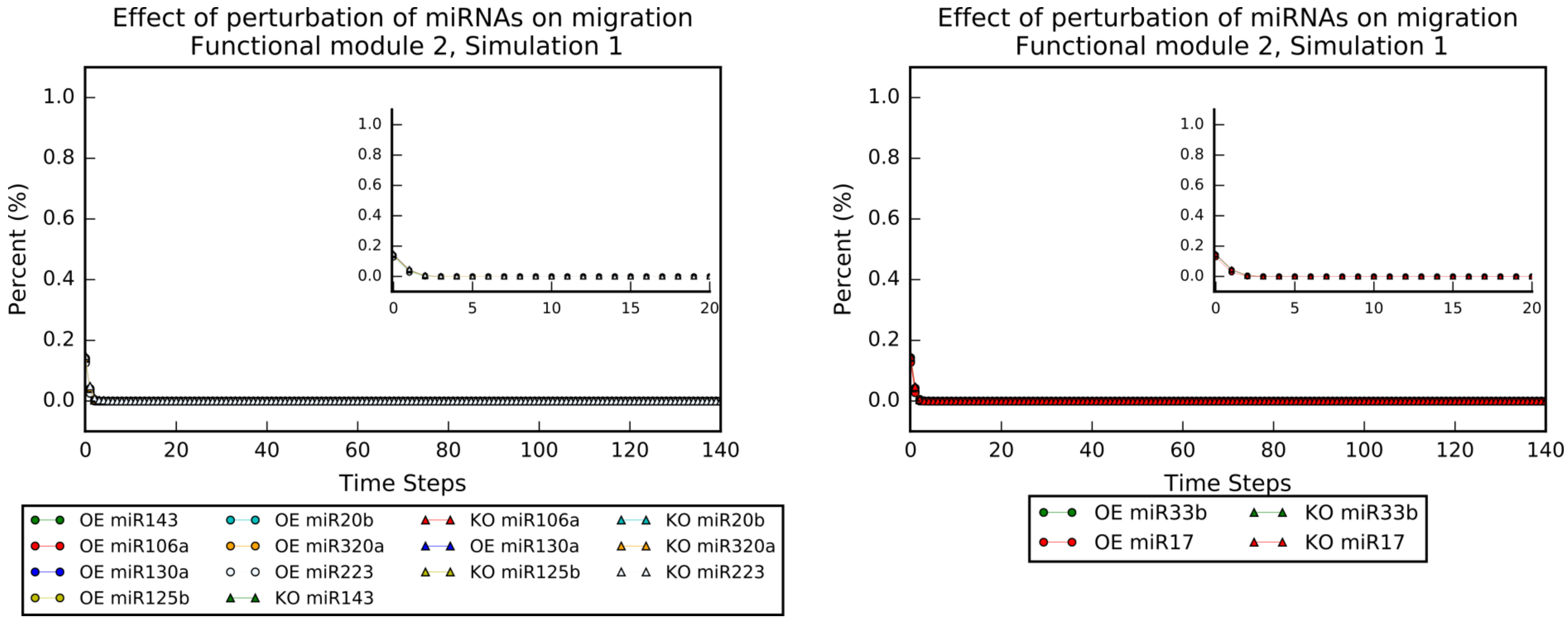

Supplementary Figure S7: Perturbation analysis FM2 Simulation 1

TF activates gene and miRNA expression. Perturbation of each gene did not upregulate migration as expression of all the genes in FM2 is essential for migration.

When each TF was overexpressed (OE), migration was downregulated (0%) as miRNA downregulated TF expression (and in turn inhibited gene expression) or directly repressed gene expression. When each TF was knocked out (KO), migration was downregulated as expression of miRNA repressed gene expression.

When each miRNA was OE, migration was downregulated (0%) as miRNA (by downregulating TF expression) repressed gene expression. When each miRNA was KO, migration was downregulated as expression of miRNAs (regulating other genes in the module) repressed gene expression.

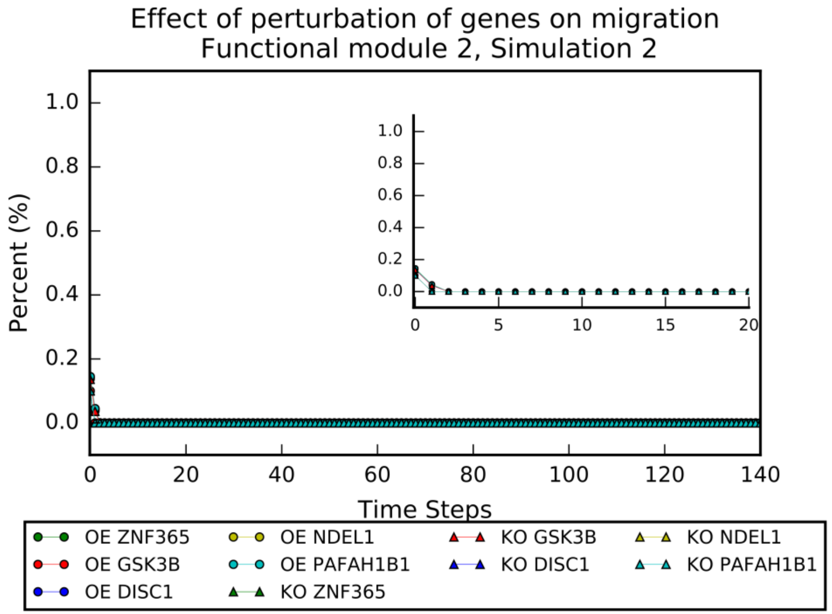
Effect of perturbation of genes on migration
Functional module 2, Simulation 2
Percent (%)
Time Steps
OE ZNF365
OE GSK3B
OE DISC1
OE NDEL1
OE PAFAH1B1
KO ZNF365
KO GSK3B
KO DISC1
KO NDEL1
KO PAFAH1B1

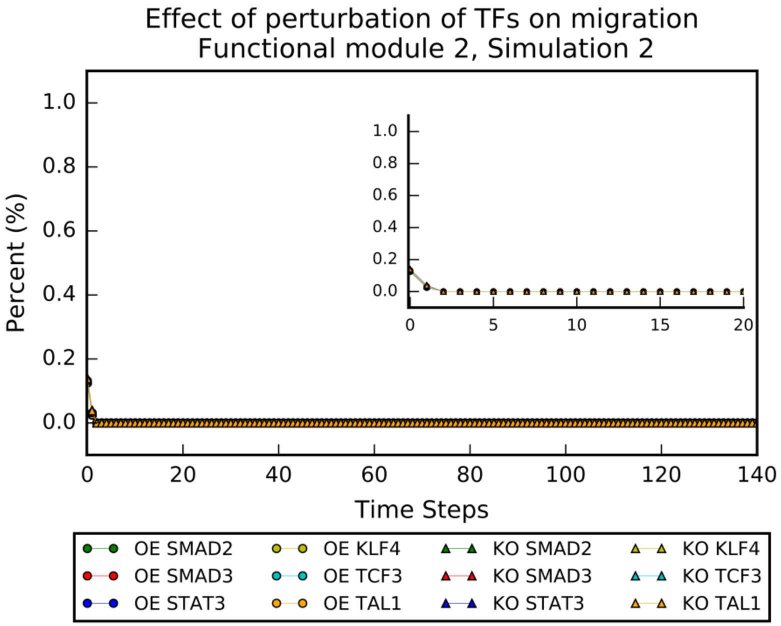
Effect of perturbation of TFs on migration
Functional module 2, Simulation 2
Percent (%)
Time Steps
OE SMAD2
OE SMAD3
OE STAT3
OE KLF4
OE TCF3
OE TAL1
KO SMAD2
KO SMAD3
KO STAT3
KO KLF4
KO TCF3
KO TAL1

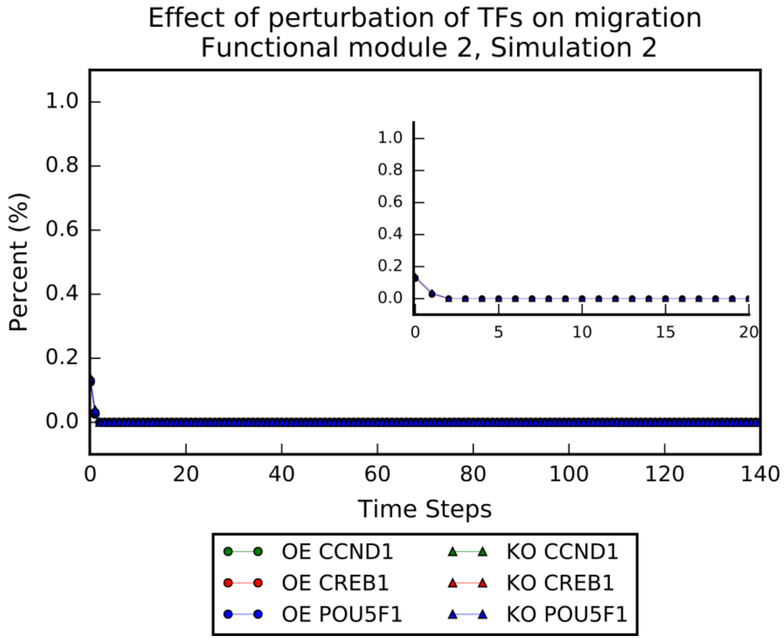
Effect of perturbation of TFs on migration
Functional module 2, Simulation 2
Percent (%)
Time Steps
OE CCND1
OE CREB1
OE POU5F1
KO CCND1
KO CREB1
KO POU5F1

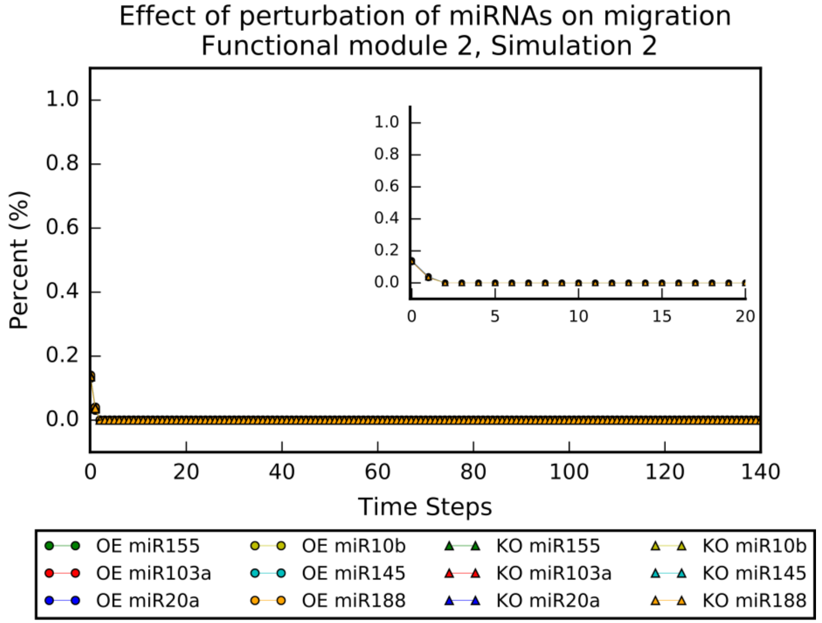
Effect of perturbation of miRNAs on migration
Functional module 2, Simulation 2
Percent (%)
Time Steps
OE miR155
OE miR103a
OE miR20a
OE miR10b
OE miR145
OE miR188
KO miR155
KO miR103a
KO miR20a
KO miR10b
KO miR145
KO miR188

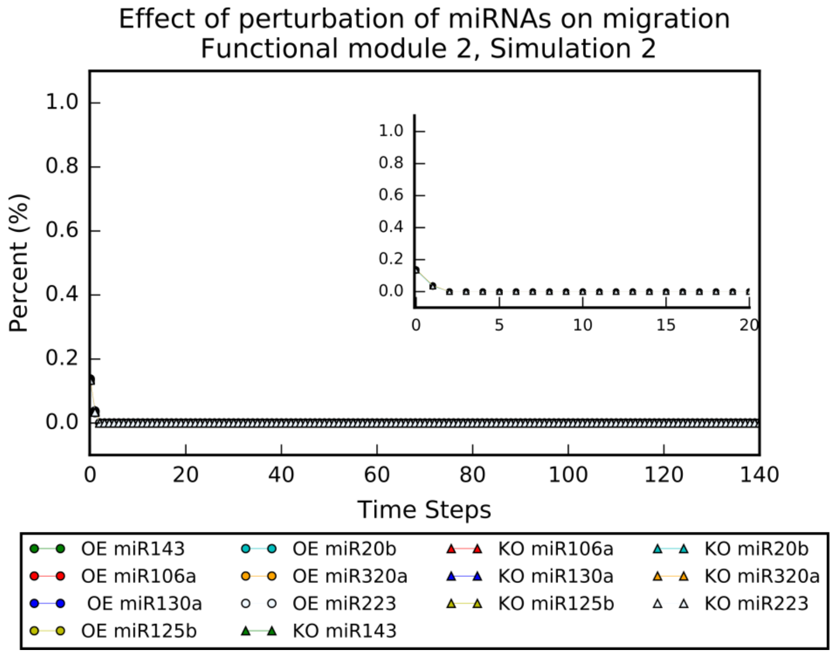

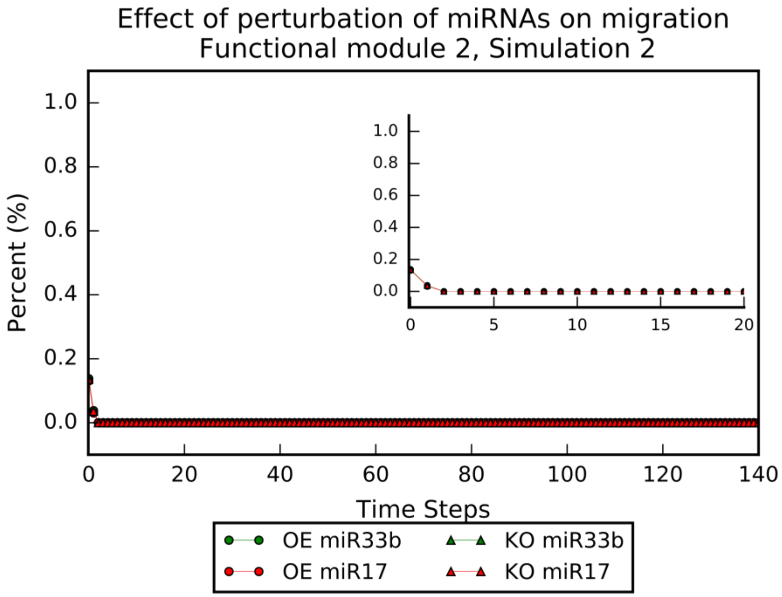

Supplementary Figure S8: Perturbation analysis FM2 Simulation 2

TF repress gene and miRNA expression. Perturbation of each gene did not upregulate migration as expression of all the genes in FM2 is essential for migration.
When each TF was overexpressed (OE), migration was downregulated as miRNA repressed gene expression or TFs which are not repressed by miRNA inhibited gene expression.
When each TF was knocked out (KO), migration was downregulated (0%) as other expressed TFs or miRNAs in the module inhibited gene expression.
When each miRNA was OE, migration was downregulated (0%) as each miRNA repressed gene expression or TFs which are not repressed by miRNAs inhibited gene expression. When each miRNA was KO, migration was downregulated as TFs not repressed by miRNAs or expression of miRNAs (regulating other genes in the module) repressed gene expression.

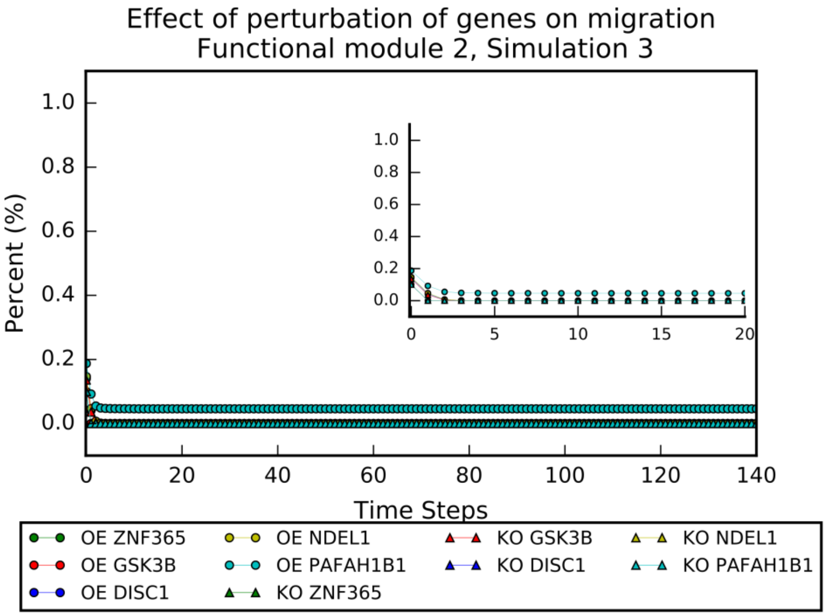
Effect of perturbation of genes on migration
Functional module 2, Simulation 3
Percent (%)
Time Steps
OE ZNF365
OE GSK3B
OE DISC1
OE NDEL1
OE PAFAH1B1
KO ZNF365
KO GSK3B
KO DISC1
KO NDEL1
KO PAFAH1B1

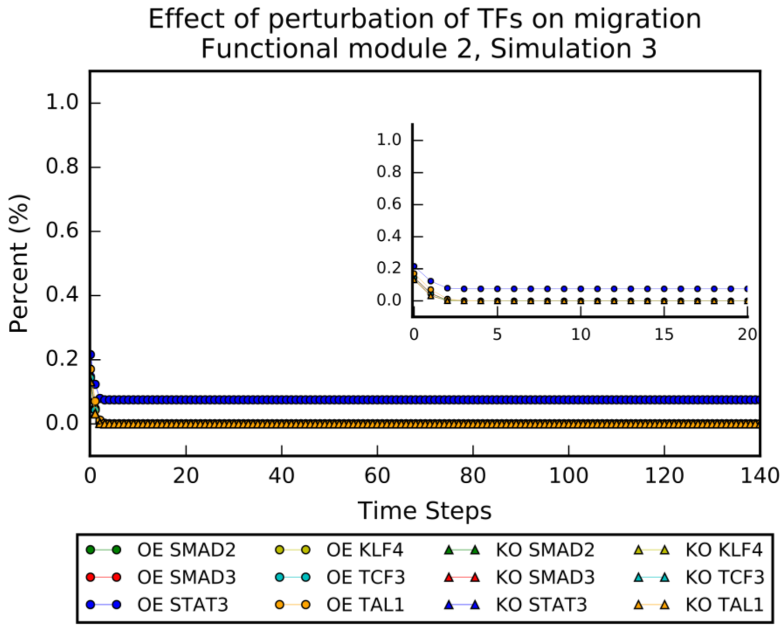
Effect of perturbation of TFs on migration
Functional module 2, Simulation 3
Percent (%)
Time Steps
OE SMAD2
OE SMAD3
OE STAT3
OE KLF4
OE TCF3
OE TAL1
KO SMAD2
KO SMAD3
KO STAT3
KO KLF4
KO TCF3
KO TAL1

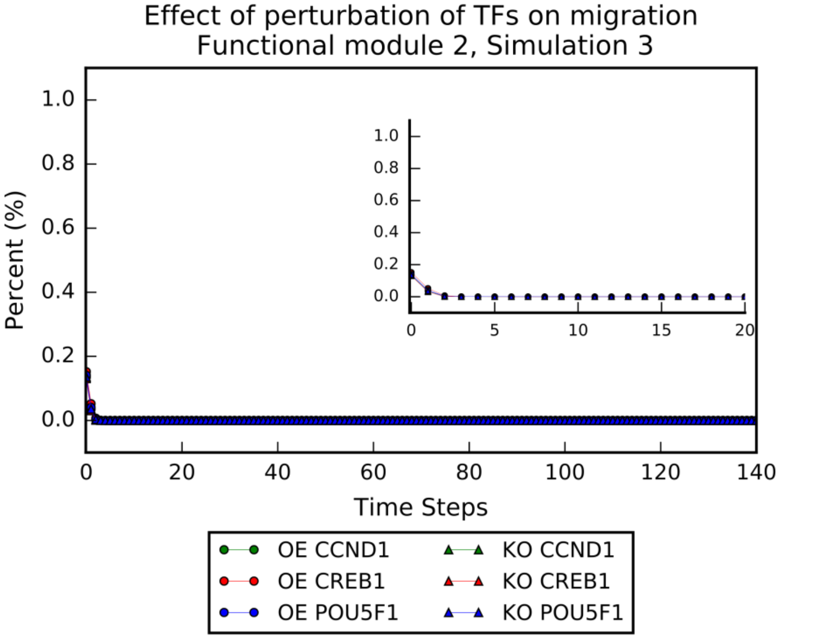
Effect of perturbation of TFs on migration
Functional module 2, Simulation 3
Percent (%)
Time Steps
OE CCND1
OE CREB1
OE POU5F1
KO CCND1
KO CREB1
KO POU5F1

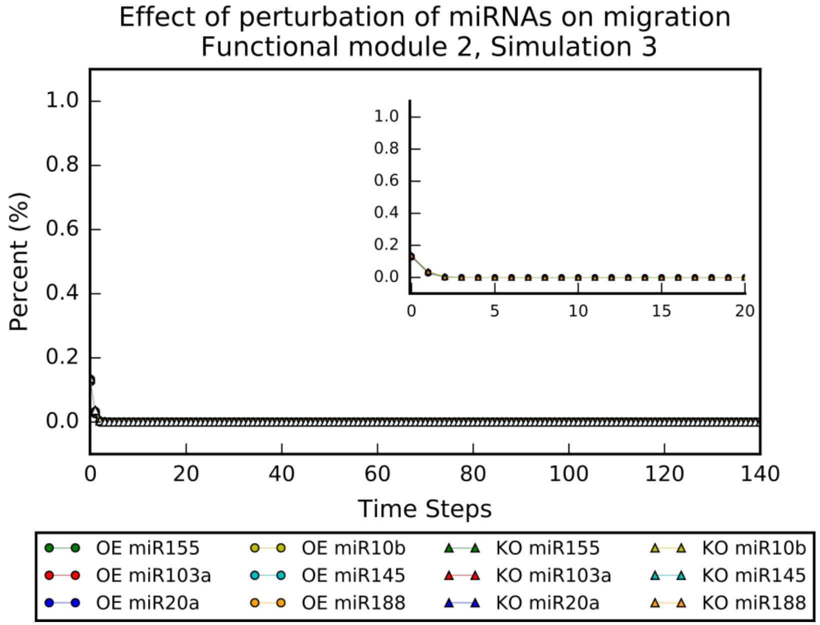
Effect of perturbation of miRNAs on migration
Functional module 2, Simulation 3
Percent (%)
Time Steps
OE miR155
OE miR103a
OE miR20a
OE miR10b
OE miR145
OE miR188
KO miR155
KO miR103a
KO miR20a
KO miR10b
KO miR145
KO miR188

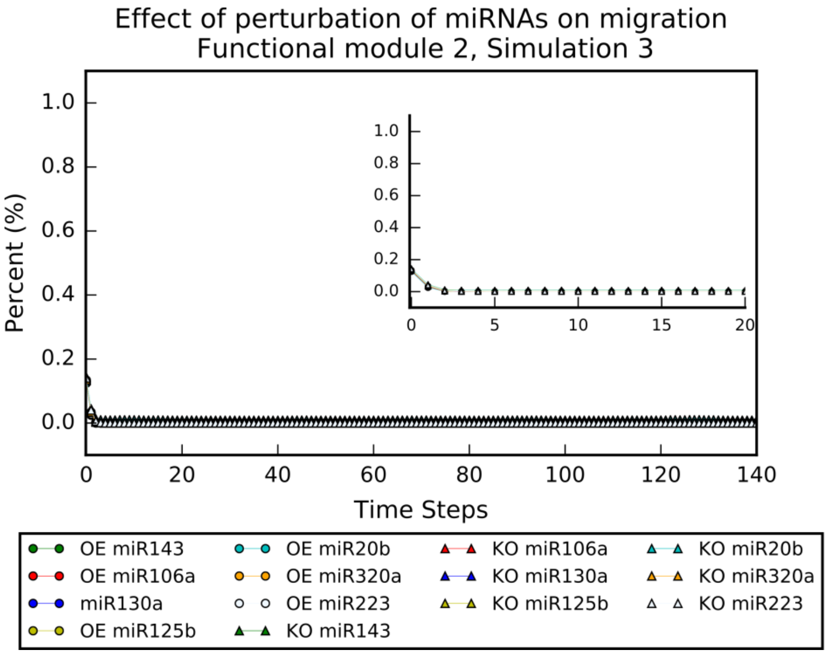

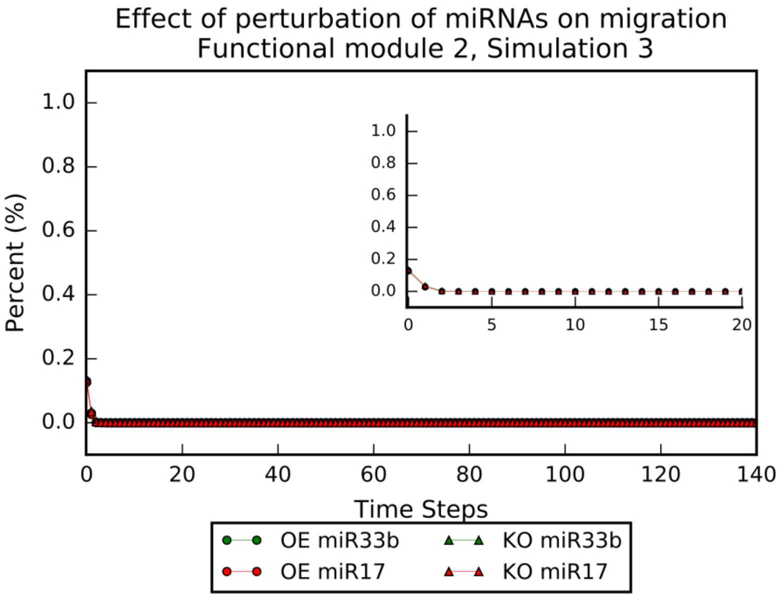

Supplementary Figure S9: Perturbation analysis FM2 Simulation 3

TF activates gene and represses miRNA expression. Perturbation of each gene did not upregulate migration as expression of all the genes in FM2 is essential for migration.
When each TF was overexpressed (OE), migration was not upregulated as miRNA repressed gene expression. When each TF was knocked-out (KO), migration was downregulated as other expressed miRNAs in the module repressed gene expression.
When each miRNA was OE, migration was downregulated (0%) as miRNA repressed gene expression. When each miRNA was KO, migration was downregulated as expression of miRNAs (regulating other genes in the module) repressed gene expression.

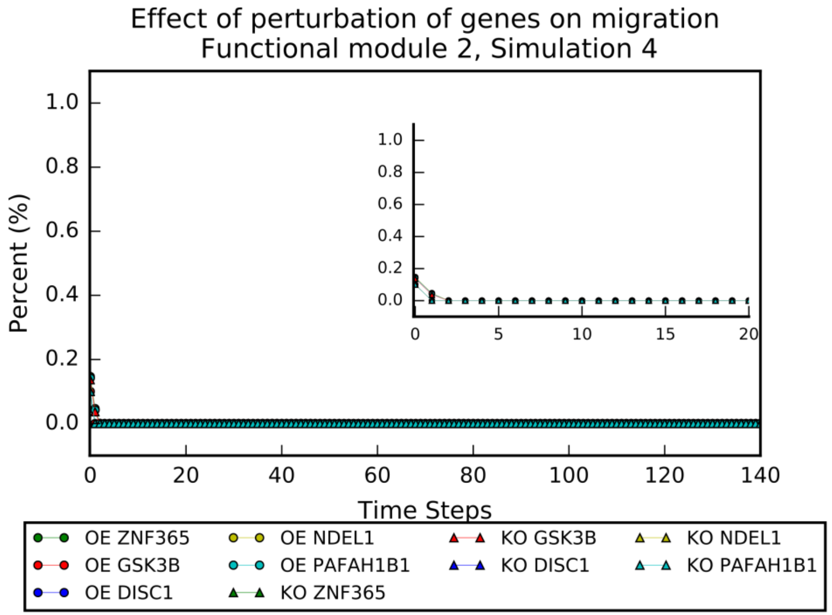
Effect of perturbation of genes on migration
Functional module 2, Simulation 4
Percent (%)
Time Steps
OE ZNF365
OE GSK3B
OE DISC1
OE NDEL1
OE PAFAH1B1
KO ZNF365
KO GSK3B
KO DISC1
KO NDEL1
KO PAFAH1B1

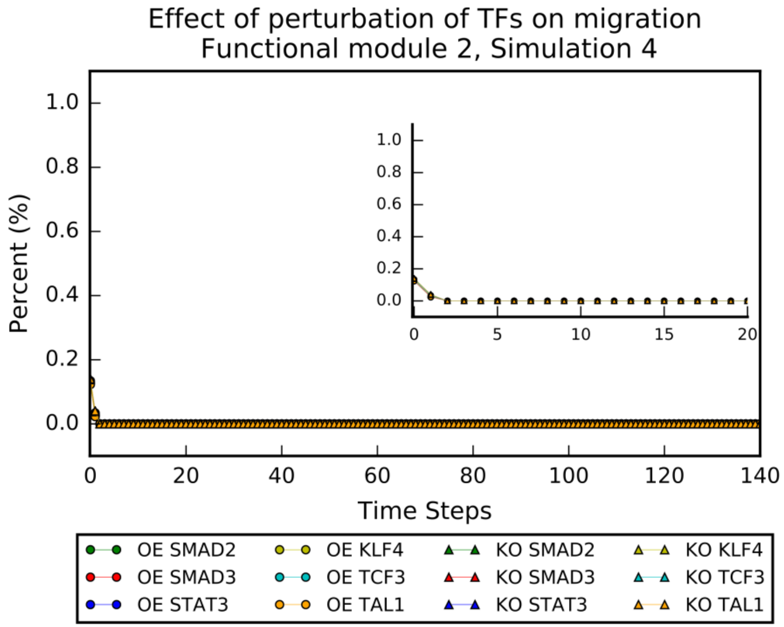
Effect of perturbation of TFs on migration
Functional module 2, Simulation 4
Percent (%)
Time Steps
OE SMAD2
OE SMAD3
OE STAT3
OE KLF4
OE TCF3
OE TAL1
KO SMAD2
KO SMAD3
KO STAT3
KO KLF4
KO TCF3
KO TAL1

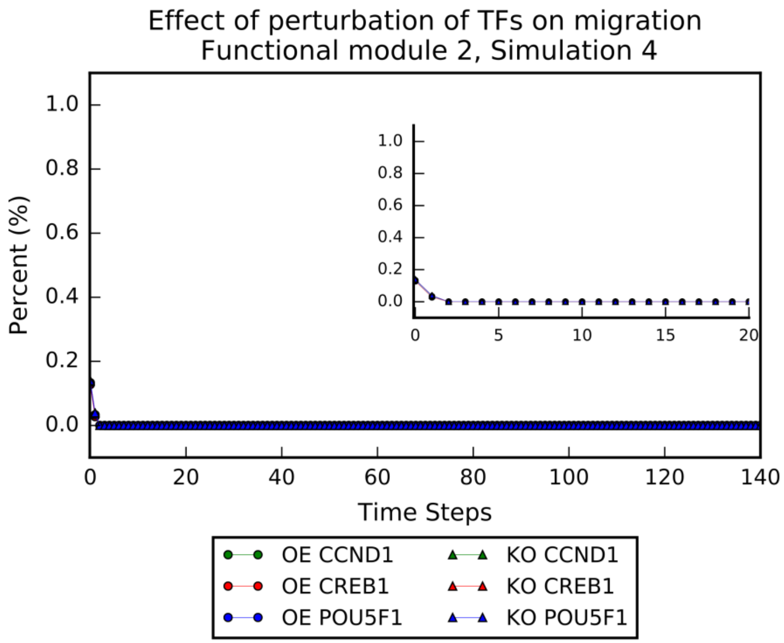
Effect of perturbation of TFs on migration
Functional module 2, Simulation 4
Percent (%)
Time Steps
OE CCND1
OE CREB1
OE POU5F1
KO CCND1
KO CREB1
KO POU5F1

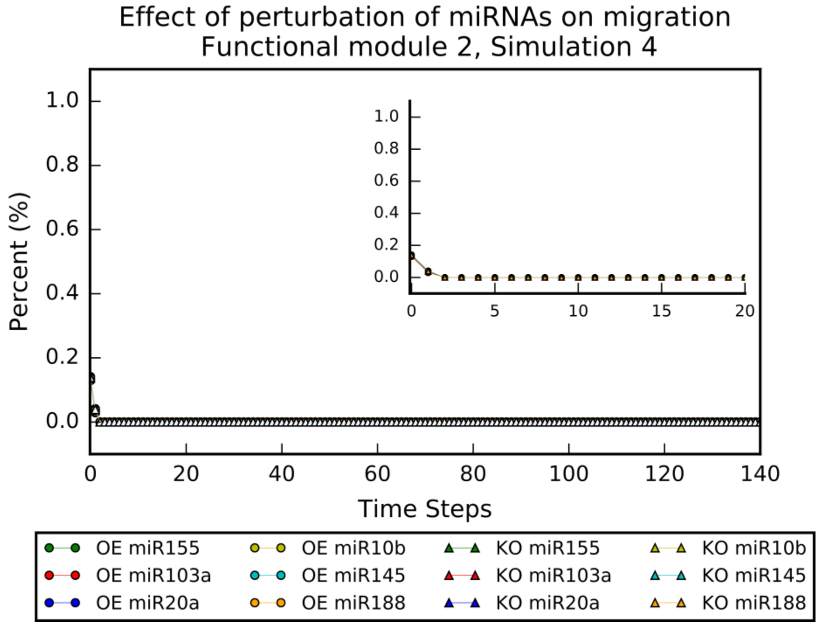
Effect of perturbation of miRNAs on migration
Functional module 2, Simulation 4
Percent (%)
Time Steps
OE miR155
OE miR103a
OE miR20a
OE miR10b
OE miR145
OE miR188
KO miR155
KO miR103a
KO miR20a
KO miR10b
KO miR145
KO miR188

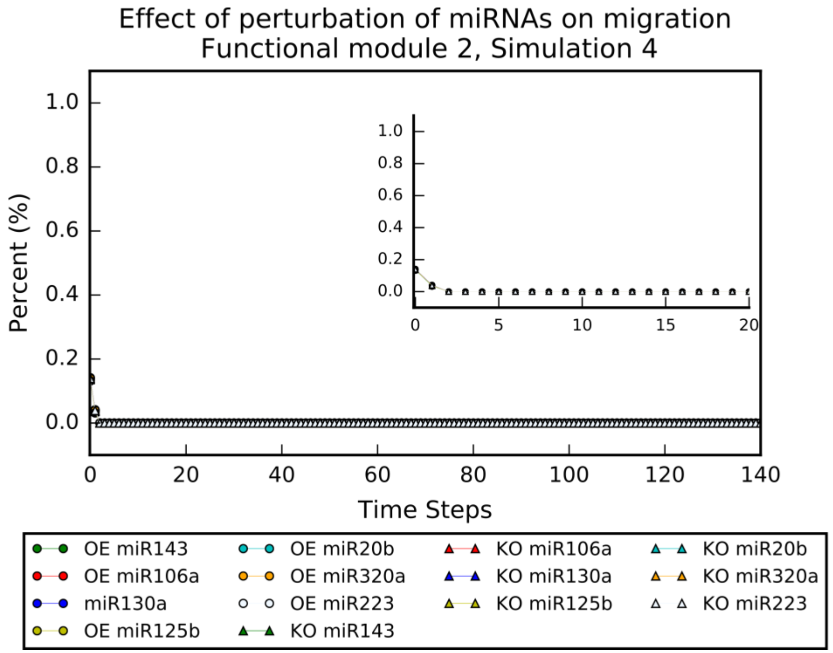

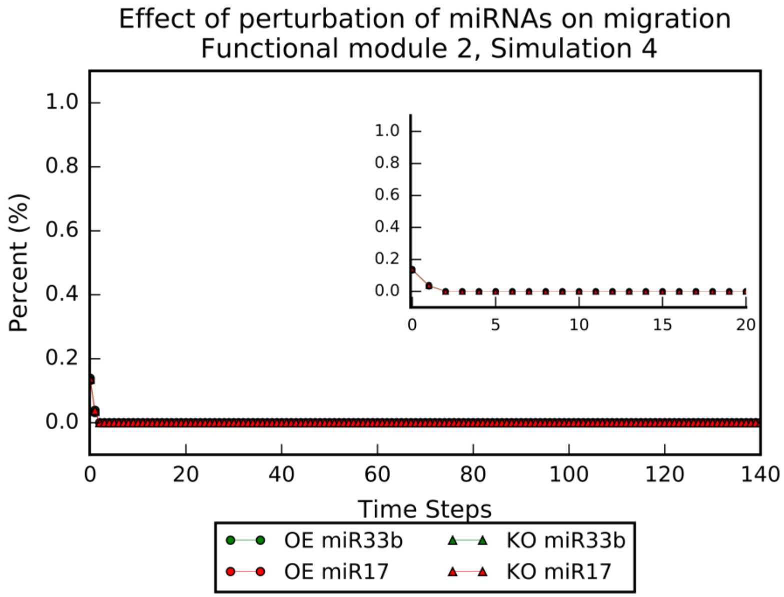

Supplementary Figure S 10: Perturbation analysis FM2 Simulation 4

TF represses gene and activates miRNA expression. Perturbation of each gene did not upregulate migration as expression of all genes in FM2 is essential for migration.

When each TF was overexpressed (OE), migration was not upregulated as miRNA repressed gene expression. When each TF was knocked-out (KO), migration was downregulated as other expressed miRNA in the module represses gene expression.

When each miRNA was OE, migration was downregulated (0%) as miRNA represses gene expression. When each miRNA was KO, migration was downregulated as expression of miRNAs (regulating other genes in the module) repressed gene expression.

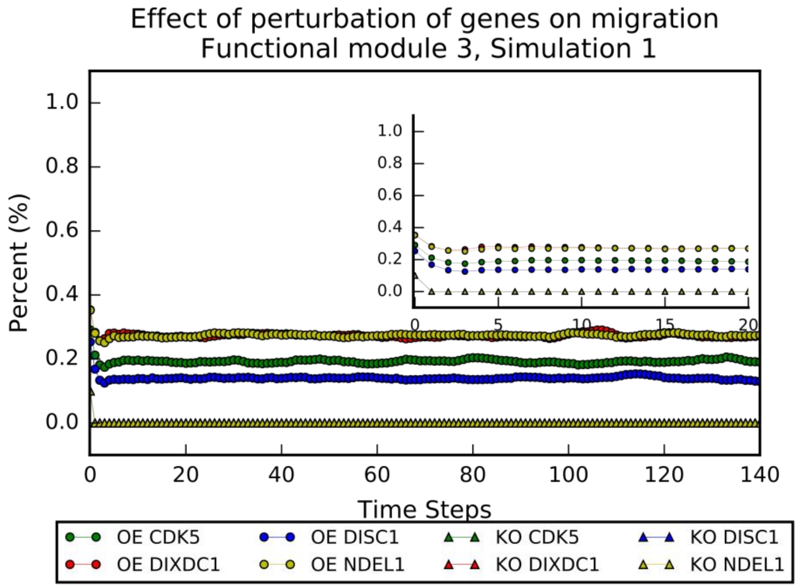
Effect of perturbation of genes on migration
Functional module 3, Simulation 1
Percent (%)
Time Steps
OE CDK5
OE DIXDC1
OE DISC1
OE NDEL1
KO CDK5
KO DIXDC1
KO DISC1
KO NDEL1

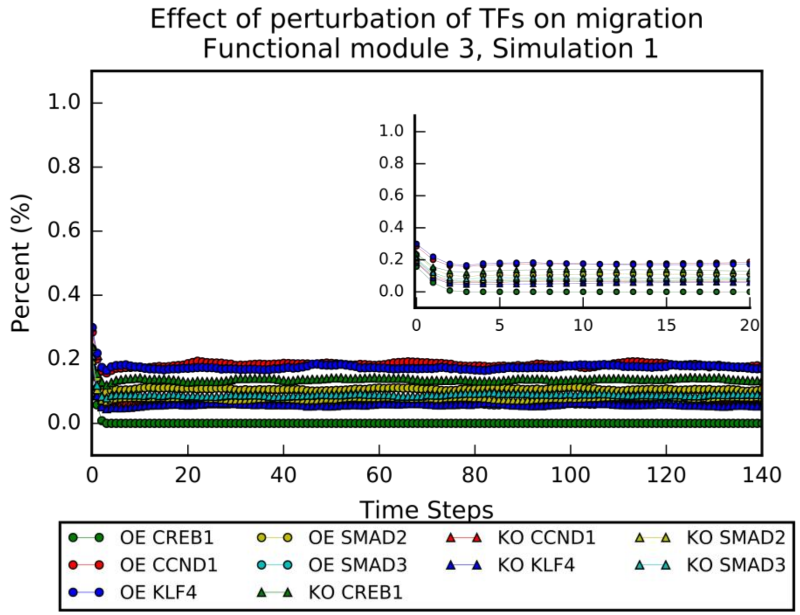
Effect of perturbation of TFs on migration
Functional module 3, Simulation 1
Percent (%)
Time Steps
OE CREB1
OE CCND1
OE KLF4
OE SMAD2
OE SMAD3
KO CREB1
KO CCND1
KO KLF4
KO SMAD2
KO SMAD3

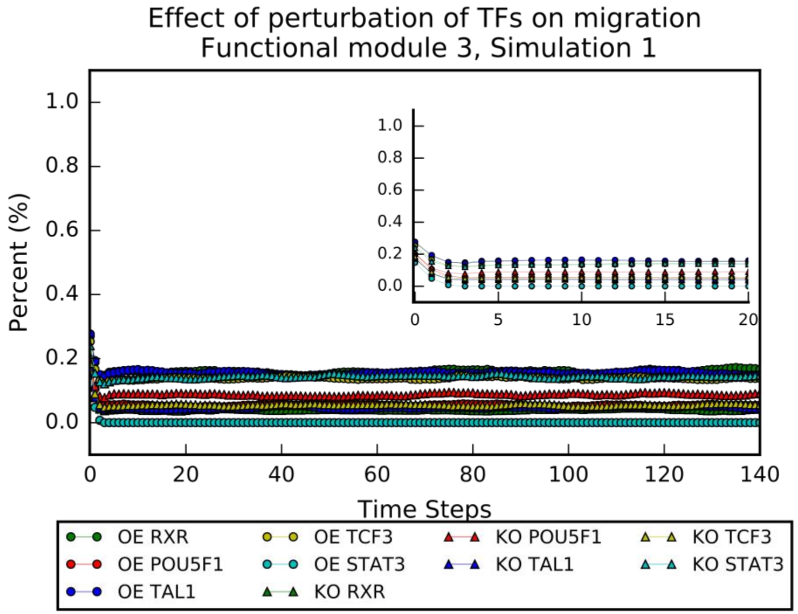
Effect of perturbation of TFs on migration
Functional module 3, Simulation 1
Percent (%)
Time Steps
OE RXR
OE POU5F1
OE TAL1
OE TCF3
OE STAT3
KO RXR
KO POU5F1
KO TAL1
KO TCF3
KO STAT3

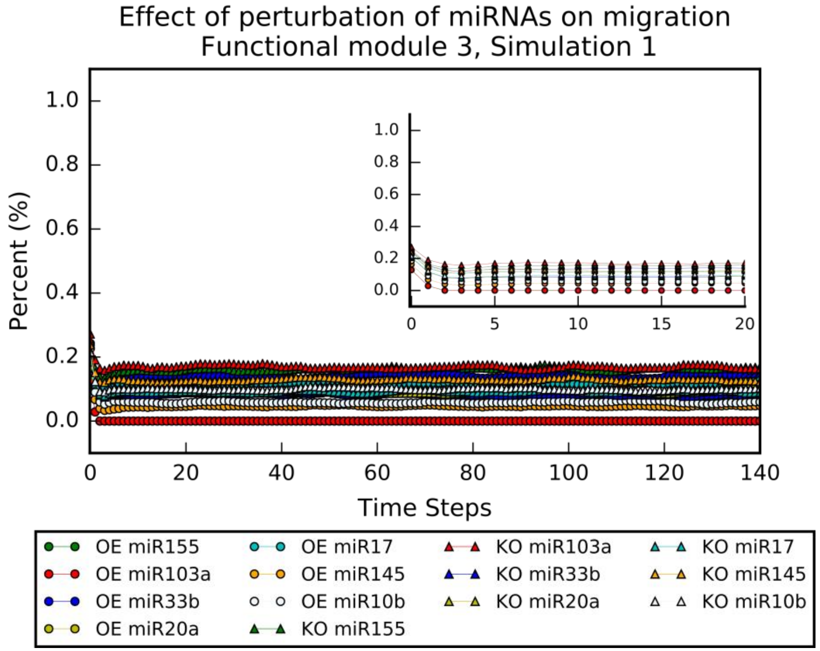
Effect of perturbation of miRNAs on migration
Functional module 3, Simulation 1
Percent (%)
Time Steps
OE miR155
OE miR103a
OE miR33b
OE miR20a
OE miR17
OE miR145
OE miR10b
KO miR155
KO miR103a
KO miR33b
KO miR20a
KO miR17
KO miR145
KO miR10b

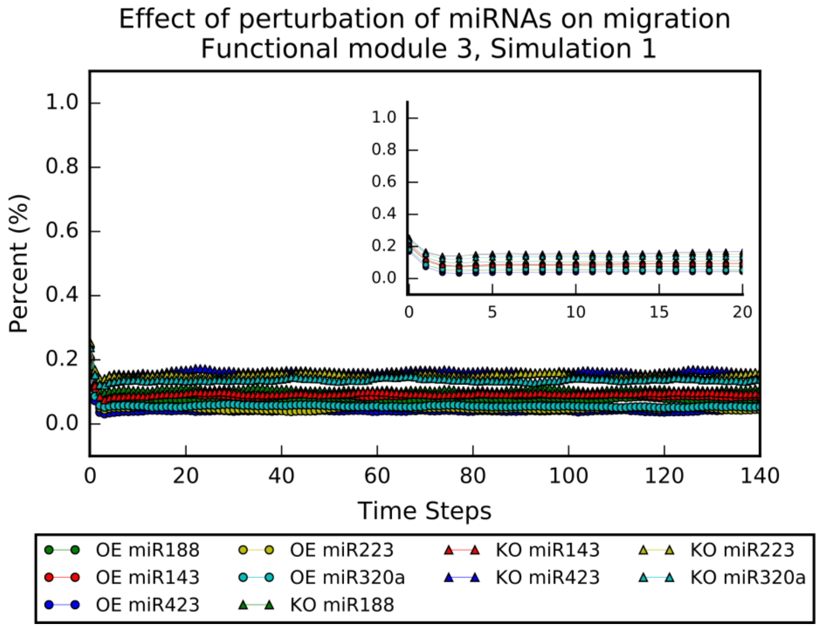

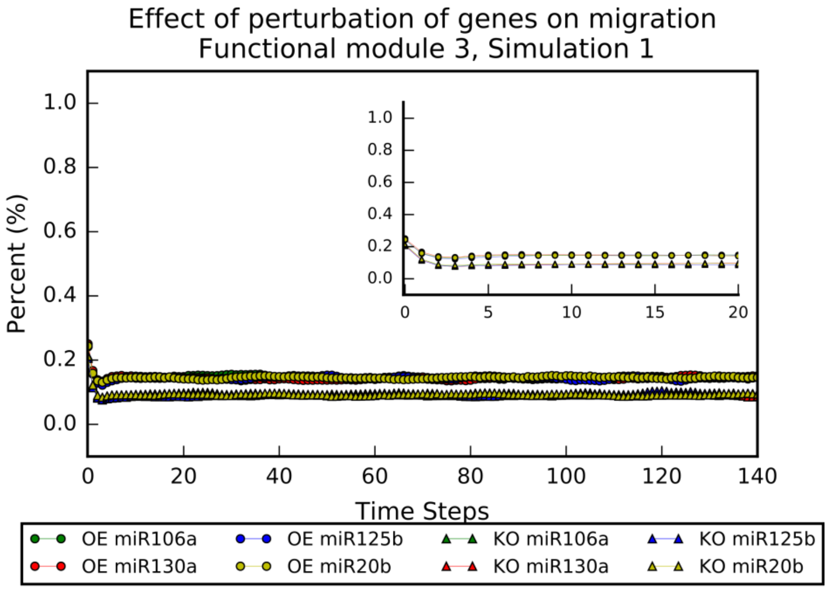

Supplementary Figure S11: Perturbation analysis FM3 Simulation 1

TF activates gene and miRNA expression. Perturbation of each gene did not upregulate migration as expression of all genes in FM3 is essential for migration. Over expression (OE) of DIXDC1 gene, regulated migration between 24%-25%, as DIXDC1 phosphorylated by CDK5 forms complex with DISC1 and NDEL1 and regulates radial migration.

Perturbation of each TF did not upregulate migration, as when TF was OE, miRNAs represses gene expression. When TF was knocked-out (KO), gene expression gets downregulated or other expressed miRNAs (in the module) inhibits gene expression.

Perturbation of each miRNA did not upregulate migration, as when miRNA was OE, TFs upregulating gene expression gets repressed. Similarly, when each miRNA was KO, migration was not upregulated as other expressed miRNAs in the module represses TF expression. OE of each miRNA (except miR103a and miR155) did not downregulate migration, as TF that was not regulated (not repressed) by this over expressed miRNA regulates migration. However, when miR155 or miR103a was OE, migration was downregulated (0%) as these miRNAs repress CDK5 and NDEL1 expression.

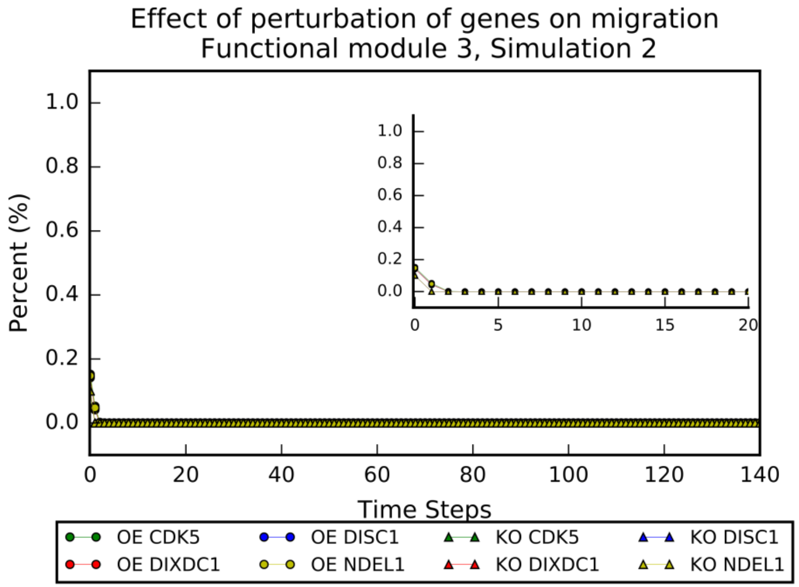

Effect of perturbation of genes on migration
Functional module 3, Simulation 2
Percent (%)
Time Steps
OE CDK5
OE DIXDC1
OE DISC1
OE NDEL1
KO CDK5
KO DIXDC1
KO DISC1
KO NDEL1

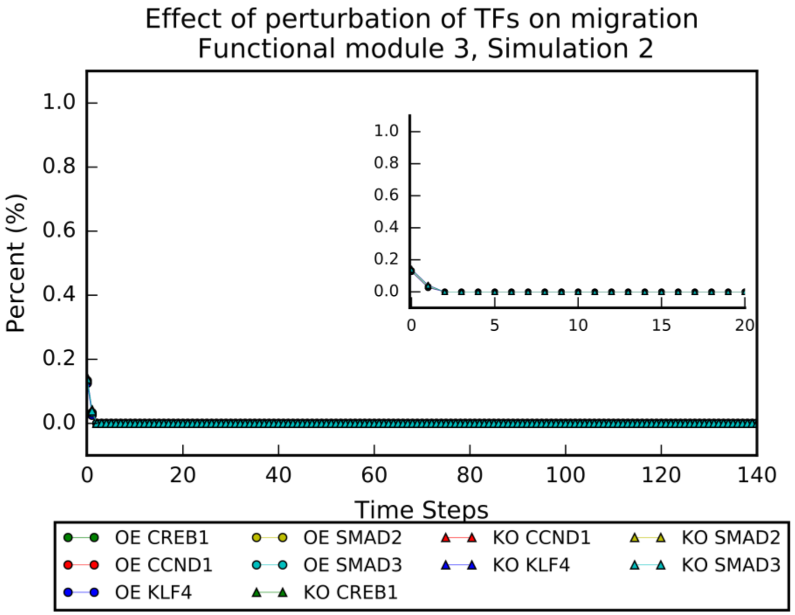

Effect of perturbation of TFs on migration
Functional module 3, Simulation 2
Percent (%)
Time Steps
OE CREB1
OE CCND1
OE KLF4
OE SMAD2
OE SMAD3
KO CREB1
KO CCND1
KO KLF4
KO SMAD2
KO SMAD3

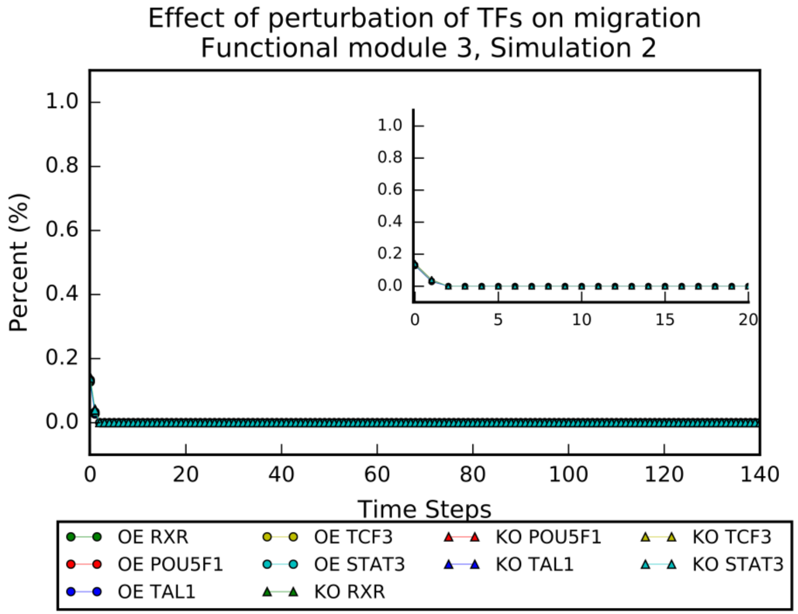

Effect of perturbation of TFs on migration
Functional module 3, Simulation 2
Percent (%)
Time Steps
OE RXR
OE POU5F1
OE TAL1
OE TCF3
OE STAT3
KO RXR
KO POU5F1
KO TAL1
KO TCF3
KO STAT3

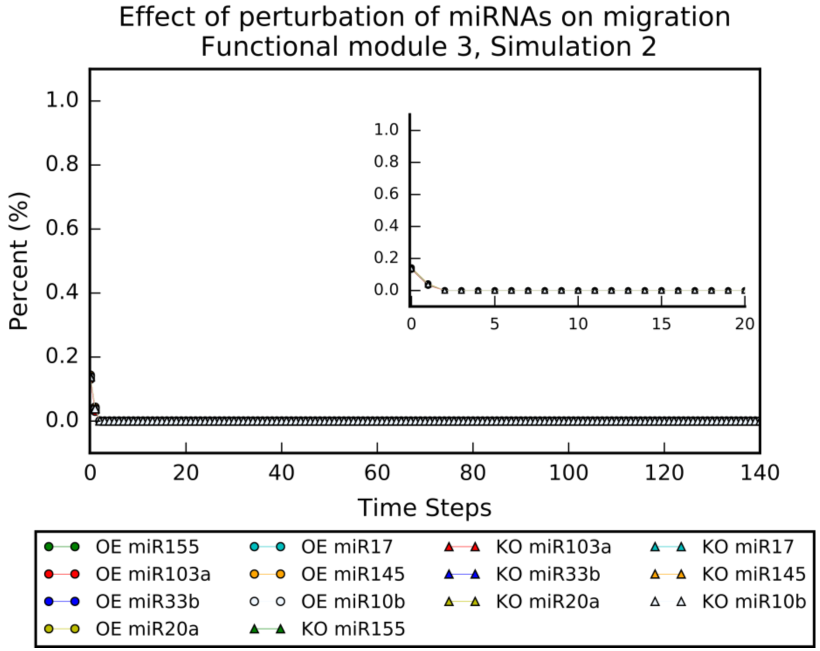

Effect of perturbation of miRNAs on migration
Functional module 3, Simulation 2
Percent (%)
Time Steps
OE miR155
OE miR103a
OE miR33b
OE miR20a
OE miR17
OE miR145
OE miR10b
KO miR155
KO miR103a
KO miR33b
KO miR20a
KO miR17
KO miR145
KO miR10b

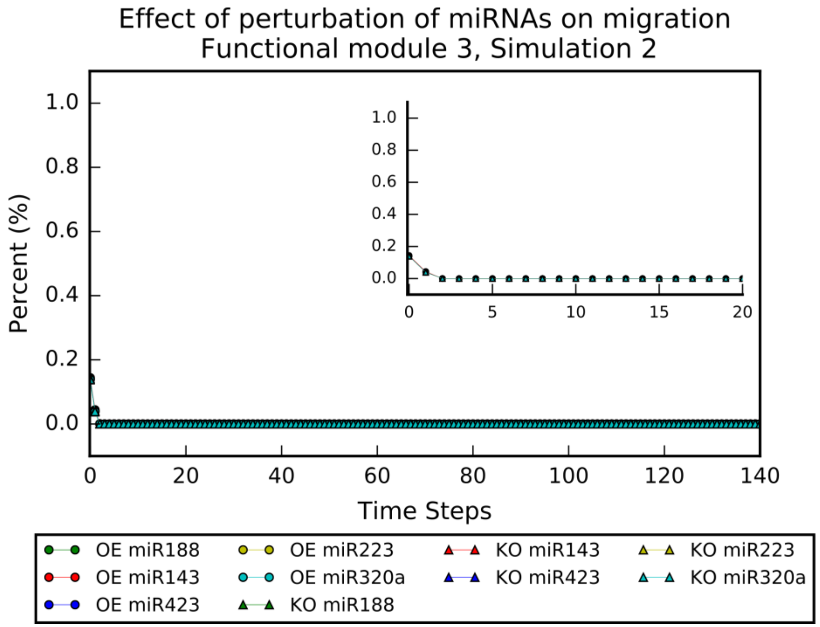

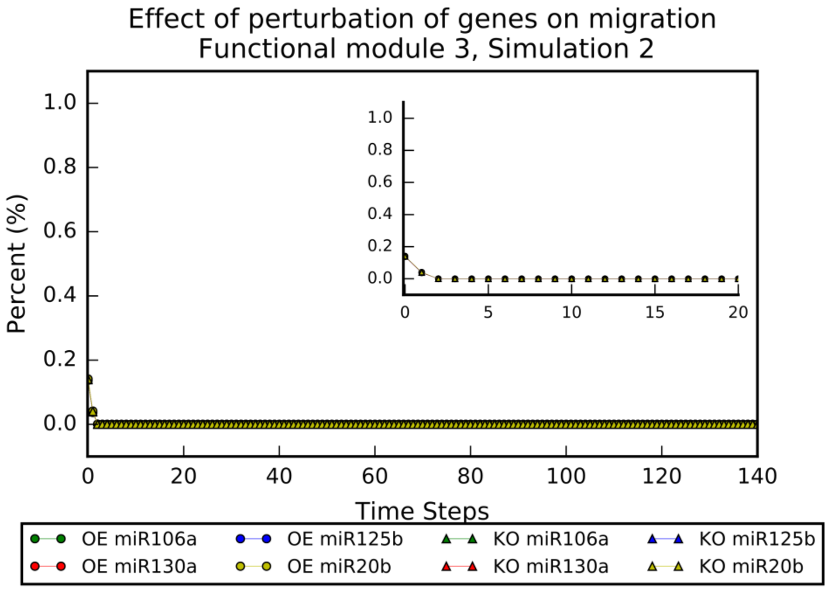

Supplementary Figure S12: Perturbation analysis FM3 Simulation 2

TF inhibits gene and miRNA expression. Perturbation of each gene did not upregulate migration as expression of all genes in FM3 is essential for migration.

Perturbation of each TF did not upregulate migration, as when TF was overexpressed (OE), miRNA or TF represses gene expression. When TF was knocked-out (KO), other expressed TFs or miRNAs in the functional module inhibits gene expression.

Perturbation of each miRNA did not upregulate migration, as when miRNA was OE, it inhibits gene expression. Similarly, when each miRNA was KO, migration was not upregulated as TF either activates miRNA expression or inhibits gene expression

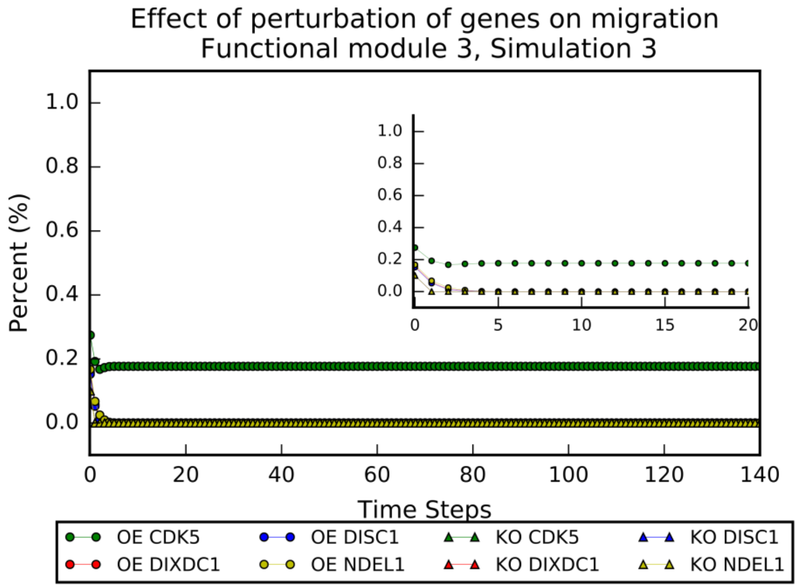
Effect of perturbation of genes on migration
Functional module 3, Simulation 3
Percent (%)
Time Steps
OE CDK5
OE DIXDC1
OE DISC1
OE NDEL1
KO CDK5
KO DIXDC1
KO DISC1
KO NDEL1

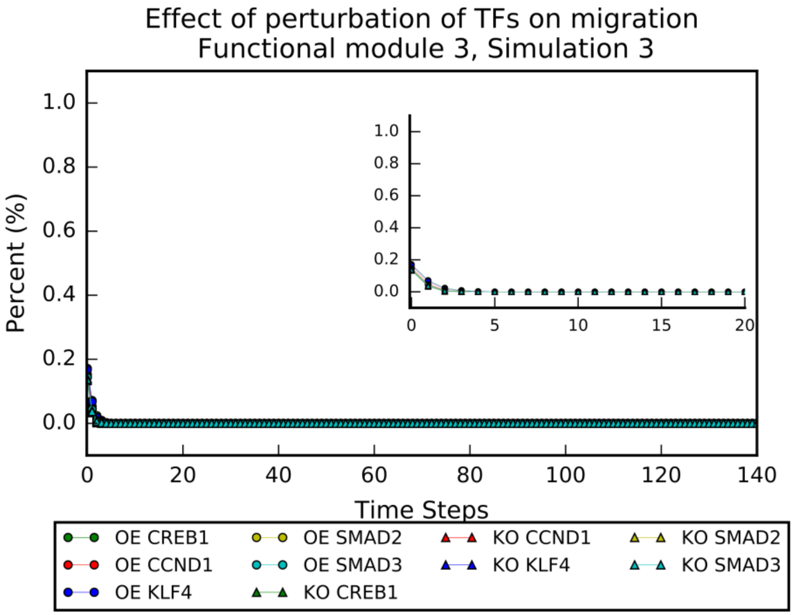
Effect of perturbation of TFs on migration
Functional module 3, Simulation 3
Percent (%)
Time Steps
OE CREB1
OE CCND1
OE KLF4
OE SMAD2
OE SMAD3
KO CREB1
KO CCND1
KO KLF4
KO SMAD2
KO SMAD3

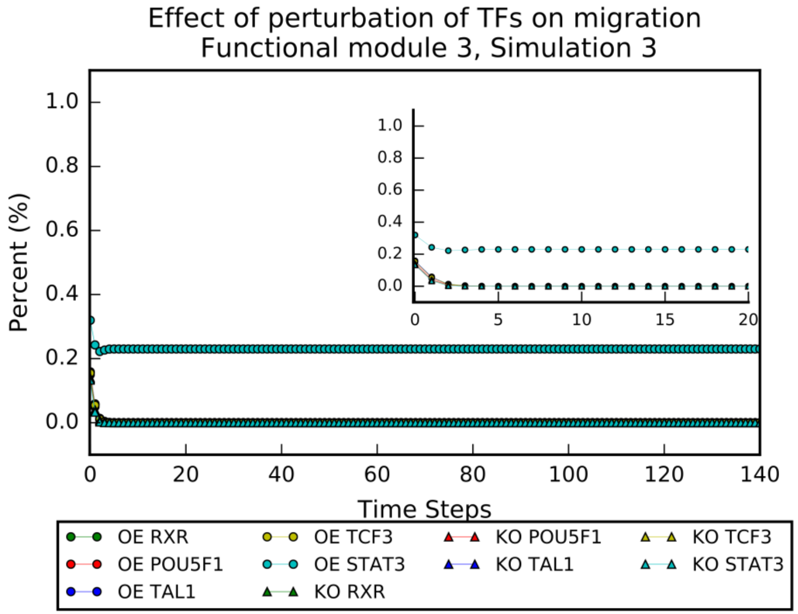
Effect of perturbation of TFs on migration
Functional module 3, Simulation 3
Percent (%)
Time Steps
OE RXR
OE POU5F1
OE TAL1
OE TCF3
OE STAT3
KO RXR
KO POU5F1
KO TAL1
KO TCF3
KO STAT3

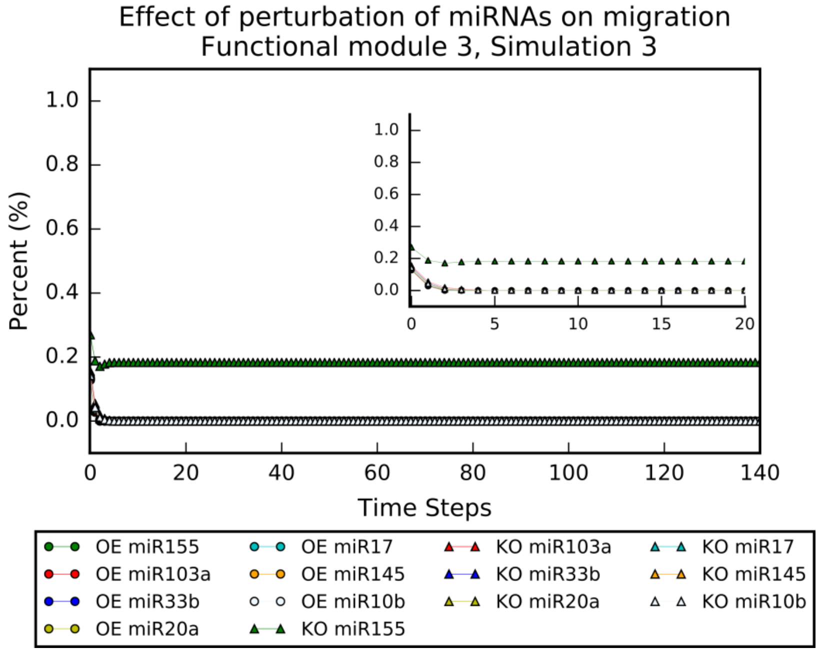
Effect of perturbation of miRNAs on migration
Functional module 3, Simulation 3
Percent (%)
Time Steps
OE miR155
OE miR103a
OE miR33b
OE miR20a
OE miR17
OE miR145
OE miR10b
KO miR155
KO miR103a
KO miR33b
KO miR20a
KO miR17
KO miR145
KO miR10b

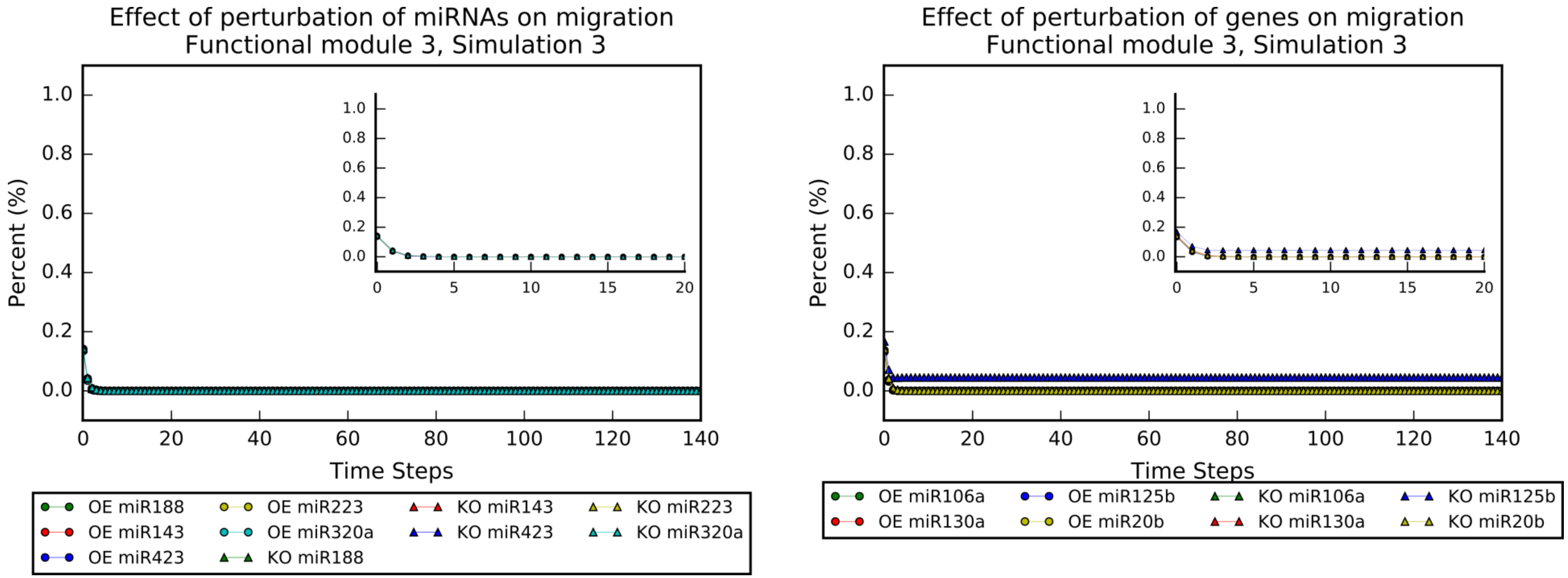

Supplementary Figure S13: Perturbation analysis FM3 Simulation 3

TF inhibits miRNA and activates gene expression. Perturbation of each gene did not upregulate migration as expression of all genes in FM3 is essential for migration. When CDK5 gene was overexpressed (OE), migration was regulated (17%), as CDK5 phosphorylates DIXDC1 and phosphorylated DIXDC1 forms complex with DISC1 and NDEL1 and regulates migration.
Perturbation of each TF did not upregulate migration, as when TF was OE, miRNAs represses gene expression and when TF was knocked-out (KO), gene expression gets downregulated or miRNA inhibits gene expression. When STAT3 was OE, it downregulates miR155 expression and activates CDK5 gene expression, thereby regulating migration up to 23%.
Perturbation of each miRNA did not upregulate migration, as when miRNA was OE, TFs upregulating gene expression gets repressed. Similarly, when each miRNA was KO, TF/gene was repressed by other expressed miRNAs in the functional module. When miR155 was KO, migration was regulated (18%), as CDK5 gene (directly repressed by miR155) gets expressed. As other TFs and miRNAs affect CDK5 gene expression, migration was not upregulated

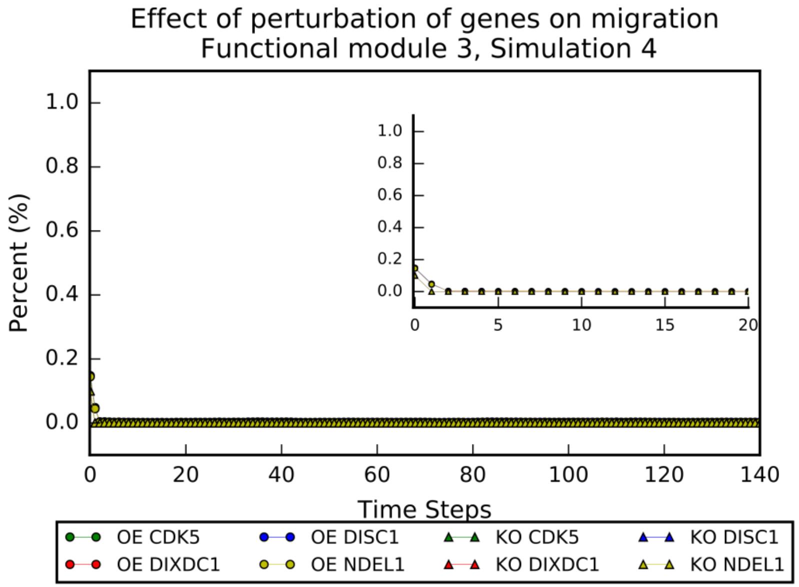
Effect of perturbation of genes on migration
Functional module 3, Simulation 4
Percent (%)
Time Steps
OE CDK5
OE DIXDC1
OE DISC1
OE NDEL1
KO CDK5
KO DIXDC1
KO DISC1
KO NDEL1

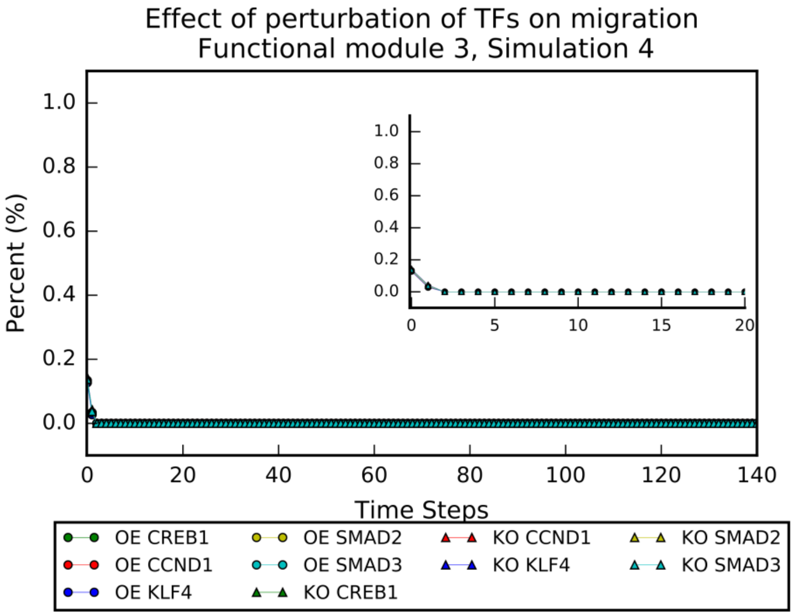
Effect of perturbation of TFs on migration
Functional module 3, Simulation 4
Percent (%)
Time Steps
OE CREB1
OE CCND1
OE KLF4
OE SMAD2
OE SMAD3
KO CREB1
KO CCND1
KO KLF4
KO SMAD2
KO SMAD3

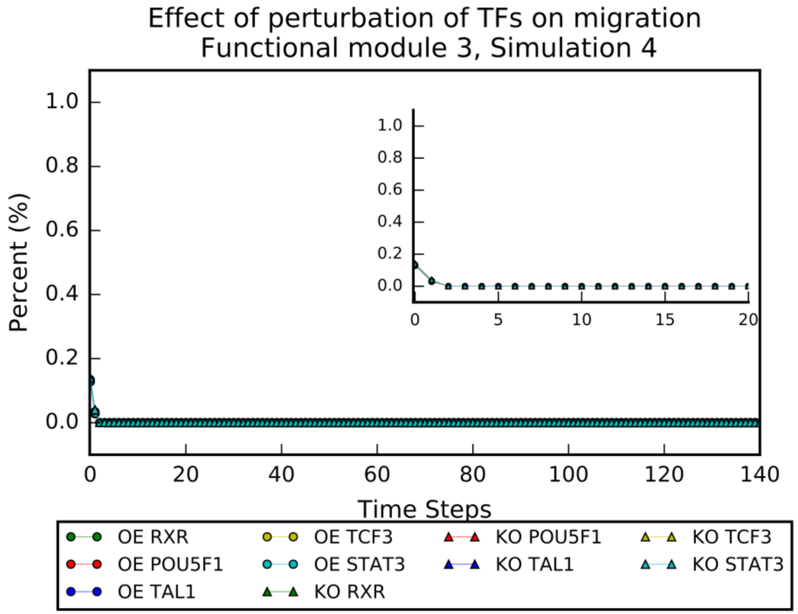
Effect of perturbation of TFs on migration
Functional module 3, Simulation 4
Percent (%)
Time Steps
OE RXR
OE POU5F1
OE TAL1
OE TCF3
OE STAT3
KO RXR
KO POU5F1
KO TAL1
KO TCF3
KO STAT3

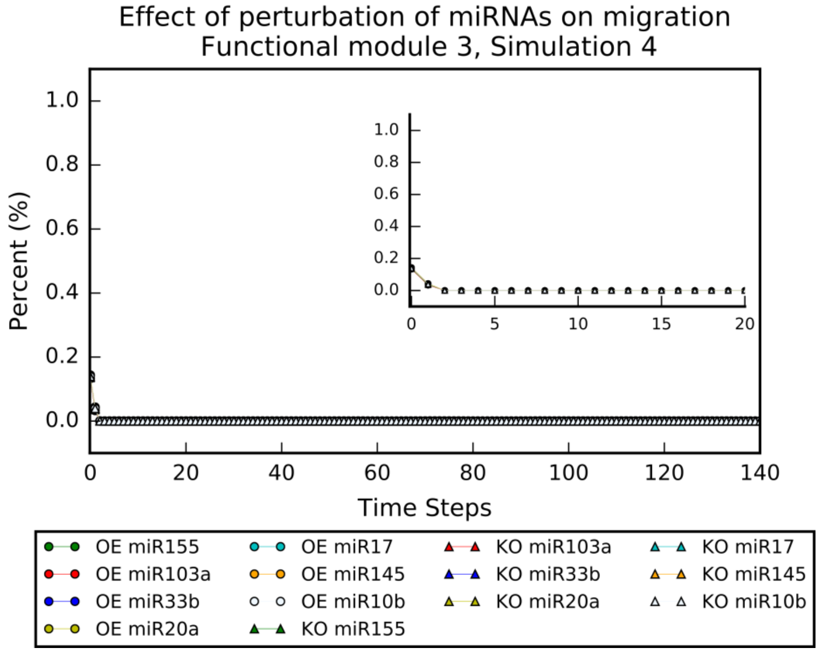
Effect of perturbation of miRNAs on migration
Functional module 3, Simulation 4
Percent (%)
Time Steps
OE miR155
OE miR103a
OE miR33b
OE miR20a
OE miR17
OE miR145
OE miR10b
KO miR155
KO miR103a
KO miR33b
KO miR20a
KO miR17
KO miR145
KO miR10b

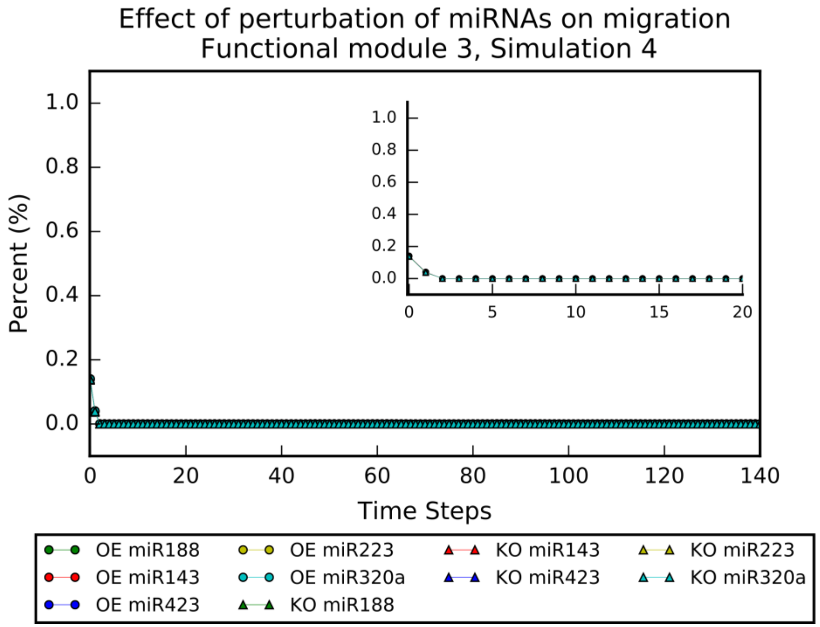

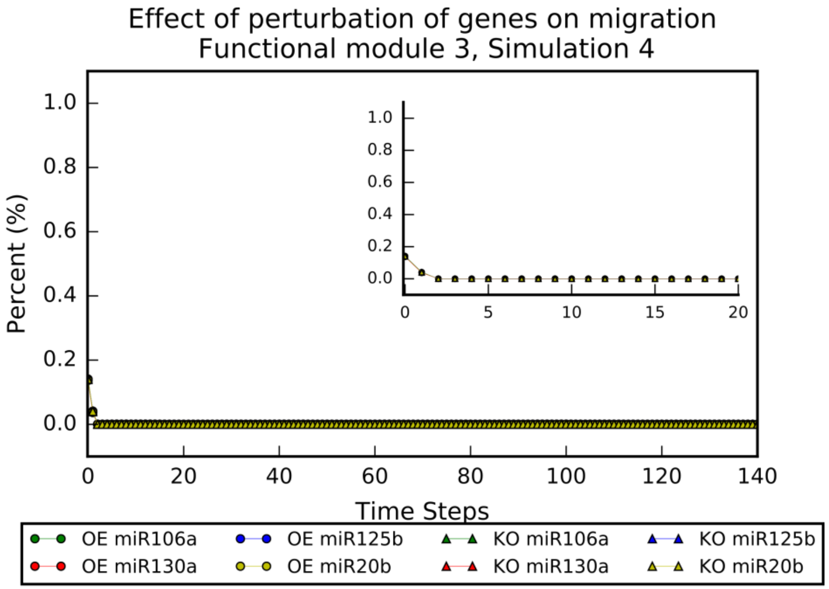

Supplementary Figure S14: Perturbation analysis FM3 Simulation 4

TF inhibits gene and activates miRNA expression. Perturbation of each gene did not upregulate migration as expression of all genes in FM3 is essential for migration.
Perturbation of each TF did not upregulate migration, as when TF was overexpressed (OE), miRNA or TF represses gene expression. When TF was knocked-out (KO), other expressed TFs or miRNAs in the functional module inhibit gene expression.
Perturbation of each miRNA did not upregulate migration, as when miRNA was OE, it inhibits gene expression. Similarly, when each miRNA was KO, migration was not upregulated as TF either activates miRNA expression or inhibits gene expression

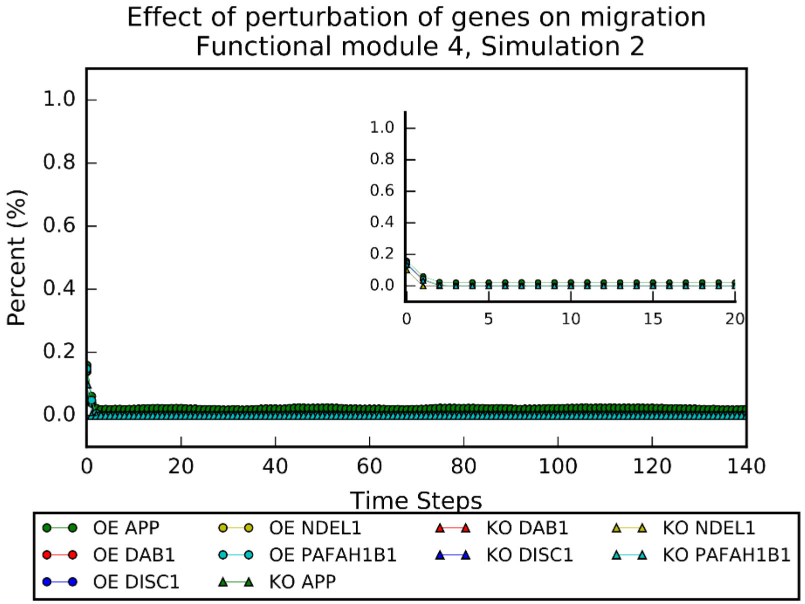
Effect of perturbation of genes on migration
Functional module 4, Simulation 2
Percent (%)
Time Steps
OE APP
OE DAB1
OE DISC1
OE NDEL1
OE PAFAH1B1
KO APP
KO DAB1
KO DISC1
KO NDEL1
KO PAFAH1B1

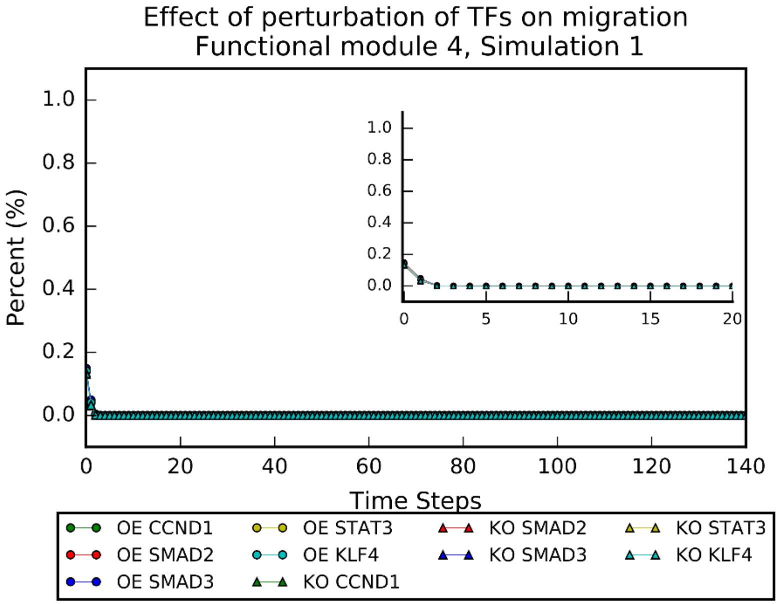
Effect of perturbation of TFs on migration
Functional module 4, Simulation 1
Percent (%)
Time Steps
OE CCND1
OE SMAD2
OE SMAD3
OE STAT3
OE KLF4
KO CCND1
KO SMAD2
KO SMAD3
KO STAT3
KO KLF4

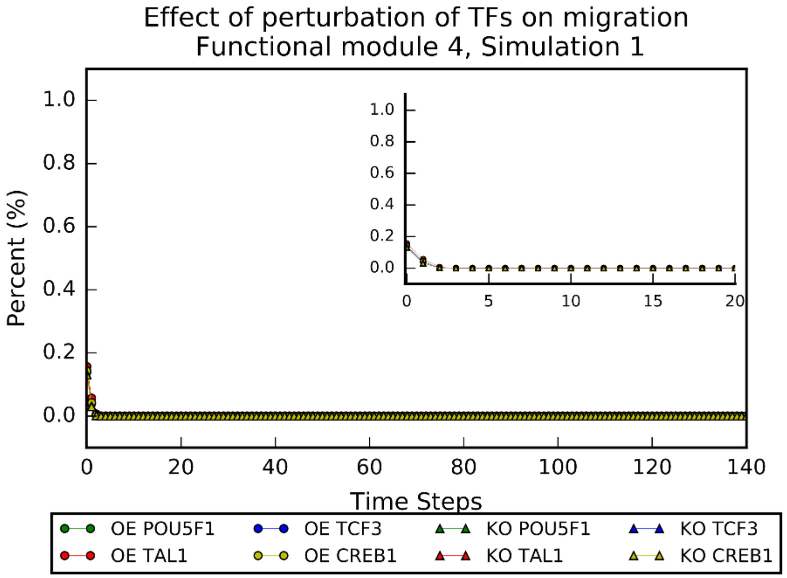
Effect of perturbation of TFs on migration
Functional module 4, Simulation 1
Percent (%)
Time Steps
OE POU5F1
OE TAL1
OE TCF3
OE CREB1
KO POU5F1
KO TAL1
KO TCF3
KO CREB1

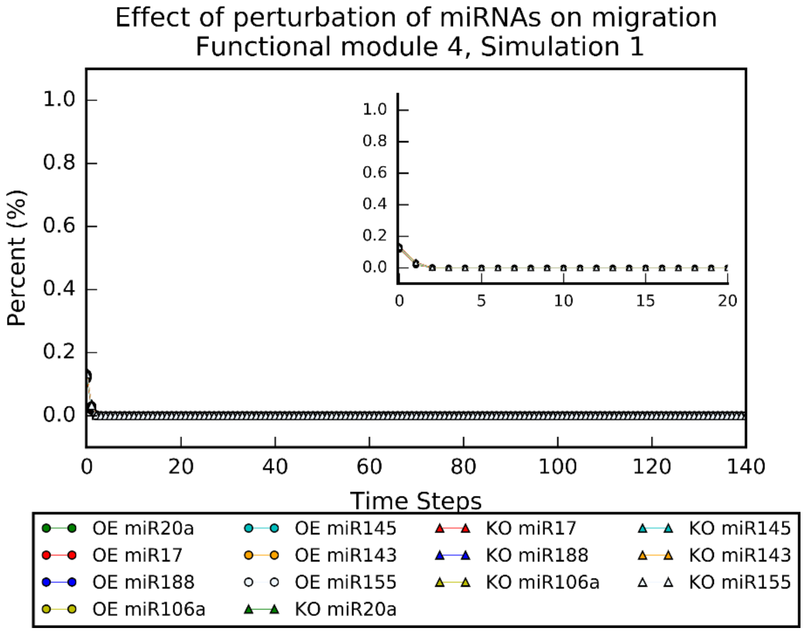
Effect of perturbation of miRNAs on migration
Functional module 4, Simulation 1
Percent (%)
Time Steps
OE miR20a
OE miR17
OE miR188
OE miR106a
OE miR145
OE miR143
OE miR155
KO miR20a
KO miR17
KO miR188
KO miR106a
KO miR145
KO miR143
KO miR155

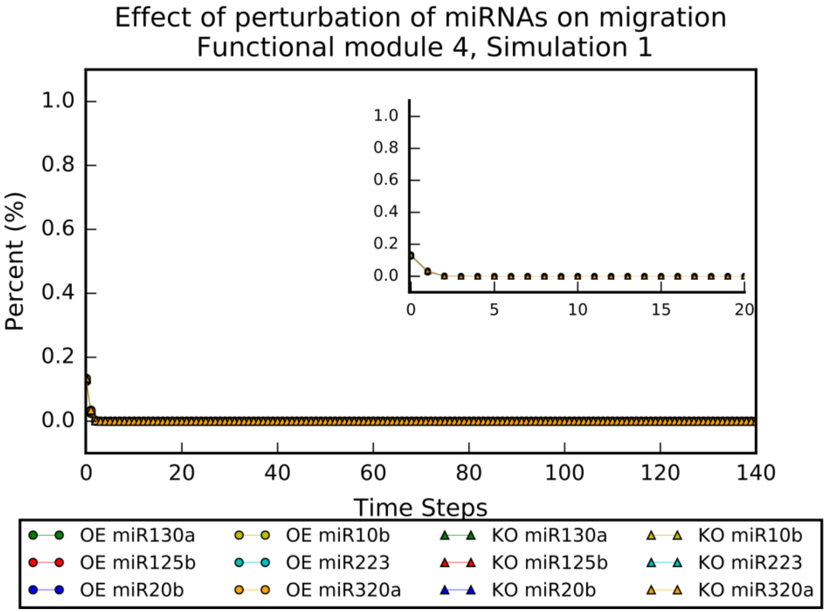

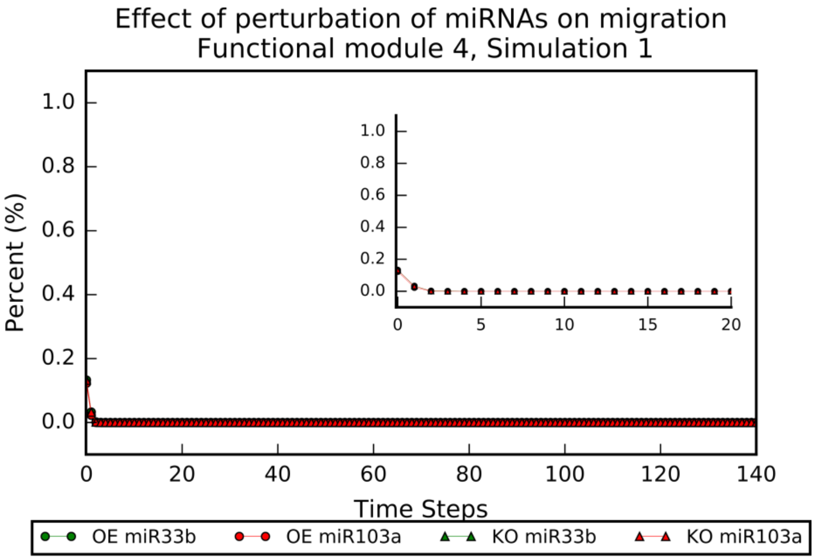

Supplementary Figure S15: Perturbation analysis FM4 Simulation 1

TF activates gene and miRNA expression. Perturbation of each gene did not upregulate migration as expression of all genes in FM4 is essential for migration.

Perturbation of each TF did not upregulate migration, as when TF was OE, miRNAs repress gene expression and when TF was knocked-out (KO), gene expression gets downregulated or miRNAs inhibit gene expression.

Perturbation of each miRNA did not upregulate migration, as when miRNA was OE, TFs upregulating gene expression gets repressed. Similarly, when each miRNA was KO, migration was not upregulated as other expressed miRNAs in the functional module repress TF expression

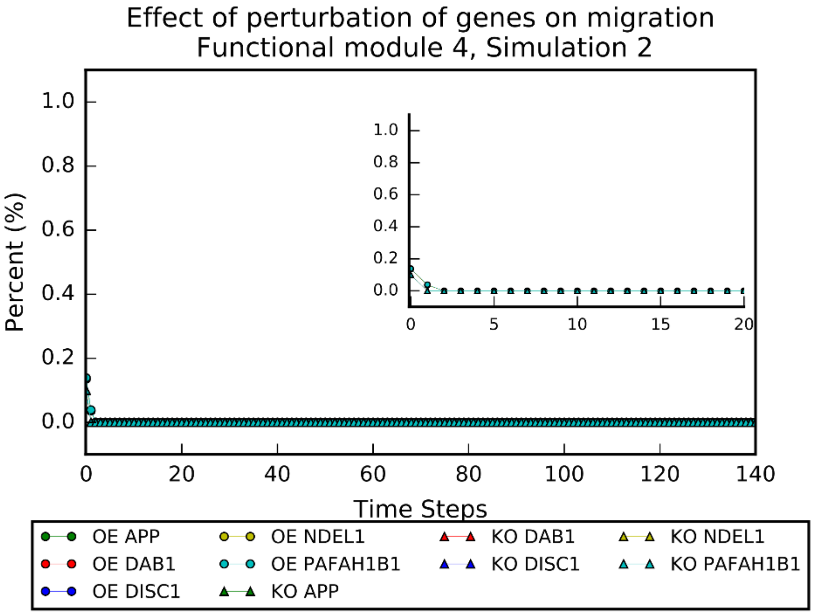
Effect of perturbation of genes on migration
Functional module 4, Simulation 2
Percent (%)
Time Steps
OE APP
OE DAB1
OE DISC1
OE NDEL1
OE PAFAH1B1
KO APP
KO DAB1
KO DISC1
KO NDEL1
KO PAFAH1B1

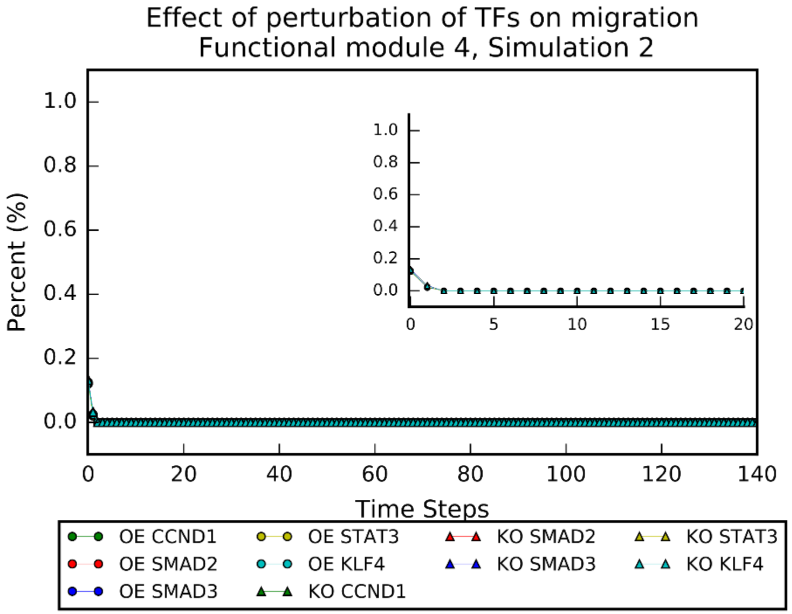
Effect of perturbation of TFs on migration
Functional module 4, Simulation 2
Percent (%)
Time Steps
OE CCND1
OE SMAD2
OE SMAD3
OE STAT3
OE KLF4
KO CCND1
KO SMAD2
KO SMAD3
KO STAT3
KO KLF4

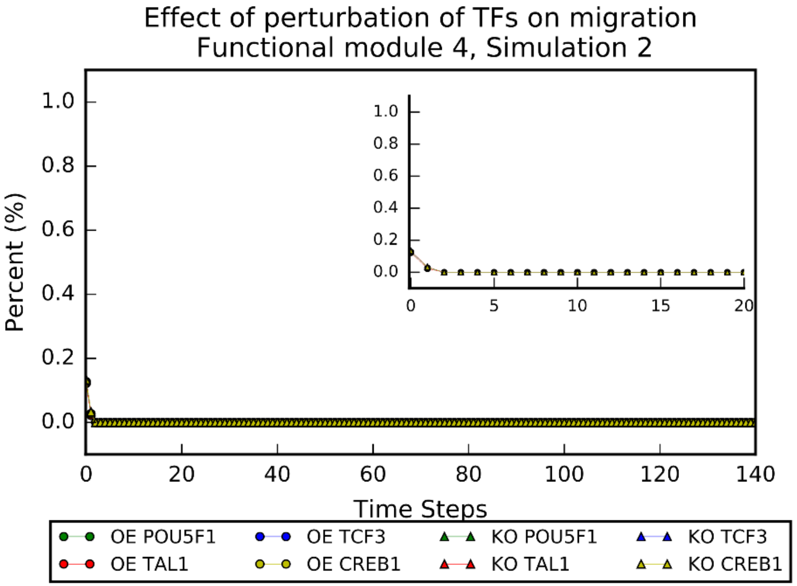
Effect of perturbation of TFs on migration
Functional module 4, Simulation 2
Percent (%)
Time Steps
OE POU5F1
OE TAL1
OE TCF3
OE CREB1
KO POU5F1
KO TAL1
KO TCF3
KO CREB1

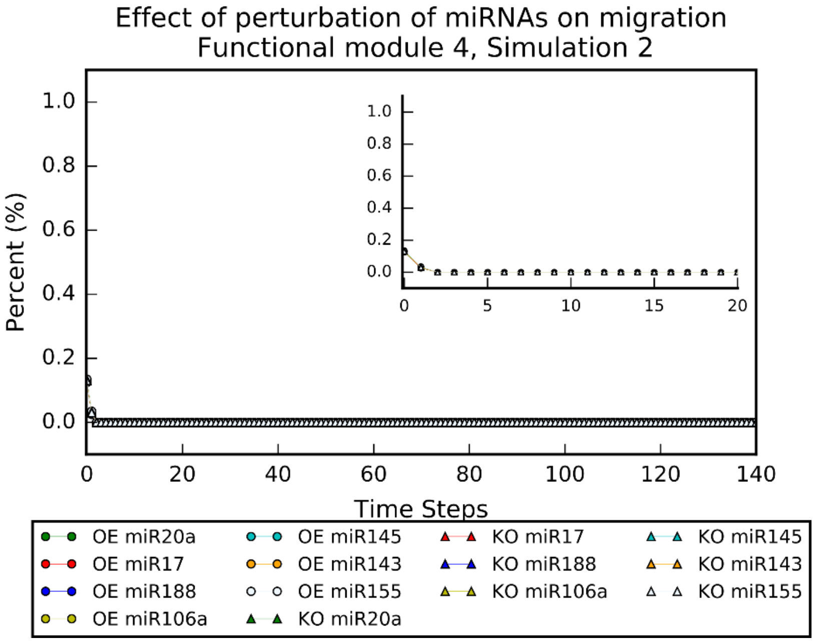
Effect of perturbation of miRNAs on migration
Functional module 4, Simulation 2
Percent (%)
Time Steps
OE miR20a
OE miR17
OE miR188
OE miR106a
OE miR145
OE miR143
OE miR155
KO miR20a
KO miR17
KO miR188
KO miR106a
KO miR145
KO miR143
KO miR155

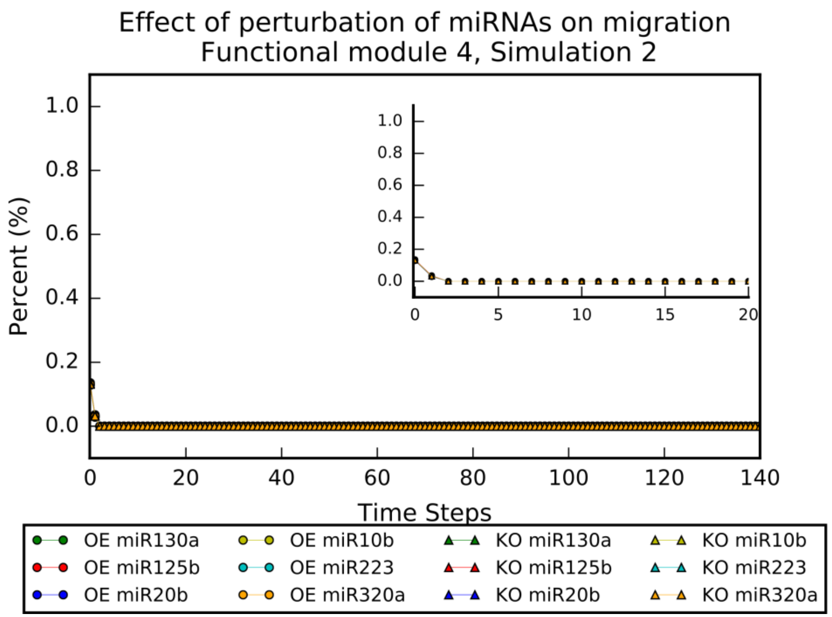

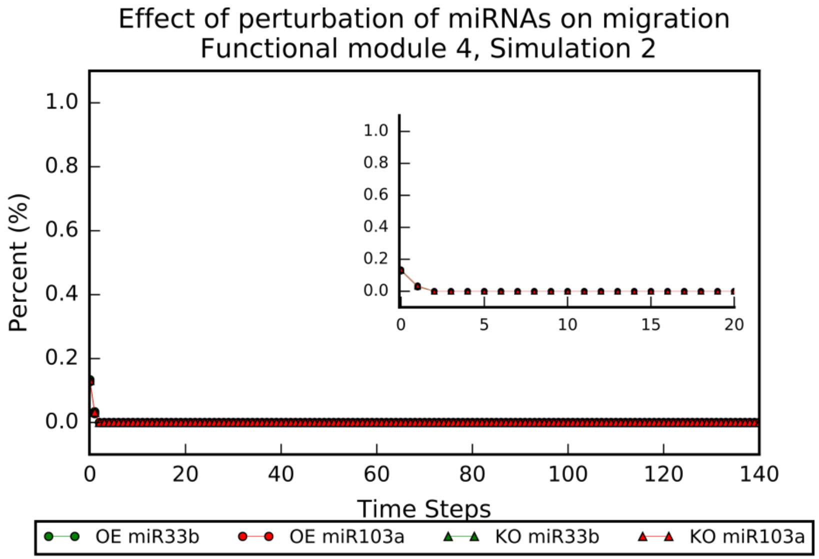

Supplementary Figure S16: Perturbation analysis FM4 Simulation 2

TF inhibits gene and miRNA expression. Perturbation of each gene did not upregulate migration as expression of all genes in FM4 is essential for migration.
Perturbation of each TF did not upregulate migration, as when TF was overexpressed (OE), miRNA or TF represses gene expression and when TF was knocked-out (KO), other expressed TFs or miRNAs in the functional module inhibited gene expression.
Perturbation of each miRNA did not upregulate migration, as when miRNA was OE, it inhibits gene expression. Similarly, when each miRNA was KO, migration was not upregulated as TF either activates miRNA expression or inhibits gene expression

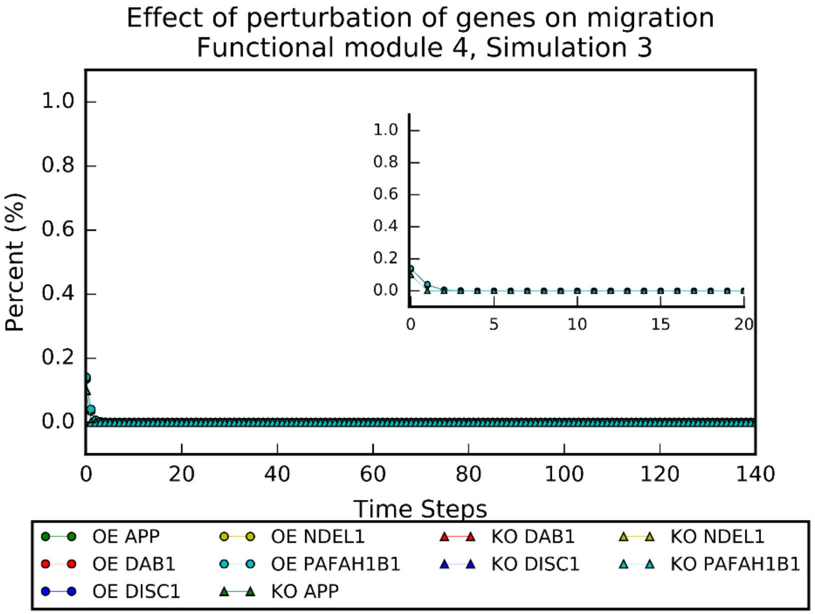
Effect of perturbation of genes on migration
Functional module 4, Simulation 3
Percent (%)
Time Steps
OE APP
OE DAB1
OE DISC1
OE NDEL1
OE PAFAH1B1
KO APP
KO DAB1
KO DISC1
KO NDEL1
KO PAFAH1B1

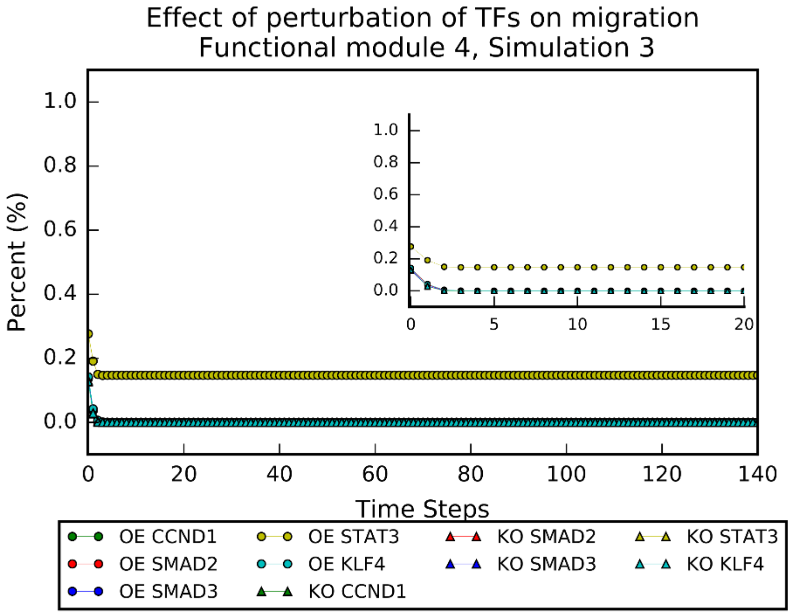
Effect of perturbation of TFs on migration
Functional module 4, Simulation 3
Percent (%)
Time Steps
OE CCND1
OE SMAD2
OE SMAD3
OE STAT3
OE KLF4
KO CCND1
KO SMAD2
KO SMAD3
KO STAT3
KO KLF4

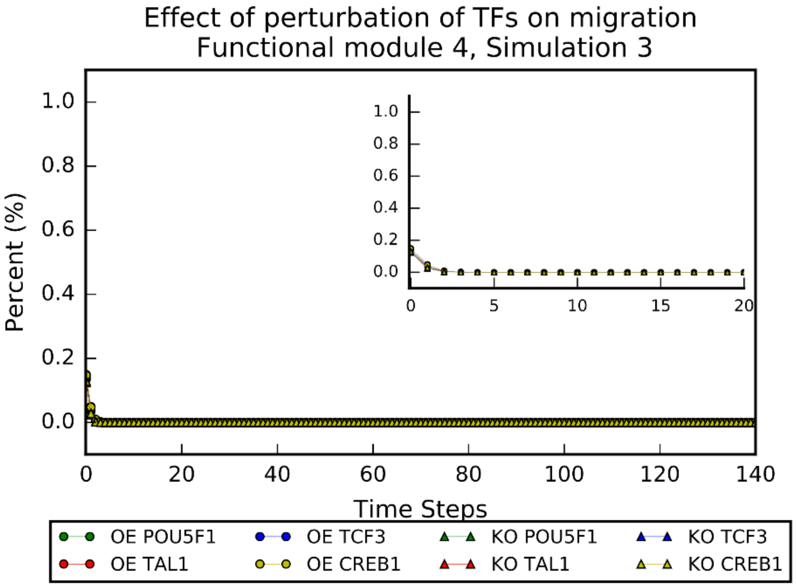
Effect of perturbation of TFs on migration
Functional module 4, Simulation 3
Percent (%)
Time Steps
OE POU5F1
OE TAL1
OE TCF3
OE CREB1
KO POU5F1
KO TAL1
KO TCF3
KO CREB1

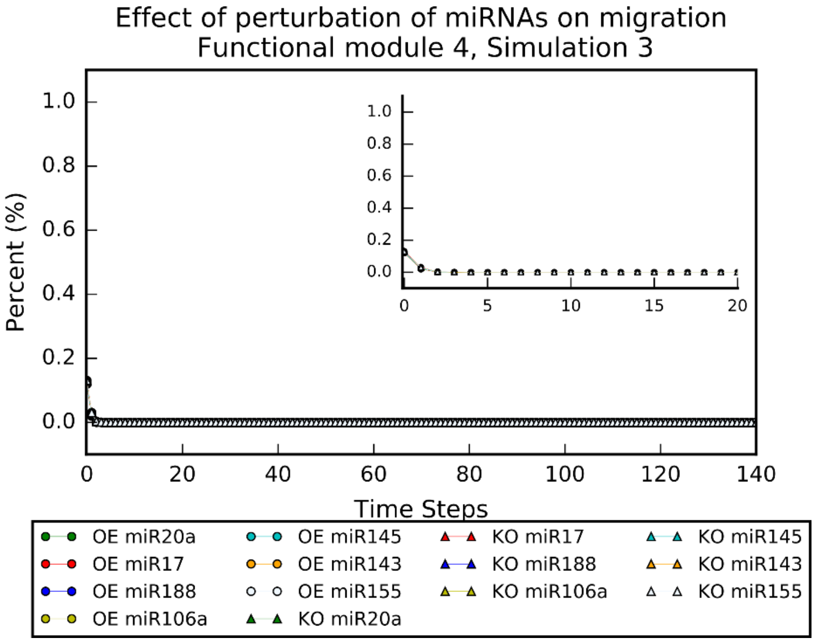
Effect of perturbation of miRNAs on migration
Functional module 4, Simulation 3
Percent (%)
Time Steps
OE miR20a
OE miR17
OE miR188
OE miR106a
OE miR145
OE miR143
OE miR155
KO miR20a
KO miR17
KO miR188
KO miR106a
KO miR145
KO miR143
KO miR155

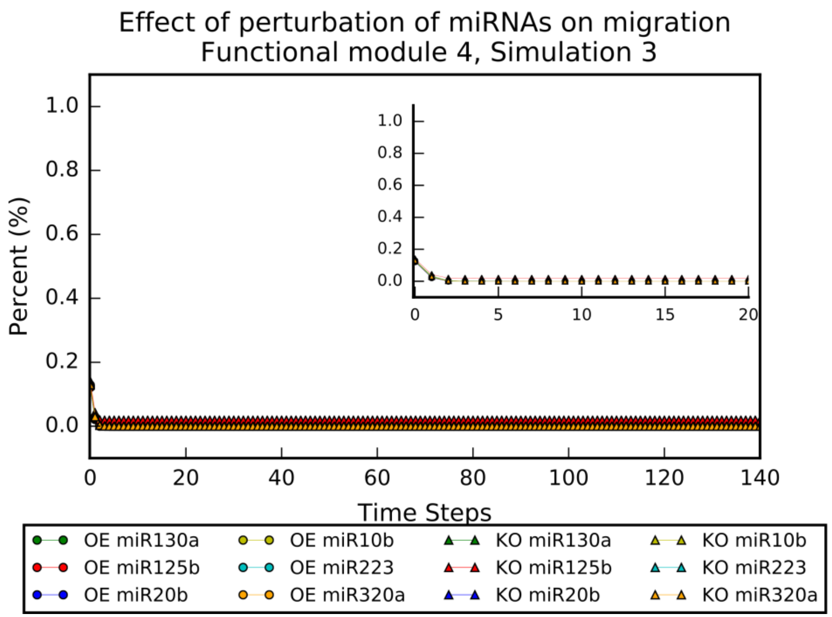

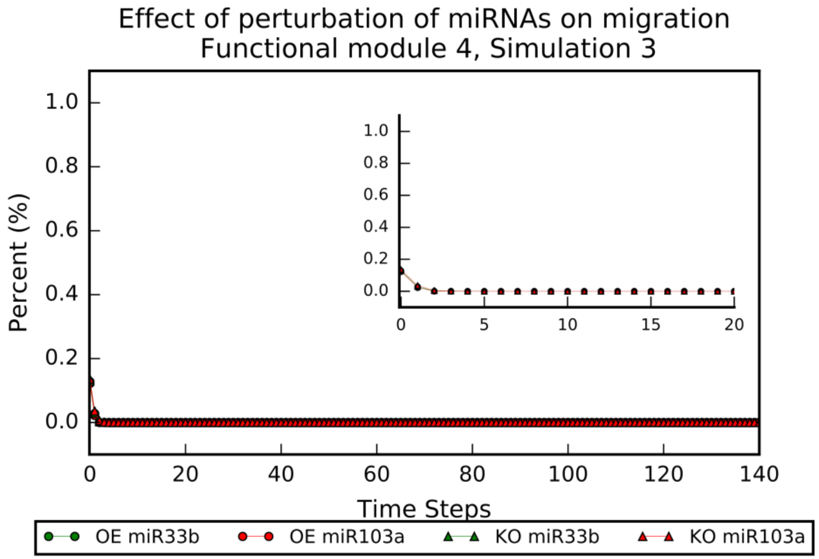

Supplementary Figure S17: Perturbation analysis FM4 Simulation 3

TF inhibits miRNA and activates gene expression. Perturbation of each gene did not upregulate migration as expression of all genes in FM4 is essential for migration.
Perturbation of each TF did not upregulate migration, as when TF was OE, miRNAs represses gene expression and when TF was knocked-out (KO), gene expression was downregulated or miRNAs inhibits gene expression.
Perturbation of each miRNA did not upregulate migration, as when miRNA was OE, TF upregulating gene expression gets repressed. Similarly, when each miRNA was KO, TF/gene gets repressed by other expressed miRNAs in the functional module.

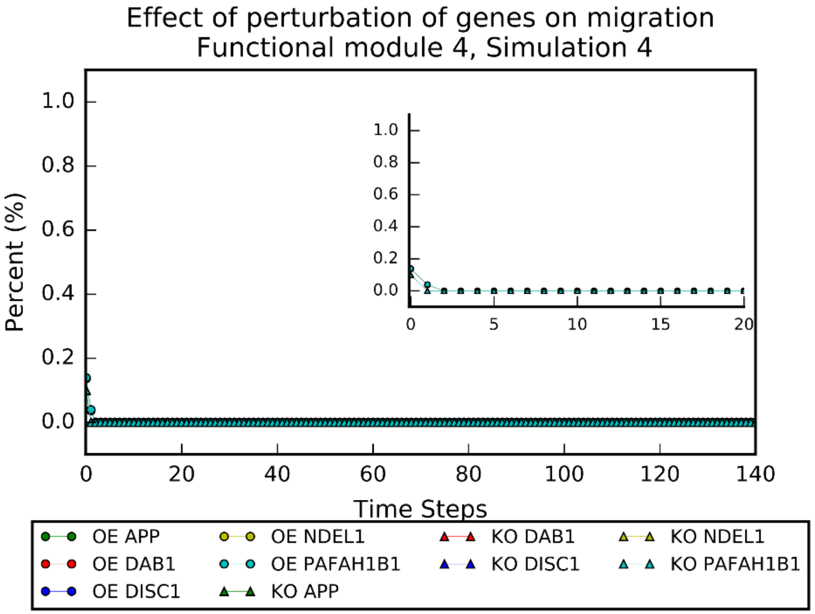
Effect of perturbation of genes on migration
Functional module 4, Simulation 4
Percent (%)
Time Steps
OE APP
OE DAB1
OE DISC1
OE NDEL1
OE PAFAH1B1
KO APP
KO DAB1
KO DISC1
KO NDEL1
KO PAFAH1B1

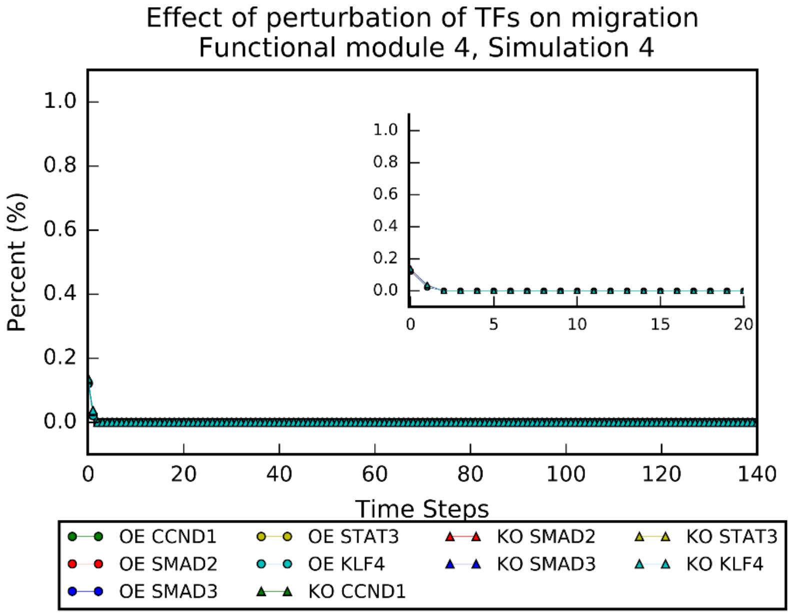
Effect of perturbation of TFs on migration
Functional module 4, Simulation 4
Percent (%)
Time Steps
OE CCND1
OE SMAD2
OE SMAD3
OE STAT3
OE KLF4
KO CCND1
KO SMAD2
KO SMAD3
KO STAT3
KO KLF4

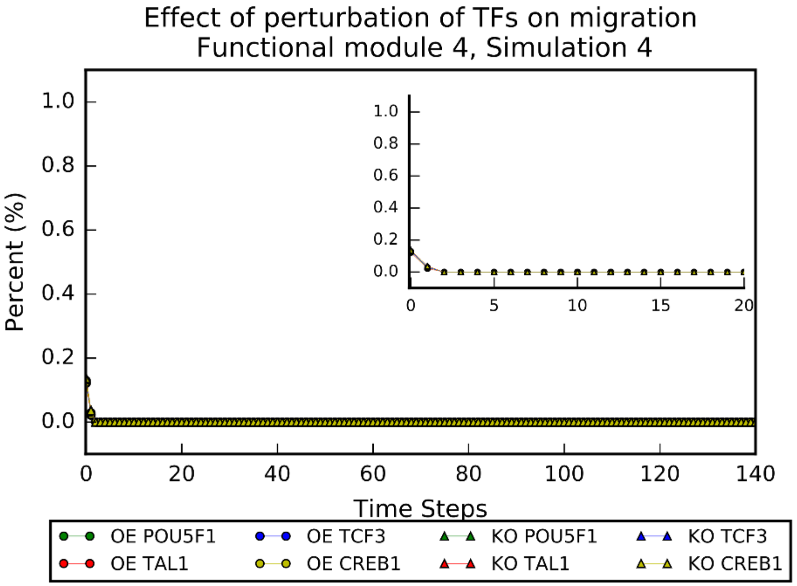
Effect of perturbation of TFs on migration
Functional module 4, Simulation 4
Percent (%)
Time Steps
OE POU5F1
OE TAL1
OE TCF3
OE CREB1
KO POU5F1
KO TAL1
KO TCF3
KO CREB1

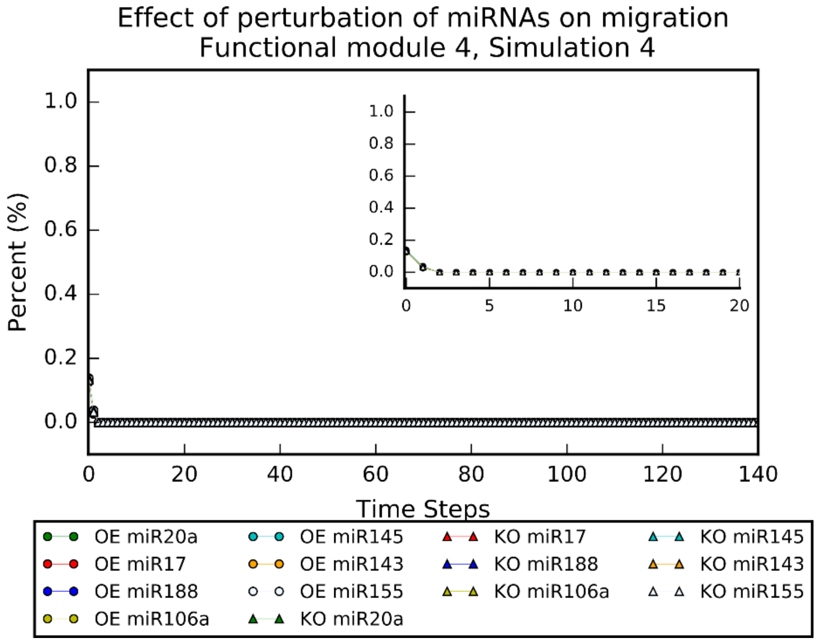
Effect of perturbation of miRNAs on migration
Functional module 4, Simulation 4
Percent (%)
Time Steps
OE miR20a
OE miR17
OE miR188
OE miR106a
OE miR145
OE miR143
OE miR155
KO miR20a
KO miR17
KO miR188
KO miR106a
KO miR145
KO miR143
KO miR155

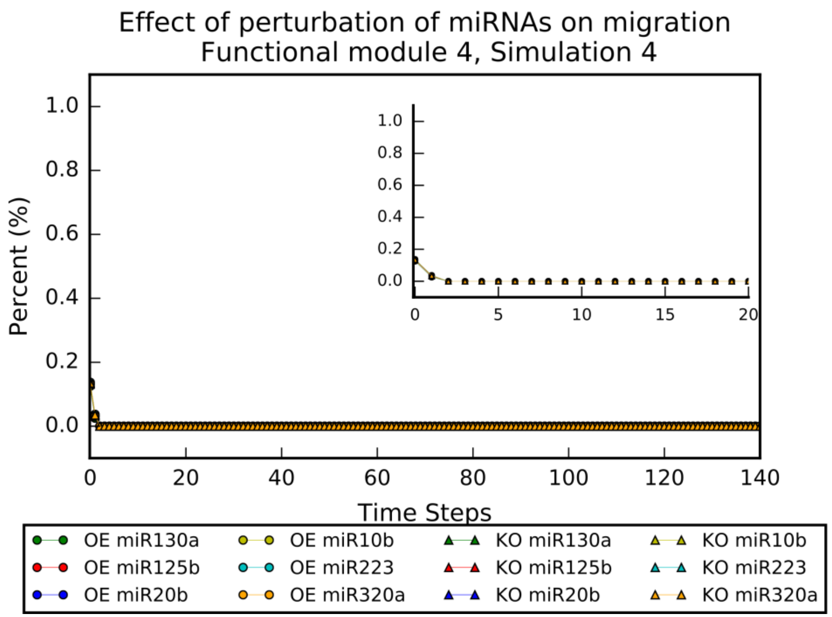

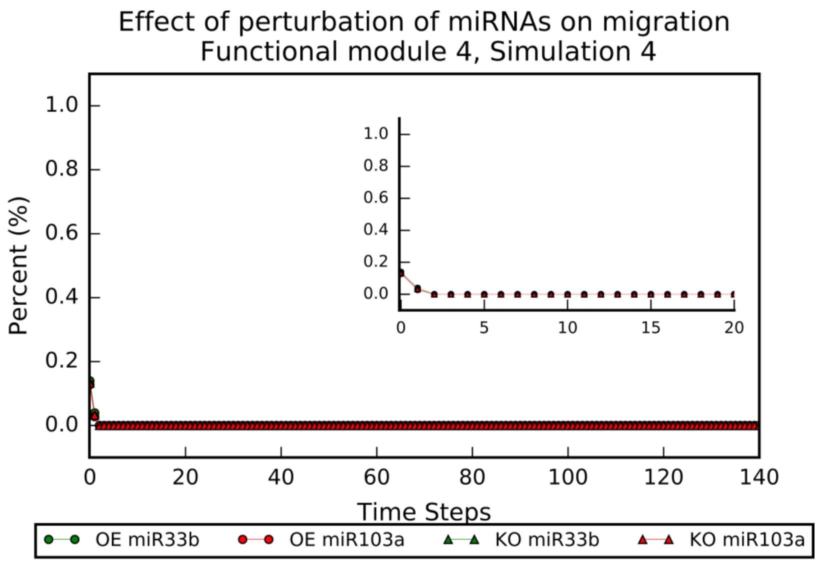

Supplementary Figure S18: Perturbation analysis FM4 Simulation 4

TF inhibits gene and activates miRNA expression. Perturbation of each gene did not upregulate migration as expression of all genes in FM4 is essential for migration.
Perturbation of each TF did not upregulate migration, as when TF was overexpressed (OE), miRNAs or TFs repress gene expression and when TF was knocked-out (KO), other expressed TFs or miRNAs in the functional module inhibited gene expression.
Perturbation of each miRNA did not upregulate migration, as when miRNA was OE, it inhibited gene expression. Similarly, when each miRNA was KO, migration was not upregulated as TF either activated miRNA expression or inhibited gene expression

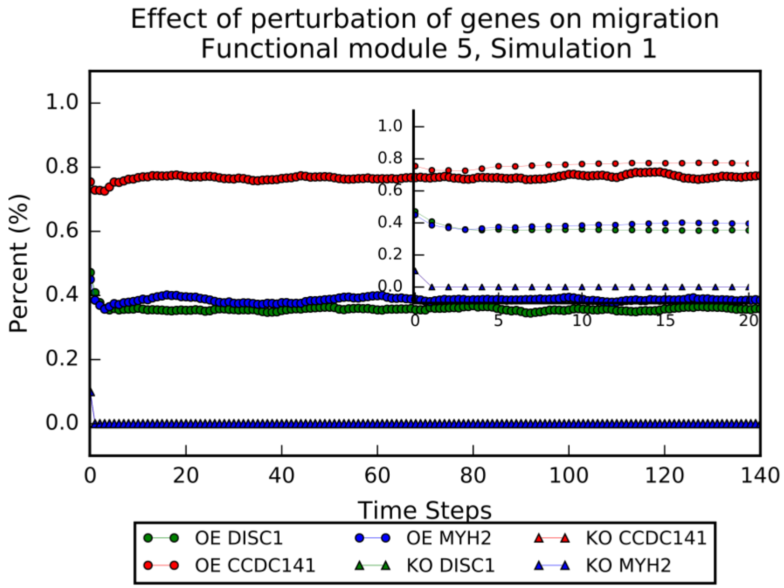

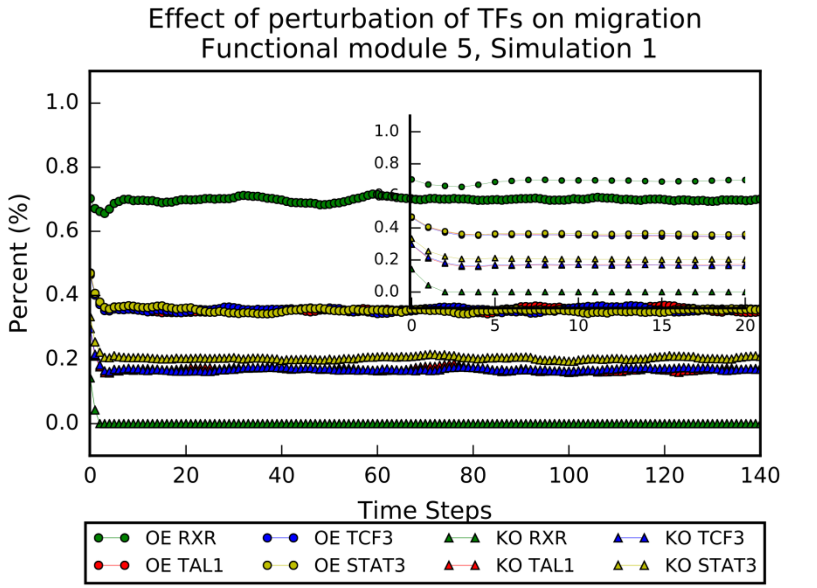

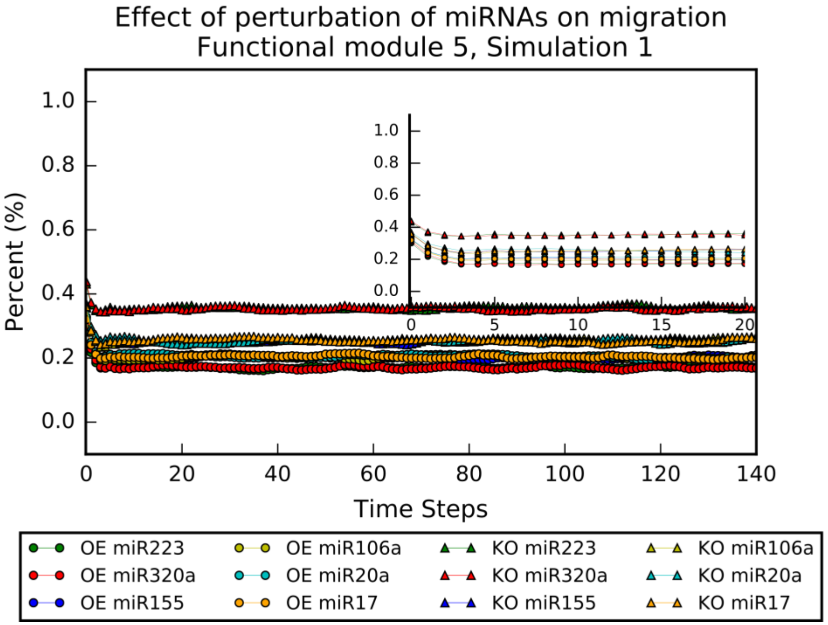

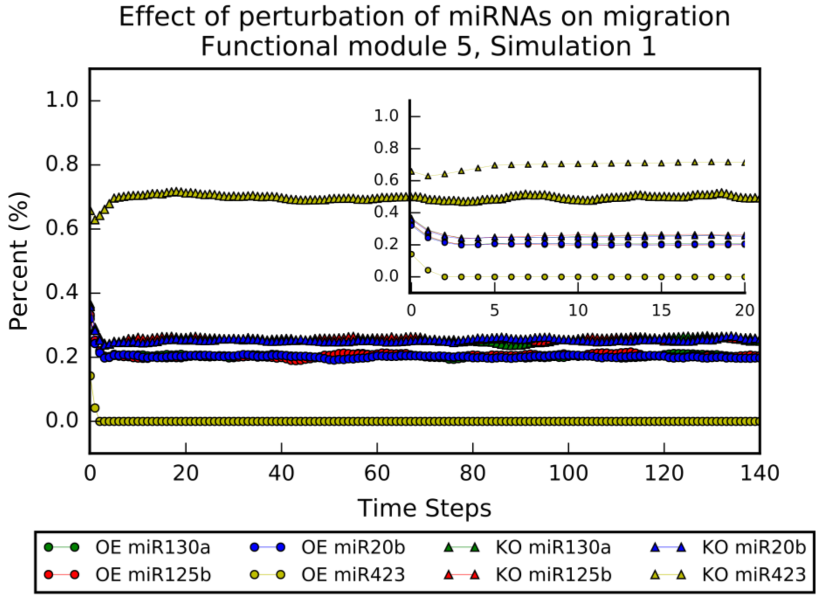

Supplementary Figure S19: Perturbation analysis FM5 Simulation 1

TF activates gene and miRNA expression. Perturbation of each gene did not upregulate migration, as expression of all genes is essential for migration in FM5.
Overexpression (OE) of CCDC141 showed regulation of migration (75-77%) as CCDC141 regulates interaction b/w DISC1 and MYH2. Perturbation of each TF did not upregulate migration, as miRNA represses gene expression. When RXR was OE, migration was regulated (70%) as CCDC141 gene was expressed. Expressed CCDC141 gene mediates interaction between DISC1 and MYH2 thereby regulating migration (70%).
Perturbation of each miRNA did not upregulate migration, as TFs/genes were repressed by miRNA. KO of miR423 regulating RXR expression showed regulation of migration (65%-69%)

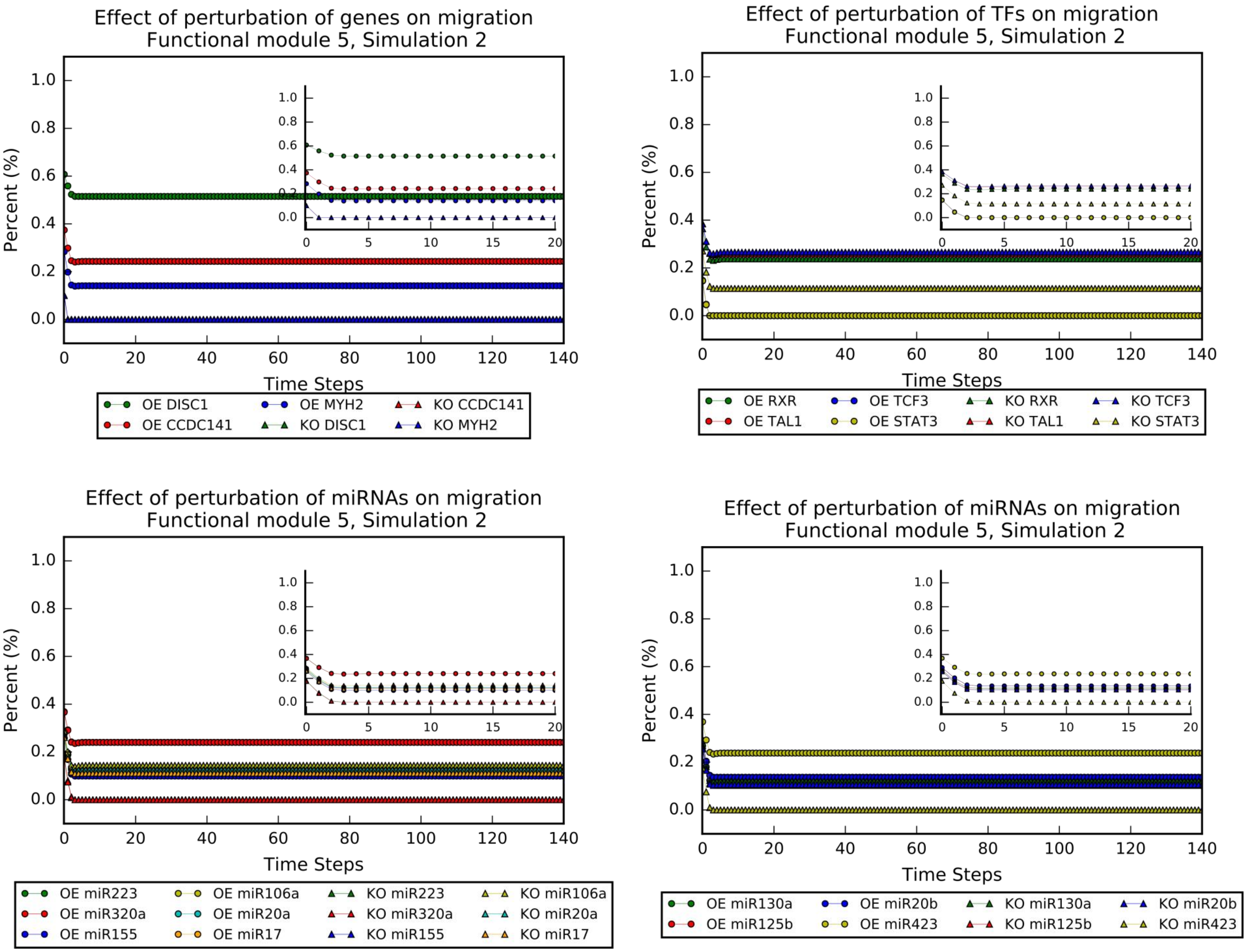

Supplementary Figure S20: Perturbation analysis FM5 Simulation 2

TF represses gene and miRNA expression. Perturbation of each gene did not upregulate migration, as expression of all genes is essential for migration in FM5. DISC1 overexpression (OE) showed regulation of migration (51%). DISC1 is expressed, only when all 3 TFs, TAL1, TCF3 and STAT3 are not expressed, which happens less number of times when MYH2 or CCDC141 was OE. So, when DISC1 was OE, it interacts with CCDC141 and MYH2 and regulates migration. Perturbation of each TF did not upregulate migration, as miRNA or TF represses gene expression. KO of TAL1 and TCF3 showed regulation of migration (25% by TAL1; 26% by TCF3), as TAL1 and TCF3 downregulation, activated DISC1 expression (when STAT3 was also not expressed). KO of RXR showed regulation of migration up to 23%, as downregulation of RXR activated CCDC141 expression. Perturbation of each miRNA did not upregulate migration, as TFs or miRNAs repress gene expression. OE of miR223 or miR320a regulating TAL1 and TCF3 expression, showed regulation of migration (24%), as DISC1 is expressed. KO of miR223 or miR320a showed downregulation of migration as TAL1 and TCF3 gets expressed, downregulating DISC1 expression.

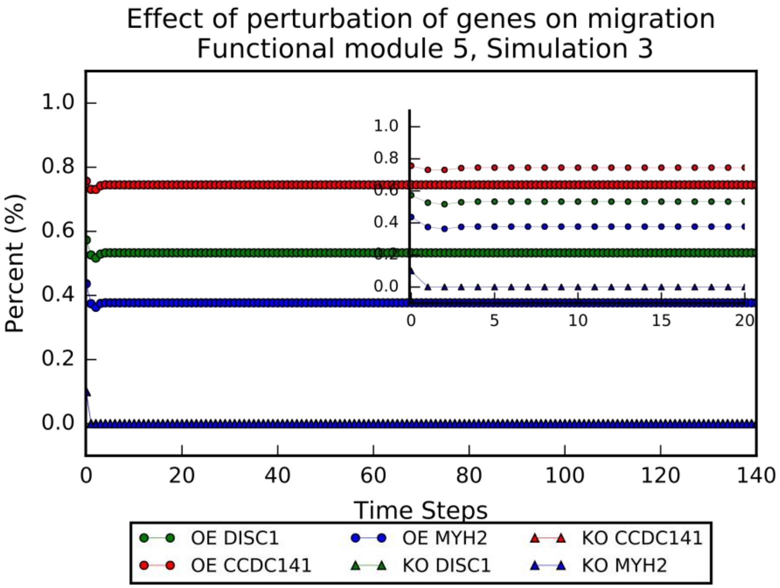

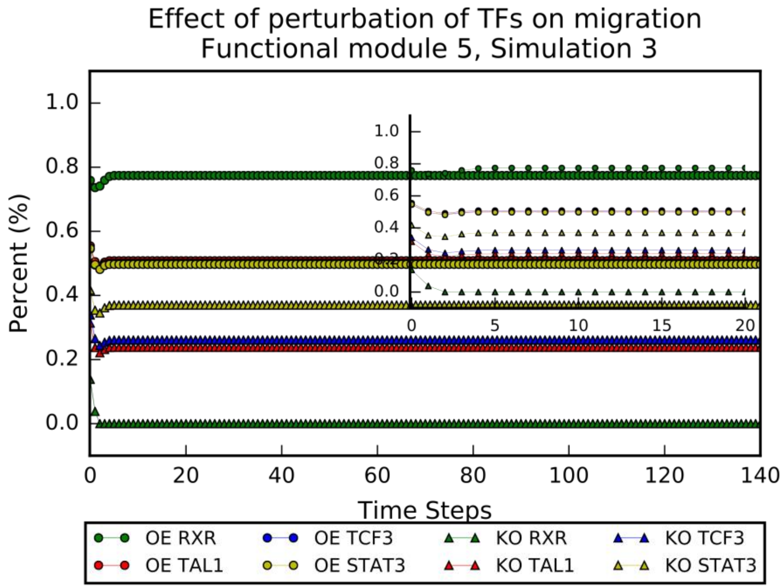

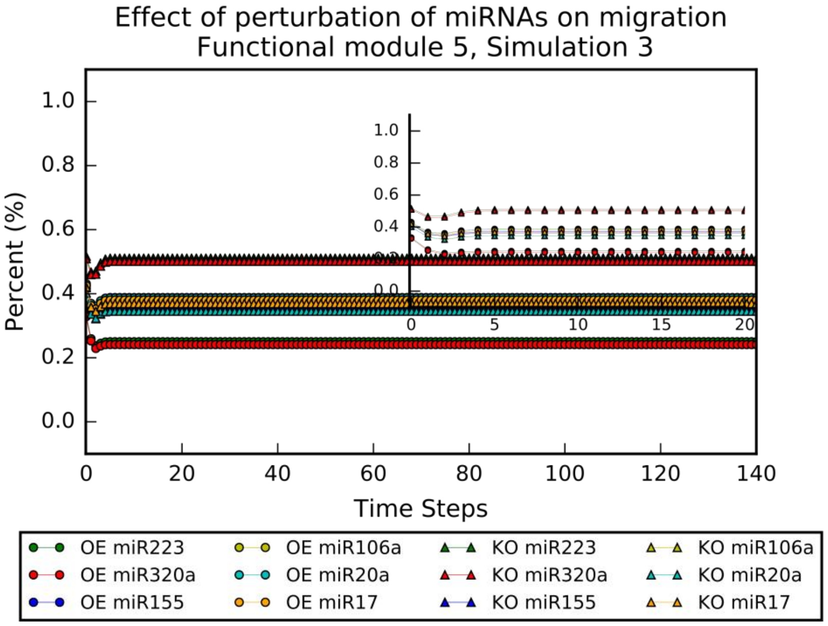

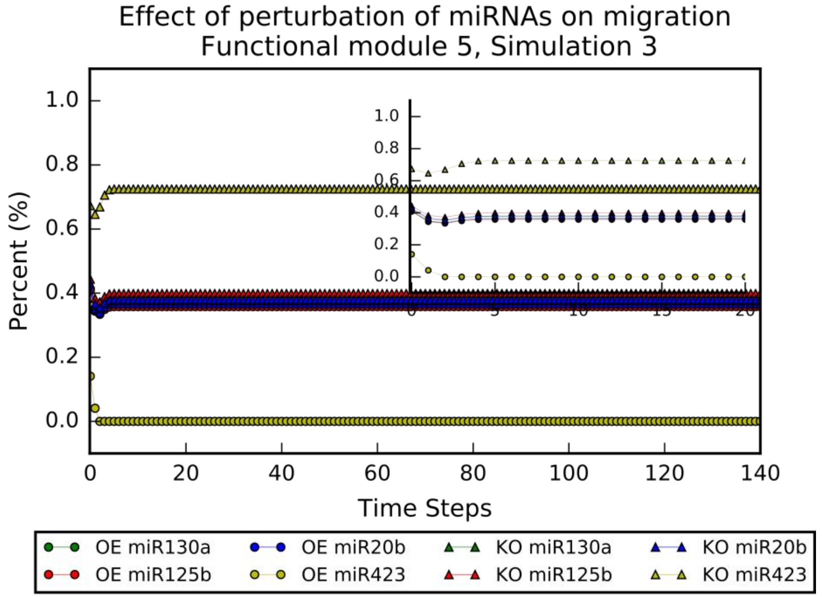

Supplementary Figure S21: Perturbation analysis FM5 Simulation 3

TF represses miRNA and activates gene expression. Perturbation of each gene did not upregulate migration, as expression of all genes is essential for migration in FM5. Overexpression (OE) of CCDC141 showed regulation of migration (74%), as CCDC141 regulates interaction b/w DISC1 and MYH2 and mediates migration.
Perturbation of each TF did not upregulate migration, as miRNA represses gene expression. When RXR was OE, migration was regulated (75-77%) as CCDC141 gene gets expressed. When STAT3 was KO, DISC1 gets expressed (by TAL1 and TCF3), regulating migration (37%).
Perturbation of each miRNA did not upregulate migration, as miRNAs repress TFs/genes expression. KO of miR423 regulating TF, RXR showed regulation of migration (72%), as CCDC141 gene was expressed. KO of miR223 or miR320a regulated migration (51% by miR320a and 50% by miR223), as TAL1 or TCF3 regulating DISC1 expression was expressed.

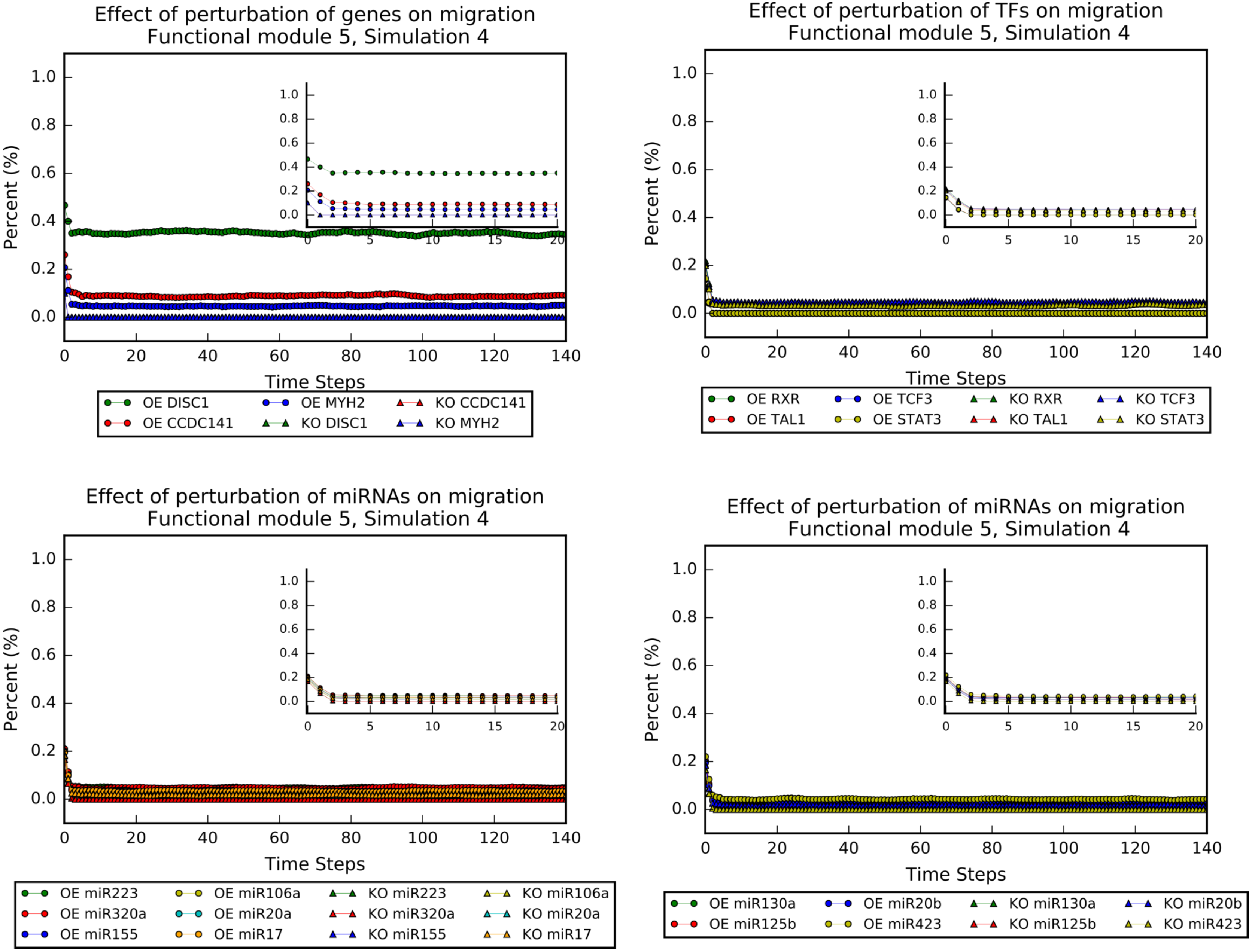

Supplementary Figure S22: Perturbation analysis FM5 Simulation 4

TF represses gene and activates miRNA expression. Perturbation of each gene did not upregulate migration, as expression of all genes is essential for migration in FM 5. DISC1 overexpression (OE) regulated migration (34%), as DISC1 interacts with CCDC141 and MYH2 and regulates migration. Perturbation of each TF did not upregulate migration, as miRNA or TF represses gene expression. Perturbation of each miRNA did not upregulate migration, as TFs or miRNAs repress gene

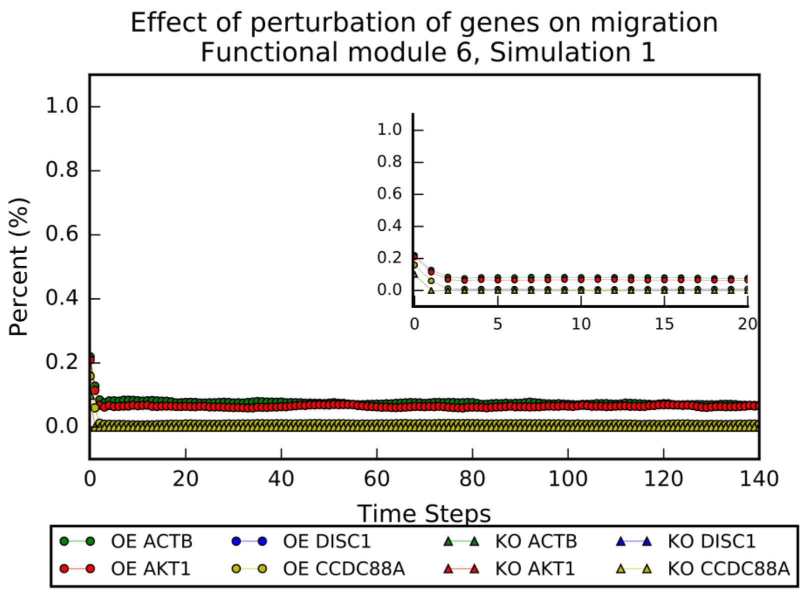
Effect of perturbation of genes on migration
Functional module 6, Simulation 1
Percent (%)
Time Steps
OE ACTB
OE AKT1
OE DISC1
OE CCDC88A
KO ACTB
KO AKT1
KO DISC1
KO CCDC88A

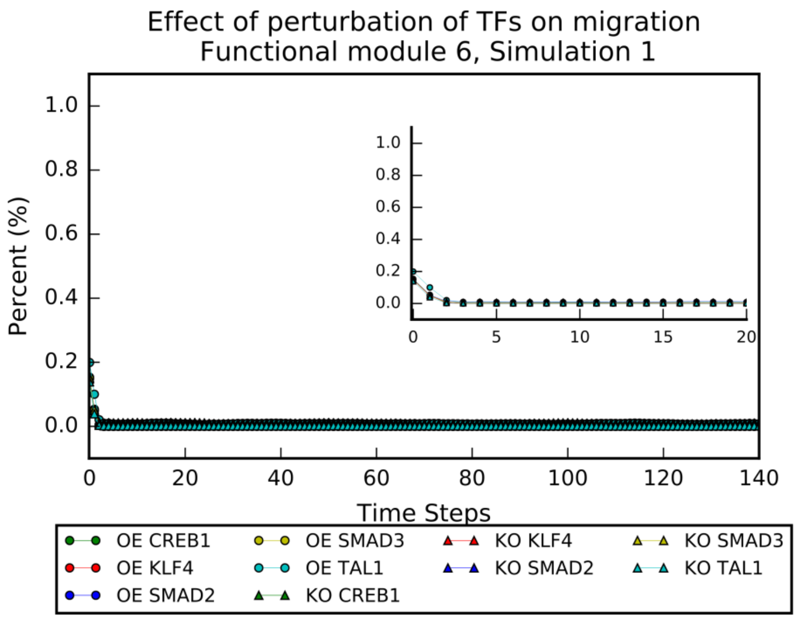
Effect of perturbation of TFs on migration
Functional module 6, Simulation 1
Percent (%)
Time Steps
OE CREB1
OE KLF4
OE SMAD2
OE SMAD3
OE TAL1
KO CREB1
KO KLF4
KO SMAD2
KO SMAD3
KO TAL1

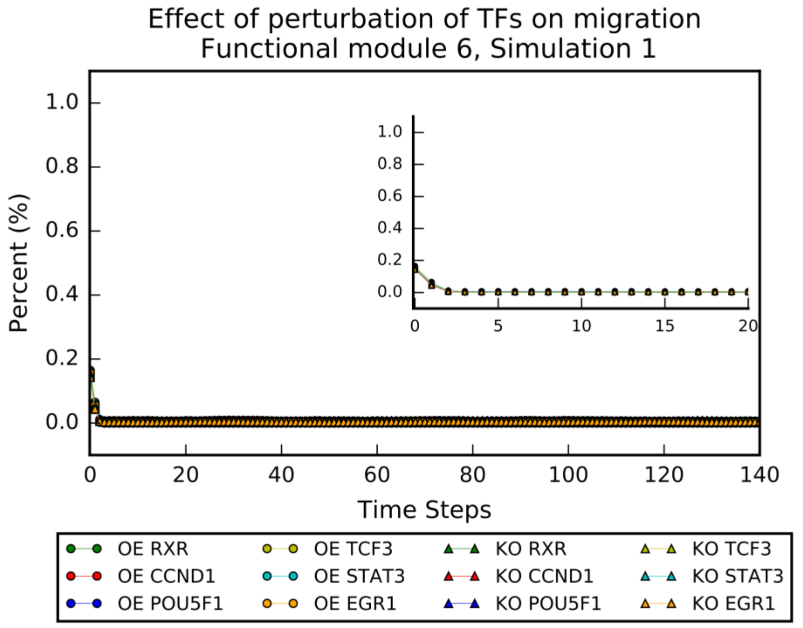
Effect of perturbation of TFs on migration
Functional module 6, Simulation 1
Percent (%)
Time Steps
OE RXR
OE CCND1
OE POU5F1
OE TCF3
OE STAT3
OE EGR1
KO RXR
KO CCND1
KO POU5F1
KO TCF3
KO STAT3
KO EGR1

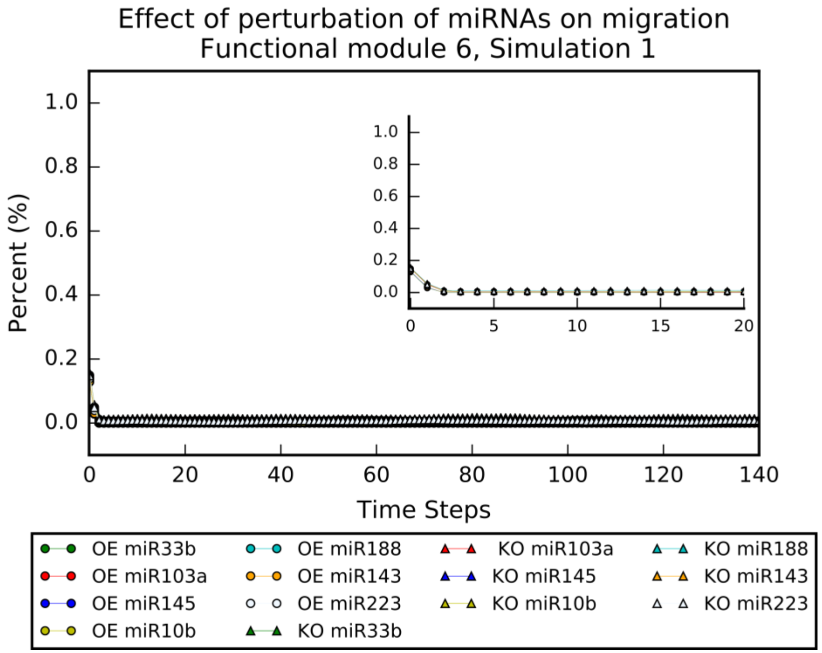
Effect of perturbation of miRNAs on migration
Functional module 6, Simulation 1
Percent (%)
Time Steps
OE miR33b
OE miR103a
OE miR145
OE miR10b
OE miR188
OE miR143
OE miR223
KO miR33b
KO miR103a
KO miR145
KO miR10b
KO miR188
KO miR143
KO miR223

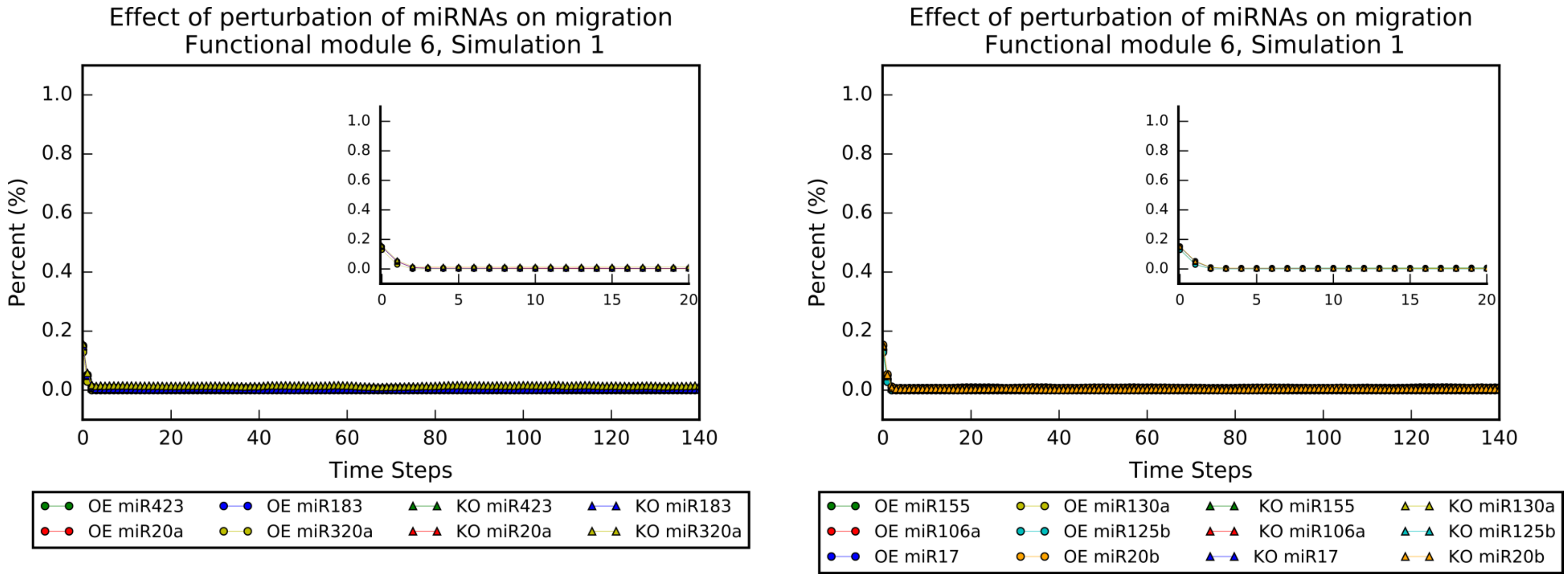

Supplementary Figure S23: Perturbation analysis FM6 Simulation 1

TF activates gene and miRNA expression. Perturbation of each gene did not upregulate migration as expression of all genes in FM6 is essential for migration.

Perturbation of each TF did not upregulate migration, as miRNA represses genes expression.

[illegible]

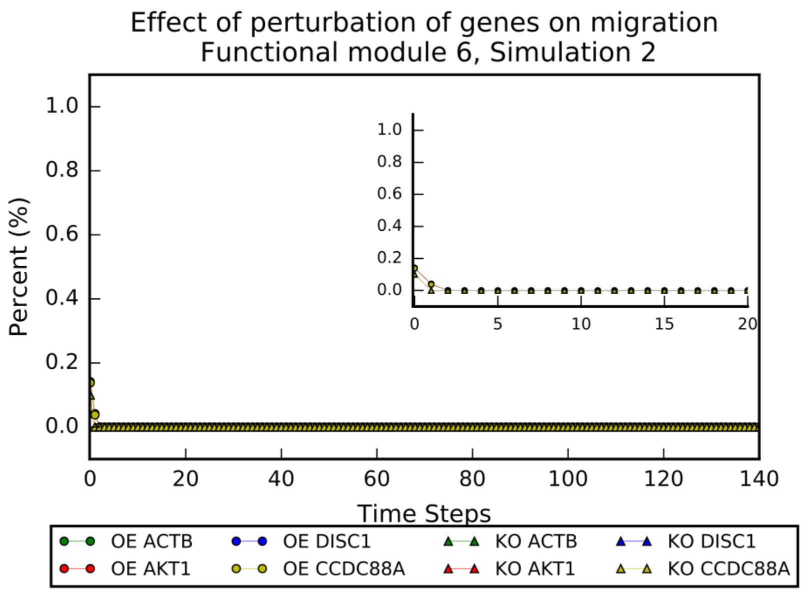
Effect of perturbation of genes on migration
Functional module 6, Simulation 2
Percent (%)
Time Steps
OE ACTB
OE AKT1
OE DISC1
OE CCDC88A
KO ACTB
KO AKT1
KO DISC1
KO CCDC88A

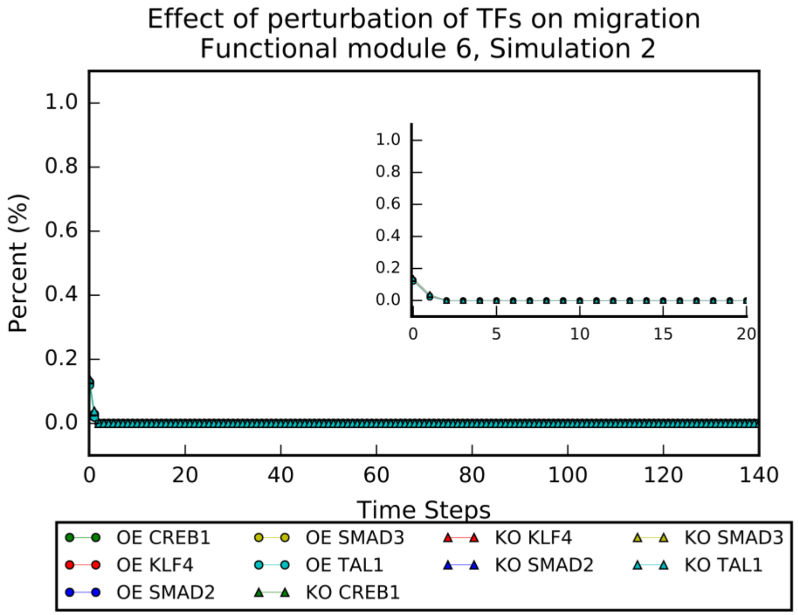
Effect of perturbation of TFs on migration
Functional module 6, Simulation 2
Percent (%)
Time Steps
OE CREB1
OE KLF4
OE SMAD2
OE SMAD3
OE TAL1
KO CREB1
KO KLF4
KO SMAD2
KO SMAD3
KO TAL1

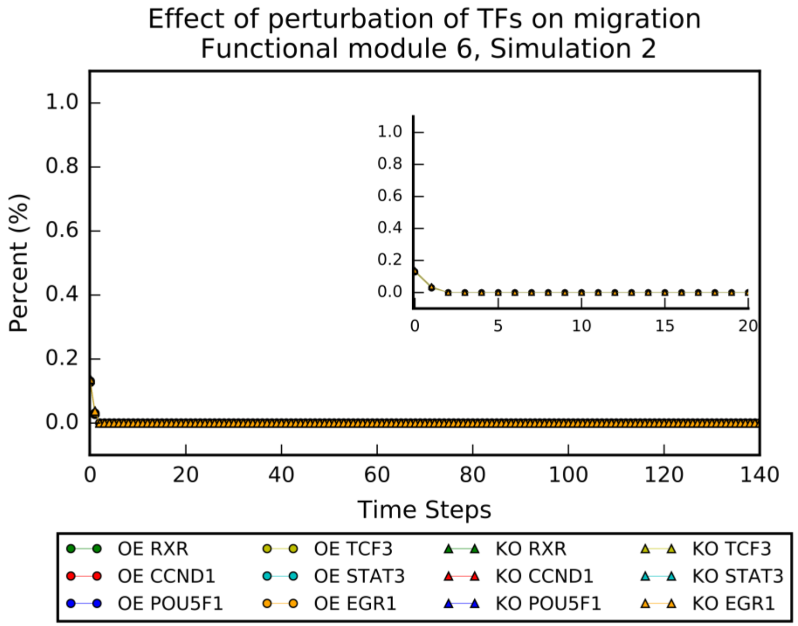
Effect of perturbation of TFs on migration
Functional module 6, Simulation 2
Percent (%)
Time Steps
OE RXR
OE CCND1
OE POU5F1
OE TCF3
OE STAT3
OE EGR1
KO RXR
KO CCND1
KO POU5F1
KO TCF3
KO STAT3
KO EGR1

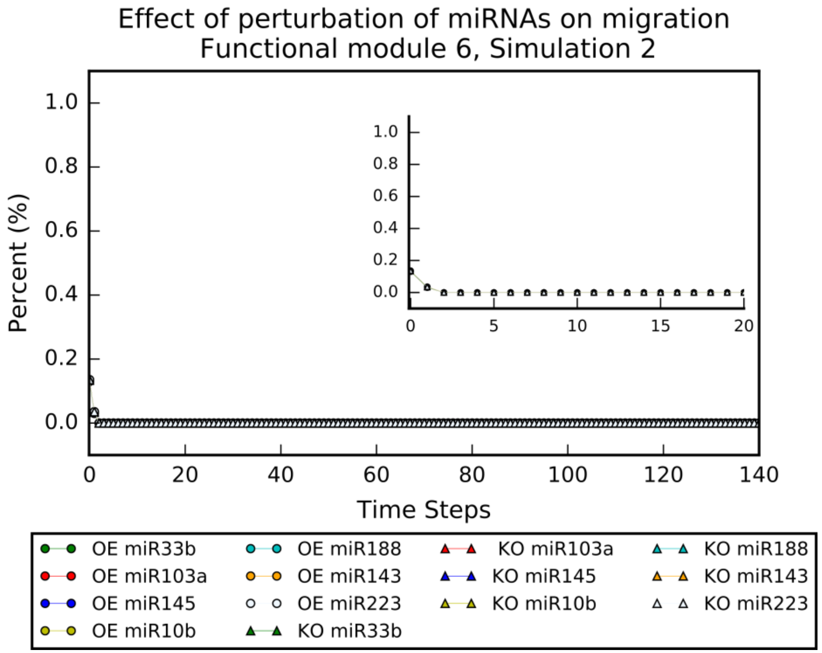
Effect of perturbation of miRNAs on migration
Functional module 6, Simulation 2
Percent (%)
Time Steps
OE miR33b
OE miR103a
OE miR145
OE miR10b
OE miR188
OE miR143
OE miR223
KO miR33b
KO miR103a
KO miR145
KO miR10b
KO miR188
KO miR143
KO miR223

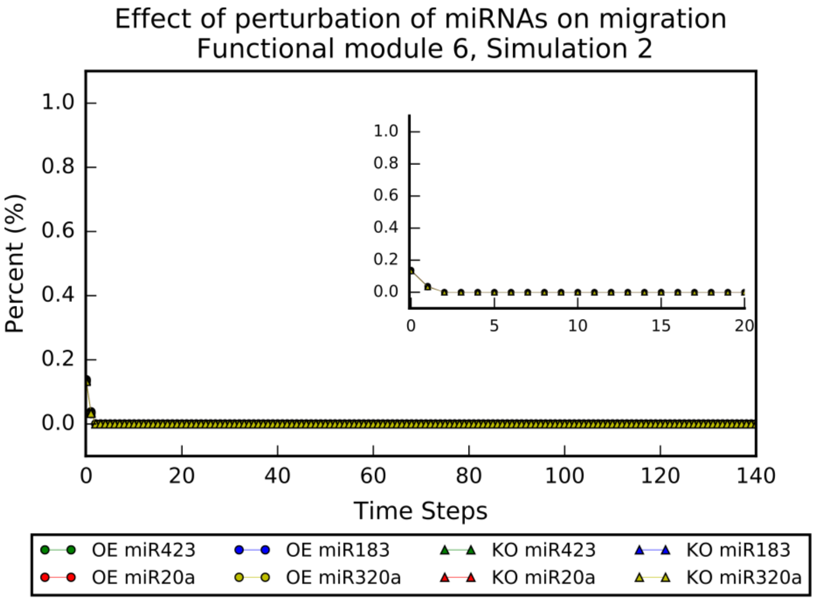

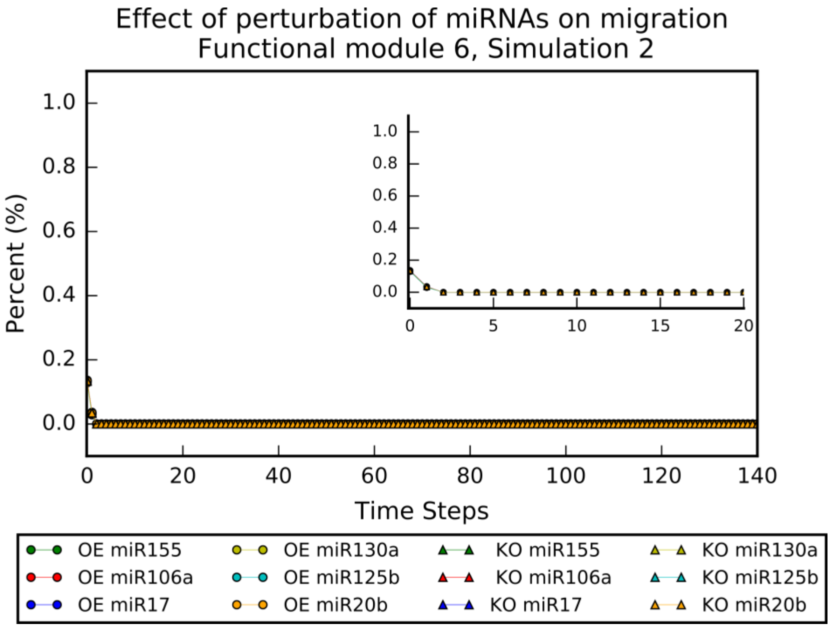

Supplementary Figure S24: Perturbation analysis FM6 Simulation 2

TF represses gene and miRNA expression. Perturbation of each gene did not upregulate migration as expression of all genes in FM6 is essential for migration.
Perturbation of each TF did not upregulate migration, as miRNA or TF represses gene expression.
[illegible]

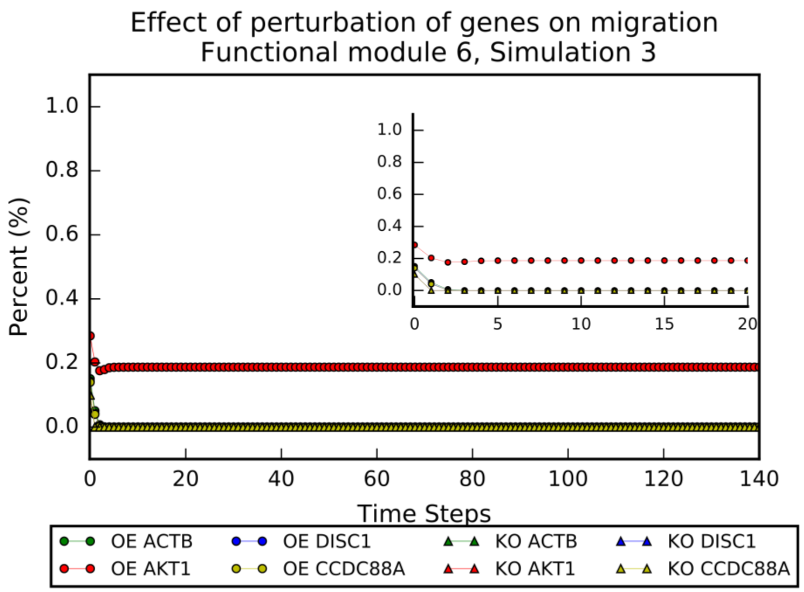
Effect of perturbation of genes on migration
Functional module 6, Simulation 3
Percent (%)
Time Steps
OE ACTB
OE AKT1
OE DISC1
OE CCDC88A
KO ACTB
KO AKT1
KO DISC1
KO CCDC88A

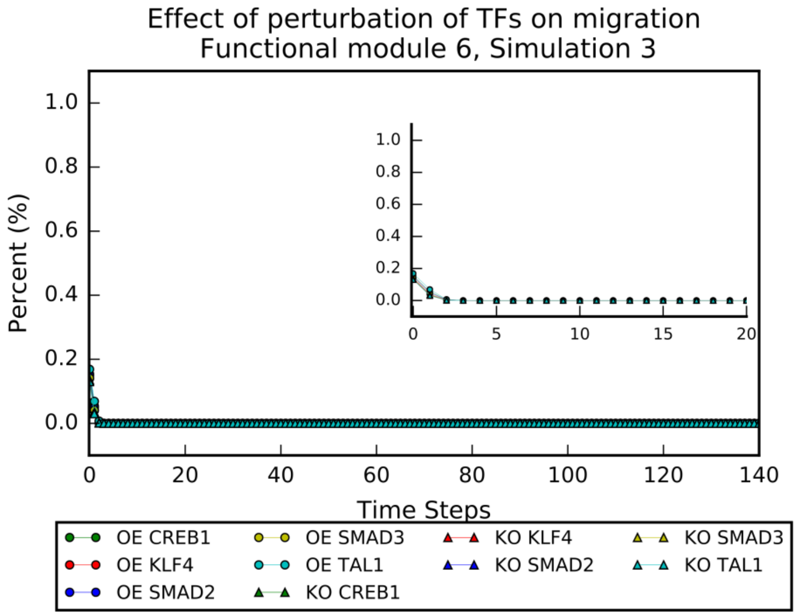
Effect of perturbation of TFs on migration
Functional module 6, Simulation 3
Percent (%)
Time Steps
OE CREB1
OE KLF4
OE SMAD2
OE SMAD3
OE TAL1
KO CREB1
KO KLF4
KO SMAD2
KO SMAD3
KO TAL1

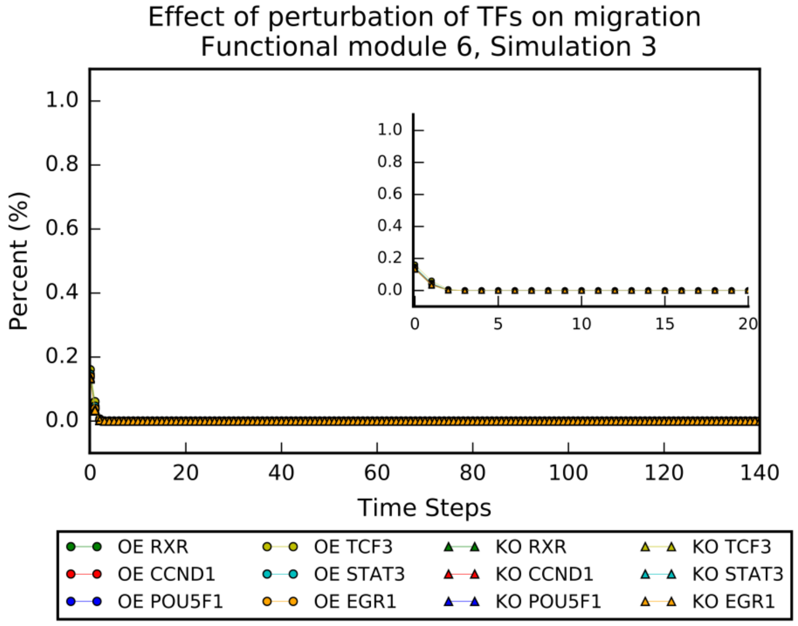
Effect of perturbation of TFs on migration
Functional module 6, Simulation 3
Percent (%)
Time Steps
OE RXR
OE CCND1
OE POU5F1
OE TCF3
OE STAT3
OE EGR1
KO RXR
KO CCND1
KO POU5F1
KO TCF3
KO STAT3
KO EGR1

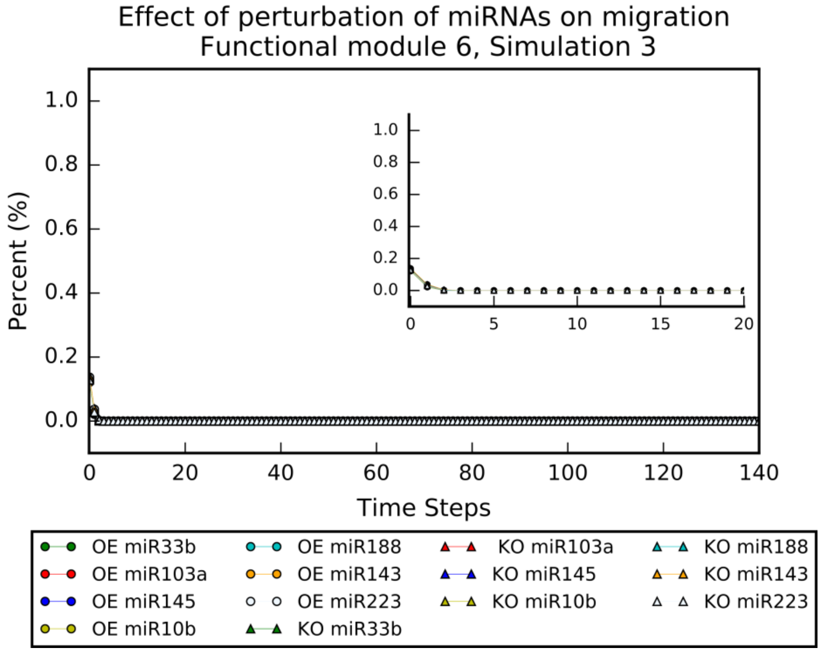
Effect of perturbation of miRNAs on migration
Functional module 6, Simulation 3
Percent (%)
Time Steps
OE miR33b
OE miR103a
OE miR145
OE miR10b
OE miR188
OE miR143
OE miR223
KO miR33b
KO miR103a
KO miR145
KO miR10b
KO miR188
KO miR143
KO miR223

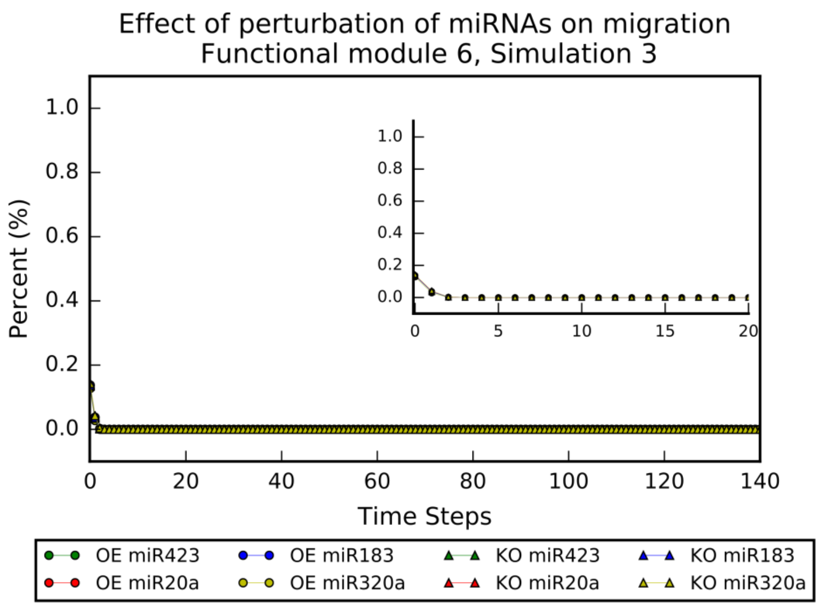

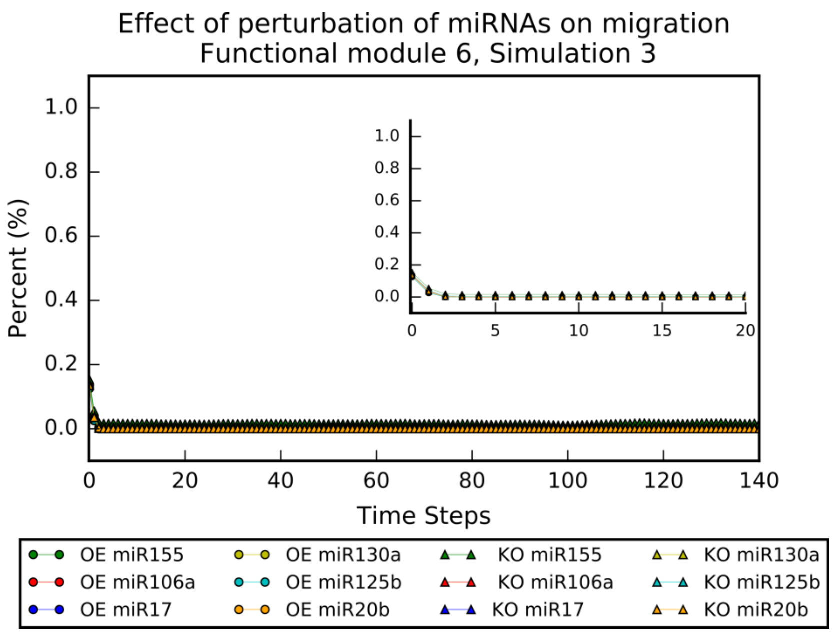

Supplementary Figure S25: Perturbation analysis FM6 Simulation 3

TF represses miRNA and activates gene expression. Perturbation of each gene did not upregulate migration as expression of all genes in FM6 is essential for migration. When AKT1 gene was overexpressed (OE), migration was regulated (18%). AKT1 gene regulated by miR143 and miR125b, is in turn regulated by TFs, SMAD3 and STAT3. So, when AKT1 was not OE, its expression was inhibited by miR143 and mIR125b. When AKT1 was OE, it is no longer inhibited by miRNAs and interacts with DISC1, CCDC88A and ACTB thereby regulating migration.
Perturbation of each TF did not upregulate migration, as miRNA represses gene expression.
Perturbation of each miRNA did not upregulate migration, as other miRNAs in the functional module repressed TFs/genes expression.

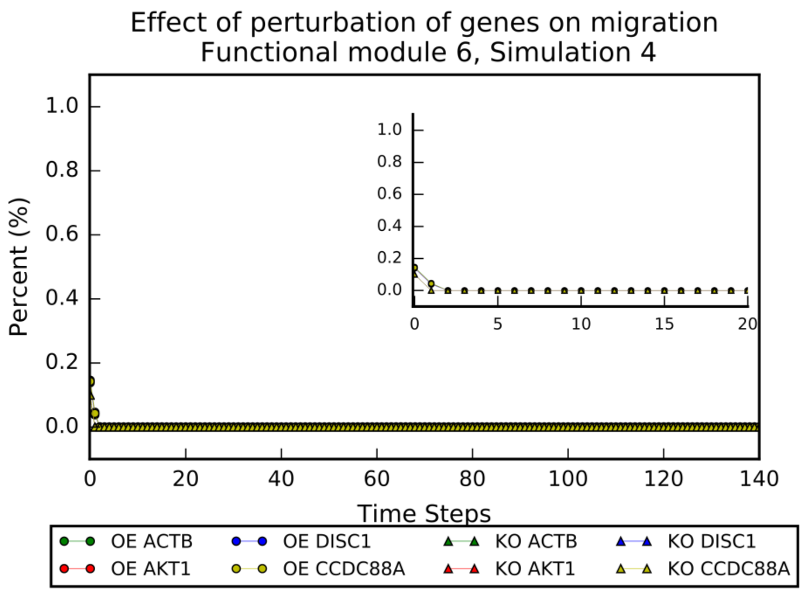
Effect of perturbation of genes on migration
Functional module 6, Simulation 4
Percent (%)
Time Steps
OE ACTB
OE AKT1
OE DISC1
OE CCDC88A
KO ACTB
KO AKT1
KO DISC1
KO CCDC88A

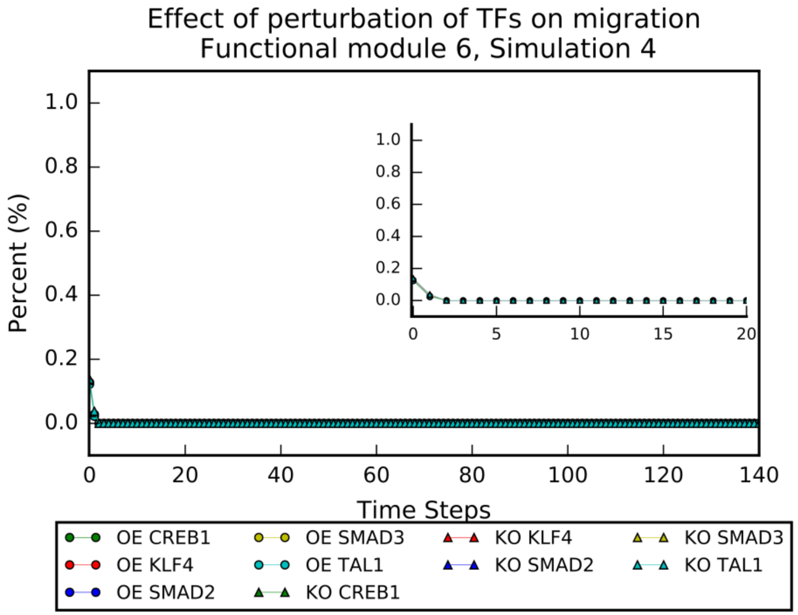
Effect of perturbation of TFs on migration
Functional module 6, Simulation 4
Percent (%)
Time Steps
OE CREB1
OE KLF4
OE SMAD2
OE SMAD3
OE TAL1
KO CREB1
KO KLF4
KO SMAD2
KO SMAD3
KO TAL1

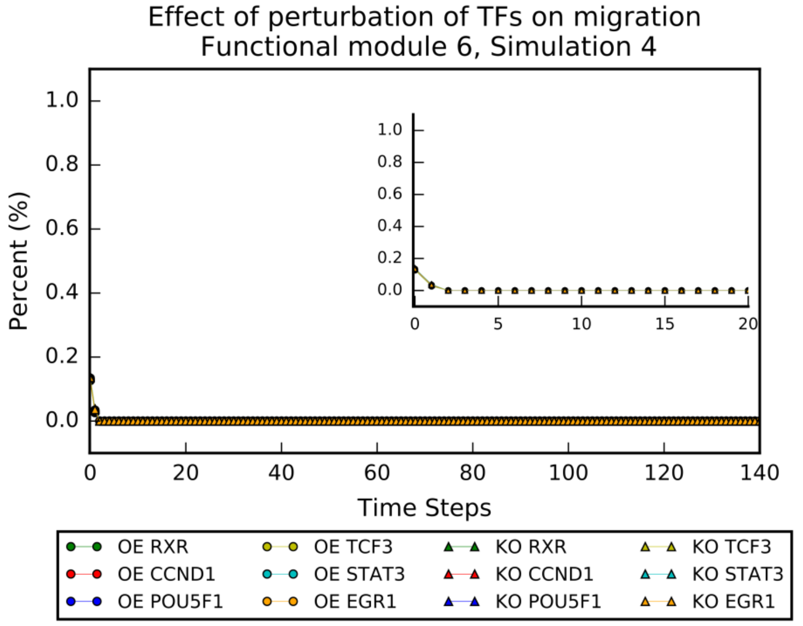
Effect of perturbation of TFs on migration
Functional module 6, Simulation 4
Percent (%)
Time Steps
OE RXR
OE CCND1
OE POU5F1
OE TCF3
OE STAT3
OE EGR1
KO RXR
KO CCND1
KO POU5F1
KO TCF3
KO STAT3
KO EGR1

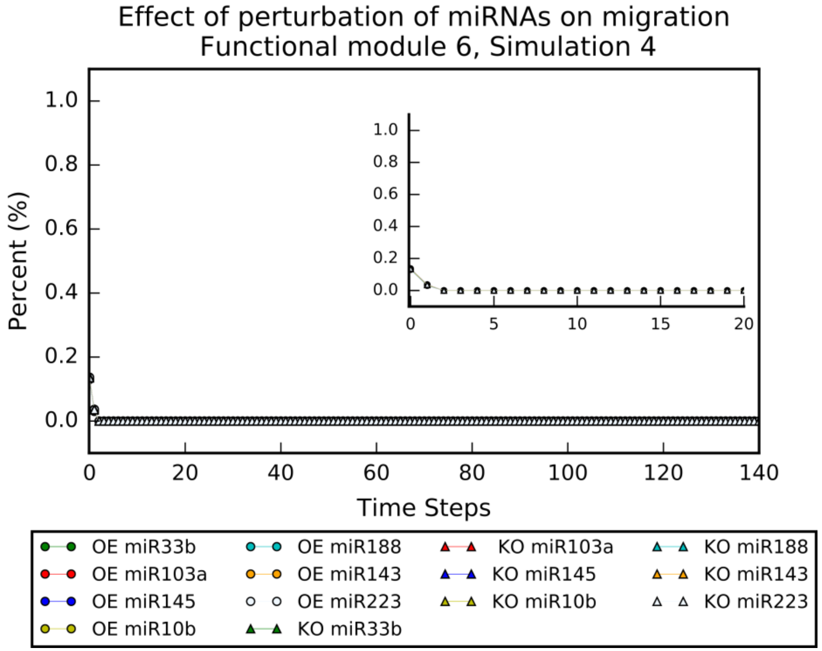
Effect of perturbation of miRNAs on migration
Functional module 6, Simulation 4
Percent (%)
Time Steps
OE miR33b
OE miR103a
OE miR145
OE miR10b
OE miR188
OE miR143
OE miR223
KO miR33b
KO miR103a
KO miR145
KO miR10b
KO miR188
KO miR143
KO miR223

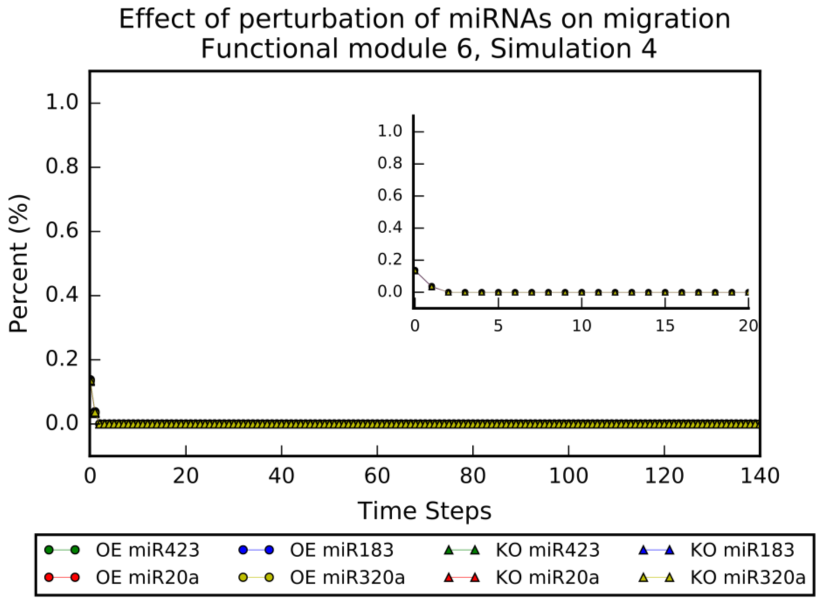

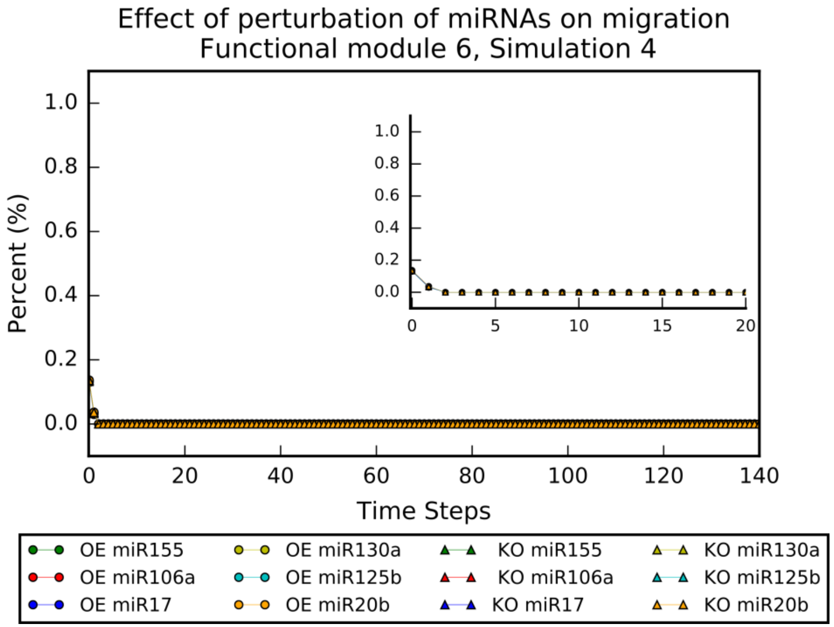

Supplementary Figure S26: Perturbation analysis FM6 Simulation 4

TF represses gene and activates miRNA expression. Perturbation of each gene did not upregulate migration as expression of all genes in FM6 is essential for migration.
Perturbation of each TF did not upregulate migration, as miRNA or TF represses gene expression.
Perturbation of each miRNA did not upregulate migration, as TF or miRNA represses gene expression.

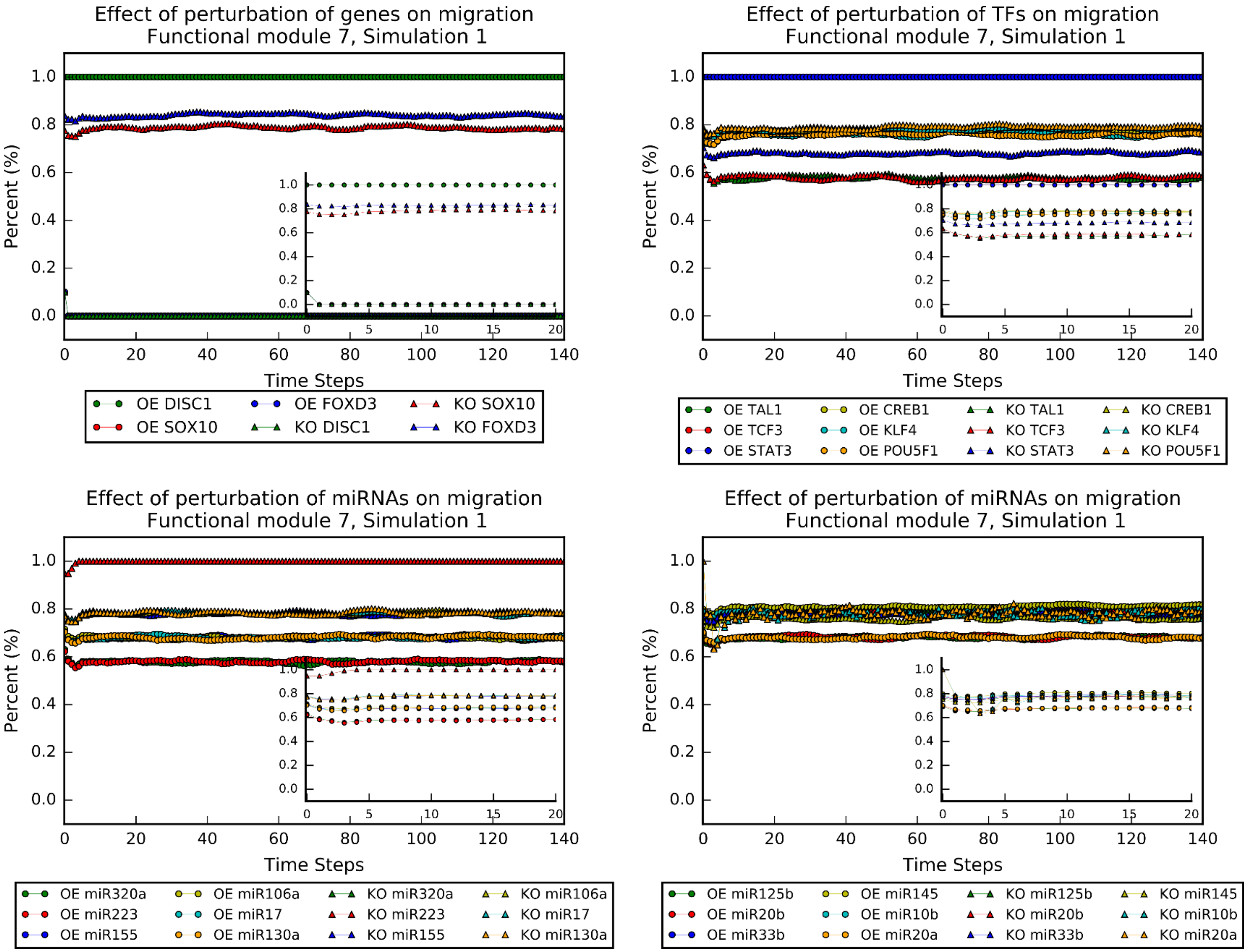

Supplementary Figure S27: Perturbation analysis FM7 Simulation 1

TF activates gene and miRNA expression. In FM7, when DISC1 gene was overexpressed (OE), migration was upregulated (100%), as DISC1 represses FOXD3 and SOX10 expression and upregulates migration. When DISC1 gene was knocked out (KO), migration was downregulated (0%), as SOX10 and FOXD3 expression downregulates migration. KO of SOX10 or FOXD3 regulated migration (78% by SOX10 and 83% by FOXD3), but migration was not upregulated, as repression of both SOX10 and FOXD3 by DISC1 upregulates migration.
Perturbation of each TF (except STAT3, TAL1 and TCF3 OE) did not upregulate migration, as miRNAs repressed gene expression. When TCF3 or TAL1 or STAT3 was OE, migration was upregulated as DISC1 gets expressed by one of these TFs. When CREB1 or KLF4 or POU5F1 was KO, migration was regulated (79%), as SOX10 and FOXD3 (which inhibits migration) was not expressed.
Perturbation of each miRNA (except for KO of miR223 or miR320a) did not upregulate migration, as other expressed miRNAs in the functional module repressed gene expression. When miR320a or miR223 was KO, migration was upregulated (100%), as DISC1 gene was expressed by TFs, TAL1 or TCF3.

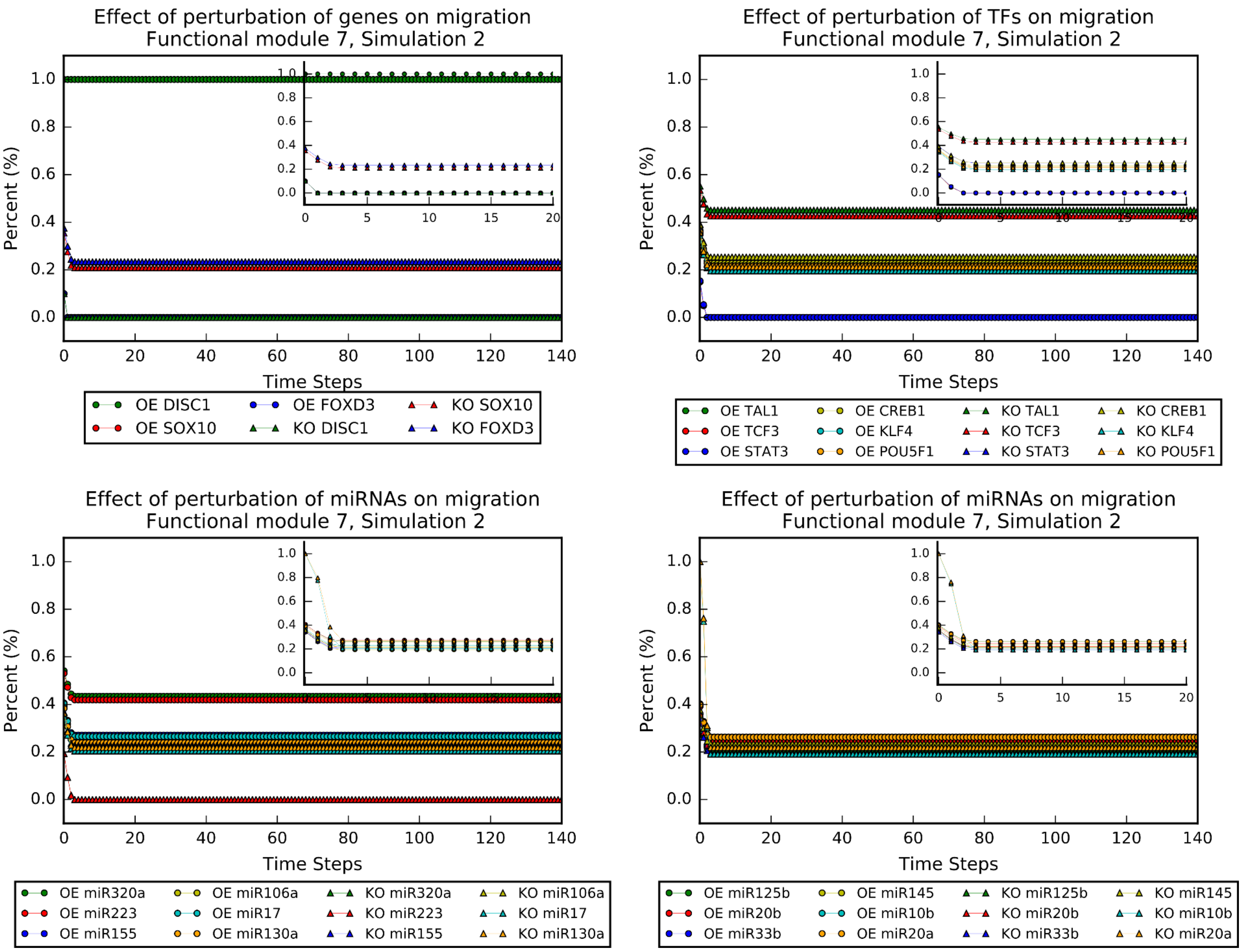

Supplementary Figure S28: Perturbation analysis FM7 Simulation 2

TF represses gene and miRNA expression. In FM7, when DISC1 gene was OE, migration was upregulated (100%), as DISC1 represses FOXD3 and SOX10 expression and upregulates migration. When DISC1 gene was KO, migration was downregulated (0%), as SOX10 and FOXD3 expression downregulates migration. KO of SOX10 or FOXD3 regulated migration (20% by SOX10, 23% by FOXD3), but migration was not upregulated, as repression of both FOXD3 and SOX10 expression by DISC1 is required for migration to be upregulated.

Perturbation of each TF did not upregulate migration, as miRNAs/TFs repress genes expression. When CREB1/KLF4/POU5F1 was OE, migration was regulated (20% by KLF4, 21% by POU5F1, 22% by CREB1) as SOX10 and FOXD3 genes were not expressed. When STAT3/TAL1/TCF3 was KO, migration was regulated (25% by STAT3, 42% by TCF3 and 45% by TAL1), as DISC1 gene gets expressed.

Perturbation of each miRNA did not upregulate migration, as miRNAs/TFs repress genes expression. When miR320a/miR223 was OE, migration was regulated (41% by miR223, 43% by miR320a), as it represses TCF3/TAL1 expression, activating DISC1 expression. When miR155/ miR106a/ miR17/ miR130a/ miR125b/ miR20a/ miR20b was KO, migration was regulated (20-26%) as STAT3 was expressed, in turn inhibits FOXD3 expression

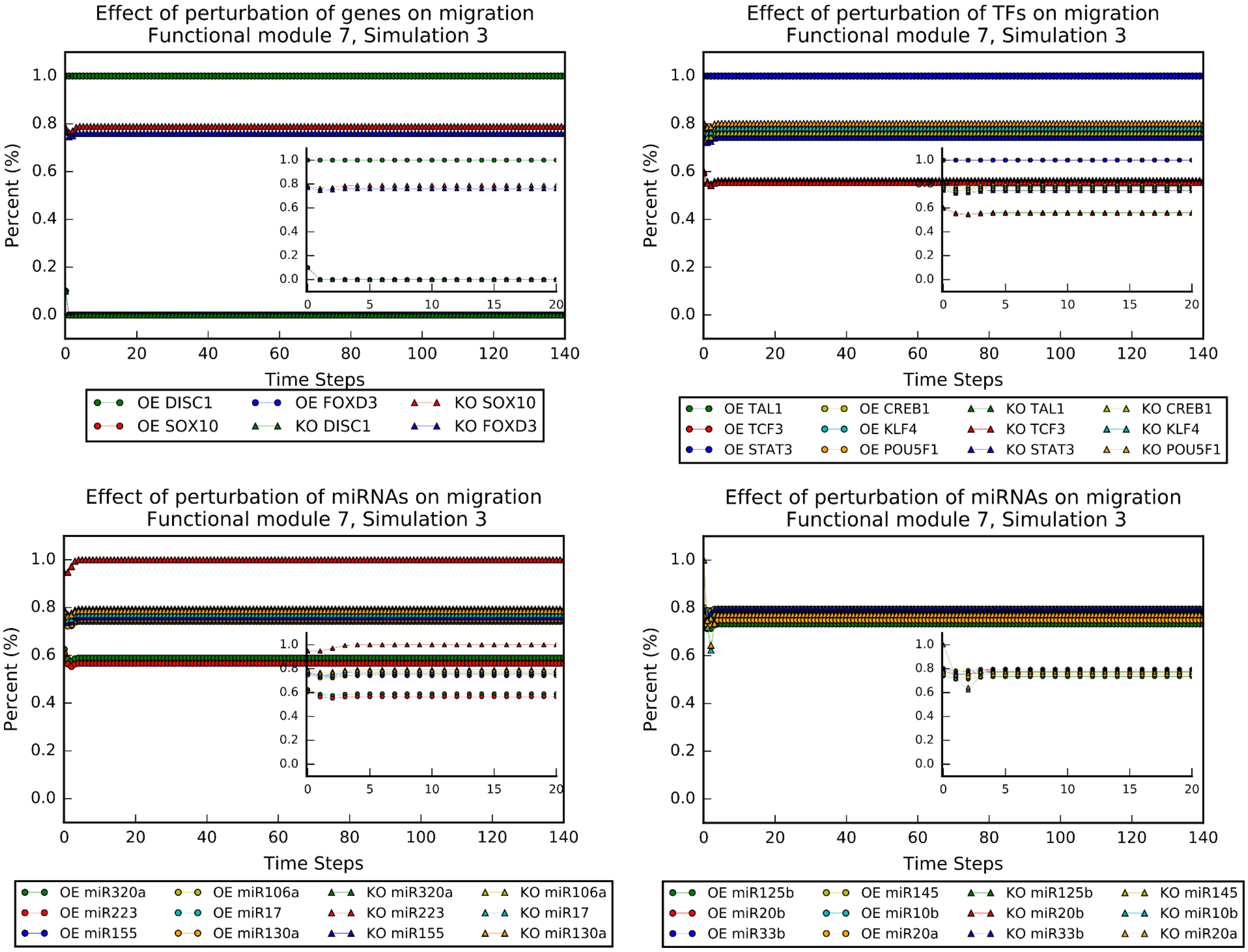

Supplementary Figure S29: Perturbation analysis FM7 Simulation 3

TF activates gene and represses miRNA expression. In FM7, when DISC1 gene was overexpressed (OE), migration was upregulated (100%), as DISC1 represses FOXD3 and SOX10 expression and upregulates migration. When DISC1 gene was knocked out (KO), migration was downregulated (0%), as SOX10 and FOXD3 expression downregulates migration. KO of SOX10 or FOXD3 regulated migration (78% by SOX10, 76% by FOXD3), but migration was not upregulated, as repression of both SOX10 and FOXD3 by DISC1 upregulates migration.
OE of TF, STAT3/TAL1/TCF3 upregulated migration (100%) as DISC1 is expressed. KO of each TF did not upregulate migration, as miRNAs repress gene expression. When CREB1 or KLF4 or POU5F1 was KO, migration was regulated (76% by CREB1, 78% by KLF4, 80% by POU5F1), as SOX10 and FOXD3 (which inhibits migration) was not expressed.
OE of each miRNA did not upregulate migration, as miRNA represses gene expression. KO of miR320a/miR223 showed upregulation of migration (100%), as TFs TAL1 /TCF3 activates DISC1 expression.

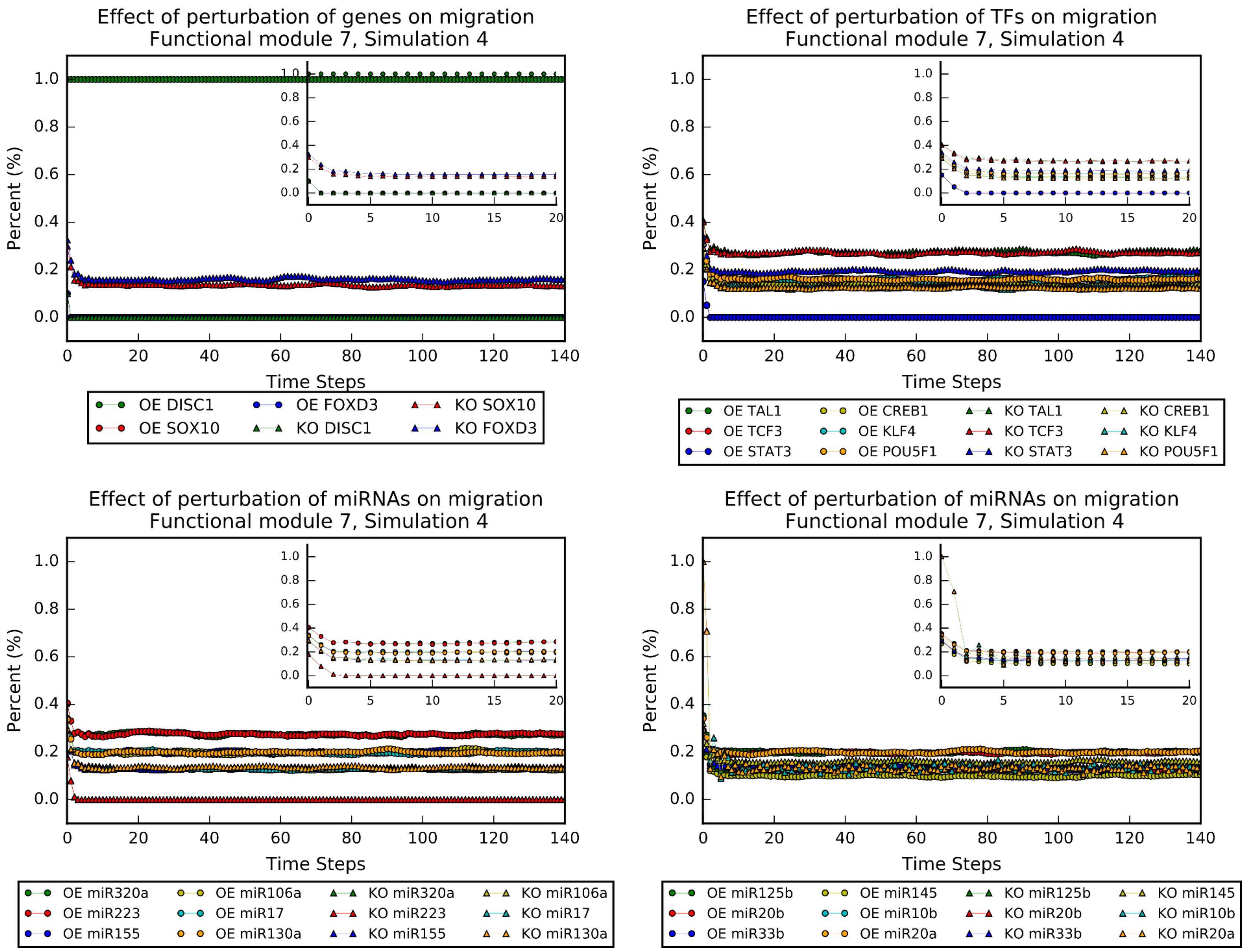

Supplementary Figure S30: Perturbation analysis FM7 Simulation 4

TF activates gene and represses miRNA expression. In FM7, when DISC1 gene was OE, migration was upregulated (100%), as DISC1 represses FOXD3 and SOX10 expression and upregulates migration. When DISC1 gene was KO, migration was downregulated (0%), as SOX10 and FOXD3 expression downregulates migration. KO of SOX10 or FOXD3 regulates migration (14% by SOX10, 15% by FOXD3), but migration was not upregulated, as repression of both SOX10 and FOXD3 by DISC1 upregulates migration.
Perturbation of each TF did not upregulate migration, as miRNAs/TFs repress gene expression. When CREB1/KLF4/POU5F1 was OE, migration was regulated (13% by CREB1, 16% by KLF4 and POU5F1) as expression of SOX10 and FOXD3 genes were downregulated. When STAT3/TAL1/TCF3 was KO, migration was regulated (19% by STAT3, 27% by TCF3, 28% by TAL1), as DISC1 gets expressed.
Perturbation of each miRNA did not upregulate migration, as miRNAs/TFs repress gene expression. When miR320a/miR223 was OE, migration was regulated (27%), as it repressed TCF3/TAL1 expression, activating DISC1 expression. When miR155/miR106a/miR17/miR130a/ miR125b/miR20a/miR20b was KO, migration was regulated (13-16%) as STAT3 (TF) gets expressed, that in turn inhibits FOXD3 gene expression.

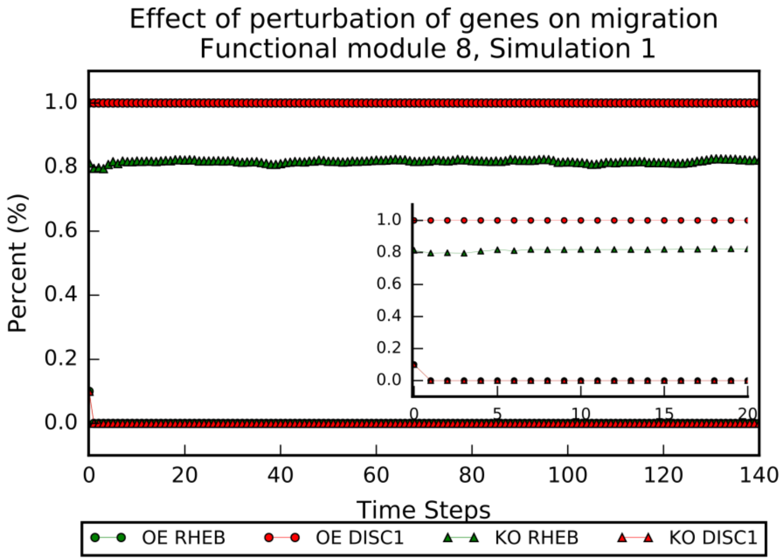
Effect of perturbation of genes on migration
Functional module 8, Simulation 1
Percent (%)
Time Steps
OE RHEB
OE DISC1
KO RHEB
KO DISC1

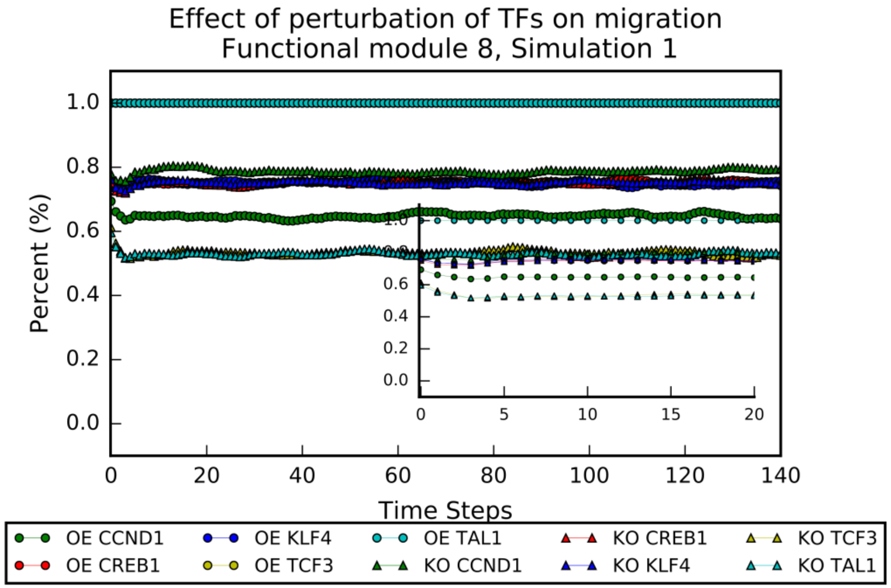
Effect of perturbation of TFs on migration
Functional module 8, Simulation 1
Percent (%)
Time Steps
OE CCND1
OE CREB1
OE KLF4
OE TCF3
OE TAL1
KO CCND1
KO CREB1
KO KLF4
KO TCF3
KO TAL1

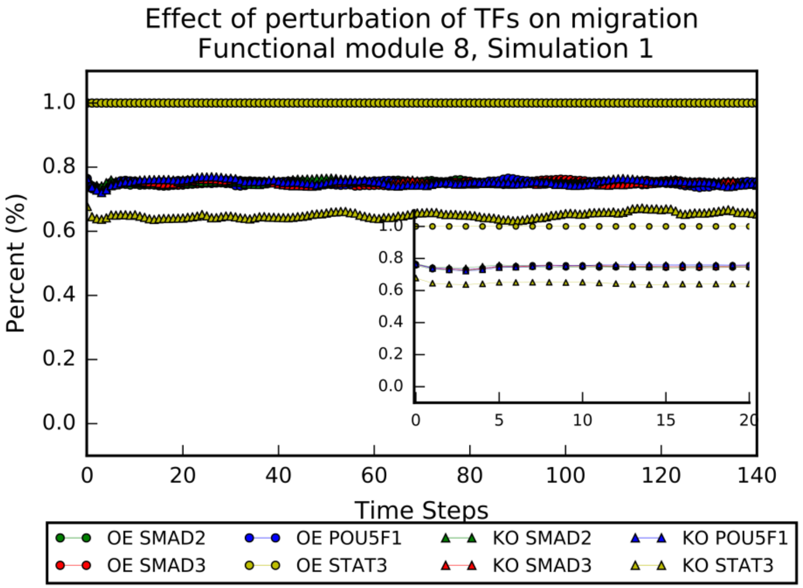
Effect of perturbation of TFs on migration
Functional module 8, Simulation 1
Percent (%)
Time Steps
OE SMAD2
OE SMAD3
OE POU5F1
OE STAT3
KO SMAD2
KO SMAD3
KO POU5F1
KO STAT3

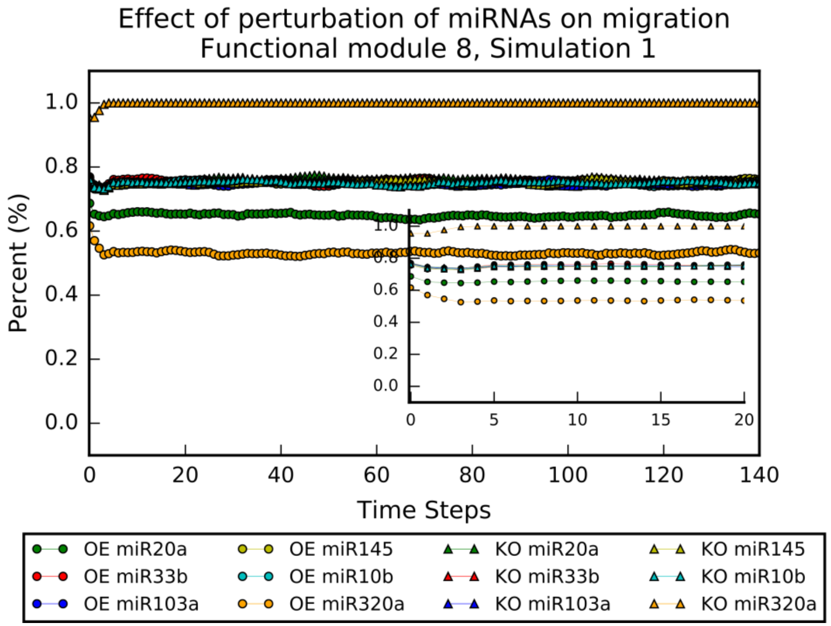
Effect of perturbation of miRNAs on migration
Functional module 8, Simulation 1
Percent (%)
Time Steps
OE miR20a
OE miR33b
OE miR103a
OE miR145
OE miR10b
OE miR320a
KO miR20a
KO miR33b
KO miR103a
KO miR145
KO miR10b
KO miR320a

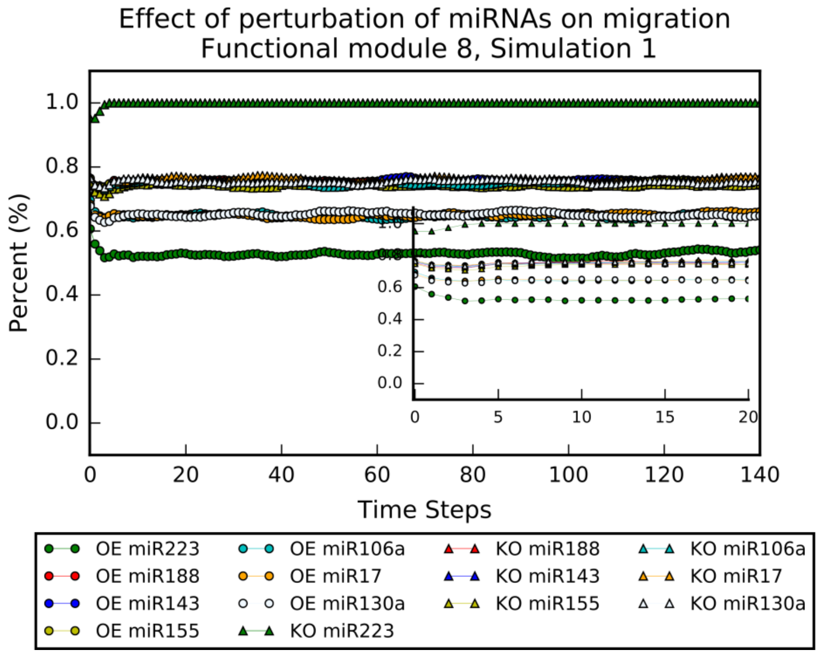

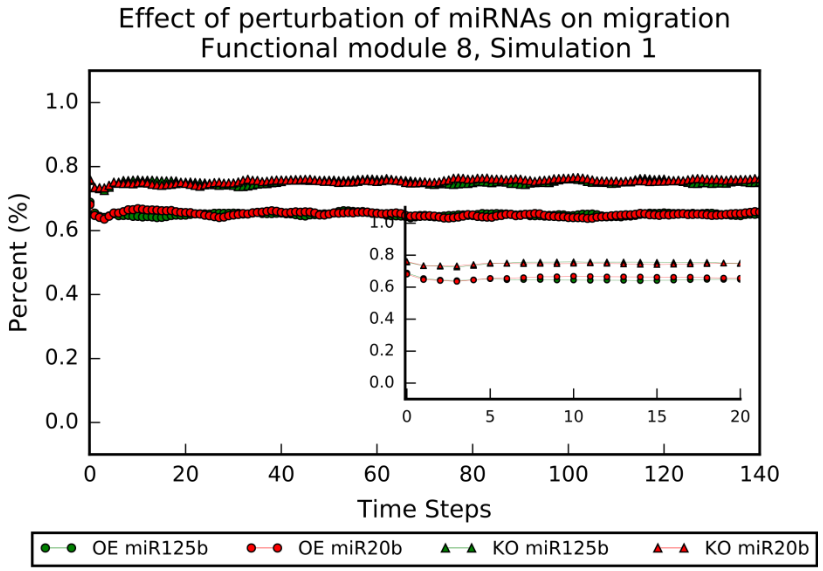

Supplementary Figure S31: Perturbation analysis FM8 Simulation 1

TF activates gene and miRNA expression. When DISC1 gene was overexpressed (OE), migration was upregulated (100%) as DISC1 represses RHEB and upregulates migration. When RHEB gene was KO, migration was regulated between 81-82%, but migration was not upregulated as DISC1 was inhibited by miRNAs (through TFs).
Perturbation of each TF (except for over expression of STAT3, TCF3 and TAL1) did not upregulate migration, as miRNA represses gene expression. OE of TCF3 or TAL1 or STAT3 showed upregulation of migration, as these TFs upregulates DISC1 expression. When SMAD3 or SMAD3 or POU5F1 was KO, RHEB expression was downregulated and migration was regulated (74% by SMAD2 and by POU5F1, 75% by SMAD3). Here migration was not upregulated, as RHEB was upregulated by the other expressed TFs in the module.
Perturbation of each miRNA (except for knock out of miR320a and miR223), did not upregulate migration, as miRNA represses TF/gene expression. KO of miR320/miR223 upregulated migration, as TFs TAL1 and TCF3 activated DISC1 expression. When each miRNA regulating STAT3 expression was KO, migration was not upregulated, as STAT3 (regulating DISC1 expression) gets repressed by more than one miRNA.

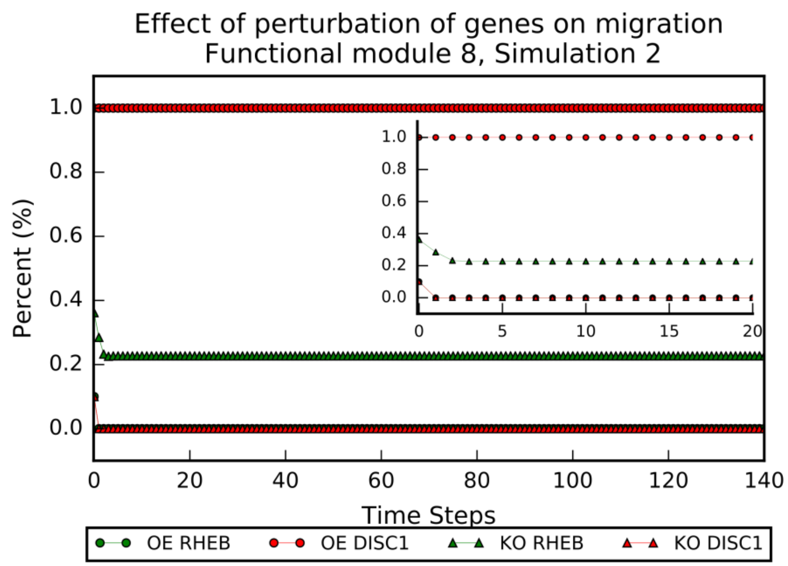
Effect of perturbation of genes on migration
Functional module 8, Simulation 2
Percent (%)
Time Steps
OE RHEB
OE DISC1
KO RHEB
KO DISC1

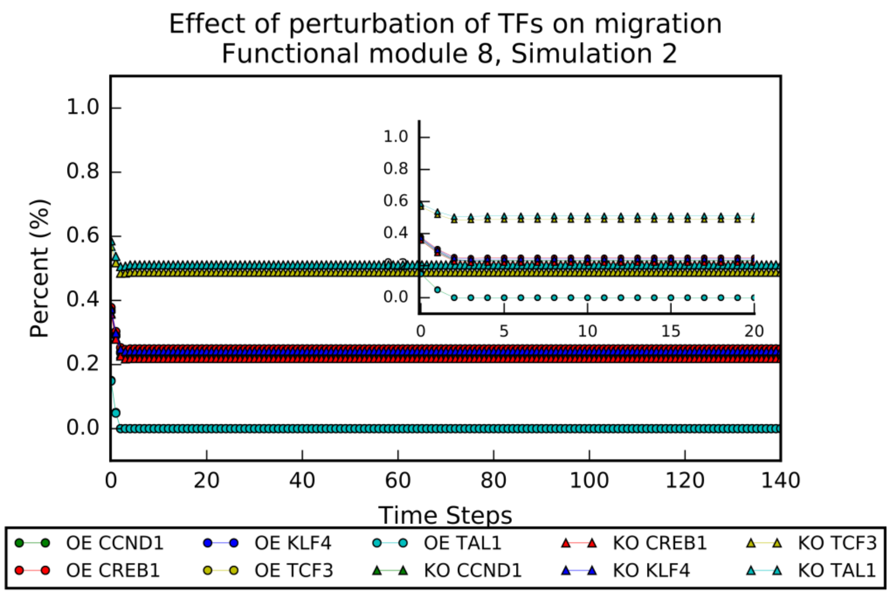
Effect of perturbation of TFs on migration
Functional module 8, Simulation 2
Percent (%)
Time Steps
OE CCND1
OE CREB1
OE KLF4
OE TCF3
OE TAL1
KO CCND1
KO CREB1
KO KLF4
KO TCF3
KO TAL1

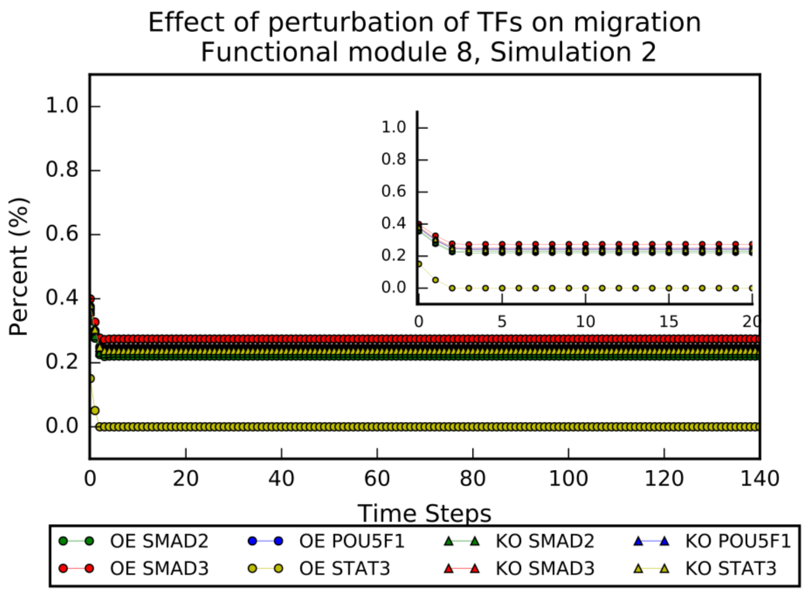
Effect of perturbation of TFs on migration
Functional module 8, Simulation 2
Percent (%)
Time Steps
OE SMAD2
OE SMAD3
OE POU5F1
OE STAT3
KO SMAD2
KO SMAD3
KO POU5F1
KO STAT3

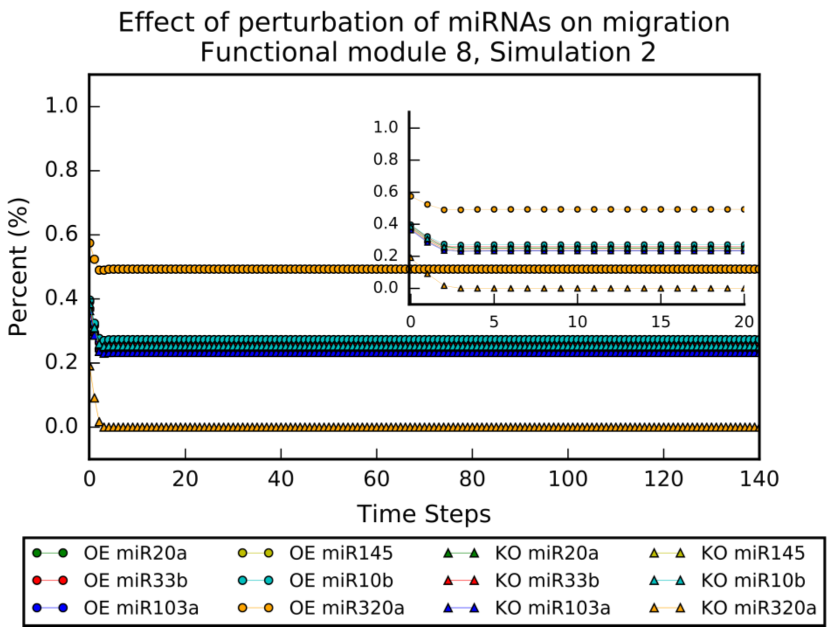
Effect of perturbation of miRNAs on migration
Functional module 8, Simulation 2
Percent (%)
Time Steps
OE miR20a
OE miR33b
OE miR103a
OE miR145
OE miR10b
OE miR320a
KO miR20a
KO miR33b
KO miR103a
KO miR145
KO miR10b
KO miR320a

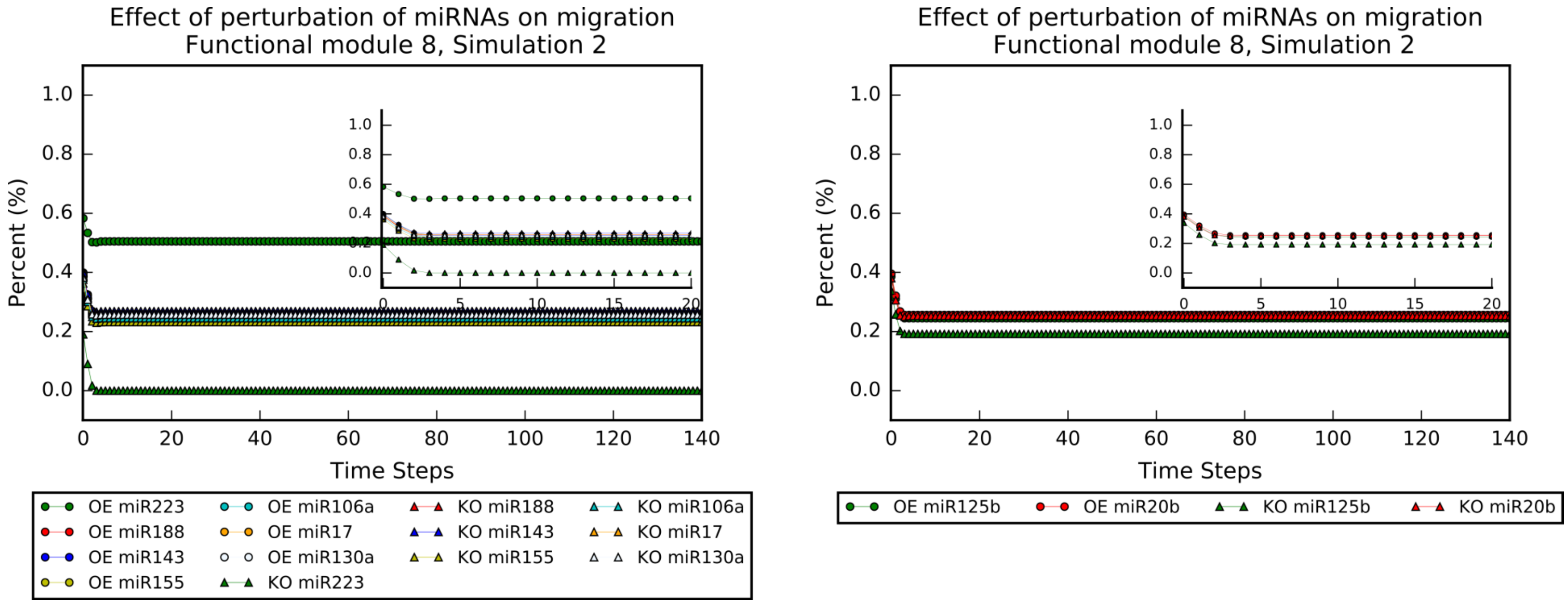

Supplementary Figure S32: Perturbation analysis FM8 Simulation 2

TF represses gene and miRNA expression. When DISC1 gene was overexpressed (OE), migration was upregulated (100%) as DISC1 represses RHEB and upregulates migration. When RHEB gene was KO, migration was regulated (22%), but migration was not upregulated as DISC1 was inhibited by miRNAs (through TFs).

Perturbation of each TF did not upregulate migration, as miRNA/TF represses gene expression. When TCF3 or TAL1 or STAT3 was OE, migration was downregulated (0%) as these TFs inhibit DISC1 expression. When CCND1 or CREB1 or KLF4 downregulating RHEB expression was OE, migration was not upregulated as DISC1 was repressed by other TFs in the module.

Perturbation of each miRNA did not upregulate migration, as miRNA/TF represses gene expression. When miR320a/miR223 was OE, migration was regulated (49% by miR320a, 50% by miR223), as TFs TCF3/TAL1 which inhibits DISC1 expression gets repressed. KO of miR223/miR320a showed downregulation of migration (0%), as TAL1/TCF3 downregulating DISC1 expression gets expressed.

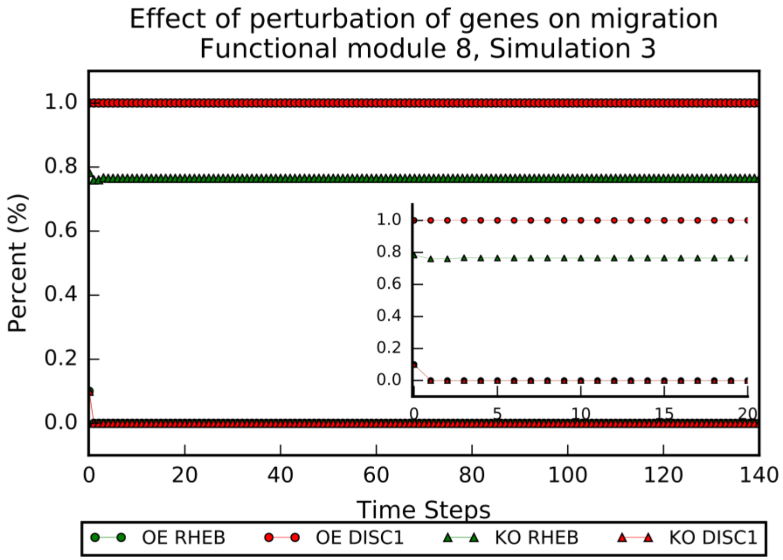
Effect of perturbation of genes on migration
Functional module 8, Simulation 3
Percent (%)
Time Steps
OE RHEB
OE DISC1
KO RHEB
KO DISC1

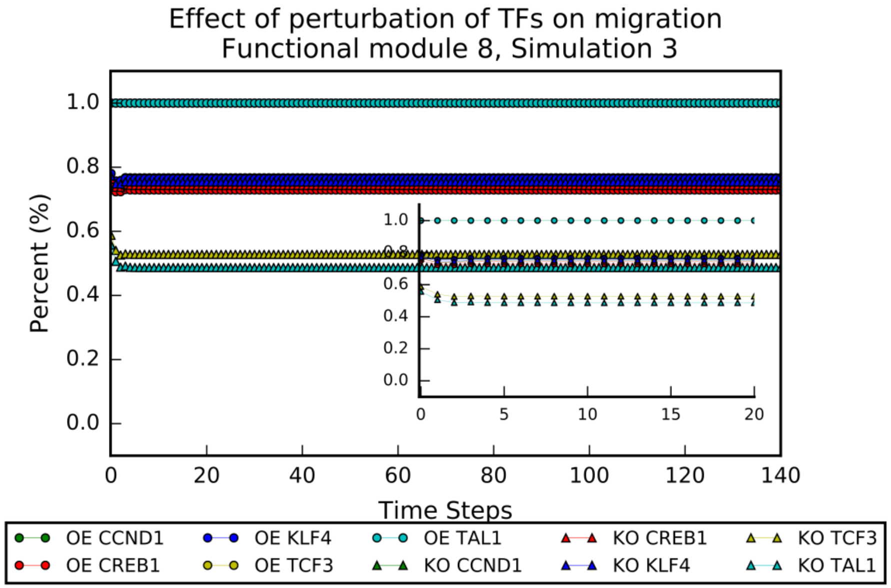
Effect of perturbation of TFs on migration
Functional module 8, Simulation 3
Percent (%)
Time Steps
OE CCND1
OE CREB1
OE KLF4
OE TCF3
OE TAL1
KO CCND1
KO CREB1
KO KLF4
KO TCF3
KO TAL1

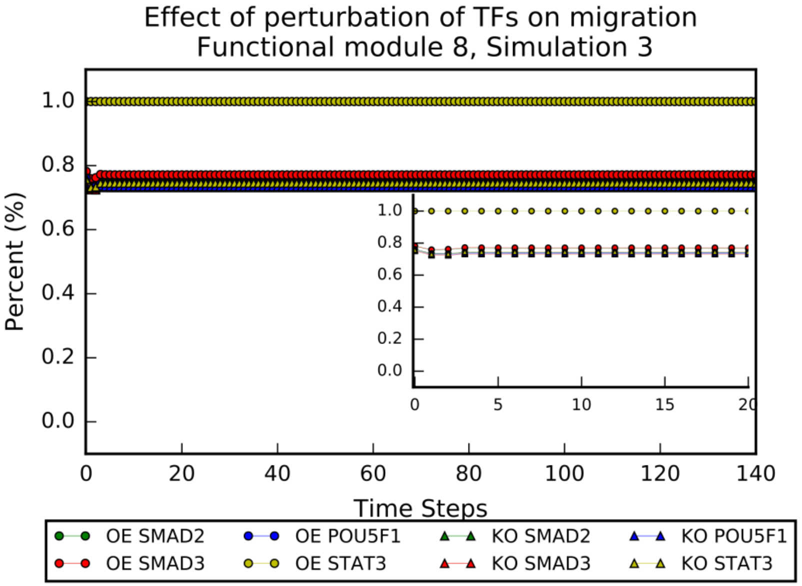
Effect of perturbation of TFs on migration
Functional module 8, Simulation 3
Percent (%)
Time Steps
OE SMAD2
OE SMAD3
OE POU5F1
OE STAT3
KO SMAD2
KO SMAD3
KO POU5F1
KO STAT3

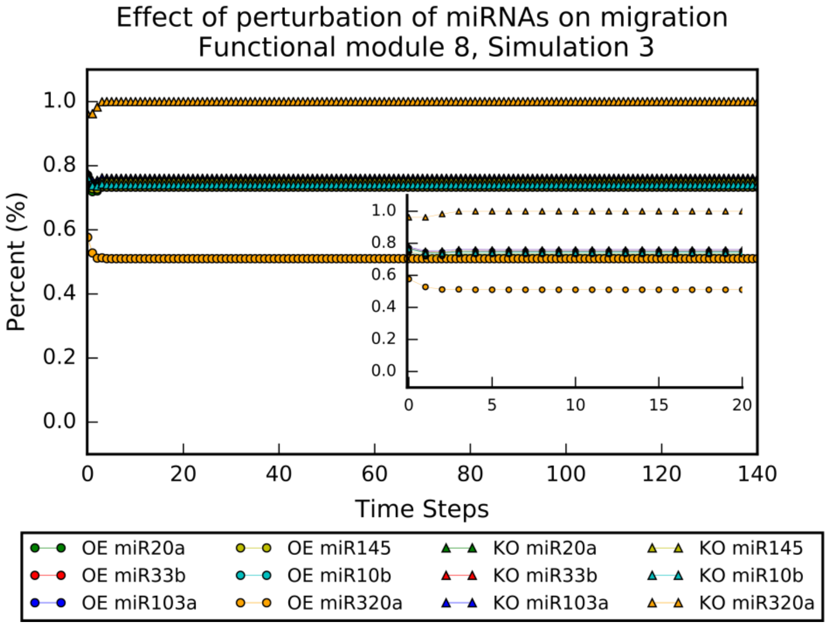
Effect of perturbation of miRNAs on migration
Functional module 8, Simulation 3
Percent (%)
Time Steps
OE miR20a
OE miR33b
OE miR103a
OE miR145
OE miR10b
OE miR320a
KO miR20a
KO miR33b
KO miR103a
KO miR145
KO miR10b
KO miR320a

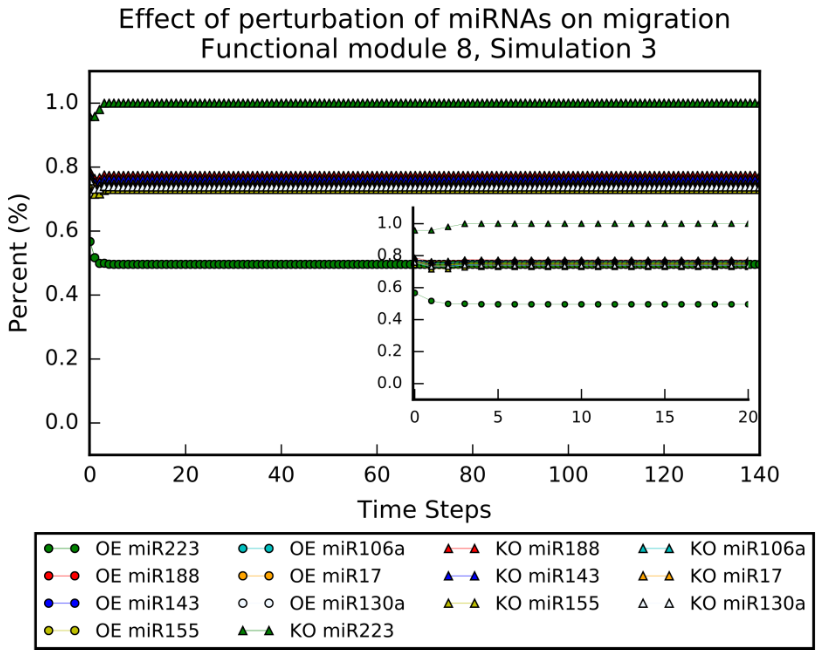

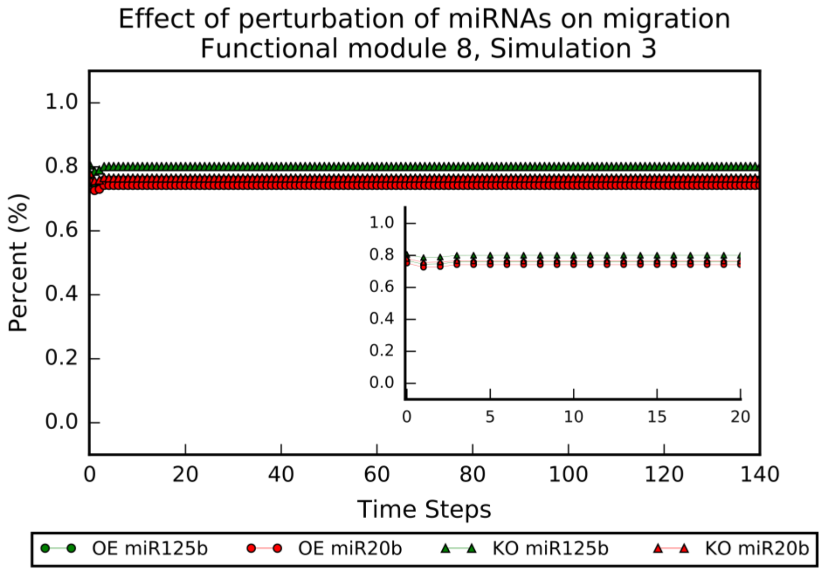

Supplementary Figure S33: Perturbation analysis FM8 Simulation 3

TF represses miRNA and activates gene expression. When DISC1 gene was overexpressed (OE), migration was upregulated (100%) as DISC1 repressed RHEB and upregulated migration. When RHEB gene was KO, migration was regulated (76%), but migration was not upregulated as DISC1 was inhibited by miRNAs (through TFs).
Perturbation of each TF (except for overexpression of STAT3, TCF3 and TAL1) did not upregulate migration, as miRNAs repressed gene expression. OE of TCF3 or TAL1 or STAT3 showed upregulation of migration, as these TFs upregulated DISC1 expression. When SMAD3 or SMAD3 or POU5F1 was KO, RHEB expression was downregulated, regulating migration (73%). Here migration was not upregulated, as RHEB expression was upregulated by expressed TFs in the functional module.
Perturbation of each miRNA (except for knock out of miR320a and miR223), did not upregulate migration, as miRNA repressed TF/gene expression. KO of miR320/miR223 upregulated migration, as TFs TAL1 and TCF3 activated DISC1 expression. When each miRNA regulating STAT3 expression was KO, migration was not upregulated, as STAT3 (regulating DISC1 expression) gets repressed by more than one miRNA.

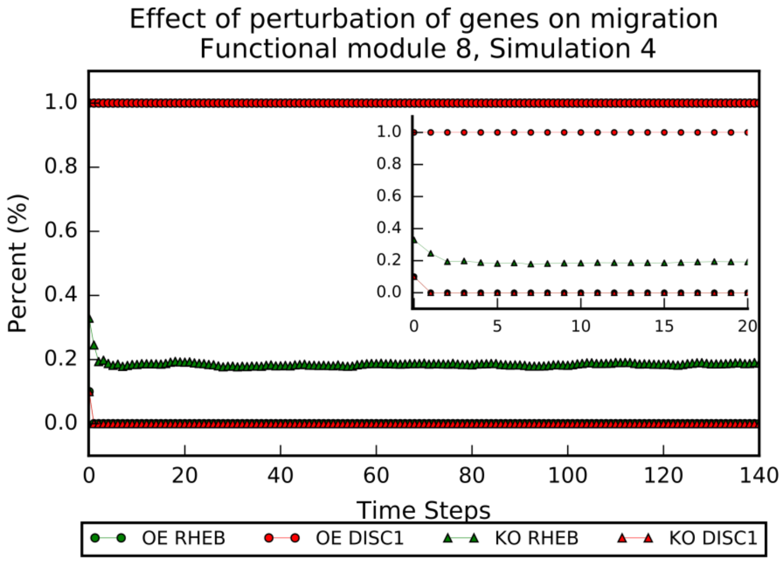
Effect of perturbation of genes on migration
Functional module 8, Simulation 4
Percent (%)
Time Steps
OE RHEB
OE DISC1
KO RHEB
KO DISC1

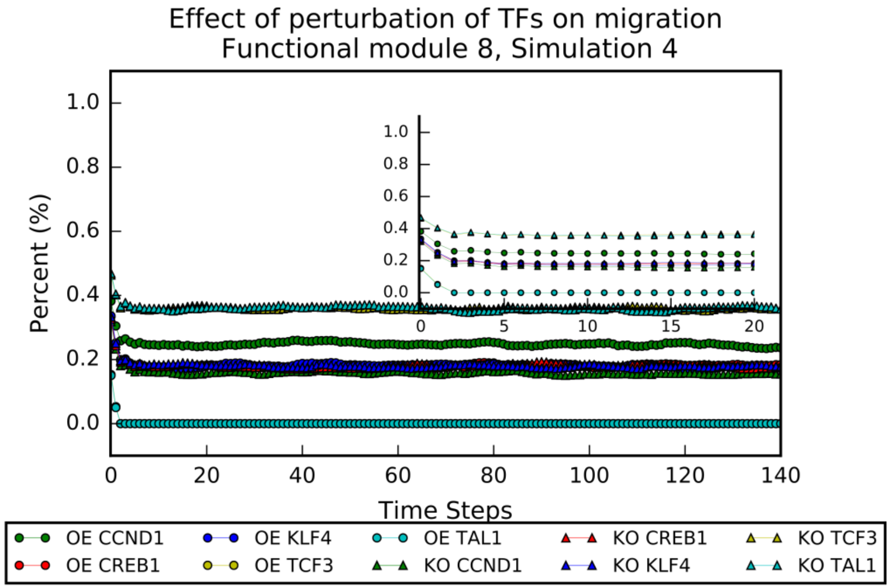
Effect of perturbation of TFs on migration
Functional module 8, Simulation 4
Percent (%)
Time Steps
OE CCND1
OE CREB1
OE KLF4
OE TCF3
OE TAL1
KO CCND1
KO CREB1
KO KLF4
KO TCF3
KO TAL1

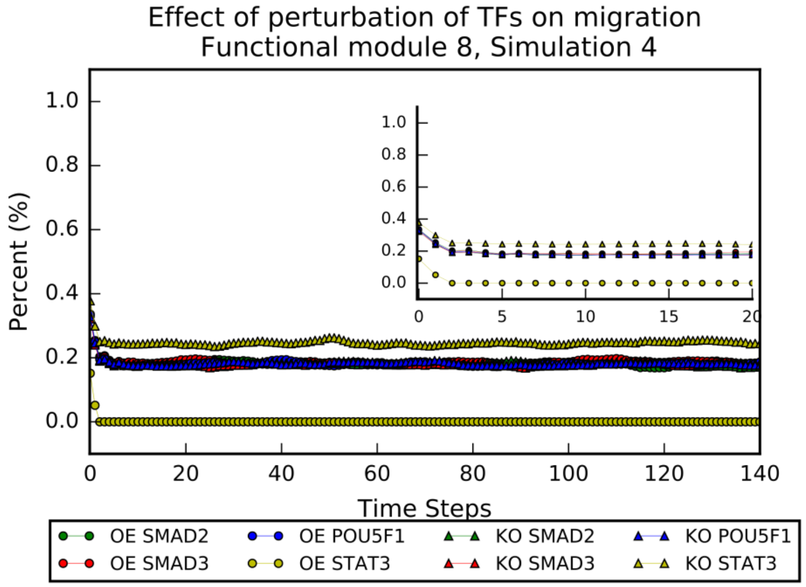
Effect of perturbation of TFs on migration
Functional module 8, Simulation 4
Percent (%)
Time Steps
OE SMAD2
OE SMAD3
OE POU5F1
OE STAT3
KO SMAD2
KO SMAD3
KO POU5F1
KO STAT3

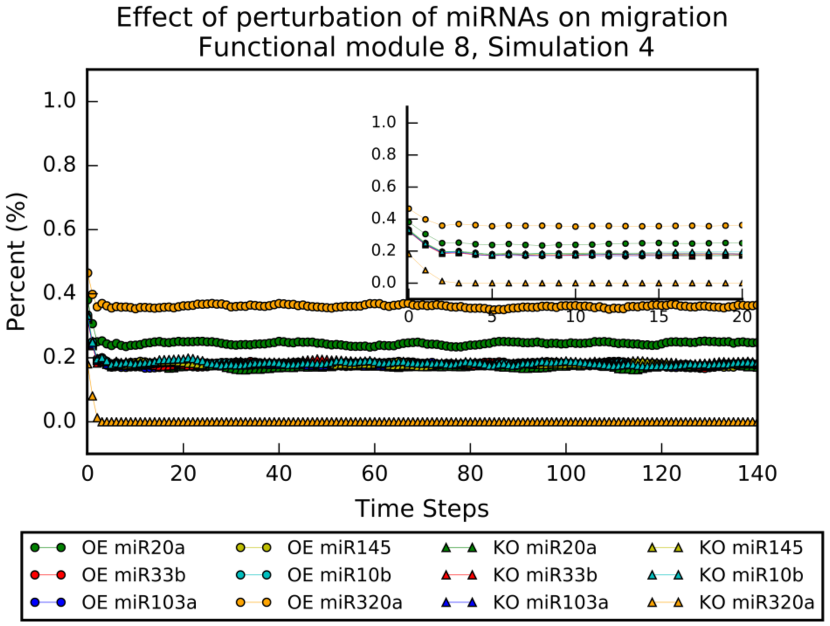
Effect of perturbation of miRNAs on migration
Functional module 8, Simulation 4
Percent (%)
Time Steps
OE miR20a
OE miR33b
OE miR103a
OE miR145
OE miR10b
OE miR320a
KO miR20a
KO miR33b
KO miR103a
KO miR145
KO miR10b
KO miR320a

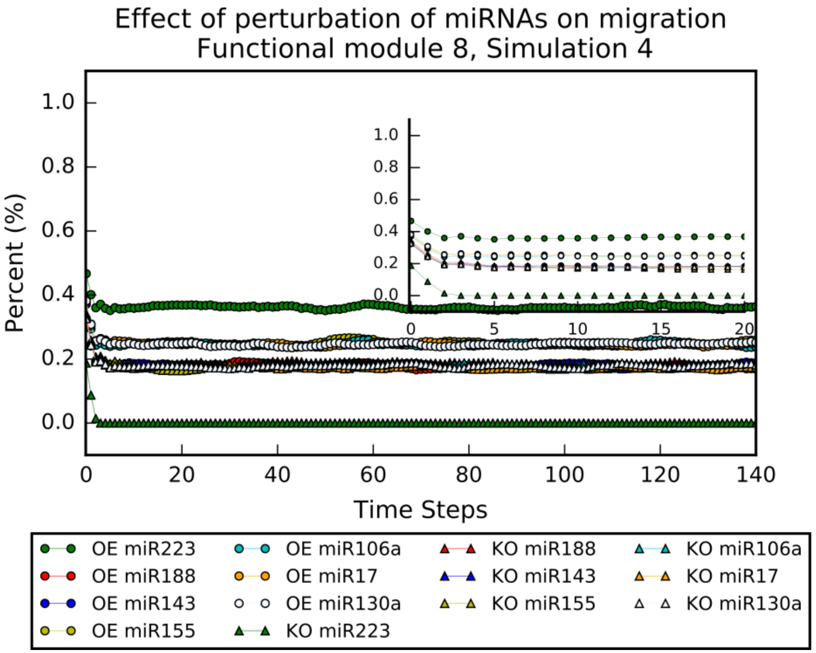

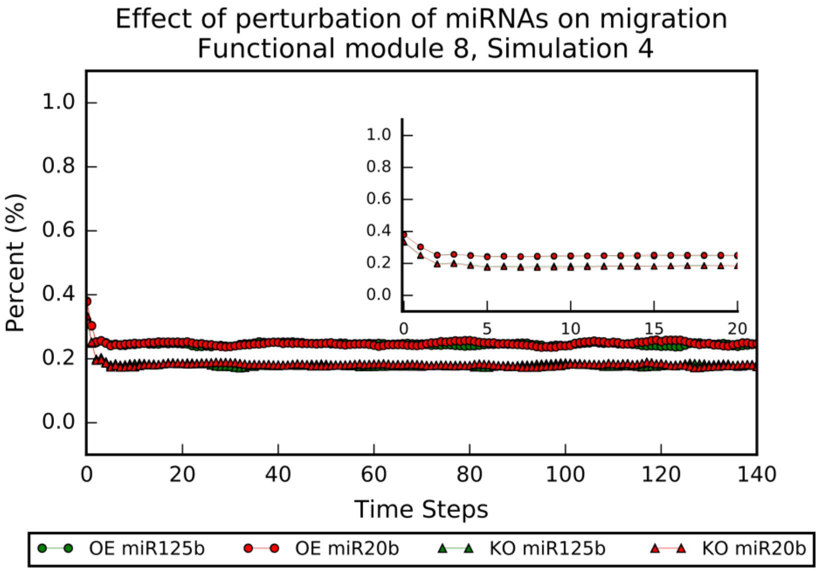

Supplementary Figure S34: Perturbation analysis FM8 Simulation 4

TF represses miRNA and gene expression. When DISC1 gene was overexpressed (OE), migration was upregulated (100%) as DISC1 represses RHEB and upregulates migration. When RHEB gene was KO, migration was regulated (18%), but migration was not upregulated as miRNAs inhibits DISC1 expression.
Perturbation of each TF did not upregulate migration, as miRNA/TF repressed gene expression. When TCF3 or TAL1 or STAT3 was OE, migration was downregulated (0%) as these TFs inhibited DISC1 expression. When CCND1 or CREB1 or KLF4 downregulating RHEB expression was OE, migration was not upregulated as DISC1 was repressed by other TFs in the module.
Perturbation of each miRNA did not upregulate migration, as miRNAs/TFs repressed DISC1 expression. When miR320a/miR223 was OE, migration was regulated (36%), as TFs TCF3/TAL1 which inhibits DISC1 expression was repressed. KO of miR223/miR320a showed downregulation of migration (0%), as TAL1/TCF3 downregulating DISC1 expression was expressed.

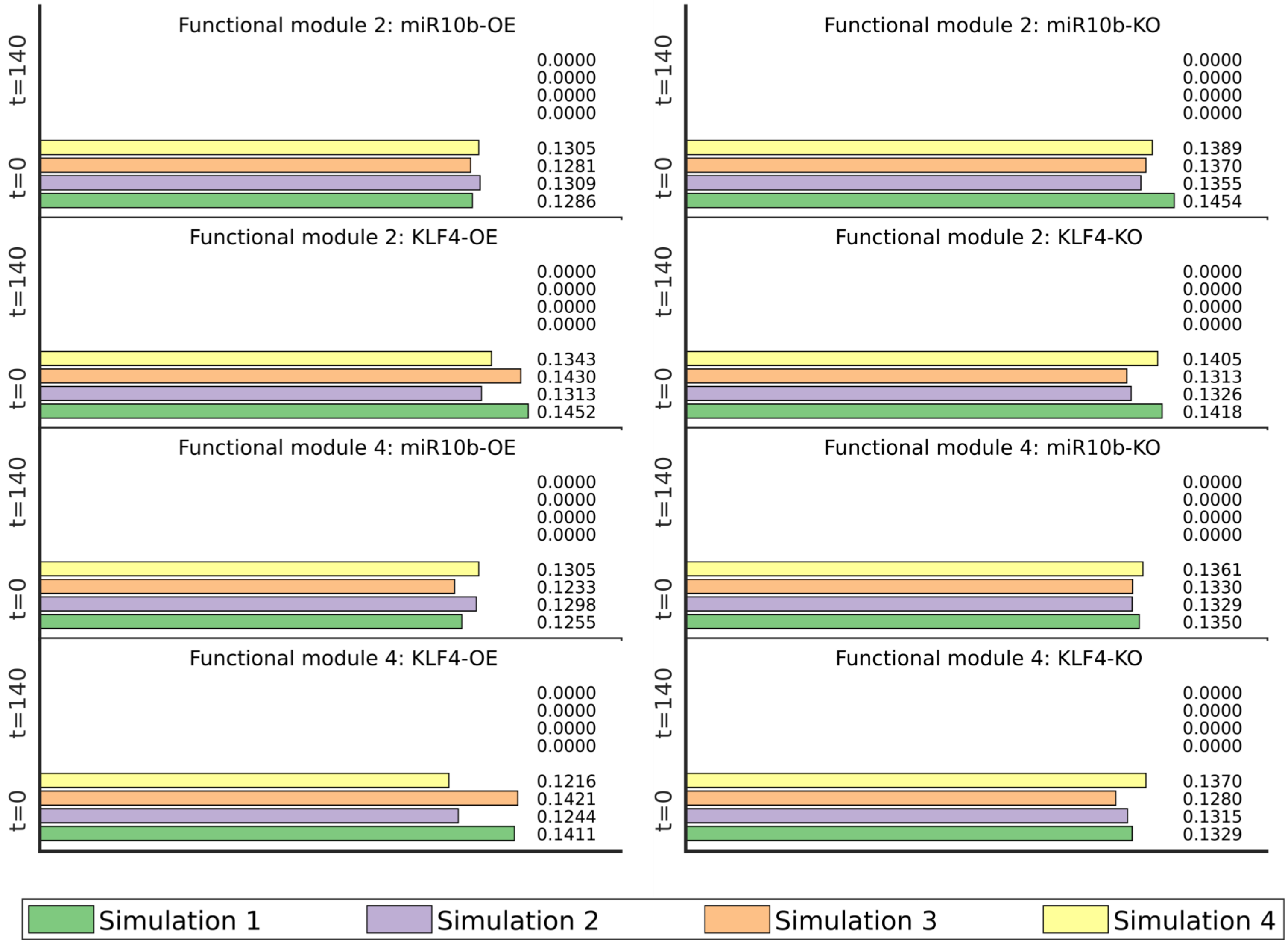

Supplementary Figure S35: Perturbation results of miR10b and KLF4 in feedback loop regulating functional modules 2 and 4

Perturbations were carried out for four different simulations i.e. when TF upregulates gene and miRNA expression (Simulation 1), when TF downregulates gene and miRNA expression (Simulation 2), when TF downregulates miRNA expression and upregulates gene expression (Simulation 3) and when TF upregulates miRNA and downregulates gene expression (Simulation 4). In all 4 simulations at *t*=140, overexpression (OE) /knock out (KO) of miR10b and KLF4 in both functional module 2 and functional module 4, showed downregulation of migration (0%).

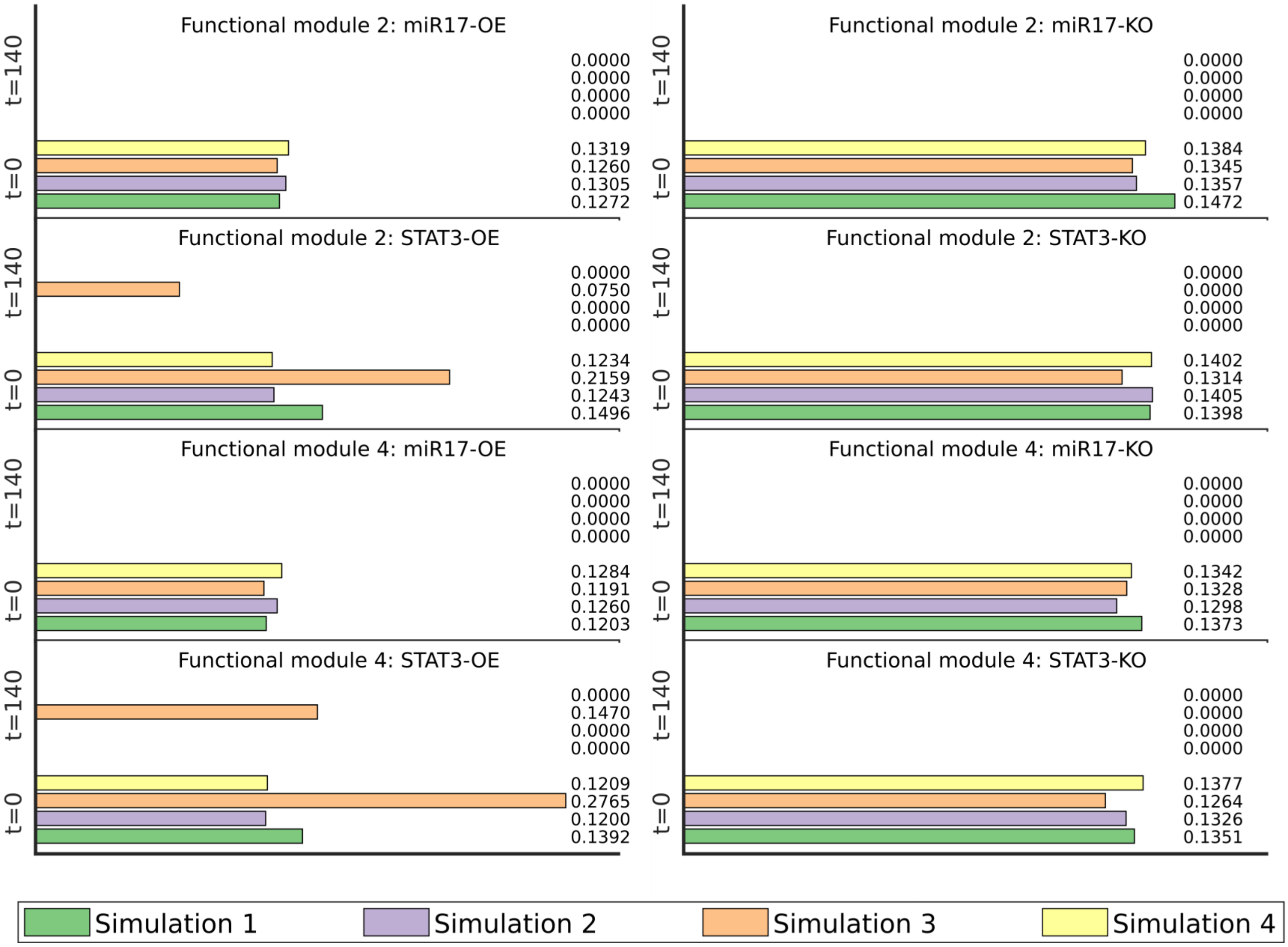

Supplementary Figure S36: Perturbation results of miR17 and STAT3 in feedback loop regulating functional modules 2 and 4

Perturbations were carried out for four different simulations i.e. when TF upregulates gene and miRNA expression (Simulation 1), when TF downregulates gene and miRNA expression (Simulation 2), when TF downregulates miRNA expression and upregulates gene expression (Simulation 3) and when TF upregulates miRNA and downregulates gene expression (Simulation 4). At all 4 simulations, overexpression (OE) /knock out (KO) of miR17 and KO of STAT3 in both functional module 2 and functional module 4, showed downregulation of migration (0%) at t=140. At simulation 1, 3 and 4, OE of STAT3 showed downregulation of migration in functional modules 2 and 4 at t=140. At simulation 3, OE of STAT3, showed regulation of migration in both functional modules 2 and 4 (7% in functional module 2; 14% in functional module 4) at t=140

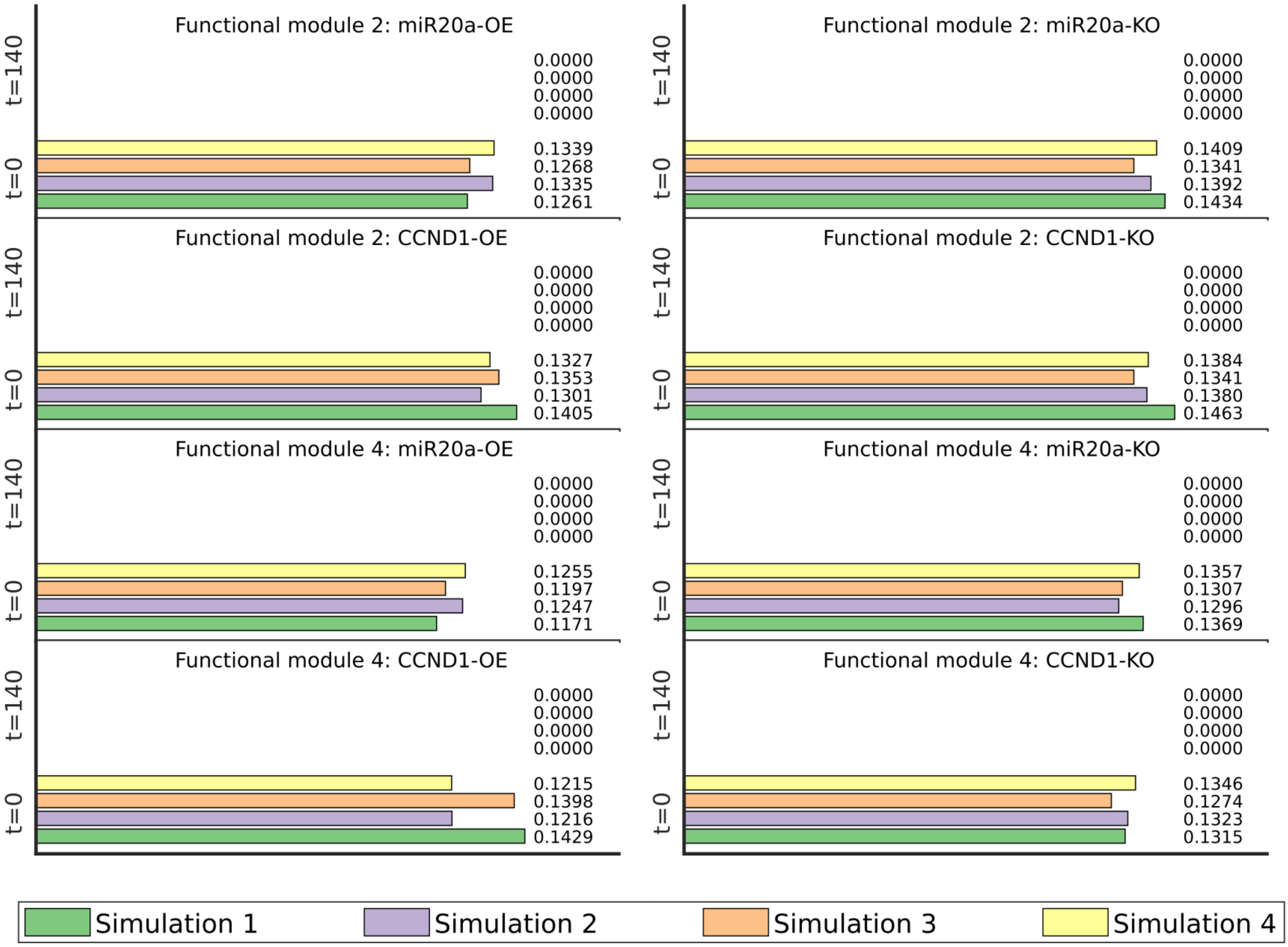

Supplementary Figure S37: Perturbation results of miR20a and CCND1 in feedback loop regulating functional modules 2 and 4

Perturbations were carried out for four different simulations i.e. when TF upregulates gene and miRNA expression (Simulation 1), when TF downregulates gene and miRNA expression (Simulation 2), when TF downregulates miRNA expression and upregulates gene expression (Simulation 3) and when TF upregulates miRNA and downregulates gene expression (Simulation 4). In all 4 simulations at *t*=140, overexpression (OE) /knock out (KO) of miR20a and CCND1 in both functional module 2 and functional module 4, showed downregulation of migration (0%).

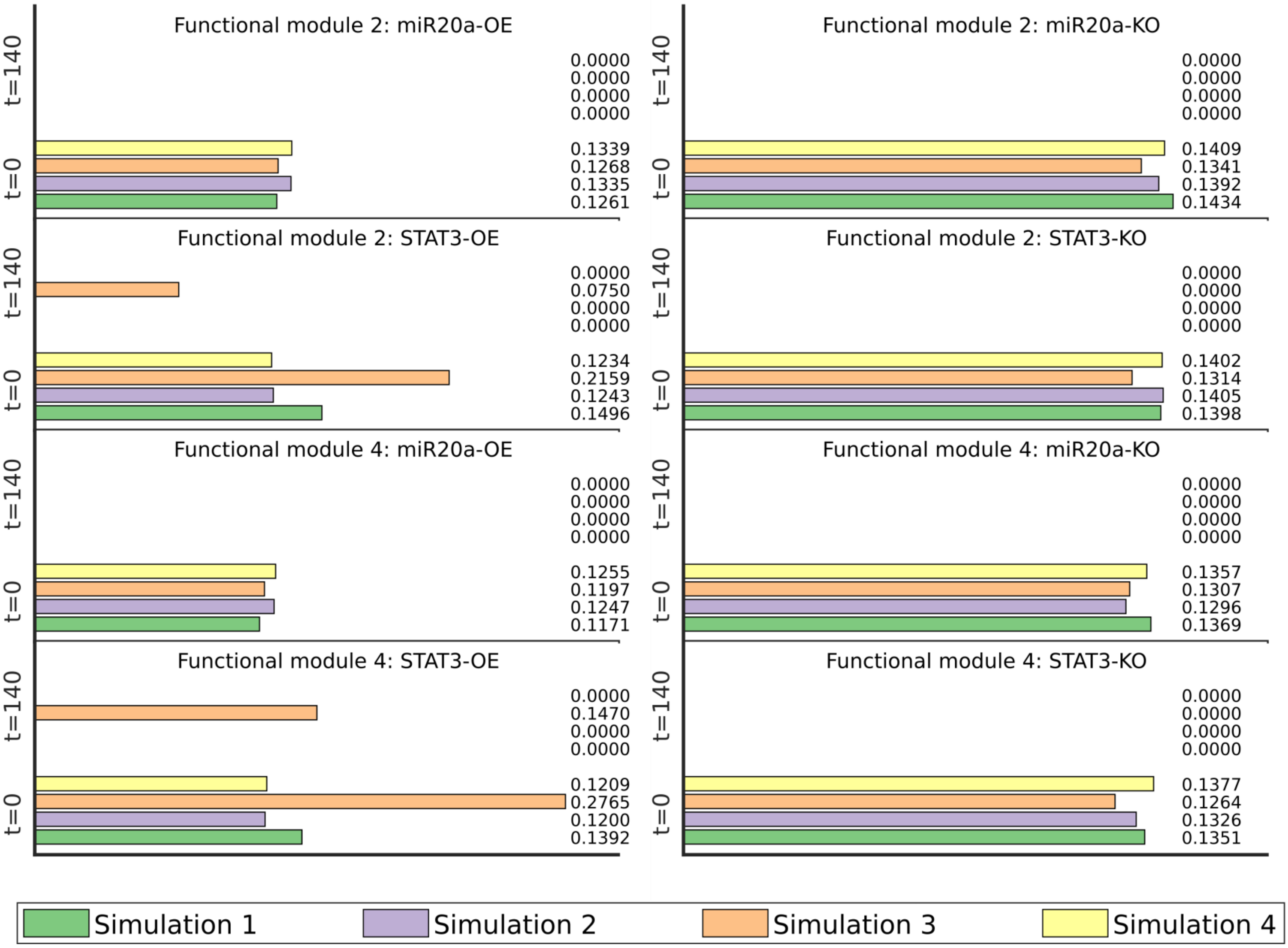

Supplementary Figure S38: Perturbation results of miR20a and STAT3 in feedback loop regulating functional modules 2 and 4

Perturbations were carried out for four different simulations i.e. when TF upregulates gene and miRNA expression (Simulation 1), when TF downregulates gene and miRNA expression (Simulation 2), when TF downregulates miRNA expression and upregulates gene expression (Simulation 3) and when TF upregulates miRNA and downregulates gene expression (Simulation 4). In all 4 simulations at *t*=140, overexpression (OE) /knock out (KO) of miR20a and KO of STAT3 in both functional module 2 and functional module 4, showed downregulation of migration (0%). In simulations 1, 3 and 4 at *t*=140, OE of STAT3 showed downregulation of migration in functional modules 2 and 4. In simulation 2 at *t*=140, OE of STAT3, showed regulation of migration in both functional modules 2 and 4 (8% in functional module 2; 15% in functional module 4)

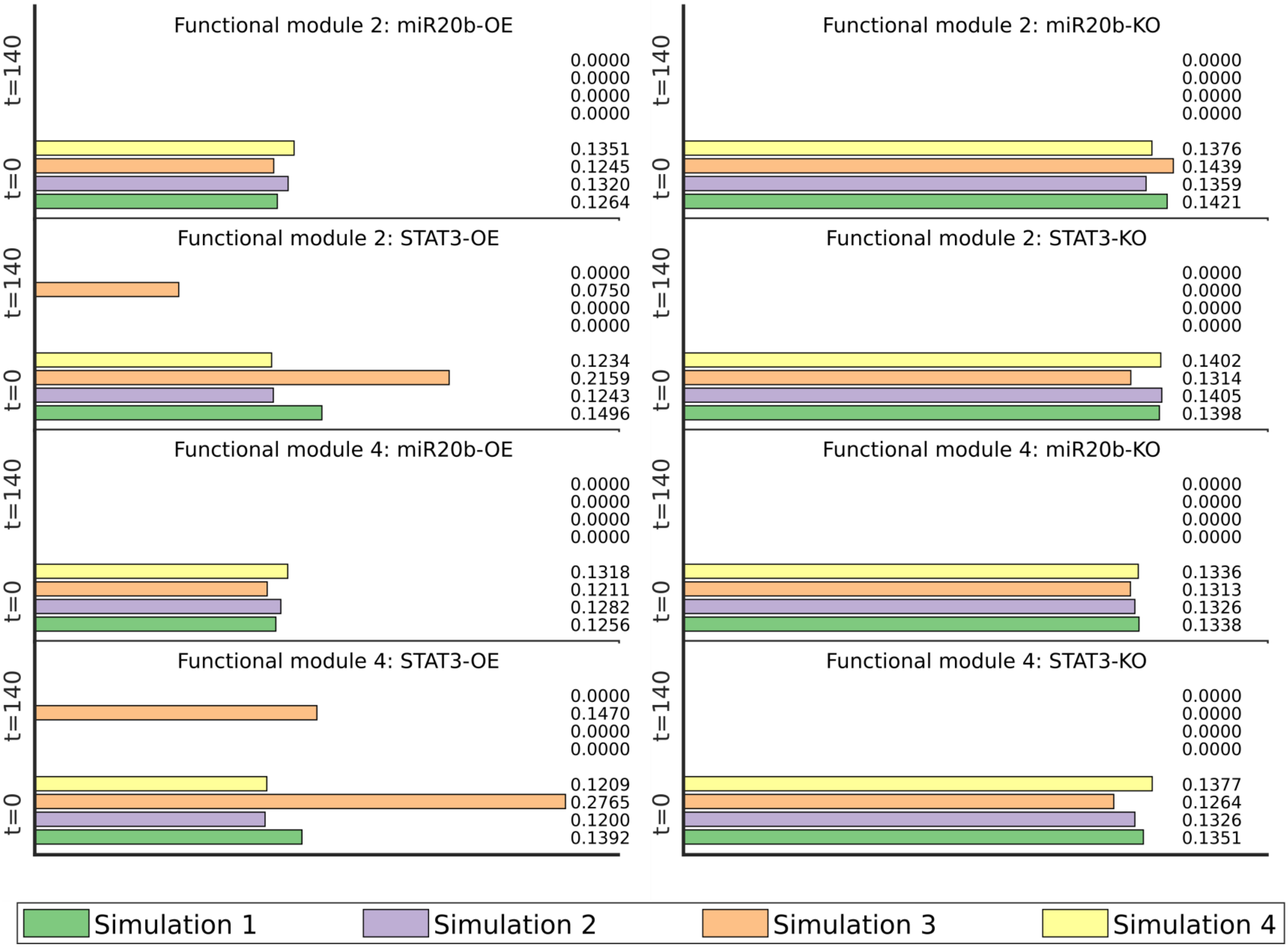

Supplementary Figure S39: Perturbation results of miR20b and STAT3 in feedback loop regulating functional modules 2 and 4

Perturbations were carried out for four different simulations i.e. when TF upregulates gene and miRNA expression (Simulation 1), when TF downregulates gene and miRNA expression (Simulation 2), when TF downregulates miRNA expression and upregulates gene expression (Simulation 3) and when TF upregulates miRNA and downregulates gene expression (Simulation 4). In all 4 simulations at *t*=140, overexpression (OE) /knock out (KO) of miR20b and KO of STAT3 in both functional module 2 and functional module 4, showed downregulation of migration (0%). In simulations 1, 3 and 4 at *t*=140, OE of STAT3 showed downregulation of migration in functional modules 2 and 4. In simulation 2 at *t*=140, OE of STAT3, showed regulation of migration in both functional modules 2 and 4 (7% in functional module 2; 14% in functional module 4)

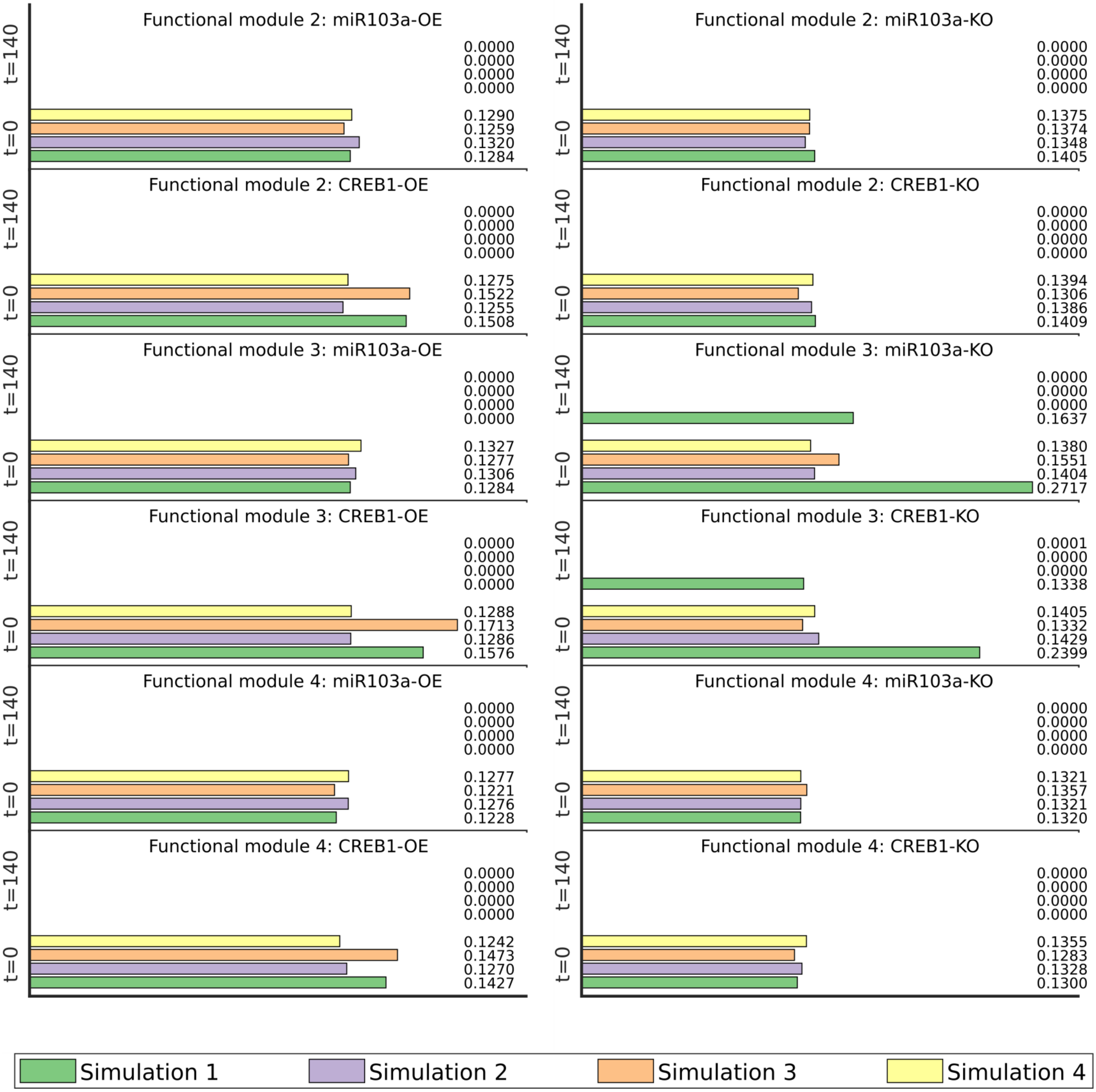

Supplementary Figure S40: Perturbation results of miR103a and CREB1 in feedback loop regulating functional modules 2, 3 and 4

Perturbations were carried out for four different simulations i.e. when TF upregulates gene and miRNA expression (Simulation 1), when TF downregulates gene and miRNA expression (Simulation 2), when TF downregulates miRNA expression and upregulates gene expression (Simulation 3) and when TF upregulates miRNA and downregulates gene expression (Simulation 4).

In all 4 simulations at *t*=140, overexpression (OE) of miR103a/CREB1 in functional modules 2, 3 and 4, showed downregulation of migration (0%). In simulations 2, 3 and 4 at *t*=140, knock out (KO) of CREB1/miR103a showed downregulation of migration in functional modules 2, 3 and 4. In simulation 1 at *t*=140, KO of CREB1/miR103a, showed downregulation of migration in functional modules 2 and 4. At simulation 1 at *t*=140, KO of CREB1, showed regulation of migration (13%) in functional module 3 at t=140; KO of miR103a showed regulation of migration (16%) in functional module 3

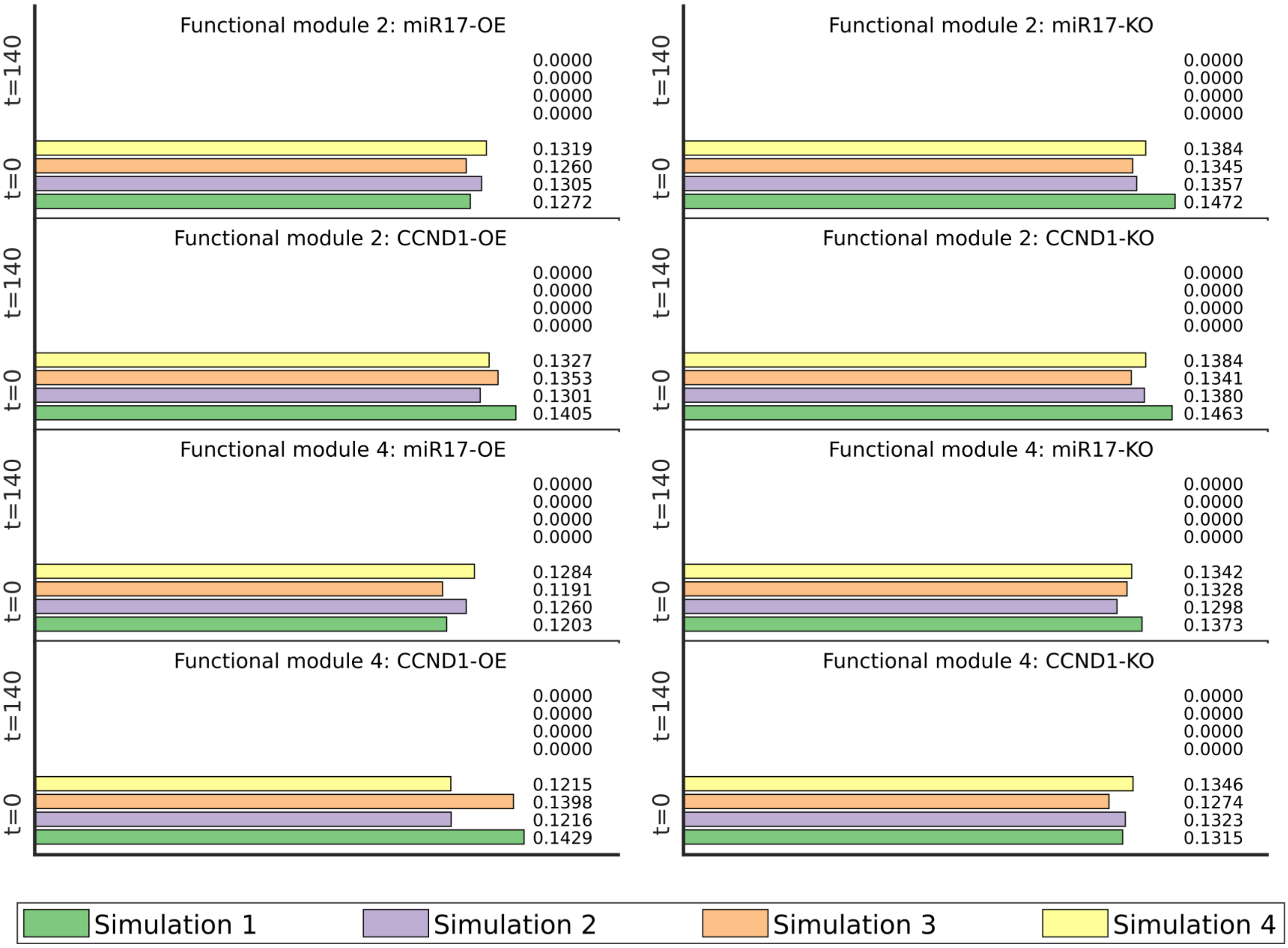

Supplementary Figure S41: Perturbation results of miR17 and CCND1 in feedback loop regulating functional modules 2 and 4

Perturbations were carried out for four different simulations i.e. when TF upregulates gene and miRNA expression (Simulation 1), when TF downregulates gene and miRNA expression (Simulation 2), when TF downregulates miRNA expression and upregulates gene expression (Simulation 3) and when TF upregulates miRNA and downregulates gene expression (Simulation 4). In all 4 simulations at *t*=140, overexpression (OE) /knock out (KO) of miR17 and CCND1 in both functional module 2 and functional module 4, showed downregulation of migration (0%).

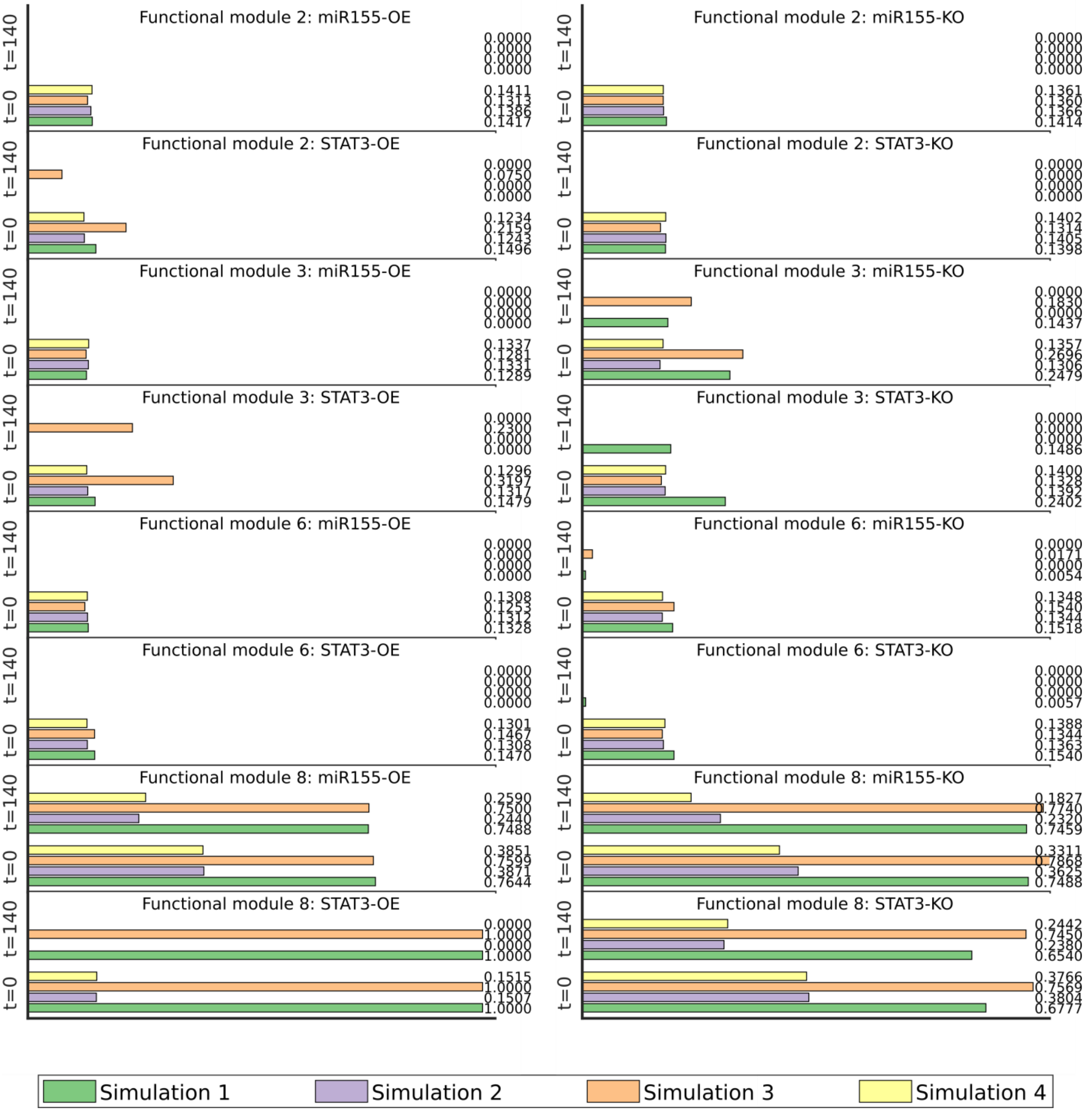

Supplementary Figure S42: Perturbation results of miR155 and STAT3 in feedback loop regulating functional modules 2, 3, 6 and 8.

Perturbations were carried out for four different simulations, when TF upregulates gene and miRNA expression (Simulation 1), when TF downregulates gene and miRNA expression (Simulation 2), when TF downregulates miRNA expression and upregulates gene expression (Simulation 3) and when TF upregulates miRNA and downregulates gene expression (Simulation 4). In all the 4 simulations at *t*=140, overexpression (OE) of miR155 showed downregulation of migration (0%) in functional modules 2, 3 and 6. In all 4 simulations at *t*=140, miR155/STAT3 knock out (KO) in functional module 2 downregulated migration (0%). In simulations 1 and 3 at *t*=140, miR155 KO showed regulation of migration in functional modules 3 and 6; at simulation 1, 14% in functional module 3 and 0.5% in functional module 6; at simulation 3, 18% in functional module 3 and 1% in functional module 6. At *t*=140, OE of miR155, regulated migration in functional module 8, 74% in simulation 1, 24% in simulation 2, 75% in simulation 3 and 25% in simulation 4. At *t*=140, miR155 KO in functional module 8, showed regulation of migration, 74% in simulation 1, 23% in simulation 2, 77% in simulation 3 and 18% in simulation 4. At t=140 in simulation 3, STAT3 OE regulated migration in functional module 2 (7%) and in functional module 3 (23%). At *t*=140, in simulation 1, STAT3 KO in functional module 3 showed regulation of migration, 14%. At *t*=140, in simulation 1, STAT3 KO in functional modules 3 and 6 showed regulation of migration, 0.5% in functional module 6. In all the four simulations at *t*=140, STAT3 OE showed downregulation of migration in functional module 6. At *t*=140, in simulations 1 and 3, STAT3 OE in functional module 8 showed upregulation of migration (100%). In simulation 4 at *t*=140, STAT3 KO in functional module 8 showed regulation of migration, 65% in simulation 1, 23% in simulation 2, 74% in simulation 3 and 24% in simulation 4.
