# Supplementary material for: An in-silico approach for discovery of microRNA-TF regulation of DISC1 interactome mediating neuronal migration
Source: NPJ Syst Biol Appl. 2019 May 7;5:17. doi: 10.1038/s41540-019-0094-3 (PMC6504871; doi:10.1038/s41540-019-0094-3)
Supplement: Supplementary file 2 — Supplementary Table S2 [file 41540_2019_94_MOESM2_ESM.pdf]

Genes involved in migration	EntrezID
ACTB	60
ACTB	60
ACTB	60
ACTB	60
ACTB	60
ACTB	60
ACTB	60
ACTB	60
ACTB	60
ACTB	60
ACTB	60
ACTB	60
ACTB	60
ACTB	60
ACTB	60
ACTB	60
ACTB	60
ACTB	60
ACTB	60
ACTB	60
ACTB	60
ACTB	60
ACTB	60
ACTB	60
ACTB	60
ACTB	60
ACTB	60
ACTB	60
ACTB	60
ACTB	60
ACTB	60
ACTB	60
ACTB	60
ACTB	60
ACTB	60
ACTB	60
ACTB	60
ACTB	60
ACTB	60
ACTB	60
ACTB	60
ACTB	60
ACTB	60
ACTB	60
ACTB	60
ACTB	60

ACTB	60
ACTB	60
ACTB	60
ACTB	60
ACTB	60
ACTB	60
ACTB	60
ACTB	60
ACTB	60
ACTB	60
ACTB	60
ACTB	60
ACTB	60
ACTB	60
ACTB	60
ACTB	60
ACTB	60
ACTB	60
ACTB	60
ACTB	60
ACTB	60
ACTB	60
ACTB	60
ACTB	60
ACTB	60
ACTB	60
ACTB	60
ACTB	60
ACTB	60
ACTB	60
ACTB	60
ACTB	60
ACTB	60
ACTB	60
ACTB	60
ACTB	60
ACTB	60
ACTB	60
ACTB	60
ACTB	60
ACTB	60
ACTB	60
ACTB	60
ACTB	60
ACTB	60
ACTB	60
ACTB	60

ACTB	60
ACTB	60
ACTB	60
ACTB	60
ACTB	60
ACTB	60
ACTB	60
ACTB	60
ACTB	60
ACTB	60
ACTB	60
ACTB	60
ACTB	60
ACTB	60
ACTB	60
ACTB	60
ACTB	60
ACTB	60
ACTB	60
ACTB	60
ACTB	60
ACTB	60
ACTB	60
ACTB	60
ACTB	60
ACTB	60
ACTB	60
ACTB	60
ACTB	60
ACTB	60
ACTB	60
ACTB	60
ACTB	60
ACTB	60
ACTB	60
ACTB	60
ACTB	60
ACTB	60
ACTB	60
ACTB	60
ACTB	60
ACTB	60
ACTB	60
ACTB	60
ACTB	60
ACTB	60
ACTB	60

ACTB	60
ACTB	60
ACTB	60
ACTB	60
ACTB	60
ACTB	60
ACTB	60
ACTB	60
ACTB	60
ACTB	60
ACTB	60
ACTB	60
ACTB	60
ACTB	60
ACTB	60
ACTB	60
AKT1	207
AKT1	207
AKT1	207
AKT1	207
AKT1	207
AKT1	207
AKT1	207
AKT1	207
AKT1	207
AKT1	207
AKT1	207
AKT1	207
AKT1	207
AKT1	207
AKT1	207
AKT1	207
AKT1	207
AKT1	207
AKT1	207
AKT1	207
AKT1	207
AKT1	207
AKT1	207
AKT1	207
AKT1	207
AKT1	207
AKT1	207
AKT1	207
AKT1	207
AKT1	207
AKT1	207

AKT1	207
AKT1	207
AKT1	207
AKT1	207
AKT1	207
AKT1	207
AKT1	207
AKT1	207
AKT1	207
AKT1	207
AKT1	207
AKT1	207
AKT1	207
AKT1	207
AKT1	207
AKT1	207
AKT1	207
AKT1	207
AKT1	207
APP	351
APP	351
APP	351
APP	351
APP	351
APP	351
APP	351
APP	351
APP	351
APP	351
APP	351
APP	351
APP	351
APP	351
APP	351
APP	351
APP	351
APP	351
APP	351
APP	351
APP	351
APP	351
APP	351
APP	351
APP	351
APP	351
APP	351
APP	351

APP	351
APP	351
APP	351
APP	351
APP	351
APP	351
APP	351
APP	351
APP	351
APP	351
APP	351
APP	351
APP	351
APP	351
APP	351
APP	351
APP	351
APP	351
APP	351
APP	351
APP	351
APP	351
APP	351
APP	351
APP	351
APP	351
APP	351
APP	351
APP	351
APP	351
APP	351
APP	351
APP	351
APP	351
APP	351
APP	351
APP	351
APP	351
APP	351
APP	351
APP	351
APP	351
APP	351
APP	351
APP	351
APP	351
APP	351

APP	351
APP	351
APP	351
APP	351
APP	351
APP	351
APP	351
APP	351
APP	351
APP	351
APP	351
APP	351
APP	351
BBS4	585
BBS4	585
BBS4	585
BBS4	585
BBS4	585
CCDC141	285025
CCDC141	285025
CCDC141	285025
CCDC141	285025
CCDC141	285025
CCDC141	285025
CCDC141	285025
CCDC141	285025
CCDC141	285025
CCDC88A	55704
CCDC88A	55704
CCDC88A	55704
CCDC88A	55704
CCDC88A	55704
CCDC88A	55704
CCDC88A	55704
CCDC88A	55704
CCDC88A	55704
CCDC88A	55704
CCDC88A	55704
CCDC88A	55704
CCDC88A	55704
CCDC88A	55704
CCDC88A	55704
CCDC88A	55704
CCDC88A	55704
CDK5	1020
DISC1	27185
DISC1	27185

DISC1	27185
DISC1	27185
DISC1	27185
DISC1	27185
DISC1	27185
DISC1	27185
DISC1	27185
DISC1	27185
DISC1	27185
DISC1	27185
DISC1	27185
DISC1	27185
DISC1	27185
DISC1	27185
DISC1	27185
DISC1	27185
DISC1	27185
DISC1	27185
DISC1	27185
DISC1	27185
DISC1	27185
DISC1	27185
DISC1	27185
DISC1	27185
DISC1	27185
DISC1	27185
DISC1	27185
DISC1	27185
DISC1	27185
DISC1	27185
DISC1	27185
DISC1	27185
DISC1	27185
DISC1	27185
DISC1	27185
DISC1	27185
DISC1	27185
DISC1	27185
DISC1	27185
DISC1	27185
DISC1	27185
DISC1	27185
DISC1	27185
DISC1	27185
DISC1	27185
DIXDC1	85458
DIXDC1	85458

DIXDC1	85458
DIXDC1	85458
FOXD3	27022
GSK3B	2932
GSK3B	2932
GSK3B	2932
GSK3B	2932
GSK3B	2932
GSK3B	2932
GSK3B	2932
GSK3B	2932
GSK3B	2932
GSK3B	2932
GSK3B	2932
GSK3B	2932
GSK3B	2932
GSK3B	2932
GSK3B	2932
GSK3B	2932
GSK3B	2932
GSK3B	2932
GSK3B	2932
GSK3B	2932
GSK3B	2932
GSK3B	2932
GSK3B	2932
GSK3B	2932
GSK3B	2932
GSK3B	2932
GSK3B	2932
GSK3B	2932
GSK3B	2932
GSK3B	2932
GSK3B	2932
GSK3B	2932
GSK3B	2932
GSK3B	2932
GSK3B	2932
GSK3B	2932
GSK3B	2932
GSK3B	2932
GSK3B	2932
GSK3B	2932
GSK3B	2932
GSK3B	2932
GSK3B	2932
GSK3B	2932

GSK3B	2932
GSK3B	2932
GSK3B	2932
GSK3B	2932
GSK3B	2932
GSK3B	2932
GSK3B	2932
GSK3B	2932
GSK3B	2932
GSK3B	2932
GSK3B	2932
GSK3B	2932
GSK3B	2932
GSK3B	2932
GSK3B	2932
GSK3B	2932
GSK3B	2932
GSK3B	2932
GSK3B	2932
GSK3B	2932
GSK3B	2932
GSK3B	2932
GSK3B	2932
GSK3B	2932
GSK3B	2932
GSK3B	2932
GSK3B	2932
GSK3B	2932
GSK3B	2932
GSK3B	2932
GSK3B	2932
GSK3B	2932
GSK3B	2932
GSK3B	2932
GSK3B	2932
GSK3B	2932
GSK3B	2932
GSK3B	2932
GSK3B	2932
GSK3B	2932
GSK3B	2932
GSK3B	2932
GSK3B	2932
GSK3B	2932
GSK3B	2932
GSK3B	2932
GSK3B	2932

GSK3B	2932
GSK3B	2932
GSK3B	2932
GSK3B	2932
GSK3B	2932
GSK3B	2932
GSK3B	2932
GSK3B	2932
GSK3B	2932
GSK3B	2932
GSK3B	2932
GSK3B	2932
GSK3B	2932
GSK3B	2932
MYH2	4620
MYH2	4620
MYH2	4620
MYH2	4620
MYH2	4620
MYH2	4620
MYH2	4620
MYH2	4620
MYH2	4620
MYH2	4620
MYH2	4620
MYH2	4620
MYH2	4620
MYH2	4620
MYH2	4620
MYH2	4620
MYH2	4620
MYH2	4620
MYH2	4620
MYH2	4620
MYH2	4620
MYH2	4620
MYH2	4620
MYH2	4620
MYH2	4620
MYH2	4620
MYH2	4620
MYH2	4620
MYH2	4620
MYH2	4620
MYH2	4620
MYH2	4620
MYH2	4620

MYH2	4620
MYH2	4620
MYH2	4620
MYH2	4620
MYH2	4620
NDEL1	81565
NDEL1	81565
NDEL1	81565
NDEL1	81565
NDEL1	81565
NDEL1	81565
NDEL1	81565
NDEL1	81565
NDEL1	81565
NDEL1	81565
NDEL1	81565
NDEL1	81565
NDEL1	81565
NDEL1	81565
NDEL1	81565
NDEL1	81565
NDEL1	81565
NDEL1	81565
NDEL1	81565
NDEL1	81565
NDEL1	81565
NDEL1	81565
NDEL1	81565
NDEL1	81565
NDEL1	81565
NDEL1	81565
NDEL1	81565
PAFAH1B1	5048
PAFAH1B1	5048
PAFAH1B1	5048
PAFAH1B1	5048
PAFAH1B1	5048
PAFAH1B1	5048
PAFAH1B1	5048
PAFAH1B1	5048
PAFAH1B1	5048
PAFAH1B1	5048
PAFAH1B1	5048
PAFAH1B1	5048
PAFAH1B1	5048
PAFAH1B1	5048
PAFAH1B1	5048

PAFAH1B1	5048
PAFAH1B1	5048
PAFAH1B1	5048
PAFAH1B1	5048
PAFAH1B1	5048
PAFAH1B1	5048
PAFAH1B1	5048
PAFAH1B1	5048
PAFAH1B1	5048
PAFAH1B1	5048
PAFAH1B1	5048
PAFAH1B1	5048
PAFAH1B1	5048
PAFAH1B1	5048
PAFAH1B1	5048
PAFAH1B1	5048
PAFAH1B1	5048
PAFAH1B1	5048
PAFAH1B1	5048
PAFAH1B1	5048
PAFAH1B1	5048
PAFAH1B1	5048
PAFAH1B1	5048
PAFAH1B1	5048
PAFAH1B1	5048
PAFAH1B1	5048
PAFAH1B1	5048
PAFAH1B1	5048
PAFAH1B1	5048
PAFAH1B1	5048
PAFAH1B1	5048
PAFAH1B1	5048
PCM1	5108
ZNF365	22891
ZNF365	22891
ZNF365	22891
RHEB	6009
RHEB	6009
RHEB	6009
RHEB	6009
RHEB	6009
RHEB	6009

miRNAs (from miRWALK database) targeting gene involved in migration	MIMATid	PMID
hsa-miR-557	MIMAT0003221	20371350
hsa-miR-19b-3p	MIMAT0000074	23592263
hsa-miR-1295a	MIMAT0005885	23622248
hsa-miR-937-5p	MIMAT0022938	23592263
hsa-let-7b-5p	MIMAT0000063	23622248
hsa-miR-191-3p	MIMAT0001618	24398324
hsa-miR-548l	MIMAT0005889	23446348
hsa-miR-6507-3p	MIMAT0025471	21572407
hsa-miR-5681a	MIMAT0022469	20371350
hsa-miR-7-2-3p	MIMAT0004554	21572407
hsa-miR-507	MIMAT0002879	20371350
hsa-miR-19a-3p	MIMAT0000073	23592263
hsa-miR-744-5p	MIMAT0004945	23622248
hsa-miR-1200	MIMAT0005863	23592263
hsa-let-7a-5p	MIMAT0000062	23622248
hsa-miR-329-5p	MIMAT0026555	24398324
hsa-miR-548n	MIMAT0005916	23446348
hsa-miR-937-5p	MIMAT0022938	21572407
hsa-miR-191-3p	MIMAT0001618	20371350
hsa-miR-7-1-3p	MIMAT0004553	21572407
hsa-miR-450b-5p	MIMAT0004909	20371350
hsa-miR-5010-5p	MIMAT0021043	23592263
hsa-miR-455-3p	MIMAT0004784	23622248
hsa-miR-5702	MIMAT0022495	23592263
hsa-miR-3176	MIMAT0015053	23622248
hsa-miR-4464	MIMAT0018988	24398324
hsa-miR-506-3p	MIMAT0002878	23446348
hsa-miR-1200	MIMAT0005863	21572407
hsa-miR-329-5p	MIMAT0026555	20371350
hsa-miR-145-5p	MIMAT0000437	23592263
hsa-miR-548t-3p	MIMAT0022730	23592263
hsa-miR-33b-5p	MIMAT0003301	20371350
hsa-miR-4525	MIMAT0019064	23592263
hsa-miR-93-3p	MIMAT0004509	23622248
hsa-miR-544b	MIMAT0015004	23592263
hsa-miR-544a	MIMAT0003164	23446348
hsa-miR-4748	MIMAT0019884	24398324
hsa-miR-124-3p	MIMAT0000422	23446348
hsa-miR-5702	MIMAT0022495	21572407
hsa-miR-4464	MIMAT0018988	20371350
hsa-miR-548aa	MIMAT0018447	23592263
hsa-miR-33a-5p	MIMAT0000091	20371350
hsa-miR-7111-5p	MIMAT0028119	23592263
hsa-miR-484	MIMAT0002174	23622248
hsa-miR-4324	MIMAT0016876	23592263
hsa-miR-6772-5p	MIMAT0027444	23446348

hsa-miR-3912-3p	MIMAT0018186	24398324
hsa-miR-6738-3p	MIMAT0027378	23446348
hsa-miR-544b	MIMAT0015004	21572407
hsa-miR-4748	MIMAT0019884	20371350
hsa-miR-548ap-3p	MIMAT0021038	23592263
hsa-miR-382-3p	MIMAT0022697	20371350
hsa-miR-6870-5p	MIMAT0027640	23592263
hsa-miR-324-5p	MIMAT0000761	23622248
hsa-miR-4328	MIMAT0016926	23592263
hsa-let-7i-3p	MIMAT0004585	23446348
hsa-miR-412-3p	MIMAT0002170	24374217
hsa-miR-548t-3p	MIMAT0022730	21572407
hsa-miR-4324	MIMAT0016876	21572407
hsa-miR-3912-3p	MIMAT0018186	20371350
hsa-miR-4289	MIMAT0016920	23592263
hsa-miR-5698	MIMAT0022491	23592263
hsa-miR-326	MIMAT0000756	23622248
hsa-miR-3613-3p	MIMAT0017991	23592263
hsa-miR-5681a	MIMAT0022469	23446348
hsa-miR-544a	MIMAT0003164	24374217
hsa-miR-548aa	MIMAT0018447	21572407
hsa-miR-3613-3p	MIMAT0017991	21572407
hsa-miR-4477a	MIMAT0019004	20371350
hsa-miR-5572	MIMAT0022260	23592263
hsa-miR-4723-5p	MIMAT0019838	23592263
hsa-miR-378a-3p	MIMAT0000732	23622248
hsa-miR-605-5p	MIMAT0003273	23592263
hsa-miR-191-3p	MIMAT0001618	23446348
hsa-miR-432-3p	MIMAT0002815	24374217
hsa-miR-548ap-3p	MIMAT0021038	21572407
hsa-miR-605-5p	MIMAT0003273	21572407
hsa-miR-4699-5p	MIMAT0019794	20371350
hsa-miR-4260	MIMAT0016881	23592263
hsa-miR-644a	MIMAT0003314	23091630
hsa-miR-6876-3p	MIMAT0027653	23592263
hsa-miR-320a	MIMAT0000510	23622248
hsa-miR-548z	MIMAT0018446	23592263
hsa-miR-329-5p	MIMAT0026555	23446348
hsa-miR-4289	MIMAT0016920	21572407
hsa-miR-145-5p	MIMAT0000437	19439999
hsa-miR-548z	MIMAT0018446	21572407
hsa-miR-525-3p	MIMAT0002839	20371350
hsa-miR-450a-2-3p	MIMAT0031074	23592263
hsa-miR-626	MIMAT0003295	23592263
hsa-miR-222-3p	MIMAT0000279	23622248
hsa-miR-548h-3p	MIMAT0022723	23592263
hsa-miR-4464	MIMAT0018988	23446348

hsa-miR-493-5p	MIMAT0002813	24374217
hsa-miR-3611	MIMAT0017988	21572407
hsa-miR-548h-3p	MIMAT0022723	21572407
hsa-miR-524-3p	MIMAT0002850	20371350
hsa-miR-3611	MIMAT0017988	23592263
hsa-miR-520h	MIMAT0002867	23592263
hsa-miR-221-3p	MIMAT0000278	23622248
hsa-miR-548d-3p	MIMAT0003323	23592263
hsa-miR-4748	MIMAT0019884	23446348
hsa-miR-127-5p	MIMAT0004604	24374217
hsa-miR-19b-3p	MIMAT0000074	21572407
hsa-miR-4282	MIMAT0016912	20371350
hsa-miR-548d-3p	MIMAT0003323	21572407
hsa-miR-548l	MIMAT0005889	20371350
hsa-miR-6803-5p	MIMAT0027506	23592263
hsa-miR-520g-3p	MIMAT0002858	23592263
hsa-miR-196a-5p	MIMAT0000226	23622248
hsa-miR-548ac	MIMAT0018938	23592263
hsa-miR-3912-3p	MIMAT0018186	23446348
hsa-miR-493-3p	MIMAT0003161	24374217
hsa-miR-19a-3p	MIMAT0000073	21572407
hsa-miR-4698	MIMAT0019793	20371350
hsa-miR-548ac	MIMAT0018938	21572407
hsa-miR-548n	MIMAT0005916	20371350
hsa-miR-6751-5p	MIMAT0027402	23592263
hsa-miR-3973	MIMAT0019358	23592263
hsa-miR-100-5p	MIMAT0000098	23622248
hsa-miR-5195-3p	MIMAT0021127	23592263
hsa-miR-4477a	MIMAT0019004	23446348
hsa-miR-520h	MIMAT0002867	21572407
hsa-miR-8063	MIMAT0030990	20371350
hsa-miR-192-3p	MIMAT0004543	21572407
hsa-miR-506-3p	MIMAT0002878	20371350
hsa-miR-7109-5p	MIMAT0028115	23592263
hsa-miR-218-5p	MIMAT0000275	23212916
hsa-miR-6510-3p	MIMAT0025477	23592263
hsa-miR-92a-3p	MIMAT0000092	23622248
hsa-miR-6772-5p	MIMAT0027444	24398324
hsa-miR-4699-5p	MIMAT0019794	23446348
hsa-miR-520g-3p	MIMAT0002858	21572407
hsa-miR-544a	MIMAT0003164	20371350
hsa-miR-6870-3p	MIMAT0027641	21572407
hsa-miR-124-3p	MIMAT0000422	20371350
hsa-miR-4665-5p	MIMAT0019739	23592263
hsa-miR-1307-3p	MIMAT0005951	23622248
hsa-miR-3529-3p	MIMAT0022741	23592263
hsa-miR-16-5p	MIMAT0000069	23622248

hsa-let-7i-3p	MIMAT0004585	24398324
hsa-miR-525-3p	MIMAT0002839	23446348
hsa-miR-3973	MIMAT0019358	21572407
hsa-miR-6772-5p	MIMAT0027444	20371350
hsa-miR-6872-3p	MIMAT0027645	21572407
hsa-miR-6738-3p	MIMAT0027378	20371350
hsa-miR-3680-3p	MIMAT0018107	20371350
hsa-miR-1275	MIMAT0005929	23592263
hsa-miR-1303	MIMAT0005891	23622248
hsa-miR-6507-3p	MIMAT0025471	23592263
hsa-let-7c-5p	MIMAT0000064	23622248
hsa-miR-5681a	MIMAT0022469	24398324
hsa-miR-524-3p	MIMAT0002850	23446348
hsa-miR-3529-3p	MIMAT0022741	21572407
hsa-let-7i-3p	MIMAT0004585	20371350
hsa-miR-216a-5p	MIMAT0000273	21572407
hsa-miR-708-5p	MIMAT0004926	23754151
hsa-miR-378c	MIMAT0016847	21572407
hsa-miR-26b-5p	MIMAT0000083	19088304
hsa-miR-27a-5p	MIMAT0004501	23963114
hsa-miR-378b	MIMAT0014999	21572407
hsa-miR-133b	MIMAT0000770	25414595
hsa-miR-378a-3p	MIMAT0000732	21572407
hsa-miR-4638-3p	MIMAT0019696	21572407
hsa-miR-105-5p	MIMAT0000102	25280563
hsa-miR-6824-3p	MIMAT0027549	21572407
hsa-miR-143-3p	MIMAT0000435	23104321
hsa-miR-192-3p	MIMAT0004543	22100165
hsa-miR-6764-3p	MIMAT0027429	21572407
hsa-miR-149-3p	MIMAT0004609	20623644
hsa-miR-193b-3p	MIMAT0002819	23622248
hsa-miR-155-5p	MIMAT0000646	22100165
hsa-miR-199a-3p	MIMAT0000232	18456660
hsa-miR-330-5p	MIMAT0004693	21572407
hsa-miR-451a	MIMAT0001631	20816946
hsa-miR-496	MIMAT0002818	22100165
hsa-miR-3191-5p	MIMAT0022732	21572407
hsa-miR-125b-5p	MIMAT0000423	18649363
hsa-miR-326	MIMAT0000756	21572407
hsa-miR-4742-3p	MIMAT0019873	22100165
hsa-miR-6873-3p	MIMAT0027647	21572407
hsa-miR-6817-3p	MIMAT0027535	21572407
hsa-miR-302a-3p	MIMAT0000684	23185040
hsa-miR-654-3p	MIMAT0004814	22100165
hsa-miR-100-5p	MIMAT0000098	23724047
hsa-miR-6503-3p	MIMAT0025463	21572407
hsa-miR-185-5p	MIMAT0000455	19688090

hsa-miR-518c-5p	MIMAT0002847	21572407
hsa-miR-302b-3p	MIMAT0000715	23185040
hsa-miR-4757-3p	MIMAT0019902	22100165
hsa-miR-422a	MIMAT0001339	21572407
hsa-miR-7110-3p	MIMAT0028118	21572407
hsa-miR-302c-3p	MIMAT0000717	23185040
hsa-miR-365b-3p	MIMAT0022834	22100165
hsa-miR-378i	MIMAT0019074	21572407
hsa-miR-6866-3p	MIMAT0027633	21572407
hsa-miR-302d-3p	MIMAT0000718	23185040
hsa-miR-365a-3p	MIMAT0000710	22100165
hsa-miR-378h	MIMAT0018984	21572407
hsa-miR-188-5p	MIMAT0000457	21572407
hsa-miR-378f	MIMAT0018932	21572407
hsa-miR-625-3p	MIMAT0004808	21572407
hsa-miR-19a-3p	MIMAT0000073	25400827
hsa-miR-378e	MIMAT0018927	21572407
hsa-miR-19a-3p	MIMAT0000073	25914465
hsa-miR-378d	MIMAT0018926	21572407
hsa-miR-4698	MIMAT0019793	21572407
hsa-miR-3620-3p	MIMAT0018001	23622248
hsa-miR-548ar-3p	MIMAT0022266	23824327
hsa-miR-6073	MIMAT0023698	23313552
hsa-miR-16-5p	MIMAT0000069	21572407
hsa-miR-3646	MIMAT0018065	19536157
hsa-miR-4495	MIMAT0019030	21572407
hsa-miR-101-3p	MIMAT0000099	21172309
hsa-miR-548a-3p	MIMAT0003251	23824327
hsa-miR-3132	MIMAT0014997	23313552
hsa-miR-106b-5p	MIMAT0000680	19110058
hsa-let-7a-2-3p	MIMAT0010195	21572407
hsa-miR-3120-3p	MIMAT0014982	19536157
hsa-miR-583	MIMAT0003248	21572407
hsa-miR-1238-3p	MIMAT0005593	23824327
hsa-miR-197-5p	MIMAT0022691	23313552
hsa-miR-106a-5p	MIMAT0000103	19110058
hsa-let-7g-3p	MIMAT0004584	21572407
hsa-miR-551b-5p	MIMAT0004794	19536157
hsa-miR-1276	MIMAT0005930	21572407
hsa-miR-670-3p	MIMAT0026640	23824327
hsa-miR-3908	MIMAT0018182	23313552
hsa-miR-20a-5p	MIMAT0000075	19110058
hsa-miR-455-3p	MIMAT0004784	21572407
hsa-miR-4484	MIMAT0019018	19536157
hsa-miR-4311	MIMAT0016863	21572407
hsa-miR-6881-3p	MIMAT0027663	23824327
hsa-miR-522-3p	MIMAT0002868	23313552

hsa-miR-17-5p	MIMAT0000070	19110058
hsa-miR-298	MIMAT0004901	21572407
hsa-miR-424-3p	MIMAT0004749	19536157
hsa-miR-297	MIMAT0004450	21572407
hsa-miR-7111-3p	MIMAT0028120	23824327
hsa-miR-224-3p	MIMAT0009198	23313552
hsa-miR-15a-5p	MIMAT0000068	19110058
hsa-miR-490-5p	MIMAT0004764	21572407
hsa-miR-3924	MIMAT0018199	21572407
hsa-miR-6780a-3p	MIMAT0027461	23824327
hsa-miR-130a-3p	MIMAT0000425	19110058
hsa-miR-545-5p	MIMAT0004785	21572407
hsa-miR-190a-3p	MIMAT0026482	21572407
hsa-miR-17-5p	MIMAT0000070	23622248
hsa-miR-664b-3p	MIMAT0022272	23824327
hsa-let-7d-5p	MIMAT0000065	19110058
hsa-miR-3688-3p	MIMAT0018116	21572407
hsa-miR-5011-5p	MIMAT0021045	21572407
hsa-miR-532-3p	MIMAT0004780	23622248
hsa-miR-579-3p	MIMAT0003244	23824327
hsa-let-7a-5p	MIMAT0000062	19110058
hsa-miR-3662	MIMAT0018083	21572407
hsa-miR-567	MIMAT0003231	21572407
hsa-miR-500a-5p	MIMAT0004773	23622248
hsa-miR-5696	MIMAT0022489	23824327
hsa-miR-15b-5p	MIMAT0000417	21572407
hsa-miR-6838-5p	MIMAT0027578	21572407
hsa-miR-944	MIMAT0004987	21572407
hsa-miR-484	MIMAT0002174	23622248
hsa-miR-302d-5p	MIMAT0004685	23824327
hsa-miR-6504-3p	MIMAT0025465	19536157
hsa-miR-497-5p	MIMAT0002820	21572407
hsa-miR-423-3p	MIMAT0001340	23622248
hsa-miR-302b-5p	MIMAT0000714	23824327
hsa-miR-4786-5p	MIMAT0019954	19536157
hsa-miR-424-5p	MIMAT0001341	21572407
hsa-miR-328-3p	MIMAT0000752	23622248
hsa-miR-5582-3p	MIMAT0022280	23824327
hsa-miR-16-5p	MIMAT0000069	18668040
hsa-miR-4709-3p	MIMAT0019812	19536157
hsa-miR-195-5p	MIMAT0000461	21572407
hsa-miR-222-3p	MIMAT0000279	23622248
hsa-miR-548f-3p	MIMAT0005895	23824327
hsa-miR-5093	MIMAT0021085	19536157
hsa-miR-144-3p	MIMAT0000436	21572407
hsa-miR-101-3p	MIMAT0000099	20395292
hsa-miR-196a-5p	MIMAT0000226	23622248

hsa-miR-548e-3p	MIMAT0005874	23824327
hsa-miR-1229-5p	MIMAT0022942	23313552
hsa-miR-520c-3p	MIMAT0002846	18684319
hsa-miR-101-3p	MIMAT0000099	21572407
hsa-miR-5584-5p	MIMAT0022283	19536157
hsa-miR-4786-5p	MIMAT0019954	21572407
hsa-miR-20a-5p	MIMAT0000075	20458444
hsa-miR-1260b	MIMAT0015041	23622248
hsa-miR-548az-3p	MIMAT0025457	23824327
hsa-miR-3606-5p	MIMAT0017983	23313552
hsa-miR-106a-5p	MIMAT0000103	18684319
hsa-miR-15a-5p	MIMAT0000068	21572407
hsa-miR-6502-3p	MIMAT0025461	19536157
hsa-miR-376c-3p	MIMAT0000720	24374217
hsa-miR-377-3p	MIMAT0000730	24374217
hsa-miR-127-5p	MIMAT0004604	24374217
hsa-miR-432-5p	MIMAT0002814	24374217
hsa-miR-382-5p	MIMAT0000737	24374217
hsa-miR-7703	MIMAT0030018	23824327
hsa-miR-6776-5p	MIMAT0027452	23824327
hsa-miR-6861-5p	MIMAT0027623	23824327
hsa-miR-335-5p	MIMAT0000765	18185580
hsa-miR-4793-3p	MIMAT0019966	23824327
hsa-miR-3925-3p	MIMAT0019228	23824327
hsa-miR-1273g-3p	MIMAT0022742	23824327
hsa-miR-622	MIMAT0003291	23824327
hsa-miR-508-5p	MIMAT0004778	23824327
hsa-miR-8085	MIMAT0031012	24398324
hsa-miR-4644	MIMAT0019704	24398324
hsa-miR-6731-5p	MIMAT0027363	24398324
hsa-miR-4306	MIMAT0016858	24398324
hsa-miR-185-5p	MIMAT0000455	24398324
hsa-miR-6133	MIMAT0024617	24398324
hsa-miR-6130	MIMAT0024614	24398324
hsa-miR-6129	MIMAT0024613	24398324
hsa-miR-6127	MIMAT0024610	24398324
hsa-miR-4510	MIMAT0019047	24398324
hsa-miR-4419a	MIMAT0018931	24398324
hsa-miR-6760-5p	MIMAT0027420	24398324
hsa-miR-375	MIMAT0000728	20215506
hsa-miR-6873-5p	MIMAT0027646	24398324
hsa-miR-583	MIMAT0003248	24398324
hsa-miR-4448	MIMAT0018967	24861464
hsa-miR-4311	MIMAT0016863	24398324
hsa-miR-155-5p	MIMAT0000646	18668040
hsa-miR-455-3p	MIMAT0004784	23592263
hsa-miR-4458	MIMAT0018980	23592263

hsa-miR-6895-3p	MIMAT0027691	23824327
hsa-miR-4451	MIMAT0018973	23592263
hsa-let-7i-5p	MIMAT0000415	23592263
hsa-miR-6845-3p	MIMAT0027591	23824327
hsa-miR-6799-5p	MIMAT0027498	23592263
hsa-let-7g-5p	MIMAT0000414	23592263
hsa-miR-136-5p	MIMAT0000448	23824327
hsa-miR-6883-5p	MIMAT0027666	23592263
hsa-let-7f-5p	MIMAT0000067	23592263
hsa-miR-622	MIMAT0003291	23824327
hsa-miR-6785-5p	MIMAT0027470	23592263
hsa-let-7e-5p	MIMAT0000066	23592263
hsa-miR-545-3p	MIMAT0003165	23824327
hsa-miR-4728-5p	MIMAT0019849	23592263
hsa-let-7d-5p	MIMAT0000065	23592263
hsa-miR-149-3p	MIMAT0004609	23592263
hsa-let-7c-5p	MIMAT0000064	23592263
hsa-miR-7106-5p	MIMAT0028109	23592263
hsa-let-7b-5p	MIMAT0000063	23592263
hsa-miR-6516-5p	MIMAT0030417	23592263
hsa-miR-744-5p	MIMAT0004945	23592263
hsa-let-7a-5p	MIMAT0000062	23592263
hsa-miR-6780a-5p	MIMAT0027460	23592263
hsa-miR-4749-5p	MIMAT0019885	23592263
hsa-miR-4537	MIMAT0019080	23592263
hsa-miR-124-3p	MIMAT0000422	18668037
hsa-miR-6779-5p	MIMAT0027458	23592263
hsa-miR-4706	MIMAT0019806	23592263
hsa-miR-5186	MIMAT0021116	23592263
hsa-miR-3689c	MIMAT0019007	23592263
hsa-miR-6499-3p	MIMAT0025451	23592263
hsa-miR-3192-5p	MIMAT0015076	23592263
hsa-miR-3689b-3p	MIMAT0018181	23592263
hsa-miR-6086	MIMAT0023711	23592263
hsa-miR-6831-5p	MIMAT0027562	23592263
hsa-miR-3689a-3p	MIMAT0018118	23592263
hsa-miR-377-5p	MIMAT0004689	23592263
hsa-miR-3927-3p	MIMAT0018202	23592263
hsa-miR-30b-3p	MIMAT0004589	23592263
hsa-miR-98-5p	MIMAT0000096	23592263
hsa-miR-7851-3p	MIMAT0030426	23592263
hsa-miR-1273h-5p	MIMAT0030415	23592263
hsa-miR-4500	MIMAT0019036	23592263
hsa-miR-490-3p	MIMAT0002806	23592263
hsa-miR-4291	MIMAT0016922	23824327
hsa-miR-615-3p	MIMAT0003283	20371350
hsa-miR-16-5p	MIMAT0000069	23622248

hsa-miR-582-3p	MIMAT0004797	23295946
hsa-miR-582-5p	MIMAT0003247	23295946
hsa-miR-335-5p	MIMAT0000765	18185580
hsa-miR-26a-5p	MIMAT0000082	20525681
hsa-miR-4789-5p	MIMAT0019959	21572407
hsa-miR-374b-5p	MIMAT0004955	23313552
hsa-miR-5692b	MIMAT0022497	21572407
hsa-miR-199a-5p	MIMAT0000231	23764775
hsa-miR-5011-5p	MIMAT0021045	21572407
hsa-miR-877-3p	MIMAT0004950	23622248
hsa-miR-8064	MIMAT0030991	23824327
hsa-miR-374a-5p	MIMAT0000727	23313552
hsa-miR-513c-3p	MIMAT0022728	21572407
hsa-miR-369-3p	MIMAT0000721	21572407
hsa-miR-190a-3p	MIMAT0026482	22012620
hsa-miR-16-5p	MIMAT0000069	22473208
hsa-miR-425-5p	MIMAT0003393	23622248
hsa-miR-4643	MIMAT0019703	23824327
hsa-miR-410-3p	MIMAT0002171	23313552
hsa-miR-513a-3p	MIMAT0004777	21572407
hsa-miR-1277-5p	MIMAT0022724	22012620
hsa-miR-5011-5p	MIMAT0021045	22012620
hsa-miR-195-5p	MIMAT0000461	22473208
hsa-miR-92a-3p	MIMAT0000092	23622248
hsa-miR-383-3p	MIMAT0026485	23824327
hsa-miR-5011-5p	MIMAT0021045	23446348
hsa-miR-29b-3p	MIMAT0000100	21501592
hsa-miR-5692c	MIMAT0022476	23313552
hsa-miR-3606-3p	MIMAT0022965	21572407
hsa-miR-374b-5p	MIMAT0004955	22012620
hsa-miR-26a-5p	MIMAT0000082	22484120
hsa-miR-15b-5p	MIMAT0000417	22473208
hsa-miR-3065-3p	MIMAT0015378	23824327
hsa-miR-1277-5p	MIMAT0022724	23446348
hsa-miR-5692b	MIMAT0022497	23313552
hsa-miR-6083	MIMAT0023708	21572407
hsa-miR-374a-5p	MIMAT0000727	22012620
hsa-miR-15a-5p	MIMAT0000068	22473208
hsa-miR-377-3p	MIMAT0000730	23824327
hsa-miR-374b-5p	MIMAT0004955	23446348
hsa-miR-513c-3p	MIMAT0022728	23313552
hsa-miR-26a-5p	MIMAT0000082	20371350
hsa-let-7a-3p	MIMAT0004481	20371350
hsa-miR-4465	MIMAT0018992	21572407
hsa-miR-410-3p	MIMAT0002171	22012620
hsa-miR-6787-3p	MIMAT0027475	23824327
hsa-miR-374a-5p	MIMAT0000727	23446348

hsa-miR-190a-3p	MIMAT0026482	23313552
hsa-miR-409-5p	MIMAT0001638	24374217
hsa-miR-369-3p	MIMAT0000721	23313552
hsa-let-7b-3p	MIMAT0004482	20371350
hsa-miR-26b-5p	MIMAT0000083	21572407
hsa-miR-5692c	MIMAT0022476	22012620
hsa-miR-548c-3p	MIMAT0003285	23824327
hsa-miR-410-3p	MIMAT0002171	23446348
hsa-miR-346	MIMAT0000773	24023731
hsa-miR-513a-3p	MIMAT0004777	23313552
hsa-let-7f-1-3p	MIMAT0004486	20371350
hsa-miR-1297	MIMAT0005886	21572407
hsa-miR-5692b	MIMAT0022497	22012620
hsa-miR-5692c	MIMAT0022476	23446348
hsa-miR-4465	MIMAT0018992	20371350
hsa-miR-3606-3p	MIMAT0022965	23313552
hsa-let-7f-2-3p	MIMAT0004487	20371350
hsa-miR-5697	MIMAT0022490	21572407
hsa-miR-513c-3p	MIMAT0022728	22012620
hsa-miR-5692b	MIMAT0022497	23446348
hsa-miR-26b-5p	MIMAT0000083	20371350
hsa-miR-155-5p	MIMAT0000646	19289835
hsa-miR-6083	MIMAT0023708	23313552
hsa-miR-98-3p	MIMAT0022842	20371350
hsa-miR-5683	MIMAT0022472	21572407
hsa-miR-369-3p	MIMAT0000721	22012620
hsa-miR-513c-3p	MIMAT0022728	23446348
hsa-miR-183-5p	MIMAT0000261	24335145
hsa-miR-1297	MIMAT0005886	20371350
hsa-miR-1910-5p	MIMAT0007884	23313552
hsa-miR-1185-2-3p	MIMAT0022713	20371350
hsa-miR-33a-3p	MIMAT0004506	21572407
hsa-miR-513a-3p	MIMAT0004777	22012620
hsa-miR-26a-5p	MIMAT0000082	21572407
hsa-miR-369-3p	MIMAT0000721	23446348
hsa-miR-5697	MIMAT0022490	20371350
hsa-miR-1277-5p	MIMAT0022724	21572407
hsa-miR-1185-1-3p	MIMAT0022838	20371350
hsa-miR-409-3p	MIMAT0001639	21572407
hsa-miR-3606-3p	MIMAT0022965	22012620
hsa-let-7f-2-3p	MIMAT0004487	21572407
hsa-miR-513a-3p	MIMAT0004777	23446348
hsa-miR-96-5p	MIMAT0000095	24335145
hsa-miR-5683	MIMAT0022472	20371350
hsa-miR-374b-5p	MIMAT0004955	21572407
hsa-miR-4789-5p	MIMAT0019959	20371350
hsa-miR-1910-5p	MIMAT0007884	21572407

hsa-miR-190a-3p	MIMAT0026482	21572407
hsa-miR-190a-3p	MIMAT0026482	23446348
hsa-miR-3606-3p	MIMAT0022965	23446348
hsa-miR-182-5p	MIMAT0000259	24335145
hsa-miR-33a-3p	MIMAT0004506	20371350
hsa-miR-374a-5p	MIMAT0000727	21572407
hsa-miR-1185-2-3p	MIMAT0022713	21572407
hsa-miR-6083	MIMAT0023708	23446348
hsa-miR-5011-5p	MIMAT0021045	23313552
hsa-miR-409-3p	MIMAT0001639	20371350
hsa-miR-410-3p	MIMAT0002171	21572407
hsa-miR-1185-1-3p	MIMAT0022838	21572407
hsa-miR-1277-5p	MIMAT0022724	23313552
hsa-miR-5692c	MIMAT0022476	21572407
hsa-miR-4773	MIMAT0019928	20371350
hsa-miR-1258	MIMAT0005909	21572407
hsa-miR-3162-5p	MIMAT0015036	20371350
hsa-miR-143-5p	MIMAT0004599	21572407
hsa-miR-1258	MIMAT0005909	20371350
hsa-miR-3944-5p	MIMAT0019231	21572407
hsa-miR-143-5p	MIMAT0004599	20371350
hsa-miR-3175	MIMAT0015052	21572407
hsa-miR-3944-5p	MIMAT0019231	20371350
hsa-miR-4747-5p	MIMAT0019882	21572407
hsa-miR-3175	MIMAT0015052	20371350
hsa-miR-5196-5p	MIMAT0021128	21572407
hsa-miR-4747-5p	MIMAT0019882	20371350
hsa-miR-3202	MIMAT0015089	21572407
hsa-miR-5196-5p	MIMAT0021128	20371350
hsa-miR-4771	MIMAT0019925	21572407
hsa-miR-3202	MIMAT0015089	20371350
hsa-miR-3166	MIMAT0015040	21572407
hsa-miR-4771	MIMAT0019925	20371350
hsa-miR-3179	MIMAT0015056	21572407
hsa-miR-3166	MIMAT0015040	20371350
hsa-miR-873-5p	MIMAT0004953	21572407
hsa-miR-3179	MIMAT0015056	20371350
hsa-miR-6822-5p	MIMAT0027544	21572407
hsa-miR-873-5p	MIMAT0004953	20371350
hsa-miR-6750-5p	MIMAT0027400	21572407
hsa-miR-6822-5p	MIMAT0027544	20371350
hsa-miR-23a-3p	MIMAT0000078	22771720
hsa-miR-5584-5p	MIMAT0022283	21572407
hsa-miR-6750-5p	MIMAT0027400	20371350
hsa-miR-5584-5p	MIMAT0022283	20371350
hsa-miR-1273h-3p	MIMAT0030416	21572407
hsa-miR-92a-3p	MIMAT0000092	23622248

hsa-miR-576-3p	MIMAT0004796	21572407
hsa-miR-576-3p	MIMAT0004796	20371350
hsa-miR-3162-5p	MIMAT0015036	21572407
hsa-miR-4773	MIMAT0019928	21572407
hsa-miR-1273h-3p	MIMAT0030416	20371350
hsa-miR-485-5p	MIMAT0002175	23313552
hsa-miR-125a-5p	MIMAT0000443	23622248
hsa-miR-6087	MIMAT0023712	23313552
hsa-miR-3197	MIMAT0015082	23313552
hsa-miR-30a-5p	MIMAT0000087	22473208
hsa-miR-4784	MIMAT0019948	23313552
hsa-miR-30e-5p	MIMAT0000692	22473208
hsa-miR-3150b-3p	MIMAT0018194	23313552
hsa-miR-4524a-3p	MIMAT0019063	23313552
hsa-miR-30d-5p	MIMAT0000245	22473208
hsa-miR-7162-3p	MIMAT0028235	23313552
hsa-miR-30c-5p	MIMAT0000244	22473208
hsa-miR-4649-3p	MIMAT0019712	23313552
hsa-miR-30b-5p	MIMAT0000420	22473208
hsa-miR-330-5p	MIMAT0004693	23313552
hsa-miR-6765-5p	MIMAT0027430	23313552
hsa-miR-30a-5p	MIMAT0000087	18668040
hsa-miR-2861	MIMAT0013802	23313552
hsa-miR-5787	MIMAT0023252	23313552
hsa-miR-130b-3p	MIMAT0000691	20371350
hsa-miR-4505	MIMAT0019041	23313552
hsa-miR-103a-3p	MIMAT0000101	20371350
hsa-miR-6722-5p	MIMAT0025853	23313552
hsa-miR-93-5p	MIMAT0000093	20371350
hsa-miR-765	MIMAT0003945	23313552
hsa-miR-19b-3p	MIMAT0000074	20371350
hsa-miR-6884-5p	MIMAT0027668	23313552
hsa-miR-16-5p	MIMAT0000069	22473208
hsa-miR-4718	MIMAT0019831	23824327
hsa-miR-10b-5p	MIMAT0000254	23446348
hsa-miR-101-3p	MIMAT0000099	20371350
hsa-miR-15b-5p	MIMAT0000417	22473208
hsa-miR-1537-5p	MIMAT0026765	23824327
hsa-miR-339-5p	MIMAT0000764	23446348
hsa-miR-18a-5p	MIMAT0000072	20371350
hsa-miR-195-5p	MIMAT0000461	22473208
hsa-miR-4742-3p	MIMAT0019873	23824327
hsa-miR-548a-3p	MIMAT0003251	23446348
hsa-miR-532-3p	MIMAT0004780	23824327
hsa-miR-616-3p	MIMAT0004805	23446348
hsa-miR-4301	MIMAT0016850	23824327
hsa-miR-548e-3p	MIMAT0005874	23446348

hsa-miR-215-5p	MIMAT0000272	19074876
hsa-miR-483-3p	MIMAT0002173	23824327
hsa-miR-548f-3p	MIMAT0005895	23446348
hsa-miR-192-5p	MIMAT0000222	19074876
hsa-miR-32-5p	MIMAT0000090	22473208
hsa-miR-1250-3p	MIMAT0026740	23446348
hsa-miR-375	MIMAT0000728	20215506
hsa-miR-92a-3p	MIMAT0000092	22473208
hsa-miR-548ar-3p	MIMAT0022266	23446348
hsa-miR-92b-3p	MIMAT0003218	22473208
hsa-miR-105-5p	MIMAT0000102	23824327
hsa-miR-5582-3p	MIMAT0022280	23446348
hsa-miR-17-5p	MIMAT0000070	22473208
hsa-miR-7853-5p	MIMAT0030428	23824327
hsa-miR-548az-3p	MIMAT0025457	23446348
hsa-miR-20a-5p	MIMAT0000075	22473208
hsa-miR-607	MIMAT0003275	23824327
hsa-miR-6732-3p	MIMAT0027366	23446348
hsa-miR-93-5p	MIMAT0000093	22473208
hsa-miR-3156-3p	MIMAT0019209	23824327
hsa-miR-6771-3p	MIMAT0027443	23446348
hsa-miR-106b-5p	MIMAT0000680	22473208
hsa-miR-3671	MIMAT0018094	23824327
hsa-miR-136-5p	MIMAT0000448	23446348
hsa-miR-20b-5p	MIMAT0001413	22473208
hsa-miR-630	MIMAT0003299	23824327
hsa-miR-885-5p	MIMAT0004947	23446348
hsa-miR-519d-3p	MIMAT0002853	22473208
hsa-miR-100-5p	MIMAT0000098	23622248
hsa-miR-15a-5p	MIMAT0000068	22473208
hsa-miR-4999-5p	MIMAT0021017	23824327
hsa-miR-10a-5p	MIMAT0000253	23446348
hsa-miR-33a-5p	MIMAT0000091	23622248
hsa-miR-223-3p	MIMAT0000280	22815788
hsa-miR-7-5p	MIMAT0000252	17612493
hsa-miR-193b-3p	MIMAT0002819	20304954
hsa-miR-155-5p	MIMAT0000646	18668040
hsa-miR-155-5p	MIMAT0000646	20584899
hsa-miR-744-5p	MIMAT0004945	23622248
hsa-miR-18a-3p	MIMAT0002891	23622248
hsa-miR-92a-3p	MIMAT0000092	23622248
hsa-miR-155-5p	MIMAT0000646	23807165

List of 402 miRNAs targeting genes (N=17) involved in migration after removing duplicates

hsa-miR-557
hsa-miR-19b-3p
hsa-miR-1295a
hsa-miR-937-5p
hsa-let-7b-5p
hsa-miR-191-3p
hsa-miR-548l
hsa-miR-6507-3p
hsa-miR-5681a
hsa-miR-7-2-3p
hsa-miR-507
hsa-miR-19a-3p
hsa-miR-744-5p
hsa-miR-1200
hsa-let-7a-5p
hsa-miR-329-5p
hsa-miR-548n
hsa-miR-7-1-3p
hsa-miR-450b-5p
hsa-miR-5010-5p
hsa-miR-455-3p
hsa-miR-5702
hsa-miR-3176
hsa-miR-4464
hsa-miR-506-3p
hsa-miR-145-5p
hsa-miR-548t-3p
hsa-miR-33b-5p
hsa-miR-4525
hsa-miR-93-3p
hsa-miR-544b
hsa-miR-544a
hsa-miR-4748
hsa-miR-124-3p
hsa-miR-548aa
hsa-miR-33a-5p
hsa-miR-7111-5p
hsa-miR-484
hsa-miR-4324
hsa-miR-6772-5p
hsa-miR-3912-3p
hsa-miR-6738-3p
hsa-miR-548ap-3p
hsa-miR-382-3p
hsa-miR-6870-5p
hsa-miR-324-5p

hsa-miR-4328
hsa-let-7i-3p
hsa-miR-412-3p
hsa-miR-4289
hsa-miR-5698
hsa-miR-326
hsa-miR-3613-3p
hsa-miR-4477a
hsa-miR-5572
hsa-miR-4723-5p
hsa-miR-378a-3p
hsa-miR-605-5p
hsa-miR-432-3p
hsa-miR-4699-5p
hsa-miR-4260
hsa-miR-644a
hsa-miR-6876-3p
hsa-miR-320a
hsa-miR-548z
hsa-miR-525-3p
hsa-miR-450a-2-3p
hsa-miR-626
hsa-miR-222-3p
hsa-miR-548h-3p
hsa-miR-493-5p
hsa-miR-3611
hsa-miR-524-3p
hsa-miR-520h
hsa-miR-221-3p
hsa-miR-548d-3p
hsa-miR-127-5p
hsa-miR-4282
hsa-miR-6803-5p
hsa-miR-520g-3p
hsa-miR-196a-5p
hsa-miR-548ac
hsa-miR-493-3p
hsa-miR-4698
hsa-miR-6751-5p
hsa-miR-3973
hsa-miR-100-5p
hsa-miR-5195-3p
hsa-miR-8063
hsa-miR-192-3p
hsa-miR-7109-5p
hsa-miR-218-5p
hsa-miR-6510-3p

hsa-miR-92a-3p
hsa-miR-6870-3p
hsa-miR-4665-5p
hsa-miR-1307-3p
hsa-miR-3529-3p
hsa-miR-16-5p
hsa-miR-6872-3p
hsa-miR-3680-3p
hsa-miR-1275
hsa-miR-1303
hsa-let-7c-5p
hsa-miR-216a-5p
hsa-miR-708-5p
hsa-miR-378c
hsa-miR-26b-5p
hsa-miR-27a-5p
hsa-miR-378b
hsa-miR-133b
hsa-miR-4638-3p
hsa-miR-105-5p
hsa-miR-6824-3p
hsa-miR-143-3p
hsa-miR-6764-3p
hsa-miR-149-3p
hsa-miR-193b-3p
hsa-miR-155-5p
hsa-miR-199a-3p
hsa-miR-330-5p
hsa-miR-451a
hsa-miR-496
hsa-miR-3191-5p
hsa-miR-125b-5p
hsa-miR-4742-3p
hsa-miR-6873-3p
hsa-miR-6817-3p
hsa-miR-302a-3p
hsa-miR-654-3p
hsa-miR-6503-3p
hsa-miR-185-5p
hsa-miR-518c-5p
hsa-miR-302b-3p
hsa-miR-4757-3p
hsa-miR-422a
hsa-miR-7110-3p
hsa-miR-302c-3p
hsa-miR-365b-3p
hsa-miR-378i

hsa-miR-6866-3p
hsa-miR-302d-3p
hsa-miR-365a-3p
hsa-miR-378h
hsa-miR-188-5p
hsa-miR-378f
hsa-miR-625-3p
hsa-miR-378e
hsa-miR-378d
hsa-miR-3620-3p
hsa-miR-548ar-3p
hsa-miR-6073
hsa-miR-3646
hsa-miR-4495
hsa-miR-101-3p
hsa-miR-548a-3p
hsa-miR-3132
hsa-miR-106b-5p
hsa-let-7a-2-3p
hsa-miR-3120-3p
hsa-miR-583
hsa-miR-1238-3p
hsa-miR-197-5p
hsa-miR-106a-5p
hsa-let-7g-3p
hsa-miR-551b-5p
hsa-miR-1276
hsa-miR-670-3p
hsa-miR-3908
hsa-miR-20a-5p
hsa-miR-4484
hsa-miR-4311
hsa-miR-6881-3p
hsa-miR-522-3p
hsa-miR-17-5p
hsa-miR-298
hsa-miR-424-3p
hsa-miR-297
hsa-miR-7111-3p
hsa-miR-224-3p
hsa-miR-15a-5p
hsa-miR-490-5p
hsa-miR-3924
hsa-miR-6780a-3p
hsa-miR-130a-3p
hsa-miR-545-5p
hsa-miR-190a-3p

hsa-miR-664b-3p
hsa-let-7d-5p
hsa-miR-3688-3p
hsa-miR-5011-5p
hsa-miR-532-3p
hsa-miR-579-3p
hsa-miR-3662
hsa-miR-567
hsa-miR-500a-5p
hsa-miR-5696
hsa-miR-15b-5p
hsa-miR-6838-5p
hsa-miR-944
hsa-miR-302d-5p
hsa-miR-6504-3p
hsa-miR-497-5p
hsa-miR-423-3p
hsa-miR-302b-5p
hsa-miR-4786-5p
hsa-miR-424-5p
hsa-miR-328-3p
hsa-miR-5582-3p
hsa-miR-4709-3p
hsa-miR-195-5p
hsa-miR-548f-3p
hsa-miR-5093
hsa-miR-144-3p
hsa-miR-548e-3p
hsa-miR-1229-5p
hsa-miR-520c-3p
hsa-miR-5584-5p
hsa-miR-1260b
hsa-miR-548az-3p
hsa-miR-3606-5p
hsa-miR-6502-3p
hsa-miR-376c-3p
hsa-miR-377-3p
hsa-miR-432-5p
hsa-miR-382-5p
hsa-miR-7703
hsa-miR-6776-5p
hsa-miR-6861-5p
hsa-miR-335-5p
hsa-miR-4793-3p
hsa-miR-3925-3p
hsa-miR-1273g-3p
hsa-miR-622

hsa-miR-508-5p
hsa-miR-8085
hsa-miR-4644
hsa-miR-6731-5p
hsa-miR-4306
hsa-miR-6133
hsa-miR-6130
hsa-miR-6129
hsa-miR-6127
hsa-miR-4510
hsa-miR-4419a
hsa-miR-6760-5p
hsa-miR-375
hsa-miR-6873-5p
hsa-miR-4448
hsa-miR-4458
hsa-miR-6895-3p
hsa-miR-4451
hsa-let-7i-5p
hsa-miR-6845-3p
hsa-miR-6799-5p
hsa-let-7g-5p
hsa-miR-136-5p
hsa-miR-6883-5p
hsa-let-7f-5p
hsa-miR-6785-5p
hsa-let-7e-5p
hsa-miR-545-3p
hsa-miR-4728-5p
hsa-miR-7106-5p
hsa-miR-6516-5p
hsa-miR-6780a-5p
hsa-miR-4749-5p
hsa-miR-4537
hsa-miR-6779-5p
hsa-miR-4706
hsa-miR-5186
hsa-miR-3689c
hsa-miR-6499-3p
hsa-miR-3192-5p
hsa-miR-3689b-3p
hsa-miR-6086
hsa-miR-6831-5p
hsa-miR-3689a-3p
hsa-miR-377-5p
hsa-miR-3927-3p
hsa-miR-30b-3p

hsa-miR-98-5p
hsa-miR-7851-3p
hsa-miR-1273h-5p
hsa-miR-4500
hsa-miR-490-3p
hsa-miR-4291
hsa-miR-615-3p
hsa-miR-582-3p
hsa-miR-582-5p
hsa-miR-26a-5p
hsa-miR-4789-5p
hsa-miR-374b-5p
hsa-miR-5692b
hsa-miR-199a-5p
hsa-miR-877-3p
hsa-miR-8064
hsa-miR-374a-5p
hsa-miR-513c-3p
hsa-miR-369-3p
hsa-miR-425-5p
hsa-miR-4643
hsa-miR-410-3p
hsa-miR-513a-3p
hsa-miR-1277-5p
hsa-miR-383-3p
hsa-miR-29b-3p
hsa-miR-5692c
hsa-miR-3606-3p
hsa-miR-3065-3p
hsa-miR-6083
hsa-let-7a-3p
hsa-miR-4465
hsa-miR-6787-3p
hsa-miR-409-5p
hsa-let-7b-3p
hsa-miR-548c-3p
hsa-miR-346
hsa-let-7f-1-3p
hsa-miR-1297
hsa-let-7f-2-3p
hsa-miR-5697
hsa-miR-98-3p
hsa-miR-5683
hsa-miR-183-5p
hsa-miR-1910-5p
hsa-miR-1185-2-3p
hsa-miR-33a-3p

hsa-miR-1185-1-3p
hsa-miR-409-3p
hsa-miR-96-5p
hsa-miR-182-5p
hsa-miR-4773
hsa-miR-1258
hsa-miR-3162-5p
hsa-miR-143-5p
hsa-miR-3944-5p
hsa-miR-3175
hsa-miR-4747-5p
hsa-miR-5196-5p
hsa-miR-3202
hsa-miR-4771
hsa-miR-3166
hsa-miR-3179
hsa-miR-873-5p
hsa-miR-6822-5p
hsa-miR-6750-5p
hsa-miR-23a-3p
hsa-miR-1273h-3p
hsa-miR-576-3p
hsa-miR-485-5p
hsa-miR-125a-5p
hsa-miR-6087
hsa-miR-3197
hsa-miR-30a-5p
hsa-miR-4784
hsa-miR-30e-5p
hsa-miR-3150b-3p
hsa-miR-4524a-3p
hsa-miR-30d-5p
hsa-miR-7162-3p
hsa-miR-30c-5p
hsa-miR-4649-3p
hsa-miR-30b-5p
hsa-miR-6765-5p
hsa-miR-2861
hsa-miR-5787
hsa-miR-130b-3p
hsa-miR-4505
hsa-miR-103a-3p
hsa-miR-6722-5p
hsa-miR-93-5p
hsa-miR-765
hsa-miR-6884-5p
hsa-miR-4718

hsa-miR-10b-5p
hsa-miR-1537-5p
hsa-miR-339-5p
hsa-miR-18a-5p
hsa-miR-616-3p
hsa-miR-4301
hsa-miR-215-5p
hsa-miR-483-3p
hsa-miR-192-5p
hsa-miR-32-5p
hsa-miR-1250-3p
hsa-miR-92b-3p
hsa-miR-7853-5p
hsa-miR-607
hsa-miR-6732-3p
hsa-miR-3156-3p
hsa-miR-6771-3p
hsa-miR-3671
hsa-miR-20b-5p
hsa-miR-630
hsa-miR-885-5p
hsa-miR-519d-3p
hsa-miR-4999-5p
hsa-miR-10a-5p
hsa-miR-223-3p
hsa-miR-7-5p
hsa-miR-18a-3p

No miRNA was found to target SOX10 and DAB1 genes
