# Supplementary material for: An in-silico approach for discovery of microRNA-TF regulation of DISC1 interactome mediating neuronal migration
Source: NPJ Syst Biol Appl. 2019 May 7;5:17. doi: 10.1038/s41540-019-0094-3 (PMC6504871; doi:10.1038/s41540-019-0094-3)
Supplement: Supplementary file 3 — Supplementary Table S3 [file 41540_2019_94_MOESM3_ESM.pdf]

TF	PMID	Study	Cell Type	Organism	Overlap
STAT3	23295773	ChIP-Seq	U87	Human	10/3165
KLF4	18358816	ChIP-ChIP	MESCs	Mouse	7/1700
SMAD2	18955504	ChIP-ChIP	HaCaT	Human	7/1936
SMAD3	18955504	ChIP-ChIP	HaCaT	Human	7/1936
EGR1	19374776	ChIP-ChIP	THP-1	Human	2/1968
CCND1	20090754	ChIP-ChIP	RETINA	Mouse	7/2137
HNF4A	19822575	ChIP-Seq	HepG2	Human	12/6083
CREB1	20920259	ChIP-Seq	GC1-SPG	Mouse	8/3057
TRIM28	19339689	ChIP-ChIP	MESCs	Mouse	8/3072
CLOCK	20551151	ChIP-Seq	293T	Human	3/407
TCF3	18467660	ChIP-ChIP	MESCs	Mouse	5/1388
RING1B	27294783	Chip-Seq	ESCs	Mouse	6/2000
SOX3	22085726	ChIP-Seq	MUSCLE	Mouse	6/2000
ZFP281	18757296	ChIP-ChIP	E14	Mouse	6/2004
ELK1	22589737	ChIP-Seq	MCF10A	Human	4/928
TTF2	22483619	ChIP-Seq	HELA	Human	5/1512
MEF2A	21415370	ChIP-Seq	HL-1	Mouse	4/1048
NUCKS1	24931609	ChIP-Seq	HEPATOCYTES	Mouse	3/588
YAP1	20516196	ChIP-Seq	MESCs	Mouse	6/2329
CREB1	23762244	ChIP-Seq	HIPPOCAMPUS	Rat	6/2393
SCL	21571218	ChIP-Seq	MEGAKARYOCYTES	Human	5/1784
RNF2	16625203	ChIP-ChIP	MESCs	Mouse	4/1219
AR	22383394	ChIP-Seq	PROSTATE CANCER	Human	5/1857
TAL1	20566737	ChIP-Seq	PRIMARY FETAL LIVER ERYTHROID	Mouse	5/1875
PIAS1	25552417	ChIP-Seq	VCAP	Human	3/749
HCFC1	20581084	ChIP-Seq	MESCs	Mouse	2/306
SIN3B	21632747	ChIP-Seq	MESCs	Mouse	8/4302
SMC4	20622854	ChIP-Seq	HELA	Human	5/2000
TAL1	26923725	Chip-Seq	HPCs	Mouse	5/2000
RXR	22158963	ChIP-Seq	LIVER	Mouse	5/2000
SPI1	23127762	ChIP-Seq	K562	Human	4/1389
POU5F1	18347094	ChIP-ChIP	MESCs	Mouse	5/2109
EZH2	27304074	Chip-Seq	ESCs	Mouse	3/885
MYBL2	22936984	ChIP-ChIP	MESCs	Mouse	5/2250
PHC1	16625203	ChIP-ChIP	MESCs	Mouse	3/922
SRY	25088423	ChIP-ChIP	EMBRYONIC GONADS	Mouse	6/3083
CTNNB1	20460455	ChIP-Seq	HCT116	Human	3/988
WT1	20215353	ChIP-ChIP	NEPHRON PROGENITOR	Mouse	4/1663
DROSHA	22980978	ChIP-Seq	HELA	Human	2/456
NR0B1	18358816	ChIP-ChIP	MESCs	Mouse	4/1691
PRDM5	23873026	ChIP-Seq	MEFs	Mouse	3/1029
POU3F2	20337985	ChIP-ChIP	501MEL	Human	4/1702
PADI4	21655091	ChIP-ChIP	MCF-7	Human	3/1037
ZFX	18555785	ChIP-Seq	MESCs	Mouse	6/3249
SALL4	18804426	ChIP-ChIP	MESCs	Mouse	3/1062
PAX3-FKHR	20663909	ChIP-Seq	RHABDOMYOSARCOMA	Human	3/1063

ELF5	23300383	ChIP-Seq	T47D	Human	3/1087
ZFP322A	24550733	ChIP-Seq	MESCs	Mouse	1/1990
SMARCA4	20176728	ChIP-ChIP	TSCs	Mouse	3/1118
SUZ12	18692474	ChIP-Seq	MEFs	Mouse	3/1135
GATA1	19941827	ChIP-Seq	MEL	Mouse	4/1834
KLF5	18264089	ChIP-ChIP	MESCs	Mouse	1/103
KLF2	18264089	ChIP-ChIP	MESCs	Mouse	1/103
KLF4	18264089	ChIP-ChIP	MESCs	Mouse	1/104
GABP	19822575	ChIP-Seq	HepG2	Human	5/2639
POU5F1	18700969	ChIP-ChIP	MESCs	Mouse	2/567
SUZ12	18692474	ChIP-Seq	MESCs	Mouse	4/1909
MEIS1	26923725	Chip-Seq	HEMOGENIC-ENDOTHELIUM	Mouse	3/1196
AR	19668381	ChIP-Seq	PC3	Human	6/3519
ZFP281	18358816	ChIP-ChIP	MESCs	Mouse	2/578
SUZ12	18974828	ChIP-Seq	MESCs	Mouse	4/1934
PRDM14	21183938	ChIP-Seq	MESCs	Mouse	4/1944
FOXA1	27197147	Chip-Seq	ENDOMETRIOID-ADENOCARCINOMA	Human	2/594
SPI1	20517297	ChIP-Seq	HL60	Human	3/1249
OCT4	18692474	ChIP-Seq	MEFs	Mouse	4/1992
CJUN	26792858	Chip-Seq	BT549	Human	4/2000
STAT1	17558387	ChIP-Seq	HELA	Human	4/2000
TBL1	22424771	ChIP-Seq	293T	Human	4/2000
CBX2	22325352	ChIP-Seq	293T-Rex	Human	4/2000
ETV1	20927104	ChIP-Seq	GIST48	Human	4/2000
GATA2	21186366	ChIP-Seq	BM-HSCs	Mouse	4/2000
CDX2	21074721	ChIP-Seq	CACO-2	Mouse	4/2000
NFYB	21822215	ChIP-Seq	K562	Human	4/2000
CSB	26484114	Chip-Seq	FIBROBLAST	Human	4/2000
P300	19829295	ChIP-Seq	ESCs	Human	4/2000
LUZP1	20508642	ChIP-Seq	ESCs	Mouse	4/2000
KLF5	25053715	ChIP-Seq	YYC3	Human	4/2000
MAF	26560356	Chip-Seq	TH2	Human	4/2000
PCGF4	22325352	ChIP-Seq	293T-Rex	Human	4/2000
SMRT	22465074	ChIP-Seq	MACROPHAGES	Mouse	4/2000
HNFA	21074721	ChIP-Seq	CACO-2	Human	4/2000
JARID1B-DAIN	22020125	ChIP-Seq	ESCs	Mouse	4/2000
NEUROD2	26341353	ChIP-Seq	CORTEX	Mouse	4/2000
CHD1	26751641	Chip-Seq	LNCaP	Human	4/2000
NFYA	21822215	ChIP-Seq	K562	Human	4/2000
PU.1	20513432	ChIP-Seq	MACROPHAGES	Mouse	4/2000
NCOR	22465074	ChIP-Seq	MACROPHAGES	Mouse	4/2000
PHF8	20622853	ChIP-Seq	HELA	Human	4/2000
ZFP281	27345836	Chip-Seq	ESCs	Mouse	4/2000
OLIG2	26023283	ChIP-Seq	AINV15	Mouse	4/2000
NFI	21473784	ChIP-Seq	ESCs	Mouse	4/2000
MYB	26560356	Chip-Seq	TH2	Human	4/2000
CREB1	26743006	Chip-Seq	LNCaP-abl	Human	4/2000

TAF2	19829295	ChIP-Seq	ESCs	Human	4/2000
FOXM1	26456572	ChIP-Seq	MCF-7	Human	4/2000
KAP1	27257070	Chip-Seq	ESCs	Mouse	4/2000
EBNA1	20929547	Chip-Seq	RAJI-cells	Human	4/2000
KLF4	26769127	Chip-Seq	PDAC-Cell line	Human	4/2000
MEIS1	26253404	ChIP-Seq	OPTIC CUPS	Mouse	4/2000
ELF3	26769127	Chip-Seq	PDAC-Cell line	Human	4/2000
SUZ12	16625203	ChIP-ChIP	MESCs	Mouse	3/1270
VDR	22108803	ChIP-Seq	LS180	Human	1/130
SOX2	16153702	ChIP-ChIP	HESCs	Human	3/1278
NOTCH1	17114293	ChIP-ChIP	T-ALL	Human	1/134
OLIG2	23332759	ChIP-Seq	OLIGODENDROCYTES	Mouse	4/2040
RNF2	18974828	ChIP-Seq	MESCs	Mouse	3/1302
EZH2	18974828	ChIP-Seq	MESCs	Mouse	3/1302
CEBPB	26923725	Chip-Seq	HEMANGIOBLAST	Mouse	2/652
DMRT1	23473982	ChIP-Seq	TESTES	Mouse	4/2072
BMI1	19503595	ChIP-Seq	MEFsC	Mouse	2/661
MITF	21258399	ChIP-Seq	MELANOMA	Human	8/5578
SOX9	24532713	ChIP-Seq	HFSC	Mouse	3/1384
MYC	19079543	ChIP-ChIP	MESCs	Mouse	3/1458
THRA	23701648	ChIP-Seq	CEREBELLUM	Mouse	1/179
RNF2	27304074	Chip-Seq	ESCs	Mouse	3/1467
FLI1	21571218	ChIP-Seq	MEGAKARYOCYTES	Human	8/5834
AR	21572438	ChIP-Seq	LNCaP	Human	1/188
NACC1	18358816	ChIP-ChIP	MESCs	Mouse	2/769
KLF4	19030024	ChIP-ChIP	MESCs	Mouse	3/1502
SOX2	18358816	ChIP-ChIP	MESCs	Mouse	2/785
HOXD13	18407260	ChIP-ChIP	DEVELOPING-LIMBS	Mouse	1/196
VDR	21846776	ChIP-Seq	THP-1	Human	2/794
XRN2	22483619	ChIP-Seq	HELA	Human	3/1529
TP53	23651856	ChIP-Seq	MEFs	Mouse	5/3193
CNOT3	19339689	ChIP-ChIP	MESCs	Mouse	3/1547
TP53	18474530	ChIP-ChIP	U2OS	Human	2/827
ISL1	27105846	Chip-Seq	CPCs	Mouse	3/1576
ETS2	20176728	ChIP-ChIP	TROPHOBLAST STEM CELLS	Mouse	1/215
GATA3	20176728	ChIP-ChIP	TSCs	Mouse	2/834
PPARG	23326641	ChIP-Seq	C3H10T1-2	Mouse	2/838
CHD1	19587682	ChIP-ChIP	MESCs	Mouse	2/843
EWS	26573619	Chip-Seq	HEK293	Human	2/854
KLF4	18555785	ChIP-Seq	MESCs	Mouse	4/2444
ESR1	21235772	ChIP-Seq	MCF-7	Human	1/228
SOX2	19030024	ChIP-ChIP	MESCs	Mouse	2/863
FOXP2	23625967	ChIP-Seq	PFSK-1 AND SK-N-MC	Human	2/863
THAP11	20581084	ChIP-Seq	MESCs	Mouse	2/864
SMAD4	21799915	ChIP-Seq	A2780	Human	4/2464
TAF15	26573619	Chip-Seq	HEK293	Human	1/247
SMARCA4	23332759	ChIP-Seq	OLIGODENDROCYTES	Mouse	4/2522

TAF7L	23326641	ChIP-Seq	C3H10T1-2	Mouse	2/912
ELK1	19687146	ChIP-ChIP	HELA	Human	2/916
SOX9	26525672	Chip-Seq	HEART	Mouse	3/1702
SOX2	20726797	ChIP-Seq	SW620	Human	4/2564
FOXM1	25889361	ChIP-Seq	OE33 AND U2OS	Human	2/932
EOMES	21245162	ChIP-Seq	HESCs	Human	2/932
WT1	25993318	ChIP-Seq	PODOCYTE	Human	5/3464
E2F1	17053090	ChIP-ChIP	MCF-7	Human	3/1726
NR1I2	20693526	ChIP-Seq	LIVER	Mouse	2/939
EOMES	20176728	ChIP-ChIP	TSCs	Mouse	3/1744
GATA1	21571218	ChIP-Seq	MEGAKARYOCYTES	Human	4/2601
GATA1	19941826	ChIP-Seq	K562	Human	2/967
STAT3	24763339	ChIP-Seq	IMN-ESCs	Mouse	3/1788
GBX2	23144817	ChIP-Seq	PC3	Human	1/286
PPARG	20176806	ChIP-Seq	3T3-L1	Mouse	3/1807
CBP	20019798	ChIP-Seq	JUKART	Human	2/1000
IRF4	20064451	ChIP-Seq	CD4+T	Mouse	2/1000
EST1	17652178	ChIP-ChIP	JURKAT	Human	2/1001
LMO2	26923725	Chip-Seq	HEMANGIOBLAST	Mouse	3/1822
HOXB4	20404135	ChIP-ChIP	EML	Mouse	3/1824
EP300	21415370	ChIP-Seq	HL-1	Mouse	2/1014
SUZ12	18555785	ChIP-Seq	MESCs	Mouse	2/1058
TFAP2A	17053090	ChIP-ChIP	MCF-7	Human	3/1904
KDM5B	21448134	ChIP-Seq	MESCs	Mouse	5/3724
CDX2	19796622	ChIP-Seq	MESCs	Mouse	1/334
FOXM1	26100407	CHIP-SEQ	Hek293 flp-in	Human	3/1934
SREBP2	21459322	ChIP-Seq	LIVER	Mouse	2/1095
SOX17	20123909	ChIP-Seq	XEN	Mouse	3/1947
NANOG	18700969	ChIP-ChIP	MESCs	Mouse	1/344
JARID2	20064375	ChIP-Seq	MESCs	Mouse	2/1117
TCF4	23295773	ChIP-Seq	U87	Human	5/3812
TP53	20018659	ChIP-ChIP	R1E	Mouse	2/1122
TCFCP2L1	18555785	ChIP-Seq	MESCs	Mouse	3/1987
SOX2	18692474	ChIP-Seq	MEFs	Mouse	3/1991
TET1	21490601	ChIP-Seq	MESCs	Mouse	3/1994
NELFA	20434984	ChIP-Seq	ESCs	Mouse	3/2000
FLI1	21867929	ChIP-Seq	TH2	Mouse	3/2000
GATA3	22897851	ChIP-Seq	JUKARTE6-1	Human	3/2000
KDM2B	26808549	Chip-Seq	REH	Human	3/2000
RUNX1	22897851	ChIP-Seq	JUKARTE6-1	Human	3/2000
P63	20808887	ChIP-Seq	KERATINOCYTES	Human	3/2000
LXR	22292898	ChIP-Seq	THP-1	Human	3/2000
TAL1	21186366	ChIP-Seq	BM-HSCs	Mouse	3/2000
TBX20	22328084	ChIP-Seq	HEART	Mouse	3/2000
RBPJ	21746931	ChIP-Seq	IB4	Human	3/2000
ESET	19884257	ChIP-Seq	ESCs	Mouse	3/2000
SMAD3	21741376	ChIP-Seq	ESCs	Human	3/2000

ETS1	21867929	ChIP-Seq	TH2	Mouse	3/2000
SPI1	26923725	Chip-Seq	HPCs	Mouse	3/2000
CTCF	27219007	Chip-Seq	Bcells	Human	3/2000
TBX20	22080862	ChIP-Seq	HEART	Mouse	3/2000
BCL6	25482012	ChIP-Seq	CML-JURL-MK1	Human	3/2000
FOXH1	21741376	ChIP-Seq	ESCs	Human	3/2000
FOXA1	26743006	Chip-Seq	LNCaP-abl	Human	3/2000
CEBPB	26923725	Chip-Seq	HEMOGENIC-ENDOTHELIUM	Mouse	3/2000
DPY	21335234	ChIP-Seq	ESCs	Mouse	3/2000
KLF5	20875108	ChIP-Seq	MESCs	Mouse	3/2000
PU.1	20513432	ChIP-Seq	Bcells	Mouse	3/2000
UBF1/2	26484160	Chip-Seq	HMEC-DERIVED	Human	3/2000
GATA4	25053715	ChIP-Seq	YYC3	Human	3/2000
AR	21915096	ChIP-Seq	LNCaP-1F5	Human	3/2000
GATA6	21074721	ChIP-Seq	CACO-2	Mouse	3/2000
FOXH1	21741376	ChIP-Seq	EPCs	Human	3/2000
CREB1	26743006	Chip-Seq	LNCaP	Human	3/2000
E2F1	18555785	Chip-Seq	ESCs	Mouse	3/2000
SMC1	22415368	ChIP-Seq	MEFs	Mouse	3/2000
RARB	27405468	Chip-Seq	BRAIN	Mouse	3/2000
FLI1	26923725	Chip-Seq	HEMOGENIC-ENDOTHELIUM	Mouse	3/2000
GATA6	21074721	ChIP-Seq	CACO-2	Human	3/2000
KDM2B	26808549	Chip-Seq	K562	Human	3/2000
CTCF	27219007	Chip-Seq	ERYTHROID	Human	3/2000
OCT4	21477851	ChIP-Seq	ESCs	Mouse	3/2000
KDM2B	26808549	Chip-Seq	SIL-ALL	Human	3/2000
SMC3	22415368	ChIP-Seq	MEFs	Mouse	3/2000
FLI1	26923725	Chip-Seq	MACROPHAGESS	Mouse	3/2000
SOX2	27498859	Chip-Seq	STOMACH	Mouse	3/2000
CREM	20920259	ChIP-Seq	GC1-SPG	Mouse	7/5776
ZIC3	20872845	ChIP-ChIP	MESCs	Mouse	1/365
MYC	19030024	ChIP-ChIP	MESCs	Mouse	5/3868
HSF1	23293686	ChIP-Seq	STHDH STRIATAL	Mouse	2/1156
FOXP2	21765815	ChIP-ChIP	NEURO2A	Mouse	2/1164
MTF2	20144788	ChIP-Seq	MESCs	Mouse	4/2981
TAL1	20887958	ChIP-Seq	HPC-7	Mouse	3/2067
TFAP2C	20629094	ChIP-Seq	MCF-7	Human	2/1203
RCOR2	21632747	ChIP-Seq	MESCs	Mouse	1/401
EP300	20729851	ChIP-Seq	FORBRAIN MIDBRAIN LIMB HEART	Mouse	3/2093
KLF4	25985364	ChIP-Seq	ATHEROSCLEROSIS LESION	Mouse	2/1211
SMAD4	19686287	ChIP-ChIP	HaCaT	Human	1/405
REST	19997604	ChIP-ChIP	NEURONS	Mouse	3/2118
SMARCD1	25818293	ChIP-Seq	ESCs	Mouse	3/2119
CUX1	19635798	ChIP-ChIP	MULTIPLE HUMAN CANCER TYPES	Human	4/3052
NANOG	18358816	ChIP-ChIP	MESCs	Mouse	2/1232
ESR2	21235772	ChIP-Seq	MCF-7	Human	1/424
JARID2	20075857	ChIP-Seq	MESCs	Mouse	2/1258

ESR1	17901129	ChIP-ChIP	LIVER	Mouse	1/444
VDR	24763502	ChIP-Seq	THP-1	Human	2/1288
TP53	16413492	ChIP-PET	HCT116	Human	1/449
TCF3	18347094	ChIP-ChIP	MESCs	Mouse	3/2221
CIITA	25753668	ChIP-Seq	RAJI	Human	1/459
YY1	21170310	ChIP-Seq	MESCs	Mouse	1/464
SPI1	23547873	ChIP-Seq	NB4	Human	4/3198
E2F1	18555785	ChIP-Seq	MESCs	Mouse	5/4172
TCF4	18268006	ChIP-ChIP	LS174T	Human	1/470
LMO2	26923725	Chip-Seq	MACROPHAGESS	Mouse	1/473
EGR1	20690147	ChIP-Seq	ERYTHROLEUKEMIA	Human	7/6207
TP63	22573176	ChIP-Seq	HFKS	Human	5/4229
TEAD4	22529382	ChIP-Seq	TROPHECTODERM	Mouse	3/2293
POU5F1	18692474	ChIP-Seq	MESCs	Mouse	5/4232
SOX2	18692474	ChIP-Seq	MESCs	Mouse	4/3319
MYC	20876797	ChIP-ChIP	MEDULLOBLASTOMA	Human	2/1406
TAL1	26923725	Chip-Seq	HEMANGIOBLAST	Mouse	2/1413
SFPI1	20887958	ChIP-Seq	HPC-7	Mouse	3/2369
NFE2L2	22581777	ChIP-Seq	LYMPHOBLASTOID	Human	1/527
SUZ12	20075857	ChIP-Seq	MESCs	Mouse	5/4356
NANOG	18555785	ChIP-Seq	MESCs	Mouse	1/542
FUS	26573619	Chip-Seq	HEK293	Human	1/543
YY1	23942234	ChIP-Seq	MYOBLASTS AND MYOTUBES	Mouse	2/1466
SOX2	21211035	ChIP-Seq	LN229	Gbm	4/3420
RXR	22108803	ChIP-Seq	LS180	Human	1/555
TEAD4	26923725	Chip-Seq	HEMANGIOBLAST	Mouse	1/559
POU5F1	26923725	Chip-Seq	MESODERM	Mouse	1/559
PPARD	21283829	ChIP-Seq	MYOFIBROBLAST	Human	4/3447
NFIB	24661679	ChIP-Seq	LUNG	Mouse	1/573
EWS-FLI1	20517297	ChIP-Seq	SK-N-MC	Human	1/574
ZNF217	24962896	ChIP-Seq	MCF-7	Human	2/1522
NANOG	16518401	ChIP-PET	MESCs	Mouse	4/3520
NR1H3	23393188	ChIP-Seq	ATHEROSCLEROTIC-FOAM	Human	1/599
CTCF	18555785	ChIP-Seq	MESCs	Mouse	2/1568
PPARG	20887899	ChIP-Seq	3T3-L1	Mouse	4/3565
TRIM28	17542650	ChIP-ChIP	NTERA2	Human	4/3568
POU5F1	16153702	ChIP-ChIP	HESCs	Human	1/622
CEBPB	20176806	ChIP-Seq	THIOMACROPHAGE	Mouse	2/1588
ETS1	20019798	ChIP-Seq	JURKAT	Human	2/1607
CTNNB1	20615089	ChIP-ChIP	FETAL BRAIN	Human	1/637
TP63	23658742	ChIP-Seq	EP156T	Human	4/3652
TDRD3	21172665	ChIP-Seq	MCF-7	Human	1/655
STAT1	20625510	ChIP-Seq	HELA	Human	1/656
SPI1	20176806	ChIP-Seq	THIOMACROPHAGE	Mouse	2/1654
TCFAP2C	20176728	ChIP-ChIP	TROPHOBLAST STEM CELLS	Mouse	3/2667
CRX	20693478	ChIP-Seq	ADULT RETINA	Mouse	1/668
PDX1	19855005	ChIP-ChIP	MIN6	Mouse	1/669

SPI1	22096565	ChIP-ChIP	GC-B	Mouse	2/1676
P53	22127205	ChIP-Seq	FIBROBLAST	Human	1/679
FOXP1	21924763	ChIP-Seq	HESCs	Human	4/3724
NANOG	16153702	ChIP-ChIP	HESCs	Human	2/1686
AHR	22903824	ChIP-Seq	MCF-7	Human	1/690
DACH1	20351289	ChIP-Seq	MDA-MB-231	Human	2/1698
FOXO3	23340844	ChIP-Seq	DLD1	Human	1/695
SREBP1	19666523	ChIP-Seq	LIVER	Mouse	1/738
POU5F1	18358816	ChIP-ChIP	MESCs	Mouse	1/753
DCP1A	22483619	ChIP-Seq	HELA	Human	1/759
RCOR3	21632747	ChIP-Seq	MESCs	Mouse	3/2851
IRF8	22096565	ChIP-ChIP	GC-B	Mouse	1/772
TET1	21451524	ChIP-Seq	MESCs	Mouse	2/1839
GFI1B	20887958	ChIP-Seq	HPC-7	Mouse	2/1871
TFEB	21752829	ChIP-Seq	HELA	Human	1/808
RUNX1	21571218	ChIP-Seq	MEGAKARYOCYTES	Human	5/5071
FOXA2	19822575	ChIP-Seq	HepG2	Human	3/2968
SOX9	25088423	ChIP-ChIP	EMBRYONIC GONADS	Mouse	2/1903
PPARG	20176806	ChIP-Seq	THIOMACROPHAGE	Mouse	1/837
NANOG	21062744	ChIP-ChIP	HESCs	Human	1/840
RUNX1	26923725	Chip-Seq	HPCs	Mouse	1/859
SPI1	22790984	ChIP-Seq	ERYTHROLEUKEMIA	Mouse	2/1962
NANOG	18692474	ChIP-Seq	MESCs	Mouse	3/3052
NANOG	18692474	ChIP-Seq	MEFs	Mouse	2/1989
STAT3	18555785	ChIP-Seq	MESCs	Mouse	2/1997
MYB	26560356	Chip-Seq	TH1	Human	2/2000
CEBPA	26348894	ChIP-Seq	LIVER	Mouse	2/2000
SOX2	21211035	ChIP-Seq	LN229	Human	2/2000
PHF8	20622854	ChIP-Seq	HELA	Human	2/2000
SMAD4	21741376	ChIP-Seq	EPCs	Human	2/2000
RACK7	27058665	Chip-Seq	MCF-7	Human	2/2000
SOX6	21985497	ChIP-Seq	MYOTUBES	Mouse	2/2000
RUNX1	27514584	Chip-Seq	MCF-7	Human	2/2000
KDM2B	26808549	Chip-Seq	JURKAT	Human	2/2000
MNX1	26342078	ChIP-Seq	MIN6-4N	Mouse	2/2000
GATA3	24758297	ChIP-Seq	MCF-7	Human	2/2000
TCFCP2L1	18555785	Chip-Seq	ESCs	Mouse	2/2000
ATF3	27146783	Chip-Seq	COLON	Human	2/2000
OCT4	20526341	ChIP-Seq	ESCs	Human	2/2000
SOX3	22085726	ChIP-Seq	NPCs	Mouse	2/2000
NANOG	20526341	ChIP-Seq	ESCs	Human	2/2000
CTCF	21964334	Chip-Seq	Bcells	Human	2/2000
SOX11	22085726	ChIP-Seq	ESNs	Mouse	2/2000
UBF1/2	26484160	Chip-Seq	FIBROBLAST	Human	2/2000
GATA3	26560356	Chip-Seq	TH2	Human	2/2000
RARA	24833708	ChIP-Seq	LIVER	Mouse	2/2000
P68	20966046	ChIP-Seq	HELA	Human	2/2000

KDM2B	26808549	Chip-Seq	HPB-ALL	Human	2/2000
NANOG	18555785	Chip-Seq	ESCs	Mouse	2/2000
PPARA	22158963	ChIP-Seq	LIVER	Mouse	2/2000
CEBPB	22108803	ChIP-Seq	LS180	Human	2/2000
SMAD4	21741376	ChIP-Seq	ESCs	Human	2/2000
FOXA1	25552417	ChIP-Seq	VCAP	Human	2/2000
TCF21	26020271	ChIP-Seq	SMOOTH MUSCLE	Human	2/2000
CMYC	18555785	Chip-Seq	ESCs	Mouse	2/2000
HOXB7	26014856	ChIP-Seq	BT474	Human	2/2000
KAP1	22055183	ChIP-Seq	ESCs	Mouse	2/2000
TCF7	22412390	ChIP-Seq	EML	Mouse	2/2000
JUN	26020271	ChIP-Seq	SMOOTH MUSCLE	Human	2/2000
FOXA1	21915096	ChIP-Seq	LNCaP-1F5	Human	2/2000
BCOR	27268052	Chip-Seq	Bcells	Human	2/2000
SMAD2/3	21741376	ChIP-Seq	EPCs	Human	2/2000
P63	26484246	Chip-Seq	KERATINOCYTES	Human	2/2000
PU.1	20176806	ChIP-Seq	MACROPHAGES	Mouse	2/2000
UBF1/2	26484160	Chip-Seq	HMECs	Human	2/2000
P300	27058665	Chip-Seq	ZR-75-30cells	Human	2/2000
SUZ12	27294783	Chip-Seq	ESCs	Mouse	2/2000
LXR	22158963	ChIP-Seq	LIVER	Mouse	2/2000
CEBPB	24764292	ChIP-Seq	MC3T3	Mouse	2/2000
P53	21459846	ChIP-Seq	SAOS-2	Human	2/2000
BRD4	27068464	Chip-Seq	AML-cells	Mouse	2/2000
CDX2	22108803	ChIP-Seq	LS180	Human	2/2000
PU	27001747	Chip-Seq	BMDM	Mouse	2/2000
CDX2	21402776	ChIP-Seq	INTESTINAL-VILLUS	Mouse	2/2000
GATA1	19941827	ChIP-Seq	MEL86	Mouse	2/2000
FLI1	21867929	ChIP-Seq	CD8	Mouse	2/2000
DNAJC2	21179169	ChIP-ChIP	NT2	Human	1/899
HOXC9	25013753	ChIP-Seq	NEUROBLASTOMA BE2-C	Human	2/2014
SETDB1	19884255	ChIP-Seq	MESCs	Mouse	2/2020
NR3C1	23031785	ChIP-Seq	PC12	Mouse	1/918
GATA4	21415370	ChIP-Seq	HL-1	Mouse	2/2039
MYB	21317192	ChIP-Seq	ERMYB	Mouse	1/923
EGR1	23403033	ChIP-Seq	LIVER	Mouse	1/944
TP53	22127205	ChIP-Seq	IMR90	Human	1/975
GRHL2	25758223	ChIP-Seq	PLACENTA	Mouse	1/1000
ETV2	25802403	ChIP-Seq	MESCs	Mouse	1/1000
AUTS2	25519132	ChIP-Seq	293T-REX	Human	1/1000
GABP	17652178	ChIP-ChIP	JURKAT	Human	1/1001
RUNX1	17652178	ChIP-ChIP	JURKAT	Human	1/1003
KDM5A	27292631	Chip-Seq	BREAST	Human	1/1012
SMAD3	22036565	ChIP-Seq	ESCs	Mouse	1/1020
TCF7L2	21901280	ChIP-Seq	H4IIE	Rat	1/1023
ARNT	22903824	ChIP-Seq	MCF-7	Human	1/1029
ASH2L	23239880	ChIP-Seq	MESCs	Mouse	3/3336

RNF2	27304074	Chip-Seq	NSC	Mouse	1/1052
VDR	23849224	ChIP-Seq	CD4+	Human	2/2231
BMI1	23680149	ChIP-Seq	NPCS	Mouse	1/1056
ERG	20517297	ChIP-Seq	VCAP	Human	1/1062
ZFP57	27257070	Chip-Seq	ESCs	Mouse	1/1088
TBX5	21415370	ChIP-Seq	HL-1	Mouse	2/2296
PBX1	22567123	ChIP-ChIP	OVCAR3	Human	2/2299
BRD4	25478319	ChIP-Seq	HGPS	Human	2/2326
HNF4A	19761587	ChIP-ChIP	CACO-2	Human	1/1126
REST	21632747	ChIP-Seq	MESCs	Mouse	2/2339
KLF1	20508144	ChIP-Seq	FETAL-LIVER-ERYTHROID	Mouse	1/1144
E2F1	21310950	ChIP-Seq	MCF-7	Human	1/1145
RCOR1	19997604	ChIP-ChIP	NEURONS	Mouse	2/2378
RELA	24523406	ChIP-Seq	FIBROSARCOMA	Human	1/1182
GATA2	19941826	ChIP-Seq	K562	Human	2/2410
MYC	18555785	ChIP-Seq	MESCs	Mouse	1/1200
EKLF	21900194	ChIP-Seq	ERYTHROCYTE	Mouse	1/1239
Nerf2	26677805	Chip-Seq	MACROPHAGESS	Mouse	1/1331
TP53	22573176	ChIP-Seq	HFKS	Human	1/1348
TCF3	18692474	ChIP-Seq	MESCs	Mouse	1/1351
BACH1	22875853	ChIP-PCR	HELA AND SCP4	Human	1/1352
GF1	26923725	Chip-Seq	HPCs	Mouse	1/1432
ESRRB	18555785	ChIP-Seq	MESCs	Mouse	1/1434
SMAD3	21741376	ChIP-Seq	HESCs	Human	1/1460
ZFP42	18358816	ChIP-ChIP	MESCs	Mouse	1/1480
POU5F1	16518401	ChIP-PET	MESCs	Mouse	1/1550
JUN	21703547	ChIP-Seq	K562	Human	1/1585
E2F4	21247883	ChIP-Seq	LYMPHOBLASTOID	Human	2/2998
SRF	21415370	ChIP-Seq	HL-1	Mouse	1/1634
GF1B	26923725	Chip-Seq	HPCs	Mouse	1/1676
CEBPD	21427703	ChIP-Seq	3T3-L1	Mouse	1/1735
LMO2	20887958	ChIP-Seq	HPC-7	Mouse	1/1741
AR	25329375	ChIP-Seq	VCAP	Human	1/1906
ESR1	22446102	ChIP-Seq	UTERUS	Mouse	1/1916
STAT4	19710469	ChIP-ChIP	TH1	Mouse	1/1939
TCF3/E2A	22897851	ChIP-Seq	JUKARTE6-1	Human	1/1946
MYC	18358816	ChIP-ChIP	MESCs	Mouse	2/3413
ERG	20887958	ChIP-Seq	HPC-7	Mouse	1/1969
SMRT	27268052	Chip-Seq	Bcells	Human	1/2000
SOX2	19829295	ChIP-Seq	ESCs	Human	1/2000
BCL6	27268052	Chip-Seq	Bcells	Human	1/2000
KLF4	19829295	ChIP-Seq	ESCs	Human	1/2000
E2A	27217539	Chip-Seq	RAMOS-Cell line	Human	1/2000
NCOR1	26117541	ChIP-Seq	K562	Human	1/2000
FOXA1	27270436	Chip-Seq	PROSTATE	Human	1/2000
GATA3	27048872	Chip-Seq	THYMUS	Human	1/2000
SA1	27219007	Chip-Seq	Bcells	Human	1/2000

EBF1	22473956	ChIP-Seq	LYMPHODE	Mouse	1/2000
GATA3	26560356	Chip-Seq	TH1	Human	1/2000
TOP2B	26459242	ChIP-Seq	MCF-7	Human	1/2000
ERA	21632823	ChIP-Seq	H3396	Human	1/2000
NCOR	22424771	ChIP-Seq	293T	Human	1/2000
NMYC	18555785	Chip-Seq	ESCs	Mouse	1/2000
RARB	24833708	ChIP-Seq	LIVER	Mouse	1/2000
KLF4	18555785	Chip-Seq	ESCs	Mouse	1/2000
CTCF	26484167	Chip-Seq	Bcells	Mouse	1/2000
POU3F1	26484290	ChIP-Seq	ESCss	Mouse	1/2000
NANOG	19829295	ChIP-Seq	ESCs	Human	1/2000
PBX	27287812	Chip-Seq	EMBYONIC-LIMB	Mouse	1/2000
FOXA1	25329375	ChIP-Seq	VCAP	Human	1/2000
GATA3	21878914	ChIP-Seq	MCF-7	Human	1/2000
LMO2	26923725	Chip-Seq	HEMOGENIC-ENDOTHELIUM	Mouse	1/2000
KDM2B	26808549	Chip-Seq	DND41	Human	1/2000
CEBPB	20513432	ChIP-Seq	MACROPHAGES	Mouse	1/2000
EBF1	22473956	ChIP-Seq	BONE MARROW	Mouse	1/2000
P300	27268052	Chip-Seq	Bcells	Human	1/2000
RUNX2	24764292	ChIP-Seq	MC3T3	Mouse	1/2000
ELK3	25401928	ChIP-Seq	HUVEC	Human	1/2000
MYC	27129775	Chip-Seq	CORNEA	Mouse	1/2000
P53	22387025	ChIP-Seq	ESCs	Mouse	1/2000
CBP	21632823	ChIP-Seq	H3396	Human	1/2000
RUNX1	27457419	Chip-Seq	LIVER	Mouse	1/2000
FOXO1	25302145	ChIP-Seq	T-LYMPHOCYTE	Mouse	1/2000
TAL1	22897851	ChIP-Seq	JUKARTE6-1	Human	1/2000
OCT1	27270436	Chip-Seq	PROSTATE	Human	1/2000
SMAD1	18555785	Chip-Seq	ESCs	Mouse	1/2000
RAC3	21632823	ChIP-Seq	H3396	Human	1/2000
GATA2	22383799	ChIP-Seq	G1ME	Mouse	1/2000
CEBPB	21427703	ChIP-Seq	3T3-L1	Mouse	1/2000
SA1	27219007	Chip-Seq	ERYTHROID	Human	1/2000
CEBPB	20176806	ChIP-Seq	MACROPHAGES	Mouse	1/2000
FOXA1	21572438	ChIP-Seq	LNCaP	Human	1/2000
ZFX	18555785	Chip-Seq	ESCs	Mouse	1/2000
PU1	27457419	Chip-Seq	LIVER	Mouse	1/2000
OCT4	19829295	ChIP-Seq	ESCs	Human	1/2000
SPI1	26923725	Chip-Seq	MACROPHAGESS	Mouse	1/2000
SA1	22415368	ChIP-Seq	MEFs	Mouse	1/2000
EZH2	27294783	Chip-Seq	ESCs	Mouse	1/2000
CRX	20693478	ChIP-Seq	RETINA	Mouse	1/2000
UTX	26944678	Chip-Seq	JUKART	Human	1/2000
FOXO3	22982991	ChIP-Seq	MACROPHAGES	Mouse	1/2000
SUZ12	18555785	Chip-Seq	ESCs	Mouse	1/2000
CEBPB	26923725	Chip-Seq	MACROPHAGESS	Mouse	1/2000
ESRRB	18555785	Chip-Seq	ESCs	Mouse	1/2000

OCT4	18555785	Chip-Seq	ESCs	Mouse	1/2000
FOXA1	26769127	Chip-Seq	PDAC-Cell line	Human	1/2000
IRF8	27001747	Chip-Seq	BMDM	Mouse	1/2000
GATA1	22383799	ChIP-Seq	G1ME	Mouse	1/2000
KDM2B	26808549	Chip-Seq	SUP-B15	Human	1/2000
ETS1	22383799	ChIP-Seq	G1ME	Mouse	1/2000
SMAD2/3	21741376	ChIP-Seq	ESCs	Human	1/2000
STAT3	18555785	Chip-Seq	ESCs	Mouse	1/2000
RING1B	27294783	Chip-Seq	NPCs	Mouse	1/2000
KLF6	26769127	Chip-Seq	PDAC-Cell line	Human	1/2000
RBPJ	21746931	ChIP-Seq	IB4-LCL	Human	1/2013
FLI1	20887958	ChIP-Seq	HPC-7	Mouse	1/2030
RAD21	21589869	ChIP-Seq	MESCs	Mouse	1/2036
AR	20517297	ChIP-Seq	VCAP	Human	1/2047
JARID1A	20064375	ChIP-Seq	MESCs	Mouse	1/2171
STAT3	20064451	ChIP-Seq	CD4+T	Mouse	1/2204
SETDB1	19884257	ChIP-Seq	MESCs	Mouse	1/2353
SMAD4	21741376	ChIP-Seq	HESCs	Human	1/2738
REST	18959480	ChIP-ChIP	MESCs	Mouse	1/2868
RUNX2	22187159	ChIP-Seq	PCA	Human	1/3423

p-value	Adjusted p-value	Old p-value	Old Adjusted p-value	*Z* -score	Combined Score
0.0002	0.1100	0.0000	0.0000	-1.2580	2.7764
0.0006	0.1561	0.0000	0.0003	-1.9631	3.6462
0.0014	0.1701	0.0000	0.0005	-1.8045	3.1963
0.0014	0.1701	0.0000	0.0005	-1.7985	3.1857
0.0019	0.1835	0.0003	0.0096	-3.2348	5.4847
0.0025	0.2023	0.0000	0.0006	-1.9778	3.1601
0.0032	0.2233	0.0000	0.0001	-0.5905	0.8854
0.0046	0.2567	0.0000	0.0006	-1.1482	1.5613
0.0047	0.2567	0.0000	0.0006	-1.5161	2.0616
0.0064	0.3019	0.0005	0.0123	-2.6537	3.1784
0.0082	0.3019	0.0001	0.0056	-2.4847	2.9760
0.0086	0.3019	0.0001	0.0035	-1.4359	1.7198
0.0086	0.3019	0.0001	0.0035	-1.3870	1.6613
0.0086	0.3019	0.0001	0.0035	-2.1449	2.5689
0.0102	0.3330	0.0004	0.0107	-2.1846	2.4022
0.0116	0.3560	0.0002	0.0068	-1.6588	1.7131
0.0155	0.4446	0.0006	0.0132	-2.8593	2.3179
0.0173	0.4521	0.0014	0.0208	-2.1723	1.7242
0.0176	0.4521	0.0002	0.0063	-1.3162	1.0447
0.0199	0.4865	0.0002	0.0068	-1.1778	0.8486
0.0226	0.5265	0.0005	0.0123	-1.4664	0.9408
0.0255	0.5584	0.0011	0.0166	-2.4055	1.4016
0.0264	0.5584	0.0006	0.0132	-1.4796	0.8621
0.0274	0.5584	0.0006	0.0132	-1.5121	0.8810
0.0324	0.5584	0.0029	0.0360	-2.1700	1.2644
0.0336	0.5584	0.0062	0.0360	-3.1744	1.8496
0.0350	0.5584	0.0001	0.0043	-0.6810	0.3968
0.0351	0.5584	0.0008	0.0141	-1.4915	0.8690
0.0351	0.5584	0.0008	0.0141	-1.3317	0.7759
0.0351	0.5584	0.0008	0.0141	-1.2774	0.7443
0.0388	0.5584	0.0018	0.0239	-1.6492	0.9609
0.0429	0.5584	0.0010	0.0166	-1.5688	0.9141
0.0493	0.5584	0.0046	0.0360	-1.9725	1.1493
0.0543	0.5584	0.0014	0.0201	-1.9800	1.1537
0.0546	0.5584	0.0051	0.0360	-2.5753	1.5005
0.0600	0.5584	0.0008	0.0141	-0.8312	0.4843
0.0646	0.5584	0.0062	0.0360	-1.8927	1.1028
0.0675	0.5584	0.0034	0.0360	-1.7572	1.0239
0.0687	0.5584	0.0133	0.0597	-2.2242	1.2959
0.0709	0.5584	0.0036	0.0360	-1.8076	1.0532
0.0712	0.5584	0.0069	0.0378	-1.7217	1.0032
0.0723	0.5584	0.0037	0.0360	-1.7202	1.0023
0.0725	0.5584	0.0071	0.0380	-1.8997	1.1069
0.0743	0.5584	0.0011	0.0166	-1.1258	0.6559
0.0767	0.5584	0.0075	0.0393	-2.3157	1.3493
0.0769	0.5584	0.0076	0.0393	-1.7695	1.0310

0.0811	0.5584	0.0080	0.0414	-1.9166	1.1168
0.0822	0.5584	0.0345	0.0989	-3.2853	1.9142
0.0866	0.5584	0.0087	0.0439	-1.9580	1.1409
0.0897	0.5584	0.0090	0.0451	-1.7356	1.0113
0.0899	0.5584	0.0048	0.0360	-1.3975	0.8143
0.0935	0.5584	0.0394	0.0989	-3.4883	2.0325
0.0935	0.5584	0.0394	0.0989	-3.4769	2.0259
0.0944	0.5584	0.0397	0.0989	-3.4927	2.0351
0.0950	0.5584	0.0027	0.0360	-1.0606	0.6180
0.0999	0.5584	0.0200	0.0804	-3.3770	1.9677
0.1008	0.5584	0.0055	0.0360	-1.4996	0.8738
0.1012	0.5584	0.0104	0.0515	-1.5538	0.9054
0.1017	0.5584	0.0016	0.0225	-1.0346	0.6029
0.1032	0.5584	0.0207	0.0818	-2.6598	1.5498
0.1046	0.5584	0.0058	0.0360	-1.3914	0.8107
0.1061	0.5584	0.0059	0.0360	-1.2087	0.7043
0.1081	0.5584	0.0218	0.0847	-2.0957	1.2211
0.1117	0.5584	0.0117	0.0568	-1.4913	0.8689
0.1136	0.5584	0.0064	0.0360	-1.1465	0.6680
0.1149	0.5584	0.0065	0.0360	-1.1779	0.6863
0.1149	0.5584	0.0065	0.0360	-1.1529	0.6717
0.1149	0.5584	0.0065	0.0360	-1.1335	0.6605
0.1149	0.5584	0.0065	0.0360	-1.1126	0.6483
0.1149	0.5584	0.0065	0.0360	-1.1104	0.6470
0.1149	0.5584	0.0065	0.0360	-1.1081	0.6457
0.1149	0.5584	0.0065	0.0360	-1.1018	0.6420
0.1149	0.5584	0.0065	0.0360	-1.0883	0.6341
0.1149	0.5584	0.0065	0.0360	-1.0855	0.6325
0.1149	0.5584	0.0065	0.0360	-1.0827	0.6308
0.1149	0.5584	0.0065	0.0360	-1.0727	0.6250
0.1149	0.5584	0.0065	0.0360	-1.0562	0.6154
0.1149	0.5584	0.0065	0.0360	-1.0547	0.6145
0.1149	0.5584	0.0065	0.0360	-1.0502	0.6119
0.1149	0.5584	0.0065	0.0360	-1.0397	0.6058
0.1149	0.5584	0.0065	0.0360	-1.0333	0.6020
0.1149	0.5584	0.0065	0.0360	-1.0332	0.6020
0.1149	0.5584	0.0065	0.0360	-1.0322	0.6014
0.1149	0.5584	0.0065	0.0360	-1.0291	0.5996
0.1149	0.5584	0.0065	0.0360	-1.0287	0.5994
0.1149	0.5584	0.0065	0.0360	-1.0258	0.5977
0.1149	0.5584	0.0065	0.0360	-1.0198	0.5942
0.1149	0.5584	0.0065	0.0360	-1.0149	0.5913
0.1149	0.5584	0.0065	0.0360	-0.9982	0.5816
0.1149	0.5584	0.0065	0.0360	-0.9955	0.5800
0.1149	0.5584	0.0065	0.0360	-0.9848	0.5738
0.1149	0.5584	0.0065	0.0360	-0.9603	0.5595
0.1149	0.5584	0.0065	0.0360	-0.9567	0.5574

0.1149	0.5584	0.0065	0.0360	-0.9531	0.5554
0.1149	0.5584	0.0065	0.0360	-0.9504	0.5538
0.1149	0.5584	0.0065	0.0360	-0.9396	0.5475
0.1149	0.5584	0.0065	0.0360	-0.9372	0.5461
0.1149	0.5584	0.0065	0.0360	-0.9282	0.5408
0.1149	0.5584	0.0065	0.0360	-0.9134	0.5322
0.1149	0.5584	0.0065	0.0360	-0.9076	0.5288
0.1160	0.5584	0.0122	0.0587	-1.9232	1.1206
0.1166	0.5584	0.0493	0.1113	-2.4219	1.4111
0.1176	0.5584	0.0125	0.0591	-2.3866	1.3906
0.1200	0.5604	0.0508	0.1124	-2.8511	1.6513
0.1214	0.5604	0.0069	0.0378	-1.0408	0.6028
0.1226	0.5604	0.0131	0.0597	-1.4306	0.8286
0.1226	0.5604	0.0131	0.0597	-1.4242	0.8248
0.1261	0.5683	0.0259	0.0983	-1.7716	1.0013
0.1267	0.5683	0.0073	0.0390	-0.8157	0.4610
0.1290	0.5733	0.0266	0.0989	-1.7074	0.9500
0.1313	0.5783	0.0006	0.0132	0.4673	-0.2560
0.1403	0.6125	0.0154	0.0667	-0.9599	0.4706
0.1570	0.6626	0.0177	0.0752	-1.6402	0.6751
0.1571	0.6626	0.0672	0.1417	-2.4882	1.0241
0.1591	0.6626	0.0180	0.0758	-1.0630	0.4375
0.1609	0.6626	0.0009	0.0148	0.5368	-0.2209
0.1643	0.6626	0.0705	0.1466	-2.3843	0.9813
0.1645	0.6626	0.0351	0.0989	-1.8474	0.7604
0.1672	0.6626	0.0191	0.0793	-1.2345	0.5081
0.1699	0.6626	0.0364	0.0989	-1.8564	0.7641
0.1707	0.6626	0.0733	0.1508	-2.8671	1.1801
0.1730	0.6626	0.0372	0.0989	-1.4931	0.6145
0.1736	0.6626	0.0201	0.0804	-0.9796	0.4032
0.1749	0.6626	0.0062	0.0360	-0.1807	0.0744
0.1779	0.6626	0.0207	0.0818	-1.2401	0.5104
0.1843	0.6626	0.0400	0.0989	-1.4521	0.5977
0.1849	0.6626	0.0217	0.0847	-0.8934	0.3677
0.1857	0.6626	0.0801	0.1626	-2.3763	0.9781
0.1867	0.6626	0.0407	0.0999	-1.7224	0.7089
0.1881	0.6626	0.0410	0.1003	-2.1095	0.8682
0.1898	0.6626	0.0415	0.1008	-1.6433	0.6764
0.1937	0.6626	0.0424	0.1026	-1.4130	0.5816
0.1953	0.6626	0.0129	0.0597	-0.8923	0.3672
0.1958	0.6626	0.0847	0.1691	-2.1978	0.9046
0.1968	0.6626	0.0432	0.1026	-1.6798	0.6914
0.1968	0.6626	0.0432	0.1026	-1.2156	0.5003
0.1971	0.6626	0.0433	0.1026	-1.7216	0.7086
0.1993	0.6626	0.0133	0.0597	-0.3921	0.1614
0.2104	0.6626	0.0915	0.1811	-2.1040	0.8660
0.2112	0.6626	0.0144	0.0634	-0.5367	0.2209

0.2140	0.6626	0.0477	0.1092	-2.2634	0.9316
0.2154	0.6626	0.0481	0.1094	-1.7190	0.7075
0.2162	0.6626	0.0265	0.0989	-0.7666	0.3155
0.2199	0.6626	0.0152	0.0665	-0.9720	0.4001
0.2211	0.6626	0.0496	0.1113	-1.2166	0.5007
0.2211	0.6626	0.0496	0.1113	-1.1679	0.4807
0.2222	0.6626	0.0087	0.0439	0.0465	-0.0191
0.2223	0.6626	0.0275	0.0989	-0.9476	0.3900
0.2235	0.6626	0.0503	0.1123	-1.7811	0.7331
0.2269	0.6626	0.0282	0.0989	-1.0668	0.4391
0.2276	0.6626	0.0160	0.0685	-0.4530	0.1864
0.2335	0.6626	0.0530	0.1162	-1.9162	0.7887
0.2383	0.6626	0.0301	0.0989	-0.6374	0.2623
0.2395	0.6626	0.1051	0.2040	-2.5744	1.0596
0.2433	0.6626	0.0309	0.0989	-0.6456	0.2657
0.2453	0.6626	0.0563	0.1214	-1.1792	0.4853
0.2453	0.6626	0.0563	0.1214	-1.1737	0.4831
0.2456	0.6626	0.0564	0.1214	-2.4013	0.9883
0.2472	0.6626	0.0316	0.0989	-0.6515	0.2681
0.2477	0.6626	0.0317	0.0989	-1.2894	0.5307
0.2503	0.6626	0.0577	0.1237	-2.3160	0.9532
0.2661	0.6626	0.0621	0.1321	-1.1914	0.4904
0.2688	0.6626	0.0353	0.0989	-0.7388	0.3041
0.2714	0.6626	0.0117	0.0568	0.2960	-0.1218
0.2739	0.6626	0.1216	0.2314	-1.9022	0.7829
0.2768	0.6626	0.0367	0.0989	-0.5562	0.2289
0.2794	0.6626	0.0660	0.1397	-0.9626	0.3962
0.2803	0.6626	0.0374	0.0989	-0.8180	0.3367
0.2809	0.6626	0.1250	0.2361	-2.7827	1.1453
0.2873	0.6626	0.0683	0.1434	-1.0103	0.4158
0.2887	0.6626	0.0129	0.0597	0.2626	-0.1081
0.2891	0.6626	0.0689	0.1439	-1.1389	0.4688
0.2910	0.6626	0.0393	0.0989	-0.7064	0.2908
0.2921	0.6626	0.0395	0.0989	-0.6213	0.2557
0.2929	0.6626	0.0397	0.0989	-0.2861	0.1178
0.2945	0.6626	0.0400	0.0989	-0.7283	0.2998
0.2945	0.6626	0.0400	0.0989	-0.4821	0.1984
0.2945	0.6626	0.0400	0.0989	-0.4793	0.1973
0.2945	0.6626	0.0400	0.0989	-0.4162	0.1713
0.2945	0.6626	0.0400	0.0989	-0.4123	0.1697
0.2945	0.6626	0.0400	0.0989	-0.4099	0.1687
0.2945	0.6626	0.0400	0.0989	-0.4031	0.1659
0.2945	0.6626	0.0400	0.0989	-0.3913	0.1610
0.2945	0.6626	0.0400	0.0989	-0.3771	0.1552
0.2945	0.6626	0.0400	0.0989	-0.3693	0.1520
0.2945	0.6626	0.0400	0.0989	-0.3627	0.1493
0.2945	0.6626	0.0400	0.0989	-0.3621	0.1490

0.2945	0.6626	0.0400	0.0989	-0.3618	0.1489
0.2945	0.6626	0.0400	0.0989	-0.3579	0.1473
0.2945	0.6626	0.0400	0.0989	-0.3553	0.1463
0.2945	0.6626	0.0400	0.0989	-0.3510	0.1445
0.2945	0.6626	0.0400	0.0989	-0.3383	0.1392
0.2945	0.6626	0.0400	0.0989	-0.3340	0.1375
0.2945	0.6626	0.0400	0.0989	-0.3268	0.1345
0.2945	0.6626	0.0400	0.0989	-0.3193	0.1314
0.2945	0.6626	0.0400	0.0989	-0.3160	0.1301
0.2945	0.6626	0.0400	0.0989	-0.3075	0.1266
0.2945	0.6626	0.0400	0.0989	-0.3063	0.1261
0.2945	0.6626	0.0400	0.0989	-0.3049	0.1255
0.2945	0.6626	0.0400	0.0989	-0.3001	0.1235
0.2945	0.6626	0.0400	0.0989	-0.2987	0.1229
0.2945	0.6626	0.0400	0.0989	-0.2901	0.1194
0.2945	0.6626	0.0400	0.0989	-0.2894	0.1191
0.2945	0.6626	0.0400	0.0989	-0.2326	0.0957
0.2945	0.6626	0.0400	0.0989	-0.2262	0.0931
0.2945	0.6626	0.0400	0.0989	-0.2228	0.0917
0.2945	0.6626	0.0400	0.0989	-0.2201	0.0906
0.2945	0.6626	0.0400	0.0989	-0.2193	0.0903
0.2945	0.6626	0.0400	0.0989	-0.2003	0.0824
0.2945	0.6626	0.0400	0.0989	-0.1984	0.0817
0.2945	0.6626	0.0400	0.0989	-0.1979	0.0815
0.2945	0.6626	0.0400	0.0989	-0.1927	0.0793
0.2945	0.6626	0.0400	0.0989	-0.1613	0.0664
0.2945	0.6626	0.0400	0.0989	-0.1594	0.0656
0.2945	0.6626	0.0400	0.0989	-0.1505	0.0619
0.2945	0.6626	0.0400	0.0989	-0.1318	0.0542
0.2953	0.6626	0.0043	0.0360	2.3656	-0.9737
0.2954	0.6626	0.1321	0.2476	-1.6850	0.6935
0.2999	0.6696	0.0137	0.0609	0.2082	-0.0835
0.3013	0.6697	0.0725	0.1503	-1.7964	0.7201
0.3042	0.6731	0.0734	0.1508	-1.0283	0.4071
0.3115	0.6856	0.0251	0.0958	0.1847	-0.0697
0.3126	0.6856	0.0434	0.1026	-0.0502	0.0189
0.3182	0.6910	0.0777	0.1590	-0.5239	0.1936
0.3195	0.6910	0.1442	0.2641	-1.2936	0.4782
0.3197	0.6910	0.0448	0.1043	-1.1254	0.4160
0.3210	0.6910	0.0786	0.1602	-0.6046	0.2235
0.3222	0.6910	0.1455	0.2643	-1.4349	0.5304
0.3265	0.6925	0.0461	0.1066	-0.6866	0.2523
0.3268	0.6925	0.0462	0.1066	0.0099	-0.0037
0.3277	0.6925	0.0271	0.0989	-1.7261	0.6342
0.3286	0.6925	0.0810	0.1629	-0.8211	0.3017
0.3346	0.7022	0.1518	0.2643	-1.2290	0.4346
0.3378	0.7060	0.0839	0.1682	-0.5264	0.1832

0.3474	0.7222	0.1584	0.2643	-1.2443	0.4050
0.3485	0.7222	0.0874	0.1737	-0.4991	0.1625
0.3505	0.7233	0.1600	0.2643	-2.2751	0.7371
0.3546	0.7285	0.0519	0.1143	-0.3372	0.1068
0.3568	0.7301	0.1633	0.2643	-1.1801	0.3713
0.3599	0.7318	0.1649	0.2643	-0.9974	0.3114
0.3615	0.7318	0.0315	0.0989	0.6530	-0.2039
0.3622	0.7318	0.0185	0.0775	0.5017	-0.1566
0.3637	0.7318	0.1669	0.2643	-1.3723	0.4285
0.3655	0.7325	0.1679	0.2643	-1.0830	0.3371
0.3718	0.7389	0.0064	0.0360	1.4261	-0.4316
0.3741	0.7389	0.0196	0.0800	0.9661	-0.2924
0.3742	0.7389	0.0561	0.1214	-0.0088	0.0026
0.3747	0.7389	0.0196	0.0800	0.4207	-0.1273
0.3897	0.7630	0.0354	0.0989	0.0765	-0.0207
0.3901	0.7630	0.1014	0.1991	-0.6356	0.1719
0.3925	0.7647	0.1022	0.1999	-0.3056	0.0820
0.3949	0.7662	0.0607	0.1297	0.2996	-0.0798
0.3981	0.7694	0.1852	0.2695	-0.9210	0.2415
0.4007	0.7714	0.0220	0.0847	1.5214	-0.3948
0.4068	0.7782	0.1899	0.2753	-1.0628	0.2666
0.4074	0.7782	0.1903	0.2753	-0.9226	0.2314
0.4108	0.7805	0.1087	0.2101	-0.1834	0.0454
0.4132	0.7805	0.0389	0.0989	0.2314	-0.0573
0.4143	0.7805	0.1940	0.2799	-1.0228	0.2535
0.4166	0.7805	0.1953	0.2801	-0.9196	0.2279
0.4166	0.7805	0.1953	0.2801	-0.9123	0.2261
0.4195	0.7829	0.0399	0.0989	0.8122	-0.1987
0.4245	0.7874	0.1997	0.2851	-0.8634	0.2064
0.4251	0.7874	0.2000	0.2851	-0.8426	0.2014
0.4299	0.7934	0.1157	0.2219	-0.2165	0.0501
0.4364	0.7942	0.0426	0.1026	-1.8783	0.4327
0.4390	0.7942	0.2078	0.2953	-0.7204	0.1659
0.4454	0.7942	0.1215	0.2314	-0.4096	0.0944
0.4468	0.7942	0.0444	0.1041	0.9814	-0.2261
0.4475	0.7942	0.0445	0.1041	-51.3675	11.8330
0.4515	0.7942	0.2149	0.3037	-1.2976	0.2989
0.4521	0.7942	0.1241	0.2352	0.0187	-0.0043
0.4584	0.7942	0.1265	0.2380	-0.0640	0.0147
0.4595	0.7942	0.2194	0.3092	-1.6271	0.3748
0.4669	0.7942	0.0478	0.1092	1.0188	-0.2347
0.4690	0.7942	0.2249	0.3136	-0.7710	0.1776
0.4695	0.7942	0.2252	0.3136	-0.7176	0.1653
0.4739	0.7942	0.1326	0.2476	0.0048	-0.0011
0.4745	0.7942	0.0806	0.1629	0.0597	-0.0137
0.4757	0.7942	0.2288	0.3156	-0.6211	0.1431
0.4762	0.7942	0.2292	0.3156	-0.9681	0.2230

0.4810	0.7942	0.1355	0.2510	-0.6413	0.1477
0.4814	0.7942	0.2322	0.3189	-0.6555	0.1510
0.4833	0.7942	0.0508	0.1124	1.4341	-0.3304
0.4842	0.7942	0.1368	0.2525	-1.0203	0.2350
0.4869	0.7942	0.2355	0.3216	-0.6236	0.1437
0.4881	0.7942	0.1384	0.2545	-0.0755	0.0174
0.4895	0.7942	0.2370	0.3228	-0.4949	0.1140
0.5107	0.7942	0.2497	0.3382	-0.6638	0.1529
0.5178	0.7942	0.2541	0.3432	-0.7510	0.1730
0.5207	0.7942	0.2558	0.3446	-0.4579	0.1055
0.5219	0.7942	0.0942	0.1857	0.7166	-0.1651
0.5268	0.7942	0.2596	0.3488	-1.0273	0.2367
0.5323	0.7942	0.1573	0.2643	0.2698	-0.0621
0.5420	0.7942	0.1617	0.2643	0.2731	-0.0629
0.5434	0.7942	0.2700	0.3617	-0.4087	0.0941
0.5489	0.7942	0.0394	0.0989	2.1339	-0.4916
0.5510	0.7942	0.1033	0.2012	0.9047	-0.2084
0.5516	0.7942	0.1660	0.2643	0.4472	-0.1030
0.5563	0.7942	0.2783	0.3718	-0.3734	0.0860
0.5576	0.7942	0.2791	0.3719	-0.5138	0.1184
0.5659	0.7942	0.2845	0.3780	-0.3847	0.0886
0.5689	0.7942	0.1742	0.2643	0.5424	-0.1249
0.5713	0.7942	0.1100	0.2118	0.4306	-0.0992
0.5767	0.7942	0.1779	0.2643	0.2135	-0.0492
0.5790	0.7942	0.1791	0.2643	0.3836	-0.0884
0.5798	0.7942	0.1795	0.2643	0.3341	-0.0770
0.5798	0.7942	0.1795	0.2643	0.3455	-0.0796
0.5798	0.7942	0.1795	0.2643	0.3911	-0.0901
0.5798	0.7942	0.1795	0.2643	0.4082	-0.0940
0.5798	0.7942	0.1795	0.2643	0.4233	-0.0975
0.5798	0.7942	0.1795	0.2643	0.4364	-0.1005
0.5798	0.7942	0.1795	0.2643	0.4385	-0.1010
0.5798	0.7942	0.1795	0.2643	0.4393	-0.1012
0.5798	0.7942	0.1795	0.2643	0.4443	-0.1023
0.5798	0.7942	0.1795	0.2643	0.4496	-0.1036
0.5798	0.7942	0.1795	0.2643	0.4561	-0.1051
0.5798	0.7942	0.1795	0.2643	0.4563	-0.1051
0.5798	0.7942	0.1795	0.2643	0.4668	-0.1075
0.5798	0.7942	0.1795	0.2643	0.4727	-0.1089
0.5798	0.7942	0.1795	0.2643	0.4805	-0.1107
0.5798	0.7942	0.1795	0.2643	0.4866	-0.1121
0.5798	0.7942	0.1795	0.2643	0.4874	-0.1123
0.5798	0.7942	0.1795	0.2643	0.4917	-0.1133
0.5798	0.7942	0.1795	0.2643	0.4988	-0.1149
0.5798	0.7942	0.1795	0.2643	0.4996	-0.1151
0.5798	0.7942	0.1795	0.2643	0.5078	-0.1170
0.5798	0.7942	0.1795	0.2643	0.5087	-0.1172

0.5798	0.7942	0.1795	0.2643	0.5109	-0.1177
0.5798	0.7942	0.1795	0.2643	0.5201	-0.1198
0.5798	0.7942	0.1795	0.2643	0.5293	-0.1219
0.5798	0.7942	0.1795	0.2643	0.5335	-0.1229
0.5798	0.7942	0.1795	0.2643	0.5339	-0.1230
0.5798	0.7942	0.1795	0.2643	0.5388	-0.1241
0.5798	0.7942	0.1795	0.2643	0.5406	-0.1245
0.5798	0.7942	0.1795	0.2643	0.5534	-0.1275
0.5798	0.7942	0.1795	0.2643	0.5537	-0.1276
0.5798	0.7942	0.1795	0.2643	0.5758	-0.1326
0.5798	0.7942	0.1795	0.2643	0.5812	-0.1339
0.5798	0.7942	0.1795	0.2643	0.6114	-0.1409
0.5798	0.7942	0.1795	0.2643	0.6166	-0.1420
0.5798	0.7942	0.1795	0.2643	0.6207	-0.1430
0.5798	0.7942	0.1795	0.2643	0.6212	-0.1431
0.5798	0.7942	0.1795	0.2643	0.6234	-0.1436
0.5798	0.7942	0.1795	0.2643	0.6294	-0.1450
0.5798	0.7942	0.1795	0.2643	0.6355	-0.1464
0.5798	0.7942	0.1795	0.2643	0.6397	-0.1474
0.5798	0.7942	0.1795	0.2643	0.6468	-0.1490
0.5798	0.7942	0.1795	0.2643	0.6503	-0.1498
0.5798	0.7942	0.1795	0.2643	0.6616	-0.1524
0.5798	0.7942	0.1795	0.2643	0.6642	-0.1530
0.5798	0.7942	0.1795	0.2643	0.6833	-0.1574
0.5798	0.7942	0.1795	0.2643	0.6911	-0.1592
0.5798	0.7942	0.1795	0.2643	0.6942	-0.1599
0.5798	0.7942	0.1795	0.2643	0.7038	-0.1621
0.5798	0.7942	0.1795	0.2643	0.7204	-0.1659
0.5798	0.7942	0.1795	0.2643	0.7876	-0.1814
0.5828	0.7952	0.2956	0.3918	-0.0824	0.0189
0.5838	0.7952	0.1814	0.2664	0.6527	-0.1495
0.5855	0.7953	0.1823	0.2669	0.2324	-0.0532
0.5906	0.7982	0.3009	0.3976	-0.0935	0.0211
0.5909	0.7982	0.1849	0.2695	0.0387	-0.0087
0.5927	0.7984	0.3022	0.3984	0.1657	-0.0373
0.6011	0.8075	0.3080	0.4048	0.0333	-0.0071
0.6133	0.8216	0.3164	0.4147	0.0487	-0.0096
0.6228	0.8246	0.3231	0.4179	0.0629	-0.0121
0.6228	0.8246	0.3231	0.4179	0.0743	-0.0143
0.6228	0.8246	0.3231	0.4179	0.1691	-0.0326
0.6232	0.8246	0.3233	0.4179	-0.6866	0.1324
0.6239	0.8246	0.3239	0.4179	-0.3301	0.0637
0.6273	0.8268	0.3262	0.4198	0.1813	-0.0345
0.6303	0.8278	0.3284	0.4214	0.1996	-0.0377
0.6314	0.8278	0.3292	0.4214	0.0162	-0.0031
0.6336	0.8284	0.3307	0.4223	0.1767	-0.0333
0.6363	0.8298	0.1341	0.2494	1.7942	-0.3347

0.6420	0.8323	0.3368	0.4289	0.2211	-0.0406
0.6423	0.8323	0.2121	0.3007	0.9456	-0.1735
0.6434	0.8323	0.3378	0.4291	0.2004	-0.0368
0.6455	0.8329	0.3394	0.4300	0.2529	-0.0462
0.6547	0.8425	0.3461	0.4374	0.3039	-0.0521
0.6586	0.8441	0.2215	0.3109	0.3536	-0.0599
0.6594	0.8441	0.2219	0.3109	0.2664	-0.0452
0.6660	0.8499	0.2258	0.3136	1.0443	-0.1698
0.6676	0.8499	0.3559	0.4485	-0.2304	0.0375
0.6691	0.8499	0.2276	0.3154	1.0985	-0.1787
0.6736	0.8516	0.3604	0.4522	0.3810	-0.0612
0.6739	0.8516	0.3607	0.4522	0.0659	-0.0106
0.6785	0.8551	0.2333	0.3195	0.4387	-0.0687
0.6859	0.8601	0.3700	0.4627	0.5787	-0.0872
0.6860	0.8601	0.2379	0.3232	0.1769	-0.0267
0.6915	0.8649	0.3744	0.4671	0.1735	-0.0252
0.7035	0.8776	0.3840	0.4766	0.5670	-0.0741
0.7299	0.8824	0.4061	0.5027	0.5304	-0.0664
0.7346	0.8824	0.4100	0.5049	0.4844	-0.0606
0.7354	0.8824	0.4107	0.5049	0.2237	-0.0280
0.7357	0.8824	0.4110	0.5049	0.3075	-0.0385
0.7564	0.8824	0.4294	0.5255	0.6025	-0.0754
0.7569	0.8824	0.4298	0.5255	0.4195	-0.0525
0.7633	0.8824	0.4357	0.5313	-0.0295	0.0037
0.7681	0.8824	0.4402	0.5354	0.3611	-0.0452
0.7842	0.8824	0.4556	0.5528	-0.5034	0.0630
0.7919	0.8824	0.4631	0.5567	0.3506	-0.0439
0.8013	0.8824	0.3236	0.4179	1.4945	-0.1870
0.8021	0.8824	0.4735	0.5567	0.1010	-0.0126
0.8106	0.8824	0.4823	0.5567	0.8670	-0.1085
0.8218	0.8824	0.4943	0.5567	0.8858	-0.1108
0.8229	0.8824	0.4955	0.5567	0.9133	-0.1142
0.8510	0.8824	0.5278	0.5567	0.8040	-0.1006
0.8526	0.8824	0.5297	0.5567	0.0478	-0.0060
0.8561	0.8824	0.5340	0.5567	-0.1855	0.0232
0.8571	0.8824	0.5353	0.5567	0.8852	-0.1107
0.8598	0.8824	0.3833	0.4766	1.1474	-0.1435
0.8606	0.8824	0.5396	0.5567	1.2023	-0.1504
0.8650	0.8824	0.5453	0.5567	0.9480	-0.1186
0.8650	0.8824	0.5453	0.5567	0.9705	-0.1214
0.8650	0.8824	0.5453	0.5567	0.9860	-0.1233
0.8650	0.8824	0.5453	0.5567	1.0163	-0.1271
0.8650	0.8824	0.5453	0.5567	1.0215	-0.1278
0.8650	0.8824	0.5453	0.5567	1.0477	-0.1311
0.8650	0.8824	0.5453	0.5567	1.0662	-0.1334
0.8650	0.8824	0.5453	0.5567	1.0717	-0.1341
0.8650	0.8824	0.5453	0.5567	1.1046	-0.1382

0.8650	0.8824	0.5453	0.5567	1.1056	-0.1383
0.8650	0.8824	0.5453	0.5567	1.1091	-0.1387
0.8650	0.8824	0.5453	0.5567	1.1258	-0.1408
0.8650	0.8824	0.5453	0.5567	1.1410	-0.1427
0.8650	0.8824	0.5453	0.5567	1.1413	-0.1428
0.8650	0.8824	0.5453	0.5567	1.1448	-0.1432
0.8650	0.8824	0.5453	0.5567	1.1465	-0.1434
0.8650	0.8824	0.5453	0.5567	1.1592	-0.1450
0.8650	0.8824	0.5453	0.5567	1.1631	-0.1455
0.8650	0.8824	0.5453	0.5567	1.1636	-0.1456
0.8650	0.8824	0.5453	0.5567	1.1673	-0.1460
0.8650	0.8824	0.5453	0.5567	1.1723	-0.1467
0.8650	0.8824	0.5453	0.5567	1.1921	-0.1491
0.8650	0.8824	0.5453	0.5567	1.2007	-0.1502
0.8650	0.8824	0.5453	0.5567	1.2123	-0.1516
0.8650	0.8824	0.5453	0.5567	1.2127	-0.1517
0.8650	0.8824	0.5453	0.5567	1.2143	-0.1519
0.8650	0.8824	0.5453	0.5567	1.2217	-0.1528
0.8650	0.8824	0.5453	0.5567	1.2293	-0.1538
0.8650	0.8824	0.5453	0.5567	1.2443	-0.1557
0.8650	0.8824	0.5453	0.5567	1.2518	-0.1566
0.8650	0.8824	0.5453	0.5567	1.2554	-0.1570
0.8650	0.8824	0.5453	0.5567	1.2568	-0.1572
0.8650	0.8824	0.5453	0.5567	1.2639	-0.1581
0.8650	0.8824	0.5453	0.5567	1.2802	-0.1601
0.8650	0.8824	0.5453	0.5567	1.2864	-0.1609
0.8650	0.8824	0.5453	0.5567	1.2967	-0.1622
0.8650	0.8824	0.5453	0.5567	1.2981	-0.1624
0.8650	0.8824	0.5453	0.5567	1.3065	-0.1634
0.8650	0.8824	0.5453	0.5567	1.3197	-0.1651
0.8650	0.8824	0.5453	0.5567	1.3224	-0.1654
0.8650	0.8824	0.5453	0.5567	1.3232	-0.1655
0.8650	0.8824	0.5453	0.5567	1.3261	-0.1659
0.8650	0.8824	0.5453	0.5567	1.3279	-0.1661
0.8650	0.8824	0.5453	0.5567	1.3314	-0.1665
0.8650	0.8824	0.5453	0.5567	1.3322	-0.1667
0.8650	0.8824	0.5453	0.5567	1.3363	-0.1672
0.8650	0.8824	0.5453	0.5567	1.3501	-0.1689
0.8650	0.8824	0.5453	0.5567	1.3565	-0.1697
0.8650	0.8824	0.5453	0.5567	1.3692	-0.1713
0.8650	0.8824	0.5453	0.5567	1.3787	-0.1725
0.8650	0.8824	0.5453	0.5567	1.3842	-0.1732
0.8650	0.8824	0.5453	0.5567	1.3901	-0.1739
0.8650	0.8824	0.5453	0.5567	1.4054	-0.1758
0.8650	0.8824	0.5453	0.5567	1.4246	-0.1782
0.8650	0.8824	0.5453	0.5567	1.4276	-0.1786
0.8650	0.8824	0.5453	0.5567	1.4282	-0.1787

0.8650	0.8824	0.5453	0.5567	1.4355	-0.1796
0.8650	0.8824	0.5453	0.5567	1.4421	-0.1804
0.8650	0.8824	0.5453	0.5567	1.4440	-0.1806
0.8650	0.8824	0.5453	0.5567	1.4468	-0.1810
0.8650	0.8824	0.5453	0.5567	1.4518	-0.1816
0.8650	0.8824	0.5453	0.5567	1.4585	-0.1825
0.8650	0.8824	0.5453	0.5567	1.4758	-0.1846
0.8650	0.8824	0.5453	0.5567	1.4823	-0.1854
0.8650	0.8824	0.5453	0.5567	1.4832	-0.1855
0.8650	0.8824	0.5453	0.5567	1.4932	-0.1868
0.8669	0.8824	0.5477	0.5579	1.3318	-0.1666
0.8693	0.8824	0.5508	0.5598	1.3881	-0.1736
0.8701	0.8824	0.5518	0.5598	1.4930	-0.1868
0.8716	0.8824	0.5538	0.5607	1.4028	-0.1755
0.8874	0.8966	0.5756	0.5815	1.4639	-0.1598
0.8913	0.8987	0.5812	0.5860	-0.8484	0.0906
0.9074	0.9130	0.6057	0.6094	1.5774	-0.1436
0.9391	0.9430	0.6629	0.6656	0.9975	-0.0586
0.9473	0.9492	0.6803	0.6817	0.6642	-0.0346
0.9718	0.9718	0.7457	0.7457	2.4376	-0.0697

Genes involved in neuronal migration
FOXD3; GSK3B; APP; PCM1; DAB1; CDK5; RHEB; DISC1; BBS4; ZNF365
FOXD3; GSK3B; DAB1; CDK5; RHEB; DIXDC1; ACTB
APP; CCDC88A; CDK5; RHEB; ACTB; PAFAH1B1; ZNF365
APP; CCDC88A; CDK5; RHEB; ACTB; PAFAH1B1; ZNF365
PCM1; AKT1
GSK3B; APP; DAB1; CDK5; RHEB; ACTB; NDEL1
GSK3B; PCM1; DAB1; CDK5; RHEB; DIXDC1; AKT1; DISC1; ACTB; PAFAH1B1; ZNF365; NDEL1
GSK3B; CCDC88A; CDK5; RHEB; SOX10; ACTB; PAFAH1B1; NDEL1
FOXD3; GSK3B; APP; DAB1; CDK5; RHEB; DISC1; NDEL1
AKT1; ACTB; NDEL1
GSK3B; PCM1; RHEB; AKT1; DISC1
FOXD3; APP; CCDC88A; DAB1; CCDC141; SOX10
PCM1; CDK5; DIXDC1; AKT1; CCDC141; NDEL1
FOXD3; GSK3B; PCM1; DAB1; ACTB; PAFAH1B1
PCM1; DIXDC1; ACTB; BBS4
CDK5; DIXDC1; DISC1; ACTB; NDEL1
APP; CCDC88A; RHEB; DISC1
GSK3B; CDK5; RHEB
GSK3B; APP; DAB1; AKT1; ACTB; PAFAH1B1
GSK3B; CDK5; RHEB; ACTB; PAFAH1B1; NDEL1
GSK3B; DISC1; ACTB; ZNF365; NDEL1
APP; DAB1; RHEB; DISC1
CCDC88A; CCDC141; DISC1; BBS4; ZNF365
RHEB; AKT1; DISC1; ACTB; NDEL1
APP; DAB1; BBS4
PCM1; CDK5
CCDC88A; PCM1; DAB1; CDK5; CCDC141; BBS4; ACTB; NDEL1
CDK5; AKT1; SOX10; ACTB; BBS4
CCDC88A; PCM1; RHEB; DISC1; PAFAH1B1
PCM1; DIXDC1; AKT1; CCDC141; ACTB
DAB1; RHEB; BBS4; PAFAH1B1
FOXD3; DAB1; RHEB; AKT1; NDEL1
APP; DAB1; SOX10
FOXD3; RHEB; DIXDC1; ACTB; NDEL1
GSK3B; RHEB; DISC1
CCDC88A; GSK3B; CCDC141; DISC1; ACTB; NDEL1
DIXDC1; ACTB; ZNF365
APP; PCM1; DAB1; RHEB
DAB1; DISC1
FOXD3; DIXDC1; DISC1; ACTB
PCM1; CDK5; RHEB
GSK3B; PCM1; DIXDC1; ZNF365
GSK3B; CDK5; RHEB
RHEB; DIXDC1; AKT1; ACTB; PAFAH1B1; NDEL1
FOXD3; PCM1; RHEB
APP; DAB1; ZNF365

APP; PCM1; SOX10
PAFAH1B1
AKT1; ACTB; NDEL1
FOXD3; APP; DAB1
AKT1; DISC1; BBS4; NDEL1
FOXD3
FOXD3
FOXD3
GSK3B; CDK5; ACTB; BBS4; NDEL1
FOXD3; DAB1
FOXD3; APP; DAB1; DISC1
CCDC141; DISC1; BBS4
GSK3B; APP; DAB1; DISC1; SOX10; ZNF365
FOXD3; GSK3B
FOXD3; APP; DAB1; DISC1
FOXD3; CCDC141; ACTB; PAFAH1B1
RHEB; DISC1
RHEB; CCDC141; ACTB
DAB1; AKT1; ACTB; PAFAH1B1
FOXD3; GSK3B; CCDC141; ZNF365
APP; MYH2; RHEB; NDEL1
FOXD3; DAB1; DISC1; ZNF365
FOXD3; GSK3B; DAB1; DISC1
FOXD3; DAB1; CCDC141; DISC1
CCDC88A; MYH2; PAFAH1B1; NDEL1
FOXD3; DAB1; DISC1; ZNF365
FOXD3; DAB1; DISC1; ZNF365
CCDC88A; DISC1; SOX10; ACTB
FOXD3; GSK3B; CCDC141; DISC1
CCDC88A; MYH2; PAFAH1B1; NDEL1
PCM1; CCDC141; ACTB; PAFAH1B1
APP; DIXDC1; AKT1; ACTB
FOXD3; DAB1; CCDC141; DISC1
GSK3B; CCDC88A; DISC1; ACTB
FOXD3; DAB1; DISC1; ZNF365
CCDC88A; CDK5; SOX10; ACTB
GSK3B; APP; MYH2; RHEB
FOXD3; GSK3B; APP; ACTB
FOXD3; DAB1; DISC1; ZNF365
CCDC88A; CCDC141; ACTB; PAFAH1B1
APP; AKT1; ACTB; NDEL1
FOXD3; DAB1; DISC1; ZNF365
FOXD3; DAB1; CCDC141; ACTB
FOXD3; PCM1; AKT1; ACTB
GSK3B; CDK5; ACTB; NDEL1
GSK3B; PCM1; RHEB; ACTB
FOXD3; AKT1; ZNF365; NDEL1

GSK3B; RHEB; SOX10; ACTB
GSK3B; AKT1; DISC1; ZNF365
DAB1; AKT1; ACTB; BBS4
APP; RHEB; ACTB; PAFAH1B1
FOXD3; AKT1; DISC1; ACTB
FOXD3; MYH2; BBS4; PAFAH1B1
FOXD3; APP; DISC1; ZNF365
APP; RHEB; DISC1
BBS4
FOXD3; GSK3B; APP
CDK5
APP; DAB1; SOX10; ACTB
FOXD3; APP; DISC1
FOXD3; APP; DISC1
FOXD3; DAB1
GSK3B; APP; CCDC88A; ACTB
DAB1; DISC1
GSK3B; APP; PCM1; DAB1; RHEB; DISC1; SOX10; BBS4
APP; CCDC88A; CDK5
ACTB; PAFAH1B1; NDEL1
ACTB
APP; DAB1; SOX10
GSK3B; PCM1; DAB1; CDK5; RHEB; BBS4; ACTB; NDEL1
SOX10
FOXD3; DIXDC1
CDK5; ACTB; NDEL1
FOXD3; DAB1
PCM1
GSK3B; ACTB
DIXDC1; ACTB; NDEL1
CCDC88A; APP; AKT1; DISC1; NDEL1
RHEB; ACTB; NDEL1
DAB1; RHEB
APP; CCDC141; ACTB
NDEL1
DISC1; NDEL1
ACTB; NDEL1
CDK5; NDEL1
FOXD3; ACTB
FOXD3; CDK5; RHEB; NDEL1
ZNF365
PCM1; DAB1
APP; DISC1
PCM1; CDK5
CCDC88A; PCM1; DAB1; DISC1
APP
APP; DAB1; DIXDC1; SOX10

CCDC88A; NDEL1
PCM1; BBS4
APP; DAB1; CCDC141
PCM1; RHEB; DIXDC1; NDEL1
GSK3B; RHEB
APP; DIXDC1
GSK3B; APP; DAB1; RHEB; DISC1
AKT1; BBS4; NDEL1
GSK3B; DIXDC1
DIXDC1; BBS4; NDEL1
DAB1; DISC1; ACTB; NDEL1
DAB1; ACTB
FOXD3; CCDC141; NDEL1
DAB1
APP; RHEB; DISC1
DAB1; BBS4
DAB1; BBS4
PCM1; CDK5
DAB1; RHEB; PAFAH1B1
GSK3B; PCM1; NDEL1
APP; DISC1
FOXD3; DISC1
APP; MYH2; BBS4
PCM1; RHEB; BBS4; PAFAH1B1; NDEL1
APP
APP; DISC1; PAFAH1B1
CCDC88A; CDK5
GSK3B; APP; MYH2
DAB1
APP; DISC1
CCDC88A; DAB1; DISC1; ZNF365; NDEL1
FOXD3; DISC1
DAB1; RHEB; DIXDC1
DAB1; CDK5; PAFAH1B1
APP; DAB1; DIXDC1
ACTB; PAFAH1B1; NDEL1
APP; CCDC88A; NDEL1
PCM1; MYH2; SOX10
APP; DAB1; NDEL1
CCDC88A; RHEB; BBS4
FOXD3; RHEB; NDEL1
FOXD3; SOX10; ACTB
CCDC88A; MYH2; NDEL1
PCM1; AKT1; ACTB
FOXD3; DAB1; DISC1
DAB1; DIXDC1; BBS4
FOXD3; APP; ACTB

FOXD3; CCDC88A; PAFAH1B1
GSK3B; ACTB; PAFAH1B1
DAB1; AKT1; SOX10
PCM1; AKT1; ACTB
PCM1; ACTB; BBS4
DAB1; RHEB; DIXDC1
FOXD3; BBS4; NDEL1
FOXD3; APP; NDEL1
AKT1; ACTB; PAFAH1B1
RHEB; DIXDC1; NDEL1
FOXD3; CCDC88A; ACTB
DAB1; ACTB; PAFAH1B1
GSK3B; CCDC88A; DIXDC1
APP; CCDC141; DISC1
FOXD3; DAB1; ZNF365
DAB1; CDK5; CCDC141
FOXD3; AKT1; ZNF365
FOXD3; DAB1; CCDC141
GSK3B; CCDC88A; AKT1
CCDC88A; RHEB; CCDC141
GSK3B; APP; RHEB
FOXD3; DAB1; DISC1
FOXD3; RHEB; ACTB
FOXD3; RHEB; AKT1
CCDC88A; MYH2; PAFAH1B1
AKT1; ACTB; PAFAH1B1
APP; AKT1; ACTB
GSK3B; DIXDC1; BBS4
APP; AKT1; NDEL1
CCDC88A; GSK3B; CDK5; RHEB; ACTB; PAFAH1B1; NDEL1
NDEL1
GSK3B; PCM1; DAB1; RHEB; ACTB
FOXD3; ACTB
GSK3B; MYH2
FOXD3; APP; DAB1; AKT1
GSK3B; DIXDC1; ACTB
GSK3B; BBS4
FOXD3
DIXDC1; AKT1; ACTB
APP; DAB1
ACTB
GSK3B; AKT1; PAFAH1B1
GSK3B; APP; PCM1
PCM1; CDK5; AKT1; BBS4
DAB1; DIXDC1
ZNF365
FOXD3; SOX10

ACTB
GSK3B; ACTB
ZNF365
FOXD3; DAB1; RHEB
ACTB
ACTB
GSK3B; PCM1; DIXDC1; DISC1
FOXD3; DIXDC1; ACTB; PAFAH1B1; NDEL1
DAB1
FOXD3
FOXD3; APP; PCM1; DAB1; RHEB; DIXDC1; AKT1
FOXD3; DIXDC1; AKT1; DISC1; SOX10
CCDC88A; DAB1; DIXDC1
DAB1; DIXDC1; AKT1; ACTB; PAFAH1B1
DAB1; CDK5; DIXDC1; PAFAH1B1
FOXD3; APP
DAB1; RHEB
GSK3B; PCM1; DISC1
ZNF365
FOXD3; APP; DAB1; AKT1; SOX10
CDK5
GSK3B
APP; PAFAH1B1
APP; DAB1; DISC1; PAFAH1B1
GSK3B
DISC1
DISC1
APP; RHEB; PAFAH1B1; ZNF365
CCDC88A
ZNF365
GSK3B; DISC1
FOXD3; GSK3B; DISC1; PAFAH1B1
ACTB
APP; DAB1
CCDC88A; RHEB; DIXDC1; ACTB
FOXD3; GSK3B; RHEB; BBS4
FOXD3
APP; CCDC88A
PCM1; CDK5
SOX10
GSK3B; APP; PCM1; ZNF365
DAB1
FOXD3
APP; CCDC88A
AKT1; ACTB; NDEL1
APP
CDK5

GSK3B; ACTB
BBS4
APP; RHEB; BBS4; ACTB
FOXD3; MYH2
DISC1
GSK3B; PAFAH1B1
RHEB
CDK5
DAB1
GSK3B
FOXD3; DAB1; ACTB
ACTB
CCDC88A; CDK5
CCDC88A; PCM1
PCM1
FOXD3; CCDC88A; PCM1; DISC1; ACTB
DAB1; DIXDC1; DISC1
GSK3B; ACTB
APP
DIXDC1
FOXD3
GSK3B; BBS4
CDK5; DIXDC1; PAFAH1B1
CDK5; PAFAH1B1
FOXD3; CCDC88A
PCM1; DIXDC1
PCM1; DIXDC1
FOXD3; DAB1
ACTB; BBS4
APP; ACTB
FOXD3; AKT1
CCDC88A; PAFAH1B1
GSK3B; APP
APP; DISC1
SOX10; PAFAH1B1
GSK3B; BBS4
DAB1; CCDC141
GSK3B; AKT1
MYH2; ACTB
SOX10; PAFAH1B1
DIXDC1; ACTB
DAB1; ACTB
FOXD3; NDEL1
DAB1; BBS4
RHEB; NDEL1
GSK3B; APP
ACTB; BBS4

AKT1; ACTB
DAB1; CCDC141
CDK5; CCDC141
CDK5; BBS4
RHEB; ACTB
FOXD3; DIXDC1
CDK5; DISC1
DAB1; CCDC141
FOXD3; ZNF365
FOXD3; BBS4
GSK3B; ACTB
DISC1; SOX10
FOXD3; APP
ACTB; BBS4
DAB1; ACTB
APP; ACTB
CCDC88A; NDEL1
ACTB; ZNF365
FOXD3; PAFAH1B1
FOXD3; APP
CCDC141; NDEL1
GSK3B; ACTB
APP; ZNF365
AKT1; SOX10
PCM1; CCDC141
CCDC88A; NDEL1
CCDC88A; NDEL1
DISC1; NDEL1
FOXD3; DIXDC1
NDEL1
CCDC88A; ACTB
APP; DAB1
PAFAH1B1
CCDC88A; NDEL1
NDEL1
CCDC141
ACTB
MYH2
RHEB
RHEB
BBS4
PCM1
ACTB
DAB1
DAB1
DISC1
FOXD3; GSK3B; PCM1

FOXD3
CDK5; NDEL1
FOXD3
APP
DISC1
AKT1; ACTB
DAB1; ZNF365
GSK3B; PCM1
AKT1
DAB1; CDK5
ACTB
AKT1
AKT1; PAFAH1B1
APP
DAB1; ACTB
ACTB
AKT1
CCDC88A
ZNF365
DIXDC1
ZNF365
RHEB
AKT1
DAB1
RHEB
DAB1
PAFAH1B1
PAFAH1B1; NDEL1
DIXDC1
RHEB
ACTB
ACTB
APP
ACTB
NDEL1
CCDC141
RHEB; DIXDC1
PCM1
ZNF365
CCDC141
ACTB
ACTB
CCDC88A
DIXDC1
DIXDC1
DIXDC1
FOXD3

DAB1
ZNF365
CCDC141
CCDC141
BBS4
CCDC141
GSK3B
CCDC141
AKT1
DAB1
CCDC141
CCDC141
DIXDC1
PCM1
RHEB
PCM1
FOXD3
CCDC141
ACTB
ACTB
ACTB
DAB1
CCDC141
ZNF365
CCDC141
MYH2
DISC1
ZNF365
CCDC141
CCDC88A
GSK3B
GSK3B
PAFAH1B1
RHEB
FOXD3
CCDC141
CCDC141
RHEB
CCDC88A
APP
FOXD3
PAFAH1B1
ACTB
RHEB
CCDC141
GSK3B
CCDC141

CCDC141
FOXD3
APP
AKT1
APP
RHEB
ACTB
CCDC141
DAB1
FOXD3
ACTB
ACTB
APP
APP
NDEL1
ACTB
BBS4
DAB1
MYH2
ACTB

TF	PMID	Study	Cell Type	Organism	Overlap	*p* -value
AR	22383394	ChIP-Seq	PROSTATE CANCER	Human	5/1857	0.0264
CCND1	20090754	ChIP-ChIP	RETINA	Mouse	7/2137	0.0025
CLOCK	20551151	ChIP-Seq	293T	Human	3/407	0.0064
CREB1	20920259	ChIP-Seq	GC1-SPG	Mouse	8/3057	0.0046
CREB1	23762244	ChIP-Seq	HIPPOCAMPUS	Rat	6/2393	0.0199
EGR1	19374776	ChIP-ChIP	THP-1	Human	2/1968	0.0019
ELK1	22589737	ChIP-Seq	MCF10A	Human	4/928	0.0102
EZH2	27304074	Chip-Seq	ESCs	Mouse	3/885	0.0493
HCFC1	20581084	ChIP-Seq	MESCs	Mouse	2/306	0.0336
HNF4A	19822575	ChIP-Seq	HepG2	Human	12/6083	0.0032
KLF4	18358816	ChIP-ChIP	MESCs	Mouse	7/1700	0.0006
MEF2A	21415370	ChIP-Seq	HL-1	Mouse	4/1048	0.0155
MYBL2	22936984	ChIP-ChIP	MESCs	Mouse	5/2250	0.0543
NUCKS1	24931609	ChIP-Seq	HEPATOCYTES	Mouse	3/588	0.0173
PHC1	16625203	ChIP-ChIP	MESCs	Mouse	3/922	0.0546
PIAS1	25552417	ChIP-Seq	VCAP	Human	3/749	0.0324
POU5F1	18347094	ChIP-ChIP	MESCs	Mouse	5/2109	0.0429
RING1B	27294783	Chip-Seq	ESCs	Mouse	6/2000	0.0086
RNF2	16625203	ChIP-ChIP	MESCs	Mouse	4/1219	0.0255
RXR	22158963	ChIP-Seq	LIVER	Mouse	5/2000	0.0351
SCL	21571218	ChIP-Seq	MEGAKARYOCYTES	Human	5/1784	0.0226
SIN3B	21632747	ChIP-Seq	MESCs	Mouse	8/4302	0.0350
SMAD2	18955504	ChIP-ChIP	HaCaT	Human	7/1936	0.0014
SMAD3	18955504	ChIP-ChIP	HaCaT	Human	7/1936	0.0014
SMC4	20622854	ChIP-Seq	HELA	Human	5/2000	0.0351
SOX3	22085726	ChIP-Seq	MUSCLE	Mouse	6/2000	0.0086
SPI1	23127762	ChIP-Seq	K562	Human	4/1389	0.0388
STAT3	23295773	ChIP-Seq	U87	Human	10/3165	0.0002
TAL1	20566737	ChIP-Seq	PRIMARY FETEL LIVER ERYTHROID	Mouse	5/1875	0.0274
TAL1	26923725	Chip-Seq	HPCs	Mouse	5/2000	0.0351
TCF3	18467660	ChIP-ChIP	MESCs	Mouse	5/1388	0.0082
TRIM28	19339689	ChIP-ChIP	MESCs	Mouse	8/3072	0.0047
TTF2	22483619	ChIP-Seq	HELA	Human	5/1512	0.0116
YAP1	20516196	ChIP-Seq	MESCs	Mouse	6/2329	0.0176
ZFP281	18757296	ChIP-ChIP	E14	Mouse	6/2004	0.0086

Adjusted *p* -value	Old *p* -value	Old Adjusted *p* -value	*Z* -score	Combined Score
0.5584	0.0006	0.0132	-1.4796	0.8621
0.2023	0.0000	0.0006	-1.9778	3.1601
0.3019	0.0005	0.0123	-2.6537	3.1784
0.2567	0.0000	0.0006	-1.1482	1.5613
0.4865	0.0002	0.0068	-1.1778	0.8486
0.1835	0.0003	0.0096	-3.2348	5.4847
0.3330	0.0004	0.0107	-2.1846	2.4022
0.5584	0.0046	0.0360	-1.9725	1.1493
0.5584	0.0062	0.0360	-3.1744	1.8496
0.2233	0.0000	0.0001	-0.5905	0.8854
0.1561	0.0000	0.0003	-1.9631	3.6462
0.4446	0.0006	0.0132	-2.8593	2.3179
0.5584	0.0014	0.0201	-1.9800	1.1537
0.4521	0.0014	0.0208	-2.1723	1.7242
0.5584	0.0051	0.0360	-2.5753	1.5005
0.5584	0.0029	0.0360	-2.1700	1.2644
0.5584	0.0010	0.0166	-1.5688	0.9141
0.3019	0.0001	0.0035	-1.4359	1.7198
0.5584	0.0011	0.0166	-2.4055	1.4016
0.5584	0.0008	0.0141	-1.2774	0.7443
0.5265	0.0005	0.0123	-1.4664	0.9408
0.5584	0.0001	0.0043	-0.6810	0.3968
0.1701	0.0000	0.0005	-1.8045	3.1963
0.1701	0.0000	0.0005	-1.7985	3.1857
0.5584	0.0008	0.0141	-1.4915	0.8690
0.3019	0.0001	0.0035	-1.3870	1.6613
0.5584	0.0018	0.0239	-1.6492	0.9609
0.1100	0.0000	0.0000	-1.2580	2.7764
0.5584	0.0006	0.0132	-1.5121	0.8810
0.5584	0.0008	0.0141	-1.3317	0.7759
0.3019	0.0001	0.0056	-2.4847	2.9760
0.2567	0.0000	0.0006	-1.5161	2.0616
0.3560	0.0002	0.0068	-1.6588	1.7131
0.4521	0.0002	0.0063	-1.3162	1.0447
0.3019	0.0001	0.0035	-2.1449	2.5689

Genes involved in neuronal migration

CCDC88A; CCDC141; DISC1; BBS4; ZNF365
GSK3B; APP; DAB1; CDK5; RHEB; ACTB; NDEL1
AKT1; ACTB; NDEL1
GSK3B; CCDC88A; CDK5; RHEB; SOX10; ACTB; PAFAH1B1; NDEL1
GSK3B; CDK5; RHEB; ACTB; PAFAH1B1; NDEL1
PCM1; AKT1
PCM1; DIXDC1; ACTB; BBS4
APP; DAB1; SOX10
PCM1; CDK5
GSK3B; PCM1; DAB1; CDK5; RHEB; DIXDC1; AKT1; DISC1; ACTB; PAFAH1B1; ZNF365; NDEL1
FOXD3; GSK3B; DAB1; CDK5; RHEB; DIXDC1; ACTB
APP; CCDC88A; RHEB; DISC1
FOXD3; RHEB; DIXDC1; ACTB; NDEL1
GSK3B; CDK5; RHEB
GSK3B; RHEB; DISC1
APP; DAB1; BBS4
FOXD3; DAB1; RHEB; AKT1; NDEL1
FOXD3; APP; CCDC88A; DAB1; CCDC141; SOX10
APP; DAB1; RHEB; DISC1
PCM1; DIXDC1; AKT1; CCDC141; ACTB
GSK3B; DISC1; ACTB; ZNF365; NDEL1
CCDC88A; PCM1; DAB1; CDK5; CCDC141; BBS4; ACTB; NDEL1
APP; CCDC88A; CDK5; RHEB; ACTB; PAFAH1B1; ZNF365
APP; CCDC88A; CDK5; RHEB; ACTB; PAFAH1B1; ZNF365
CDK5; AKT1; SOX10; ACTB; BBS4
PCM1; CDK5; DIXDC1; AKT1; CCDC141; NDEL1
DAB1; RHEB; BBS4; PAFAH1B1
FOXD3; GSK3B; APP; PCM1; DAB1; CDK5; RHEB; DISC1; BBS4; ZNF365
RHEB; AKT1; DISC1; ACTB; NDEL1
CCDC88A; PCM1; RHEB; DISC1; PAFAH1B1
GSK3B; PCM1; RHEB; AKT1; DISC1
FOXD3; GSK3B; APP; DAB1; CDK5; RHEB; DISC1; NDEL1
CDK5; DIXDC1; DISC1; ACTB; NDEL1
GSK3B; APP; DAB1; AKT1; ACTB; PAFAH1B1
FOXD3; GSK3B; PCM1; DAB1; ACTB; PAFAH1B1

List of 32 TF regulating genes (N=18) involved in migration, after removing duplicates

AR
CCND1
CLOCK
CREB1
EGR1
ELK1
EZH2
HCFC1
HNF4A
KLF4
MEF2A
MYBL2
NUCKS1
PHC1
PIAS1
POU5F1
RNF2
RXR
SCL
SIN3B
SMAD2
SMAD3
SMC4
SOX3
SPI1
STAT3
TAL1
TCF3
TRIM28
TTF2
YAP1
ZFP281

TFs (From ChEA database) regulating expression of genes involved in migration
STAT3_23295773_ChIP-Seq_U87_Human
KLF4_18358816_ChIP-ChIP_MESCs_Mouse
SMAD2_18955504_ChIP-ChIP_HaCaT_Human
SMAD3_18955504_ChIP-ChIP_HaCaT_Human
EGR1_19374776_ChIP-ChIP_THP-1_Human
CCND1_20090754_ChIP-ChIP_RETINA_Mouse
HNF4A_19822575_ChIP-Seq_HepG2_Human
CREB1_20920259_ChIP-Seq_GC1-SPG_Mouse
TRIM28_19339689_ChIP-ChIP_MESCs_Mouse
CLOCK_20551151_ChIP-Seq_293T_Human
TCF3_18467660_ChIP-ChIP_MESCs_Mouse
RING1B_27294783_Chip-Seq_ESCs_Mouse
SOX3_22085726_ChIP-Seq_MUSCLE_Mouse
ZFP281_18757296_ChIP-ChIP_E14_Mouse
ELK1_22589737_ChIP-Seq_MCF10A_Human
TTF2_22483619_ChIP-Seq_HELA_Human
MEF2A_21415370_ChIP-Seq_HL-1_Mouse
NUCKS1_24931609_ChIP-Seq_HEPATOCYTES_Mouse
YAP1_20516196_ChIP-Seq_MESCs_Mouse
CREB1_23762244_ChIP-Seq_HIPPOCAMPUS_Rat
SCL_21571218_ChIP-Seq_MEGAKARYOCYTES_Human
RNF2_16625203_ChIP-ChIP_MESCs_Mouse
AR_22383394_ChIP-Seq_PROSTATE_CANCER_Human
TAL1_20566737_ChIP-Seq_PRIMARY_FETAL_LIVER_ERYTHROID_Mouse
PIAS1_25552417_ChIP-Seq_VCAP_Human
HCFC1_20581084_ChIP-Seq_MESCs_Mouse
SIN3B_21632747_ChIP-Seq_MESCs_Mouse
SMC4_20622854_ChIP-Seq_HELA_Human
TAL1_26923725_Chip-Seq_HPCs_Mouse
RXR_22158963_ChIP-Seq_LIVER_Mouse
SPI1_23127762_ChIP-Seq_K562_Human
POU5F1_18347094_ChIP-ChIP_MESCs_Mouse
EZH2_27304074_Chip-Seq_ESCs_Mouse
MYBL2_22936984_ChIP-ChIP_MESCs_Mouse
PHC1_16625203_ChIP-ChIP_MESCs_Mouse
SRY_25088423_ChIP-ChIP_EMBRYONIC_GONADS_Mouse
CTNNB1_20460455_ChIP-Seq_HCT116_Human
WT1_20215353_ChIP-ChIP_NEPHRON_PROGENITOR_Mouse
DROSHA_22980978_ChIP-Seq_HELA_Human
NR0B1_18358816_ChIP-ChIP_MESCs_Mouse
PRDM5_23873026_ChIP-Seq_MEFs_Mouse
POU3F2_20337985_ChIP-ChIP_501MEL_Human
PADI4_21655091_ChIP-ChIP_MCF-7_Human
ZFX_18555785_ChIP-Seq_MESCs_Mouse
SALL4_18804426_ChIP-ChIP_MESCs_Mouse
PAX3-FKHR_20663909_ChIP-Seq_RHABDOMYOSARCOMA_Human

ELF5_23300383_ChIP-Seq_T47D_Human
ZFP322A_24550733_ChIP-Seq_MESCs_Mouse
SMARCA4_20176728_ChIP-ChIP_TSCs_Mouse
SUZ12_18692474_ChIP-Seq_MEFs_Mouse
GATA1_19941827_ChIP-Seq_MEL_Mouse
KLF5_18264089_ChIP-ChIP_MESCs_Mouse
KLF2_18264089_ChIP-ChIP_MESCs_Mouse
KLF4_18264089_ChIP-ChIP_MESCs_Mouse
GABP_19822575_ChIP-Seq_HepG2_Human
POU5F1_18700969_ChIP-ChIP_MESCs_Mouse
SUZ12_18692474_ChIP-Seq_MESCs_Mouse
MEIS1_26923725_Chip-Seq_HEMOGENIC-ENDOTHELIUM_Mouse
AR_19668381_ChIP-Seq_PC3_Human
ZFP281_18358816_ChIP-ChIP_MESCs_Mouse
SUZ12_18974828_ChIP-Seq_MESCs_Mouse
PRDM14_21183938_ChIP-Seq_MESCs_Mouse
FOXA1_27197147_Chip-Seq_ENDOMETRIOID-ADENOCARCINOMA_Human
SPI1_20517297_ChIP-Seq_HL60_Human
OCT4_18692474_ChIP-Seq_MEFs_Mouse
CJUN_26792858_Chip-Seq_BT549_Human
STAT1_17558387_ChIP-Seq_HELA_Human
TBL1_22424771_ChIP-Seq_293T_Human
CBX2_22325352_ChIP-Seq_293T-Rex_Human
ETV1_20927104_ChIP-Seq_GIST48_Human
GATA2_21186366_ChIP-Seq_BM-HSCs_Mouse
CDX2_21074721_ChIP-Seq_CACO-2_Mouse
NFYB_21822215_ChIP-Seq_K562_Human
CSB_26484114_Chip-Seq_FIBROBLAST_Human
P300_19829295_ChIP-Seq_ESCs_Human
LUZP1_20508642_ChIP-Seq_ESCs_Mouse
KLF5_25053715_ChIP-Seq_YYC3_Human
MAF_26560356_Chip-Seq_TH2_Human
PCGF4_22325352_ChIP-Seq_293T-Rex_Human
SMRT_22465074_ChIP-Seq_MACROPHAGES_Mouse
HNFA_21074721_ChIP-Seq_CACO-2_Human
JARID1B-DAIN_22020125_ChIP-Seq_ESCs_Mouse
NEUROD2_26341353_ChIP-Seq_CORTEX_Mouse
CHD1_26751641_Chip-Seq_LNCaP_Human
NFYA_21822215_ChIP-Seq_K562_Human
PU.1_20513432_ChIP-Seq_MACROPHAGES_Mouse
NCOR_22465074_ChIP-Seq_MACROPHAGES_Mouse
PHF8_20622853_ChIP-Seq_HELA_Human
ZFP281_27345836_Chip-Seq_ESCs_Mouse
OLIG2_26023283_ChIP-Seq_AINV15_Mouse
NFI_21473784_ChIP-Seq_ESCs_Mouse
MYB_26560356_Chip-Seq_TH2_Human
CREB1_26743006_Chip-Seq_LNCaP-abl_Human

TAF2_19829295_ChIP-Seq_ESCs_Human
FOXM1_26456572_ChIP-Seq_MCF-7_Human
KAP1_27257070_Chip-Seq_ESCs_Mouse
EBNA1_20929547_Chip-Seq_RAJI-cells_Human
KLF4_26769127_Chip-Seq_PDAC-Cell_line_Human
MEIS1_26253404_ChIP-Seq_OPTIC_CUPS_Mouse
ELF3_26769127_Chip-Seq_PDAC-Cell_line_Human
SUZ12_16625203_ChIP-ChIP_MESCs_Mouse
VDR_22108803_ChIP-Seq_LS180_Human
SOX2_16153702_ChIP-ChIP_HESCs_Human
NOTCH1_17114293_ChIP-ChIP_T-ALL_Human
OLIG2_23332759_ChIP-Seq_OLIGODENDROCYTES_Mouse
RNF2_18974828_ChIP-Seq_MESCs_Mouse
EZH2_18974828_ChIP-Seq_MESCs_Mouse
CEBPB_26923725_Chip-Seq_HEMANGIOBLAST_Mouse
DMRT1_23473982_ChIP-Seq_TESTES_Mouse
BMI1_19503595_ChIP-Seq_MEFsC_Mouse
MITF_21258399_ChIP-Seq_MELANOMA_Human
SOX9_24532713_ChIP-Seq_HFSC_Mouse
MYC_19079543_ChIP-ChIP_MESCs_Mouse
THRA_23701648_ChIP-Seq_CEREBELLUM_Mouse
RNF2_27304074_Chip-Seq_ESCs_Mouse
FLI1_21571218_ChIP-Seq_MEGAKARYOCYTES_Human
AR_21572438_ChIP-Seq_LNCaP_Human
NACC1_18358816_ChIP-ChIP_MESCs_Mouse
KLF4_19030024_ChIP-ChIP_MESCs_Mouse
SOX2_18358816_ChIP-ChIP_MESCs_Mouse
HOXD13_18407260_ChIP-ChIP_DEVELOPING-LIMBS_Mouse
VDR_21846776_ChIP-Seq_THP-1_Human
XRN2_22483619_ChIP-Seq_HELA_Human
TP53_23651856_ChIP-Seq_MEFs_Mouse
CNOT3_19339689_ChIP-ChIP_MESCs_Mouse
TP53_18474530_ChIP-ChIP_U2OS_Human
ISL1_27105846_Chip-Seq_CPCs_Mouse
ETS2_20176728_ChIP-ChIP_TROPHOBLAST_STEM_CELLS_Mouse
GATA3_20176728_ChIP-ChIP_TSCs_Mouse
PPARG_23326641_ChIP-Seq_C3H10T1-2_Mouse
CHD1_19587682_ChIP-ChIP_MESCs_Mouse
EWS_26573619_Chip-Seq_HEK293_Human
KLF4_18555785_ChIP-Seq_MESCs_Mouse
ESR1_21235772_ChIP-Seq_MCF-7_Human
SOX2_19030024_ChIP-ChIP_MESCs_Mouse
FOXP2_23625967_ChIP-Seq_PFSK-1_AND_SK-N-MC_Human
THAP11_20581084_ChIP-Seq_MESCs_Mouse
SMAD4_21799915_ChIP-Seq_A2780_Human
TAF15_26573619_Chip-Seq_HEK293_Human
SMARCA4_23332759_ChIP-Seq_OLIGODENDROCYTES_Mouse

TAF7L_23326641_ChIP-Seq_C3H10T1-2_Mouse
ELK1_19687146_ChIP-ChIP_HELA_Human
SOX9_26525672_Chip-Seq_HEART_Mouse
SOX2_20726797_ChIP-Seq_SW620_Human
FOXM1_25889361_ChIP-Seq_OE33_AND_U2OS_Human
EOMES_21245162_ChIP-Seq_HESCs_Human
WT1_25993318_ChIP-Seq_PODOCYTE_Human
E2F1_17053090_ChIP-ChIP_MCF-7_Human
NR1I2_20693526_ChIP-Seq_LIVER_Mouse
EOMES_20176728_ChIP-ChIP_TSCs_Mouse
GATA1_21571218_ChIP-Seq_MEGAKARYOCYTES_Human
GATA1_19941826_ChIP-Seq_K562_Human
STAT3_24763339_ChIP-Seq_IMN-ESCs_Mouse
GBX2_23144817_ChIP-Seq_PC3_Human
PPARG_20176806_ChIP-Seq_3T3-L1_Mouse
CBP_20019798_ChIP-Seq_JUKART_Human
IRF4_20064451_ChIP-Seq_CD4+T_Mouse
EST1_17652178_ChIP-ChIP_JURKAT_Human
LMO2_26923725_Chip-Seq_HEMANGIOBLAST_Mouse
HOXB4_20404135_ChIP-ChIP_EML_Mouse
EP300_21415370_ChIP-Seq_HL-1_Mouse
SUZ12_18555785_ChIP-Seq_MESCs_Mouse
TFAP2A_17053090_ChIP-ChIP_MCF-7_Human
KDM5B_21448134_ChIP-Seq_MESCs_Mouse
CDX2_19796622_ChIP-Seq_MESCs_Mouse
FOXM1_26100407_CHIP-SEQ_Hek293_flp-in_Human
SREBP2_21459322_ChIP-Seq_LIVER_Mouse
SOX17_20123909_ChIP-Seq_XEN_Mouse
NANOG_18700969_ChIP-ChIP_MESCs_Mouse
JARID2_20064375_ChIP-Seq_MESCs_Mouse
TCF4_23295773_ChIP-Seq_U87_Human
TP53_20018659_ChIP-ChIP_R1E_Mouse
TCFCP2L1_18555785_ChIP-Seq_MESCs_Mouse
SOX2_18692474_ChIP-Seq_MEFs_Mouse
TET1_21490601_ChIP-Seq_MESCs_Mouse
NELFA_20434984_ChIP-Seq_ESCs_Mouse
FLI1_21867929_ChIP-Seq_TH2_Mouse
GATA3_22897851_ChIP-Seq_JUKARTE6-1_Human
KDM2B_26808549_Chip-Seq_REH_Human
RUNX1_22897851_ChIP-Seq_JUKARTE6-1_Human
P63_20808887_ChIP-Seq_KERATINOCYTES_Human
LXR_22292898_ChIP-Seq_THP-1_Human
TAL1_21186366_ChIP-Seq_BM-HSCs_Mouse
TBX20_22328084_ChIP-Seq_HEART_Mouse
RBPJ_21746931_ChIP-Seq_IB4_Human
ESET_19884257_ChIP-Seq_ESCs_Mouse
SMAD3_21741376_ChIP-Seq_ESCs_Human

ETS1_21867929_ChIP-Seq_TH2_Mouse
SPI1_26923725_Chip-Seq_HPCs_Mouse
CTCF_27219007_Chip-Seq_Bcells_Human
TBX20_22080862_ChIP-Seq_HEART_Mouse
BCL6_25482012_ChIP-Seq_CML-JURL-MK1_Human
FOXH1_21741376_ChIP-Seq_ESCs_Human
FOXA1_26743006_Chip-Seq_LNCaP-abl_Human
CEBPB_26923725_Chip-Seq_HEMOGENIC-ENDOTHELIUM_Mouse
DPY_21335234_ChIP-Seq_ESCs_Mouse
KLF5_20875108_ChIP-Seq_MESCs_Mouse
PU.1_20513432_ChIP-Seq_Bcells_Mouse
UBF1/2_26484160_Chip-Seq_HMEC-DERIVED_Human
GATA4_25053715_ChIP-Seq_YYC3_Human
AR_21915096_ChIP-Seq_LNCaP-1F5_Human
GATA6_21074721_ChIP-Seq_CACO-2_Mouse
FOXH1_21741376_ChIP-Seq_EPCs_Human
CREB1_26743006_Chip-Seq_LNCaP_Human
E2F1_18555785_Chip-Seq_ESCs_Mouse
SMC1_22415368_ChIP-Seq_MEFs_Mouse
RARB_27405468_Chip-Seq_BRAIN_Mouse
FLI1_26923725_Chip-Seq_HEMOGENIC-ENDOTHELIUM_Mouse
GATA6_21074721_ChIP-Seq_CACO-2_Human
KDM2B_26808549_Chip-Seq_K562_Human
CTCF_27219007_Chip-Seq_ERYTHROID_Human
OCT4_21477851_ChIP-Seq_ESCs_Mouse
KDM2B_26808549_Chip-Seq_SIL-ALL_Human
SMC3_22415368_ChIP-Seq_MEFs_Mouse
FLI1_26923725_Chip-Seq_MACROPHAGESS_Mouse
SOX2_27498859_Chip-Seq_STOMACH_Mouse
CREM_20920259_ChIP-Seq_GC1-SPG_Mouse
ZIC3_20872845_ChIP-ChIP_MESCs_Mouse
MYC_19030024_ChIP-ChIP_MESCs_Mouse
HSF1_23293686_ChIP-Seq_STHDH_STRIATAL_Mouse
FOXP2_21765815_ChIP-ChIP_NEURO2A_Mouse
MTF2_20144788_ChIP-Seq_MESCs_Mouse
TAL1_20887958_ChIP-Seq_HPC-7_Mouse
TFAP2C_20629094_ChIP-Seq_MCF-7_Human
RCOR2_21632747_ChIP-Seq_MESCs_Mouse
EP300_20729851_ChIP-Seq_FORBRAIN_MIDBRAIN_LIMB_HEART_Mouse
KLF4_25985364_ChIP-Seq_ATHEROSCLEROSIS_LESION_Mouse
SMAD4_19686287_ChIP-ChIP_HaCaT_Human
REST_19997604_ChIP-ChIP_NEURONS_Mouse
SMARCD1_25818293_ChIP-Seq_ESCs_Mouse
CUX1_19635798_ChIP-ChIP_MULTIPLE_HUMAN_CANCER_TYPES_Human
NANOG_18358816_ChIP-ChIP_MESCs_Mouse
ESR2_21235772_ChIP-Seq_MCF-7_Human
JARID2_20075857_ChIP-Seq_MESCs_Mouse

ESR1_17901129_ChIP-ChIP_LIVER_Mouse
VDR_24763502_ChIP-Seq_THP-1_Human
TP53_16413492_ChIP-PET_HCT116_Human
TCF3_18347094_ChIP-ChIP_MESCs_Mouse
CIITA_25753668_ChIP-Seq_RAJI_Human
YY1_21170310_ChIP-Seq_MESCs_Mouse
SPI1_23547873_ChIP-Seq_NB4_Human
E2F1_18555785_ChIP-Seq_MESCs_Mouse
TCF4_18268006_ChIP-ChIP_LS174T_Human
LMO2_26923725_Chip-Seq_MACROPHAGESS_Mouse
EGR1_20690147_ChIP-Seq_ERYTHROLEUKEMIA_Human
TP63_22573176_ChIP-Seq_HFKS_Human
TEAD4_22529382_ChIP-Seq_TROPHECTODERM_Mouse
POU5F1_18692474_ChIP-Seq_MESCs_Mouse
SOX2_18692474_ChIP-Seq_MESCs_Mouse
MYC_20876797_ChIP-ChIP_MEDULLOBLASTOMA_Human
TAL1_26923725_Chip-Seq_HEMANGIOBLAST_Mouse
SFPI1_20887958_ChIP-Seq_HPC-7_Mouse
NFE2L2_22581777_ChIP-Seq_LYMPHOBLASTOID_Human
SUZ12_20075857_ChIP-Seq_MESCs_Mouse
NANOG_18555785_ChIP-Seq_MESCs_Mouse
FUS_26573619_Chip-Seq_HEK293_Human
YY1_23942234_ChIP-Seq_MYOBLASTS_AND_MYOTUBES_Mouse
SOX2_21211035_ChIP-Seq_LN229_Gbm
RXR_22108803_ChIP-Seq_LS180_Human
TEAD4_26923725_Chip-Seq_HEMANGIOBLAST_Mouse
POU5F1_26923725_Chip-Seq_MESODERM_Mouse
PPARD_21283829_ChIP-Seq_MYOFIBROBLAST_Human
NFIB_24661679_ChIP-Seq_LUNG_Mouse
EWS-FLI1_20517297_ChIP-Seq_SK-N-MC_Human
ZNF217_24962896_ChIP-Seq_MCF-7_Human
NANOG_16518401_ChIP-PET_MESCs_Mouse
NR1H3_23393188_ChIP-Seq_ATHEROSCLEROTIC-FOAM_Human
CTCF_18555785_ChIP-Seq_MESCs_Mouse
PPARG_20887899_ChIP-Seq_3T3-L1_Mouse
TRIM28_17542650_ChIP-ChIP_NTERA2_Human
POU5F1_16153702_ChIP-ChIP_HESCs_Human
CEBPB_20176806_ChIP-Seq_THIOMACROPHAGE_Mouse
ETS1_20019798_ChIP-Seq_JURKAT_Human
CTNNB1_20615089_ChIP-ChIP_FETAL_BRAIN_Human
TP63_23658742_ChIP-Seq_EP156T_Human
TDRD3_21172665_ChIP-Seq_MCF-7_Human
STAT1_20625510_ChIP-Seq_HELA_Human
SPI1_20176806_ChIP-Seq_THIOMACROPHAGE_Mouse
TCFAP2C_20176728_ChIP-ChIP_TROPHOBLAST_STEM_CELLS_Mouse
CRX_20693478_ChIP-Seq_ADULT_RETINA_Mouse
PDX1_19855005_ChIP-ChIP_MIN6_Mouse

SPI1_22096565_ChIP-ChIP_GC-B_Mouse
P53_22127205_ChIP-Seq_FIBROBLAST_Human
FOXP1_21924763_ChIP-Seq_HESCs_Human
NANOG_16153702_ChIP-ChIP_HESCs_Human
AHR_22903824_ChIP-Seq_MCF-7_Human
DACH1_20351289_ChIP-Seq_MDA-MB-231_Human
FOXO3_23340844_ChIP-Seq_DLD1_Human
SREBP1_19666523_ChIP-Seq_LIVER_Mouse
POU5F1_18358816_ChIP-ChIP_MESCs_Mouse
DCP1A_22483619_ChIP-Seq_HELA_Human
RCOR3_21632747_ChIP-Seq_MESCs_Mouse
IRF8_22096565_ChIP-ChIP_GC-B_Mouse
TET1_21451524_ChIP-Seq_MESCs_Mouse
GFI1B_20887958_ChIP-Seq_HPC-7_Mouse
TFEB_21752829_ChIP-Seq_HELA_Human
RUNX1_21571218_ChIP-Seq_MEGAKARYOCYTES_Human
FOXA2_19822575_ChIP-Seq_HepG2_Human
SOX9_25088423_ChIP-ChIP_EMBRYONIC_GONADS_Mouse
PPARG_20176806_ChIP-Seq_THIOMACROPHAGE_Mouse
NANOG_21062744_ChIP-ChIP_HESCs_Human
RUNX1_26923725_Chip-Seq_HPCs_Mouse
SPI1_22790984_ChIP-Seq_ERYTHROLEUKEMIA_Mouse
NANOG_18692474_ChIP-Seq_MESCs_Mouse
NANOG_18692474_ChIP-Seq_MEFs_Mouse
STAT3_18555785_ChIP-Seq_MESCs_Mouse
MYB_26560356_Chip-Seq_TH1_Human
CEBPA_26348894_ChIP-Seq_LIVER_Mouse
SOX2_21211035_ChIP-Seq_LN229_Human
PHF8_20622854_ChIP-Seq_HELA_Human
SMAD4_21741376_ChIP-Seq_EPCs_Human
RACK7_27058665_Chip-Seq_MCF-7_Human
SOX6_21985497_ChIP-Seq_MYOTUBES_Mouse
RUNX1_27514584_Chip-Seq_MCF-7_Human
KDM2B_26808549_Chip-Seq_JURKAT_Human
MNX1_26342078_ChIP-Seq_MIN6-4N_Mouse
GATA3_24758297_ChIP-Seq_MCF-7_Human
TCFCP2L1_18555785_Chip-Seq_ESCs_Mouse
ATF3_27146783_Chip-Seq_COLON_Human
OCT4_20526341_ChIP-Seq_ESCs_Human
SOX3_22085726_ChIP-Seq_NPCs_Mouse
NANOG_20526341_ChIP-Seq_ESCs_Human
CTCF_21964334_Chip-Seq_Bcells_Human
SOX11_22085726_ChIP-Seq_ESNs_Mouse
UBF1/2_26484160_Chip-Seq_FIBROBLAST_Human
GATA3_26560356_Chip-Seq_TH2_Human
RARA_24833708_ChIP-Seq_LIVER_Mouse
P68_20966046_ChIP-Seq_HELA_Human

KDM2B_26808549_Chip-Seq_HPB-ALL_Human
NANOG_18555785_Chip-Seq_ESCs_Mouse
PPARA_22158963_ChIP-Seq_LIVER_Mouse
CEBPB_22108803_ChIP-Seq_LS180_Human
SMAD4_21741376_ChIP-Seq_ESCs_Human
FOXA1_25552417_ChIP-Seq_VCAP_Human
TCF21_26020271_ChIP-Seq_SMOOTH_MUSCLE_Human
CMYC_18555785_Chip-Seq_ESCs_Mouse
HOXB7_26014856_ChIP-Seq_BT474_Human
KAP1_22055183_ChIP-Seq_ESCs_Mouse
TCF7_22412390_ChIP-Seq_EML_Mouse
JUN_26020271_ChIP-Seq_SMOOTH_MUSCLE_Human
FOXA1_21915096_ChIP-Seq_LNCaP-1F5_Human
BCOR_27268052_Chip-Seq_Bcells_Human
SMAD2/3_21741376_ChIP-Seq_EPCs_Human
P63_26484246_Chip-Seq_KERATINOCYTES_Human
PU.1_20176806_ChIP-Seq_MACROPHAGES_Mouse
UBF1/2_26484160_Chip-Seq_HMECs_Human
P300_27058665_Chip-Seq_ZR-75-30cells_Human
SUZ12_27294783_Chip-Seq_ESCs_Mouse
LXR_22158963_ChIP-Seq_LIVER_Mouse
CEBPB_24764292_ChIP-Seq_MC3T3_Mouse
P53_21459846_ChIP-Seq_SAOS-2_Human
BRD4_27068464_Chip-Seq_AML-cells_Mouse
CDX2_22108803_ChIP-Seq_LS180_Human
PU_27001747_Chip-Seq_BMDM_Mouse
CDX2_21402776_ChIP-Seq_INTESTINAL-VILLUS_Mouse
GATA1_19941827_ChIP-Seq_MEL86_Mouse
FLI1_21867929_ChIP-Seq_CD8_Mouse
DNAJC2_21179169_ChIP-ChIP_NT2_Human
HOXC9_25013753_ChIP-Seq_NEUROBLASTOMA_BE2-C_Human
SETDB1_19884255_ChIP-Seq_MESCs_Mouse
NR3C1_23031785_ChIP-Seq_PC12_Mouse
GATA4_21415370_ChIP-Seq_HL-1_Mouse
MYB_21317192_ChIP-Seq_ERMYB_Mouse
EGR1_23403033_ChIP-Seq_LIVER_Mouse
TP53_22127205_ChIP-Seq_IMR90_Human
GRHL2_25758223_ChIP-Seq_PLACENTA_Mouse
ETV2_25802403_ChIP-Seq_MESCs_Mouse
AUTS2_25519132_ChIP-Seq_293T-REX_Human
GABP_17652178_ChIP-ChIP_JURKAT_Human
RUNX1_17652178_ChIP-ChIP_JURKAT_Human
KDM5A_27292631_Chip-Seq_BREAST_Human
SMAD3_22036565_ChIP-Seq_ESCs_Mouse
TCF7L2_21901280_ChIP-Seq_H4IIE_Rat
ARNT_22903824_ChIP-Seq_MCF-7_Human
ASH2L_23239880_ChIP-Seq_MESCs_Mouse

RNF2_27304074_Chip-Seq_NSC_Mouse
VDR_23849224_ChIP-Seq_CD4+_Human
BMI1_23680149_ChIP-Seq_NPCS_Mouse
ERG_20517297_ChIP-Seq_VCAP_Human
ZFP57_27257070_Chip-Seq_ESCs_Mouse
TBX5_21415370_ChIP-Seq_HL-1_Mouse
PBX1_22567123_ChIP-ChIP_OVCAR3_Human
BRD4_25478319_ChIP-Seq_HGPS_Human
HNF4A_19761587_ChIP-ChIP_CACO-2_Human
REST_21632747_ChIP-Seq_MESCs_Mouse
KLF1_20508144_ChIP-Seq_FETAL-LIVER-ERYTHROID_Mouse
E2F1_21310950_ChIP-Seq_MCF-7_Human
RCOR1_19997604_ChIP-ChIP_NEURONS_Mouse
RELA_24523406_ChIP-Seq_FIBROSARCOMA_Human
GATA2_19941826_ChIP-Seq_K562_Human
MYC_18555785_ChIP-Seq_MESCs_Mouse
EKLF_21900194_ChIP-Seq_ERYTHROCYTE_Mouse
Nerf2_26677805_Chip-Seq_MACROPHAGESS_Mouse
TP53_22573176_ChIP-Seq_HFKS_Human
TCF3_18692474_ChIP-Seq_MESCs_Mouse
BACH1_22875853_ChIP-PCR_HELA_AND_SCP4_Human
GF1_26923725_Chip-Seq_HPCs_Mouse
ESRRB_18555785_ChIP-Seq_MESCs_Mouse
SMAD3_21741376_ChIP-Seq_HESCs_Human
ZFP42_18358816_ChIP-ChIP_MESCs_Mouse
POU5F1_16518401_ChIP-PET_MESCs_Mouse
JUN_21703547_ChIP-Seq_K562_Human
E2F4_21247883_ChIP-Seq_LYMPHOBLASTOID_Human
SRF_21415370_ChIP-Seq_HL-1_Mouse
GF1B_26923725_Chip-Seq_HPCs_Mouse
CEBPD_21427703_ChIP-Seq_3T3-L1_Mouse
LMO2_20887958_ChIP-Seq_HPC-7_Mouse
AR_25329375_ChIP-Seq_VCAP_Human
ESR1_22446102_ChIP-Seq_UTERUS_Mouse
STAT4_19710469_ChIP-ChIP_TH1__Mouse
TCF3/E2A_22897851_ChIP-Seq_JUKARTE6-1_Human
MYC_18358816_ChIP-ChIP_MESCs_Mouse
ERG_20887958_ChIP-Seq_HPC-7_Mouse
SMRT_27268052_Chip-Seq_Bcells_Human
SOX2_19829295_ChIP-Seq_ESCs_Human
BCL6_27268052_Chip-Seq_Bcells_Human
KLF4_19829295_ChIP-Seq_ESCs_Human
E2A_27217539_Chip-Seq_RAMOS-Cell_line_Human
NCOR1_26117541_ChIP-Seq_K562_Human
FOXA1_27270436_Chip-Seq_PROSTATE_Human
GATA3_27048872_Chip-Seq_THYMUS_Human
SA1_27219007_Chip-Seq_Bcells_Human

EBF1_22473956_ChIP-Seq_LYMPHODE_Mouse
GATA3_26560356_Chip-Seq_TH1_Human
TOP2B_26459242_ChIP-Seq_MCF-7_Human
ERA_21632823_ChIP-Seq_H3396_Human
NCOR_22424771_ChIP-Seq_293T_Human
NMYC_18555785_Chip-Seq_ESCs_Mouse
RARB_24833708_ChIP-Seq_LIVER_Mouse
KLF4_18555785_Chip-Seq_ESCs_Mouse
CTCF_26484167_Chip-Seq_Bcells_Mouse
POU3F1_26484290_ChIP-Seq_ESCss_Mouse
NANOG_19829295_ChIP-Seq_ESCs_Human
PBX_27287812_Chip-Seq_EMBYONIC-LIMB_Mouse
FOXA1_25329375_ChIP-Seq_VCAP_Human
GATA3_21878914_ChIP-Seq_MCF-7_Human
LMO2_26923725_Chip-Seq_HEMOGENIC-ENDOTHELIUM_Mouse
KDM2B_26808549_Chip-Seq_DND41_Human
CEBPB_20513432_ChIP-Seq_MACROPHAGES_Mouse
EBF1_22473956_ChIP-Seq_BONE_MARROW_Mouse
P300_27268052_Chip-Seq_Bcells_Human
RUNX2_24764292_ChIP-Seq_MC3T3_Mouse
ELK3_25401928_ChIP-Seq_HUVEC_Human
MYC_27129775_Chip-Seq_CORNEA_Mouse
P53_22387025_ChIP-Seq_ESCs_Mouse
CBP_21632823_ChIP-Seq_H3396_Human
RUNX1_27457419_Chip-Seq_LIVER_Mouse
FOXO1_25302145_ChIP-Seq_T-LYMPHOCYTE_Mouse
TAL1_22897851_ChIP-Seq_JUKARTE6-1_Human
OCT1_27270436_Chip-Seq_PROSTATE_Human
SMAD1_18555785_Chip-Seq_ESCs_Mouse
RAC3_21632823_ChIP-Seq_H3396_Human
GATA2_22383799_ChIP-Seq_G1ME_Mouse
CEBPB_21427703_ChIP-Seq_3T3-L1_Mouse
SA1_27219007_Chip-Seq_ERYTHROID_Human
CEBPB_20176806_ChIP-Seq_MACROPHAGES_Mouse
FOXA1_21572438_ChIP-Seq_LNCaP_Human
ZFX_18555785_Chip-Seq_ESCs_Mouse
PU1_27457419_Chip-Seq_LIVER_Mouse
OCT4_19829295_ChIP-Seq_ESCs_Human
SPI1_26923725_Chip-Seq_MACROPHAGESS_Mouse
SA1_22415368_ChIP-Seq_MEFs_Mouse
EZH2_27294783_Chip-Seq_ESCs_Mouse
CRX_20693478_ChIP-Seq_RETINA_Mouse
UTX_26944678_Chip-Seq_JUKART_Human
FOXO3_22982991_ChIP-Seq_MACROPHAGES_Mouse
SUZ12_18555785_Chip-Seq_ESCs_Mouse
CEBPB_26923725_Chip-Seq_MACROPHAGESS_Mouse
ESRRB_18555785_Chip-Seq_ESCs_Mouse

OCT4_18555785_Chip-Seq_ESCs_Mouse
FOXA1_26769127_Chip-Seq_PDAC-Cell_line_Human
IRF8_27001747_Chip-Seq_BMDM_Mouse
GATA1_22383799_ChIP-Seq_G1ME_Mouse
KDM2B_26808549_Chip-Seq_SUP-B15_Human
ETS1_22383799_ChIP-Seq_G1ME_Mouse
SMAD2/3_21741376_ChIP-Seq_ESCs_Human
STAT3_18555785_Chip-Seq_ESCs_Mouse
RING1B_27294783_Chip-Seq_NPCs_Mouse
KLF6_26769127_Chip-Seq_PDAC-Cell_line_Human
RBPJ_21746931_ChIP-Seq_IB4-LCL_Human
FLI1_20887958_ChIP-Seq_HPC-7_Mouse
RAD21_21589869_ChIP-Seq_MESCs_Mouse
AR_20517297_ChIP-Seq_VCAP_Human
JARID1A_20064375_ChIP-Seq_MESCs_Mouse
STAT3_20064451_ChIP-Seq_CD4+T_Mouse
SETDB1_19884257_ChIP-Seq_MESCs_Mouse
SMAD4_21741376_ChIP-Seq_HESCs_Human
REST_18959480_ChIP-ChIP_MESCs_Mouse
RUNX2_22187159_ChIP-Seq_PCA_Human
