# Supplementary material for: An in-silico approach for discovery of microRNA-TF regulation of DISC1 interactome mediating neuronal migration
Source: NPJ Syst Biol Appl. 2019 May 7;5:17. doi: 10.1038/s41540-019-0094-3 (PMC6504871; doi:10.1038/s41540-019-0094-3)
Supplement: Supplementary file 4 — Supplementary references [file 41540_2019_94_MOESM4_ESM.pdf]

Mechanistic evidence for list of proteins that interact with DISC1 and regulate neurodevelopmental functions

List of interacting proteins that were retrieved from the database (*N*=22)	References for experimental evidence	List of proteins (*N*=65) and non-protein coding gene (*N*=1) retrieved using the literature resource from NCBI PubMed and Google Scholar	References for experimental evidence
ATF4	[1,2]	CTNB1	[3]
ATF5	[1]	BBS4	[4]
NDEL1	[1,5–13]	PCM1	[4]
TRIO	[14]	A4	[11]
MIC60	[15]	DAB1	[11]
GSK3B	[3,5]	KIF5A	[10,16]
CDC141	[17]	CDK5	[9]
PCNT	[18]	DIXC1	[9]
MIPT3	[1]	SOX10	[19]
NDE1	[6,20]	FOXD3	[19]
MAP1A	[1]	MYH2	[17]
FEZ1	[18]	ACTB	[21]
ZN365	[18]	AKT1	[21]
1433E	[10,22]	RAC1	[14,23]
LIS1	[6]	TRAK1	[16]
KALRN	[23,24]	MIRO1	[16]
PDE4B	[6]	GRB2	[25]
GRDN	[26,27]	NLGN1	[23]
TNIK	[28]	NRX1A	[23]
EXOC1	[29]	NMDZ1	[23]
SRR	[30]	ITPR1	[31]
RASF7	[32]	ZNF385A	[31]
		MTOR	[33]
		PACA	[18]
		DTBP1	[34]
		PDE4D	[2]
		BACE1	[35]
		CREB1	[20]
		NMDE1	[20]
		NRG1	[35]
		NRG2	[35]

	OLIG2	[36]
	S12A2	[27]
	ITPR3	[29]
	MIRO2	[37]
	TRAK2	[37]
	MFN1	[37]
	MFN2	[37]
	DISC1FP1 (non-protein coding gene)	[37,38]
	LRRC7	[39]
	GRM5	[39]
	ERBB4	[40]
	ERBB2	[35]
	ERBB3	[35]
	PK3CA	[35]
	RHEB	[41]
	SMO	[42]
	PTC1	[42]
	PTC2	[42]
	DZIP1	[42]
	KIF5C	[43]
	GBRB2	[43]
	GBRB3	[43]
	NCHL1	[44]
	ITB1	[45]
	MK03	[32]
	MP2K1	[32]
	GBRA1	[46]
	TLR3	[47]
	MYD88	[47]
	NPY	[48]
	PDE4B	[49]
	KCNA1	[49]
	NKX22	[50]
	DAAM1	[51]
	RHOA1	[51]

References

1. Morris, J. A., Kandpal, G., Ma, L. & Austin, C. P. DISC1 (Disrupted-In-Schizophrenia 1) is a centrosome-associated protein that interacts with MAP1A, MIPT3, ATF4/5 and NUDEL: regulation and loss of interaction with mutation. *Hum. Mol. Genet.* **12,** 1591–1608 (2003).
2. Soda, T. *et al.* DISC1-ATF4 transcriptional repression complex: dual regulation of the cAMP-PDE4 cascade by DISC1. *Mol. Psychiatry* **18,** 898–908 (2013).
3. Mao, Y. *et al.* Disrupted in schizophrenia 1 regulates neuronal progenitor proliferation via modulation of GSK3beta/beta-catenin signaling. *Cell* **136,** 1017–1031 (2009).
4. Kamiya, A. *et al.* Recruitment of PCM1 to the centrosome by the cooperative action of DISC1 and BBS4: a candidate for psychiatric illnesses. *Arch. Gen. Psychiatry* **65,** 996–1006 (2008).
5. Okamoto, M. *et al.* DBZ regulates cortical cell positioning and neurite development by sustaining the anterograde transport of Lis1 and DISC1 through control of Ndel1 dual-phosphorylation. *J. Neurosci.* **35,** 2942–2958 (2015).
6. Bradshaw, N. J. *et al.* PKA phosphorylation of NDE1 is DISC1/PDE4 dependent and modulates its interaction with LIS1 and NDEL1. *J. Neurosci.* **31,** 9043–9054 (2011).
7. Kamiya, A. *et al.* DISC1-NDEL1/NUDEL protein interaction, an essential component for neurite outgrowth, is modulated by genetic variations of DISC1. *Hum. Mol. Genet.* **15,** 3313–3323 (2006).
8. Shinoda, T. *et al.* DISC1 regulates neurotrophin-induced axon elongation via interaction with Grb2. *J. Neurosci.* **27,** 4–14 (2007).
9. Singh, K. K. *et al.* Dixdc1 is a critical regulator of DISC1 and embryonic cortical development. *Neuron* **67,** 33–48 (2010).
10. Taya, S. *et al.* DISC1 regulates the transport of the NUDEL/LIS1/14-3-3epsilon complex through kinesin-1. *J. Neurosci.* **27,** 15–26 (2007).
11. Young-Pearse, T. L., Suth, S., Luth, E. S., Sawa, A. & Selkoe, D. J. Biochemical and functional interaction of disrupted-in-schizophrenia 1 and amyloid precursor protein regulates neuronal migration during mammalian cortical development. *J. Neurosci.* **30,** 10431–10440 (2010).
12. Brandon, N. J. *et al.* Disrupted in Schizophrenia 1 and Nudel form a neurodevelopmentally regulated protein complex: implications for schizophrenia and other major neurological disorders. *Mol. Cell. Neurosci.* **25,** 42–55 (2004).
13. Ozeki, Y. *et al.* Disrupted-in-Schizophrenia-1 (DISC-1): mutant truncation prevents binding to NudE-like (NUDEL) and inhibits neurite outgrowth. *Proc. Natl. Acad. Sci. U.S.A.* **100,** 289–294 (2003).
14. Chen, S.-Y., Huang, P.-H. & Cheng, H.-J. Disrupted-in-Schizophrenia 1-mediated axon guidance involves TRIO-RAC-PAK small GTPase pathway signaling. *Proc. Natl. Acad. Sci. U.S.A.* **108,** 5861–5866 (2011).

15. Park, Y.-U. *et al.* Disrupted-in-schizophrenia 1 (DISC1) plays essential roles in mitochondria in collaboration with Mitofilin. *Proc. Natl. Acad. Sci. U.S.A.* **107,** 17785–17790 (2010).

16. Ogawa, F. *et al.* DISC1 complexes with TRAK1 and Miro1 to modulate anterograde axonal mitochondrial trafficking. *Hum. Mol. Genet.* **23,** 906–919 (2014).

17. Fukuda, T., Sugita, S., Inatome, R. & Yanagi, S. CAMDI, a Novel Disrupted in Schizophrenia 1 (DISC1)-binding Protein, Is Required for Radial Migration. *Journal of Biological Chemistry* **285,** 40554–40561 (2010).

18. Matsuzaki, S. & Tohyama, M. Molecular mechanism of schizophrenia with reference to disrupted-in-schizophrenia 1 (DISC1). *Neurochem. Int.* **51,** 165–172 (2007).

19. Drerup, C. M., Wiora, H. M., Topczewski, J. & Morris, J. A. Disc1 regulates foxd3 and sox10 expression, affecting neural crest migration and differentiation. *Development* **136,** 2623–2632 (2009).

20. Wei, J. *et al.* Regulation of N-methyl-D-aspartate receptors by disrupted-in-schizophrenia-1. *Biol. Psychiatry* **75,** 414–424 (2014).

21. Steinecke, A., Gampe, C., Nitzsche, F. & Bolz, J. DISC1 knockdown impairs the tangential migration of cortical interneurons by affecting the actin cytoskeleton. *Front Cell Neurosci* **8,** 190 (2014).

22. Ramsey, A. J. *et al.* Impaired NMDA receptor transmission alters striatal synapses and DISC1 protein in an age-dependent manner. *Proc. Natl. Acad. Sci. U.S.A.* **108,** 5795–5800 (2011).

23. Owczarek, S., Bang, M. L. & Berezin, V. Neurexin-Neuroligin Synaptic Complex Regulates Schizophrenia-Related DISC1/Kal-7/Rac1 'Signalosome'. *Neural Plast.* **2015,** 167308 (2015).

24. Hayashi-Takagi, A. *et al.* Disrupted-in-Schizophrenia 1 (DISC1) regulates spines of the glutamate synapse via Rac1. *Nat. Neurosci.* **13,** 327–332 (2010).

25. Shinoda, T. *et al.* DISC1 regulates neurotrophin-induced axon elongation via interaction with Grb2. *J. Neurosci.* **27,** 4–14 (2007).

26. Steinecke, A., Gampe, C., Nitzsche, F. & Bolz, J. DISC1 knockdown impairs the tangential migration of cortical interneurons by affecting the actin cytoskeleton. *Front Cell Neurosci* **8,** 190 (2014).

27. Kim, J. Y. *et al.* Interplay between DISC1 and GABA signaling regulates neurogenesis in mice and risk for schizophrenia. *Cell* **148,** 1051–1064 (2012).

28. Wang, Q. *et al.* The psychiatric disease risk factors DISC1 and TNIK interact to regulate synapse composition and function. *Mol. Psychiatry* **16,** 1006–1023 (2011).

29. Park, S. J. *et al.* Disrupted-in-schizophrenia-1 (DISC1) Regulates Endoplasmic Reticulum Calcium Dynamics. *Sci Rep* **5,** 8694 (2015).

30. Ma, T. M. *et al.* Pathogenic disruption of DISC1-serine racemase binding elicits schizophrenia-like behavior via D-serine depletion. *Mol. Psychiatry* **18,** 557–567 (2013).

31. Tsuboi, D. *et al.* Disrupted-in-schizophrenia 1 regulates transport of ITPR1 mRNA for synaptic plasticity. *Nat. Neurosci.* **18,** 698–707 (2015).

32. Wang, S. *et al.* DISC1 regulates astrogenesis in the embryonic brain via modulation of RAS/MEK/ERK signaling through RASSF7. *Development* **143,** 2732–2740 (2016).

33. Kim, J. Y. *et al.* DISC1 regulates new neuron development in the adult brain via modulation of AKT-mTOR signaling through KIAA1212. *Neuron* **63,** 761–773 (2009).

34. Lee, S.-A. *et al.* Disrupted-in-schizophrenia 1 (DISC1) regulates dysbindin function by enhancing its stability. *J. Biol. Chem.* **290,** 7087–7096 (2015).

35. Seshadri, S. *et al.* Disrupted-in-Schizophrenia-1 expression is regulated by beta-site amyloid precursor protein cleaving enzyme-1-neuregulin cascade. *Proc. Natl. Acad. Sci. U.S.A.* **107,** 5622–5627 (2010).

36. Wood, J. D., Bonath, F., Kumar, S., Ross, C. A. & Cunliffe, V. T. Disrupted-in-schizophrenia 1 and neuregulin 1 are required for the specification of oligodendrocytes and neurones in the zebrafish brain. *Hum. Mol. Genet.* **18,** 391–404 (2009).

37. Norkett, R. *et al.* DISC1-dependent Regulation of Mitochondrial Dynamics Controls the Morphogenesis of Complex Neuronal Dendrites. *J. Biol. Chem.* **291,** 613–629 (2016).

38. Ji, B. *et al.* Inhibition of protein translation by the DISC1-Boymaw fusion gene from a Scottish family with major psychiatric disorders. *Hum. Mol. Genet.* **23,** 5683–5705 (2014).

39. Carlisle, H. J. *et al.* Deletion of densin-180 results in abnormal behaviors associated with mental illness and reduces mGluR5 and DISC1 in the postsynaptic density fraction. *J. Neurosci.* **31,** 16194–16207 (2011).

40. Seshadri, S. *et al.* Interneuronal DISC1 regulates NRG1-ErbB4 signalling and excitatory-inhibitory synapse formation in the mature cortex. *Nat Commun* **6,** 10118 (2015).

41. Kang, E. *et al.* Rheb1 mediates DISC1-dependent regulation of new neuron development in the adult hippocampus. *Neurogenesis (Austin)* **2,** e1081715 (2015).

42. Boyd, P. J., Cunliffe, V. T., Roy, S. & Wood, J. D. Sonic hedgehog functions upstream of disrupted-in-schizophrenia 1 (disc1): implications for mental illness. *Biol Open* **4,** 1336–1343 (2015).

43. Wei, J., Graziane, N. M., Gu, Z. & Yan, Z. DISC1 Protein Regulates γ-Aminobutyric Acid, Type A (GABAA) Receptor Trafficking and Inhibitory Synaptic Transmission in Cortical Neurons. *J. Biol. Chem.* **290,** 27680–27687 (2015).

44. Ren, J., Zhao, T., Xu, Y. & Ye, H. Interaction between DISC1 and CHL1 in regulation of neurite outgrowth. *Brain Res.* **1648,** 290–297 (2016).

45. Hattori, T. *et al.* DISC1 regulates cell-cell adhesion, cell-matrix adhesion and neurite outgrowth. *Mol. Psychiatry* **15,** 778, 798–809 (2010).

46. Saito, A. *et al.* Early postnatal GABAA receptor modulation reverses deficits in neuronal maturation in a conditional neurodevelopmental mouse model of DISC1. *Mol. Psychiatry* **21,** 1449–1459 (2016).

47. Chen, C.-Y., Liu, H.-Y. & Hsueh, Y.-P. TLR3 downregulates expression of schizophrenia gene Disc1 via MYD88 to control neuronal morphology. *EMBO Rep.* **18,** 169–183 (2017).

48. Morosawa, S. *et al.* Neuropeptide Y neuronal network dysfunction in the frontal lobe of a genetic mouse model of schizophrenia. *Neuropeptides* **62,** 27–35 (2017).

49. Crabtree, G. W. *et al.* Alteration of Neuronal Excitability and Short-Term Synaptic Plasticity in the Prefrontal Cortex of a Mouse Model of Mental Illness. *J. Neurosci.* **37,** 4158–4180 (2017).

50. Katsel, P. *et al.* Expression of mutant human DISC1 in mice supports abnormalities in differentiation of oligodendrocytes. *Schizophrenia Research* **130,** 238–249 (2011).

51. De Rienzo, G. *et al.* Disc1 regulates both β-catenin-mediated and noncanonical Wnt signaling during vertebrate embryogenesis. *FASEB J.* **25,** 4184–4197 (2011).

52. Karege, F. *et al.* Association of AKT1 gene variants and protein expression in both schizophrenia and bipolar disorder. *Genes Brain Behav.* **9,** 503–511 (2010).

53. Matthews, P. R., Eastwood, S. L. & Harrison, P. J. Reduced myelin basic protein and actin-related gene expression in visual cortex in schizophrenia. *PLoS ONE* **7,** e38211 (2012).

54. Gadelha, A. *et al.* Plasma Ndel1 enzyme activity is reduced in patients with schizophrenia--a potential biomarker? *J Psychiatr Res* **47,** 657–663 (2013).

55. Tomppo, L. *et al.* Association between genes of Disrupted in schizophrenia 1 (DISC1) interactors and schizophrenia supports the role of the DISC1 pathway in the etiology of major mental illnesses. *Biol. Psychiatry* **65,** 1055–1062 (2009).

56. Nicodemus, K. K. *et al.* Evidence of statistical epistasis between DISC1, CIT and NDEL1 impacting risk for schizophrenia: biological validation with functional neuroimaging. *Hum. Genet.* **127,** 441–452 (2010).

57. Zhou, J. & Parada, L. F. PTEN signaling in autism spectrum disorders. *Curr. Opin. Neurobiol.* **22,** 873–879 (2012).

58. Bruno, D. L. *et al.* Further molecular and clinical delineation of co-locating 17p13.3 microdeletions and microduplications that show distinctive phenotypes. *J. Med. Genet.* **47,** 299–311 (2010).

59. Fukuda, T. *et al.* Rescue of CAMDI deletion-induced delayed radial migration and psychiatric behaviors by HDAC6 inhibitor. *EMBO Rep.* **17,** 1785–1798 (2016).

60. Chen, J. *et al.* The GSK3B gene confers risk for both major depressive disorder and schizophrenia in the Han Chinese population. *J Affect Disord* **185,** 149–155 (2015).

61. Iwamoto, K. *et al.* DNA methylation status of SOX10 correlates with its downregulation and oligodendrocyte dysfunction in schizophrenia. *J. Neurosci.* **25,** 5376–5381 (2005).

62. Mines, M. A., Yuskaitis, C. J., King, M. K., Beurel, E. & Jope, R. S. GSK3 influences social preference and anxiety-related behaviors during social interaction in a mouse model of fragile X syndrome and autism. *PLoS ONE* **5,** e9706 (2010).
